# Supplementary material for: Next generation thiazolyl ketone inhibitors of cytosolic phospholipase A2 α for targeted cancer therapy
Source: Nat Commun. 2025 Jan 2;16:164. doi: 10.1038/s41467-024-55536-9 (PMC11696576; doi:10.1038/s41467-024-55536-9)
Supplement: Supplementary file 1 — Supplementary Information [file 41467_2024_55536_MOESM1_ESM.pdf]

Contents

Supporting Data **4**

Figure S1. Ex vivo suppression of eicosanoid production. 4

Figure S2. Binding mode of GK420 in cPLA2α binding pocket after MD simulation 5

Figure S3. LC-HRMS analysis of AVX420 in human plasma. 6

Figure S4. Higher selectivity of AVX420 in the cancer cell lines screen. 7

Figure S5. Comparative profiling of cPLA$_2$α inhibitors 8

Table S1. Compounds in the comparative profiling study 9

Table S2. List of the compounds that reliably clustered with cPLA$_2$α inhibitors 10

Table S3. Genetic modifications associated with AVX420 sensitivity 11

Table S4: Cancer-related genes that correlated with sensitivity to AVX420 12

Figure S6. *PLA2G4A* expression and sensitivity to cPLA$_2$α inhibitors. 13

Table S5. Genes associated with resistance to cPLA$_2$α inhibition 14

Figure S7. Sensitivity of cell lines derived from hematological malignancies. 15

Figure S8. AVX420 does not affect the viability of peripheral blood mononuclear cells. 16

Figure S9. Intracellular ROS accumulation and cell death. 17

Table S6. Differentially expressed genes 18

Table S7. Differential ribosomal occupancy 19

Table S8. Gene set enrichment analysis performed on ribosomal occupancy data 20

Table S9. Gene set enrichment analysis performed on differential expression data 21

Supplemental Methods **22**

Synthesis of Inhibitors **22**

Cancer cell line screening **49**

Cell proliferation assays 49

Compound activity profiling 50

Identification of genetic modifications associated with inhibitor sensitivity. 50

Correlation of gene expression with drug response. 51

Gene set enrichment and overlap analyses. 51

RNA-Seq and ribosome-associated RNA(Ribo)-Seq **52**

HPLC chromatograms of inhibitors **53**

Figure S9. HPLC chromatogram of inhibitor **17a** 53

Figure S10. HPLC chromatogram of inhibitor 17b 53

Figure S11. HPLC chromatogram of inhibitor 17c 54

Figure S12. HPLC chromatogram of inhibitor **17d** 54

Figure S13. HPLC chromatogram of inhibitor **17g** 55

Figure S14. HPLC chromatogram of inhibitor **17h** 55

Figure S15. HPLC chromatogram of inhibitor **17i** 56

^{1}H and ^{13}C NMR Traces **57**

Figure S16. 11b......57
Figure S17. 11d......58
Figure S18. 11e......60
Figure S19. 11f......61
Figure S20. 11g......62
Figure S21. 11h......63
Figure S22. 11i......64
Figure S23. 11j......65
Figure S24. 11b......66
Figure S25. 12d......67
Figure S26. 12e......69
Figure S27. 12g......70
Figure S28. 12h......71
Figure S29. 12i......72
Figure S30. 12j......73
Figure S31. 13b......74
Figure S32. 13h......75
Figure S33. 13j......76
Figure S34. 14a......77
Figure S35. 14b......78
Figure S36. 14d......79
Figure S37. 14e......81
Figure S38. 14h......82
Figure S39. 14j......83
Figure S40. 15a......84
Figure S41. 15b......85
Figure S42. 15c......86
Figure S43. 15d......87
Figure S44. 15e......89
Figure S45. 15f......90
Figure S46. 15g......91
Figure S47. 15h......92
Figure S48. 15j......93
Figure S49. 16a......94
Figure S50. 16c......95
Figure S51. 16d......96
Figure S52. 16e......98
Figure S53. 16f......99
Figure S54. 16g......100
Figure S55. 16i......101

Figure S56. 16j 102
Figure S57. 17a 103
Figure S58. 17b 104
Figure S59. 17c 105
Figure S60. 17d 106
Figure S61. 17e 107
Figure S62. 17f 108
Figure S63. 17g 109
Figure S64. 17h 110
Figure S65. 17i 111
Figure S66. 17j 112
Figure S67. 18a 113
Figure S68. 18b 114
Figure S69. 18c 115
Figure S70. 18d 116
Supplemental references 117

Supporting Data

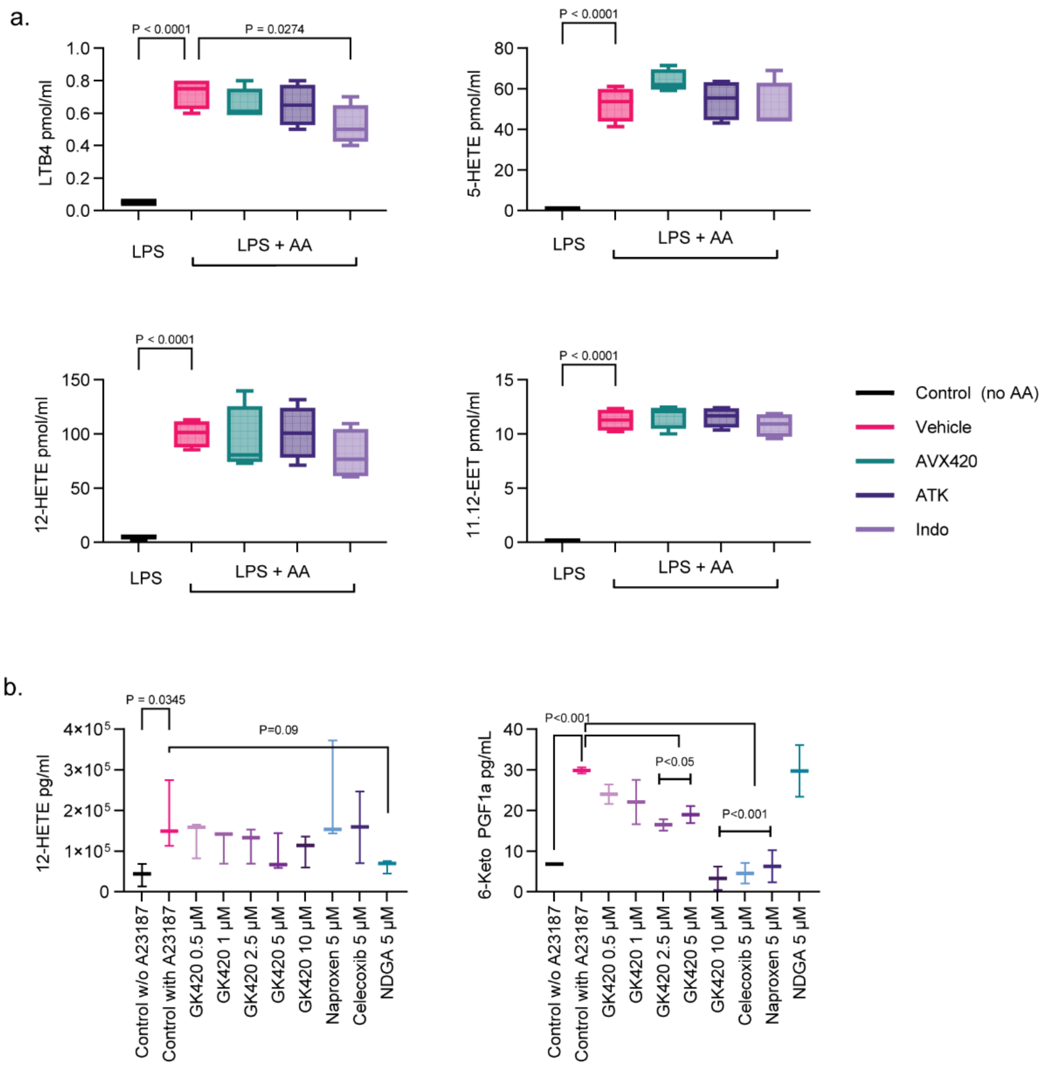

Figure S1. Ex vivo suppression of eicosanoid production. **a.** Eicosanoid levels (pmol/mL) in the plasma of whole blood pretreated with vehicle or inhibitor (10 µM AVX420, 10 µM ATK, or 1 µM indomethacin) before stimulation with 1 µg/mL LPS (24 h) and 5 µM arachidonic acid (AA). Box and whisker plots show the mean (line), stdev (box), and min and max values (whiskers) from 4 biological replicates. P values were determined using RM-one way ANOVA with Dunnett's correction for multiple comparisons. **b.** Eicosanoid levels in the supernatants of isolated human PBMCs pretreated with vehicle (DMSO), GK420, naproxen, celecoxib, or NDGA for 90 min before the addition of A23187 (30 µM, 15 min). Box and whisker plots show the mean (line), stdev (box), and min and max values (whiskers) from 2 and 3 biological replicates for 6-Keto PGF1α and 12-HETE repectively. P values were determined using RM one-way ANOVA with Dunnett's correction for multiple testing.

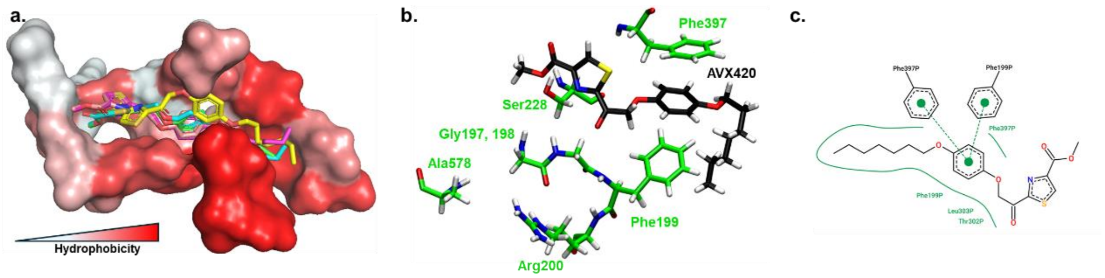

Figure S2. Binding mode of GK420 in cPLA2α binding pocket after MD simulation. **a.** The docking poses with the five lowest docking scores in the substrate binding site of cPLA$_2$α are shown. The binding site is shown as a surface and colored based on the Eisenberg scale of the amino acids.[1] **b.** The 3D binding mode of the compound after 200 ns representative MD simulation is shown. **c.** The 2D interaction map of the compound after 200 ns representative MD simulation is shown.

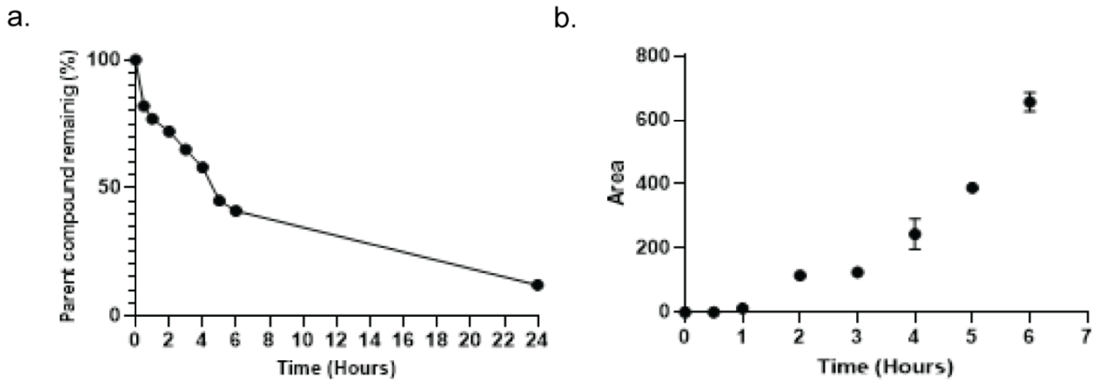

Figure S3. LC-HRMS analysis of AVX420 in human plasma. a. The stability of AVX420 in human plasma monitored for 24 hours by LC-HRMS b. The generation of the free acid form of AVX420 measured by LC-HRMS. Data are mean of 3 biological replicates ± stdev.

Instrumentation: An ABSciex Triple TOF 4600 combined with a micro-LC Eksigent and an autosampler set at 5 ˚C and a thermostated column compartment were used to perform the LC-HRMS measurements. Electrospray ionization (ESI) in positive (for GK420) and negative (for GK420 acid) mode was used for the MS experiments. The data acquisition method consisted of a TOF-MS full scan *m/z* 50–850 and several information dependent acquisition (IDA)-TOF-MS/MS product ion scans using 40 V collision energy (CE) with 15 V collision energy spread (CES) used for each candidate ion in each data acquisition cycle (1091). The MS resolution working conditions were: ion energy 1 (IE1) −2.3, vertical steering (VS1) −0.65, horizontal steering (HST) 1.15 and vertical steering 2 (VS2) 0.00. A Halo C18 2.7 µm, 90 Å, 0.5 × 50 mm column from Eksigent was used for the present study. The mobile phase system consisted of solvent A: H_2O/0.01% and solvent B: acetonitrile/0.01% formic acid/isopropanol 80/20 v/v. The gradient elution program was as follows: 0–0.5 min, 5% B; 0.5–8.0 min, gradually increasing to 98% B; 8.0–8.5 min, 98% B, followed by a 1.5 min equilibration step to the initial conditions prior to the next injection. The injection volume was set at 5 µL and the flow rate at 55 µL/min. MultiQuant 3.0.2 and PeakView 2.1 (ABSciex, Darmstadt, Germany) were employed for the data acquisition. EICs were obtained creating the base peak chromatograms for masses that achieve a 0.01 Da mass accuracy width. The integration of the peak areas was performed manually using MultiQuant 3.0.2.

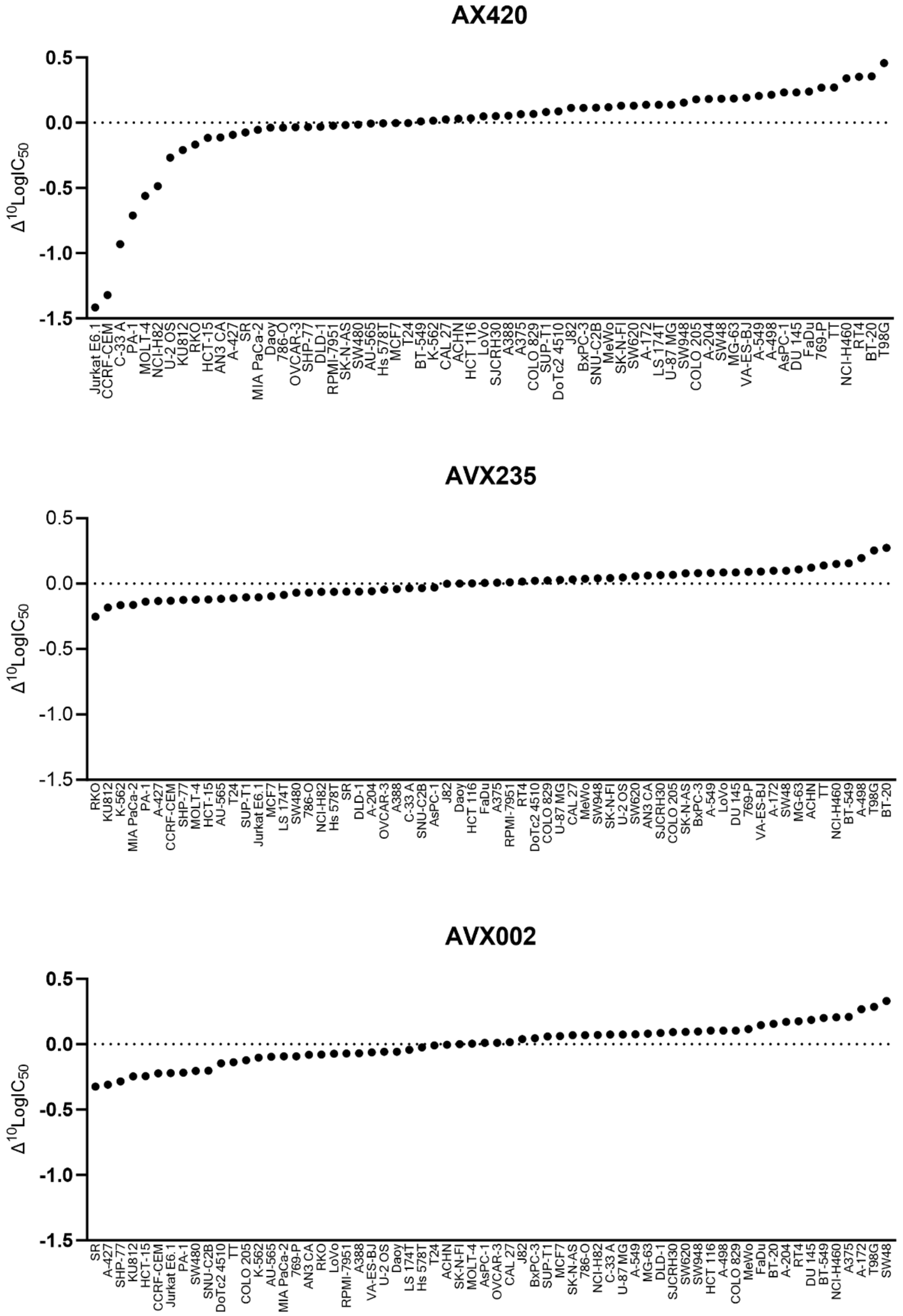

Figure S4. Higher selectivity of AVX420 in the cancer cell lines screen. The charts depict $^{10}\log IC_{50}$ values for each cell line relative to the average $^{10}\log IC_{50}$ measured in the panel. A number of -1 on the y axis reflects a 10x lower IC_{50} compared to the panel average for that compound. The average panel $^{10}\log IC_{50}$ (IC_{50} in nM) in this study was 4.17, 4.20 and 3.98, corresponding to IC_{50} values of 18.4, 16.3 and 10.2 µM for AVX420, AVX235, and AVX002 respectively. Source data are provided.

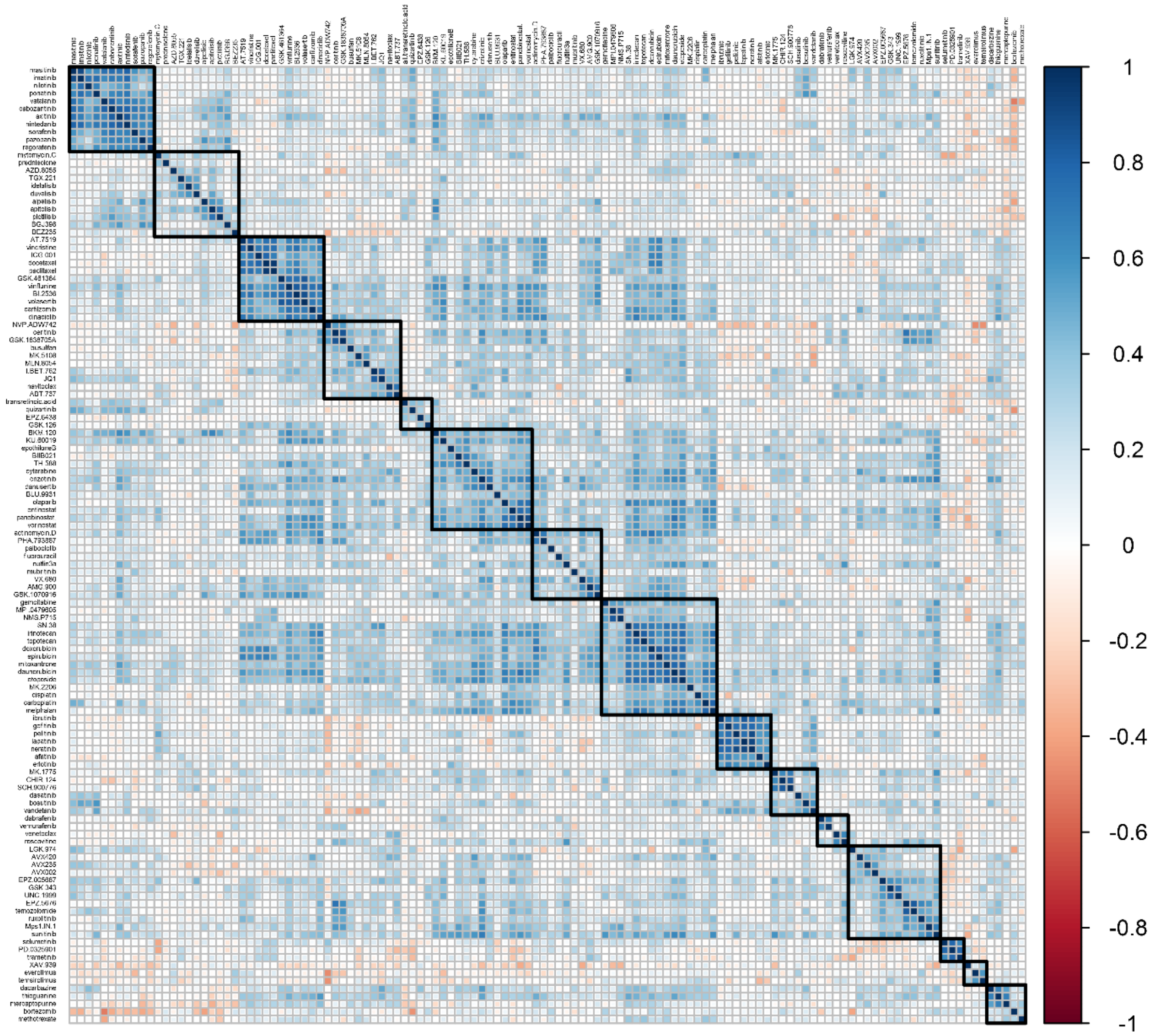

Figure S5. Comparative profiling of cPLA$_2\alpha$ inhibitors. Correlation matrix shows the Pearson correlations corr (a,b) between all compound pairs. Rows and columns were ordered according to the results of unsupervised hierarchical clustering to highlight similarities. The color legend on the right indicates matching values for the Pearson correlation coefficients. Reliable clusters, defined by bootstrapping, are outlined in bold.

Table S1. Compounds in the comparative profiling study

compound name	main target	source
**ABT-737**	BCL2	Selleck Chemicals
**actinomycin-D**	transcription	Sigma-Aldrich
**afatinib**	EGFR	Selleck Chemicals
**all-trans retinoic acid**	RAR	Sigma-Aldrich
**alpelisib**	PI3Ka	Selleck Chemicals
**AMG-900**	Aurora kinases	Selleck Chemicals
**apitolisib**	PI3K	Selleck Chemicals
**AT-7519**	CDK	Selleck Chemicals
**axitinib**	VEGFR / PDGFR	Selleck Chemicals
**AZD-8055**	mTOR	Selleck Chemicals
**BEZ235**	PI3K / mTOR	Selleck Chemicals
**BGJ-398**	FGFR	Selleck Chemicals
**BI-2536**	PLK1	synthesized at NTRC
**BIIB021**	HSP90	Selleck Chemicals
**BKM-120**	PI3K	Selleck Chemicals
**BLU-9931**	FGFR4	Selleck Chemicals
**bortezomib**	proteasome	LC Laboratories
**bosutinib**	ABL	Selleck Chemicals
**busulfan**	DNA alkylating	Sigma-Aldrich
**cabozantinib**	MET/VEGFR	Selleck Chemicals
**carboplatin**	DNA damage	MedKoo
**carfilzomib**	proteasome	MedKoo
**ceritinib**	EGFR	Selleck Chemicals
**CHIR-124**	CHK1	Selleck Chemicals
**cisplatin**	DNA damage	Sigma-Aldrich
**crizotinib**	ALK / MET	Selleck Chemicals
**cytarabine**	nucleoside analog	Sigma-Aldrich
**dabrafenib**	RAF	Selleck Chemicals
**dacarbazine**	DNA alkylating	Sigma-Aldrich
**danusertib**	Aurora kinases	Selleck Chemicals
**dasatinib**	ABL / VEGFR	LC Laboratories
**daunorubicin**	topoisomerase II	Sigma-Aldrich
**dinaciclib**	CDK	Selleck Chemicals
**docetaxel**	tubulin	LC Laboratories
**doxorubicin**	topoisomerase II	LC Laboratories
**duvelisib**	PI3K	Selleck Chemicals
**entinostat**	HDAC	Axon Medchem
**epirubicin**	Topoisomerase II	Selleck Chemicals
**epothilone B**	tubulin	Selleck Chemicals
**EPZ-005687**	EZH2	Selleck Chemicals
**EPZ-5676**	DOT1L	Selleck Chemicals
**EPZ-6438**	EZH2	Selleck Chemicals
**erlotinib**	EGFR	LC Laboratories
**etoposide**	topoisomerase II	Sigma-Aldrich
**everolimus**	mTOR	Selleck Chemicals
**fluorouracil**	nucleoside analog	Sigma-Aldrich
**gefitinib**	EGFR	LC Laboratories
**gemcitabine**	nucleoside analog	Selleck Chemicals
**GSK-1070916**	Aurora kinases	Selleck Chemicals
**GSK-126**	EZH2	Selleck Chemicals
**GSK-1838705A**	IGF1R	Selleck Chemicals
**GSK-343**	EZH2	Selleck Chemicals
**GSK-461364**	PLK1	Selleck Chemicals
**I-BET-762**	BET	Selleck Chemicals
**ibrutinib**	BTK	Axon Medchem
**ICG-001**	Wnt-pathway	Selleck Chemicals
**idelalisib**	PI3K	Selleck Chemicals
**imatinib**	ABL	LC Laboratories
**irinotecan**	topoisomerase I	LC Laboratories
**JQ1**	BET	Selleck Chemicals
**KU-60019**	ATM	Selleck Chemicals

compound name	main target	source
**lapatinib**	EGFR	LC Laboratories
**LGK-974**	PORCN	Selleck Chemicals
**masitinib**	KIT	Selleck Chemicals
**melphalan**	DNA alkylating	Sigma-Aldrich
**mercaptopurine**	nucleside analog	Selleck Chemicals
**methotrexate**	folate synthesis	Tocris Bioscience
**mitoxantrone**	topoisomerase II	Selleck Chemicals
**MK-1775**	WEE1	Selleck Chemicals
**MK-2206**	AKT	Selleck Chemicals
**MK-5108**	Aurora kinases	Selleck Chemicals
**MLN-8054**	Aurora kinases	Selleck Chemicals
**MPI-0479605**	TTK	synthesized at NTRC
**Mps1-IN-1**	TTK	Tocris Bioscience
**mubritinib**	ERBB2	Selleck Chemicals
**mytomycin-C**	DNA crosslinking	Sigma-Aldrich
**navitoclax**	BCL2	Selleck Chemicals
**neratinib**	EGFR	Selleck Chemicals
**nilotinib**	ABL	Axon Medchem
**nintedanib**	VEGFR/FGFR	Selleck Chemicals
**NMS-P715**	TTK	synthesized at NTRC
**nutlin 3a**	MDM2	Selleck Chemicals
**NVP-ADW742**	IGF1R	Selleck Chemicals
**olaparib**	PARP	LC Laboratories
**paclitaxel**	tubulin	Sigma-Aldrich
**palbociclib**	CDK	Selleck Chemicals
**panobinostat**	HDAC	Axon Medchem
**pazopanib**	VEGFR / PDGFR	Selleck Chemicals
**PD-0325901**	MEK	Selleck Chemicals
**pelitinib**	EGFR	Selleck Chemicals
**PHA-793887**	CDK	Selleck Chemicals
**pictilisib**	PI3K	Selleck Chemicals
**ponatinib**	ABL	Selleck Chemicals
**prednisolone**	GR	Sigma-Aldrich
**quizartinib**	FLT3	Selleck Chemicals
**regorafenib**	VEGFR / PDGFR	Selleck Chemicals
**roscovitine**	CDK	Fluorochem
**ruxolitinib**	JAK2 / JAK3	Selleck Chemicals
**SCH-900776**	CHK1	Selleck Chemicals
**selumetinib**	MEK	Selleck Chemicals
**SN-38**	topoisomerase I	Selleck Chemicals
**sorafenib**	VEGFR / PDGFR	LC Laboratories
**sunitinib**	VEGFR / PDGFR	LC Laboratories
**temozolomide**	DNA alkylating	Selleck Chemicals
**temsirolimus**	mTOR	Selleck Chemicals
**TGX-221**	PI3K	Selleck Chemicals
**TH-588**	MTH1	Axon Medchem
**thioguanine**	nucleoside analog	Sigma-Aldrich
**topotecan**	topoisomerase I	MedKoo
**trametinib**	MEK	Selleck Chemicals
**UNC-1999**	EZH1 / EZH2	Selleck Chemicals
**vandetanib**	VEGFR / PDGFR	Selleck Chemicals
**vatalanib**	VEGFR	Selleck Chemicals
**vemurafenib**	RAF	Selleck Chemicals
**venetoclax**	BCL2	Selleck Chemicals
**vincristine**	tubulin	Selleck Chemicals
**vinflunine**	tubulin	Selleck Chemicals
**volasertib**	PLK1	Selleck Chemicals
**vorinostat**	HDAC	Selleck Chemicals
**VX-680**	Aurora kinases	synthesized at NTRC
**XAV-939**	TNKS (tankyrase)	Axon Medchem

Table S2. List of the compounds that reliably clustered with $cPLA_2\alpha$ inhibitors

Drug	Target
LGK.974	PORCN
AVX420	cPLA2
AVX235	cPLA2
AVX002	cPLA2
EPZ.005687	EZH2
GSK.343	EZH2
UNC.1999	EZH1/EZH2
EPZ.5676	DOTL1
Temozolomide	DNA alkylating
Ruxolitinib	JAK2/JAK3
Mps1.IN.1	TTK
sunitinib	VEGFR/PDGFR

Table S3. Genetic modifications associated with AVX420 sensitivity. A negative IC_{50} shift indicates that the compound is more potent in cell lines that carry the mutated gene. Bold text indicates gene modifications that were also associated with sensitivity to AVX235.

Cancer Gene	IC50 shift	p-value	adj. P-value
ARHGAP35	-0.75	3.55E-06	3.77E-04
**MLL**	-0.65	1.15E-05	6.09E-04
NRAS	-0.55	2.96E-04	0.01
PTEN	-0.39	3.03E-04	0.01
DNMT1	-0.69	3.41E-04	0.01
POLE	-0.69	3.63E-04	0.01
CDK12	-0.64	1.00E-03	0.01
MLL2	-0.33	1.01E-03	0.01
FLT3	-0.63	1.10E-03	0.01
FBXW7	-0.37	1.84E-03	0.02
EGFR	-0.4	2.39E-03	0.02
ING1	-0.49	3.77E-03	0.03
ERBB3	-0.44	4.41E-03	0.04
ARID1B	-0.43	1.00E-02	0.09
SMC3	-0.43	1.00E-02	0.09
SMARCA4	-0.33	1.00E-02	0.09
ARID1A	-0.31	2.00E-02	0.12
CREBBP	-0.46	2.00E-02	0.12
**ASXL1**	-0.33	2.00E-02	0.12
TSC1	-0.35	3.00E-02	0.13
MET	-0.35	3.00E-02	0.15

Table S4: Cancer-related genes that correlated with sensitivity to AVX420 at an FDR of 5%, (adjusted P value < 0.05).

Gene	cor.pearson	p.value.pearson	adjusted.p
TSHR	-0.78082	2.06E-12	7.42E-10
IKZF1	-0.61768	5.07E-07	7.82E-05
RUNX1	-0.61308	6.50E-07	7.82E-05
CD1D	-0.60461	1.02E-06	9.17E-05
ACVR2B	-0.58134	3.25E-06	0.000235
PBRM1	-0.48649	0.000166	0.006005
CHD1	-0.48125	0.0002	0.006005
RAD21	-0.47126	0.000282	0.007823
MDM4	-0.46864	0.000308	0.007938
WHSC1L1	-0.46031	0.000406	0.008903
BAP1	-0.45934	0.000419	0.008903
SPEN	-0.45502	0.000483	0.009678
EZH2	-0.45028	0.000562	0.010672
PHF6	-0.44432	0.000678	0.012239
DNMT3A	-0.4373	0.000843	0.014131
TDRD9	-0.43658	0.000861	0.014131
KAT6A	-0.42641	0.001169	0.018355
DIAPH1	-0.42469	0.00123	0.018466
UHRF2	-0.42278	0.001302	0.018466
E2F3	-0.42075	0.001381	0.018466
BCOR	-0.40943	0.001909	0.024617
IKZF2	-0.40581	0.002113	0.02608
MGA	-0.4049	0.002167	0.02608
MED23	-0.39764	0.002645	0.02884
ARID1A	-0.39749	0.002656	0.02884
RBM10	-0.39632	0.002741	0.02884
FAM123B	-0.39495	0.002844	0.02884
CTCF	-0.39454	0.002876	0.02884
BRCA2	-0.38315	0.003886	0.037912

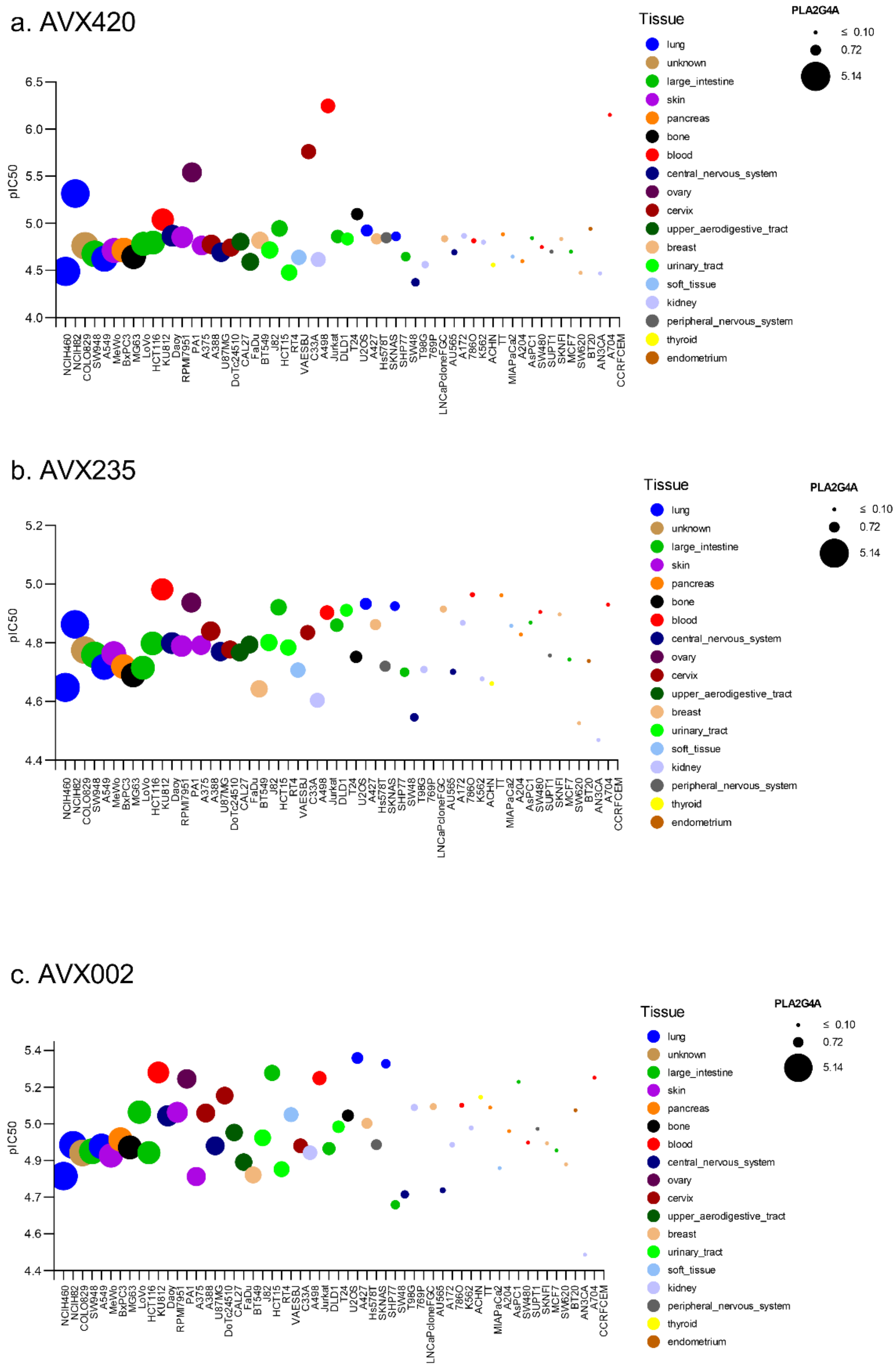

Figure S6. *PLA2G4A* expression and sensitivity to cPLA$_2$α inhibitors. The pIC$_{50}$ of the inhibitor is plotted on the y-axis for each cell line ordered by expression of *PLA2G4A* (with the highest on the left). The size of the bubble is relative to *PLA2G4A* expression, and the color indicates the tissue of origin of the cell line

Table S5. Genes associated with resistance to cPLA$_2$α inhibition**.** The sigma score (sigma.score) corresponds to the number of standard deviations a particular gene-compound correlation stands out above or below the mean for the compound library. Positive sigma scores with a $P<0.05$ were considered significant and genes associated with resistance to AVX420 and at least one other cPLA$_2$α inhibitor are listed. Sigma scores for AVX420 are shown.

gene	sigma.score	p.value.sigma		gene	sigma.score	p.value.sigma
PIR	4.01	6.05E-05		AKR1B10	2.25	2.44E-02
CES2	3.57	3.55E-04		GTF2I	2.23	2.56E-02
UBC	3.37	7.51E-04		IFI35	2.19	2.86E-02
NFE2L2	3.30	9.56E-04		UGDH	2.18	2.96E-02
ALDH3A1	2.86	4.26E-03		MSH6	2.17	3.02E-02
EPHX1	2.83	4.65E-03		TBXAS1	2.17	3.03E-02
C9orf3	2.80	5.07E-03		HSD17B2	2.17	3.03E-02
AKR1C2	2.69	7.20E-03		TNFRSF1A	2.16	3.08E-02
SLC16A3	2.68	7.30E-03		HARS2	2.13	3.34E-02
LOC344887	2.63	8.43E-03		C2orf18	2.13	3.35E-02
ITPRIPL2	2.51	1.19E-02		RETSAT	2.11	3.52E-02
MYO1E	2.43	1.50E-02		G6PC3	2.08	3.74E-02
GPC1	2.42	1.54E-02		C1RL	2.08	3.76E-02
AVPI1	2.41	1.60E-02		NCSTN	2.08	3.78E-02
HLA-G	2.40	1.62E-02		SRC	2.06	3.94E-02
ABCC3	2.39	1.67E-02		PCBP1	2.04	4.12E-02
ACVRL1	2.39	1.68E-02		CD44	2.04	4.16E-02
LYNX1	2.37	1.76E-02		ARF1	2.02	4.31E-02
MTMR11	2.37	1.77E-02		SLC30A5	2.02	4.33E-02
ADSS	2.36	1.83E-02		CRYBA1	2.02	4.35E-02
DEFB1	2.35	1.86E-02		ATOH1	2.01	4.48E-02
SNTB2	2.35	1.87E-02		LOC400550	2.00	4.57E-02
BCL3	2.35	1.88E-02		AMIGO2	1.99	4.63E-02
SHC1	2.35	1.90E-02		RAB11B	1.99	4.67E-02
HAS3	2.33	1.98E-02		SULF2	1.98	4.80E-02
RARG	2.32	2.03E-02		LOC100134713	1.98	4.81E-02
LURAP1L	2.31	2.12E-02		GPR64	1.97	4.83E-02
AKR1C3	2.28	2.26E-02		CYP4F11	1.97	4.84E-02
SAMD5	2.26	2.38E-02		ADM	1.96	4.98E-02
AKR1B10	2.25	2.44E-02				

a.

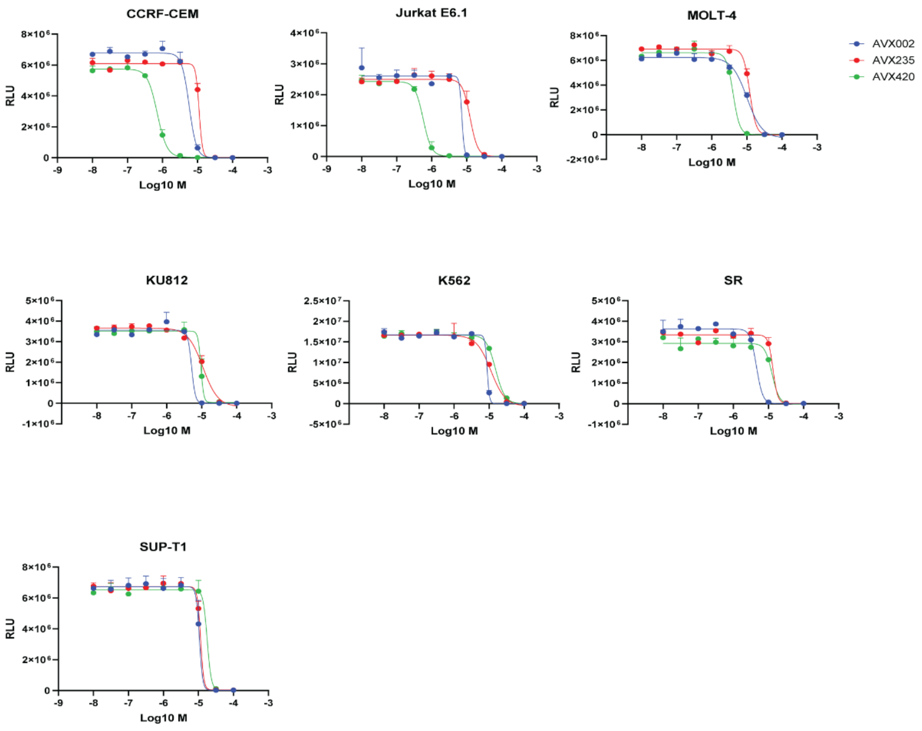

b.

Cell Line	Disease origin	IC_{50} (nM) AVX420	IC_{50} (nM) AVX235	IC_{50} (nM) AVX002
Jurkat E6.1	Leukemia, T lymphocyte	569	12489	5769
CCRF-CEM	Acute T lymphoblastic leukemia	708	11738	5745
MOLT-4	Acute lymphoblastic leukemia	4069	11950	9675
KU812	Chronic myelogenous leukemia	9134	10434	5442
SR	Large cell immunoblastic lymphoma	12476	13786	4551
K562	Chronic myelogenous leukemia	15334	10870	7571
SUP-T1	T-cell lymphoblastic lymphoma	17900	12454	11009

c.

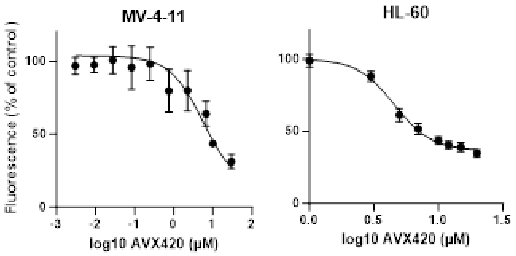

Figure S7. Sensitivity of cell lines derived from hematological malignancies. **a.** cell viability expressed as relative light units (RLU) is plotted for all cell lines in the screen that were derived from hematological malignancies. **b.** corresponding IC_{50} values calculated by non-linear regression analysis. **c**. Cell viability assessed by resazurin assays in MV-4-11 and HL-60 cells. Data were normalized to the vehicle-treated control (100%) and are the mean of 4 biological replicates ±stdev (MV-4-11) and 5 biological replicates ±stdev (HL-60), Non-linear regression analysis was performed in GraphPad Prism v 10.0.2 giving IC_{50} values of 5700 nM and 4600 nM for MV-4-11 and HL-60 respectively.

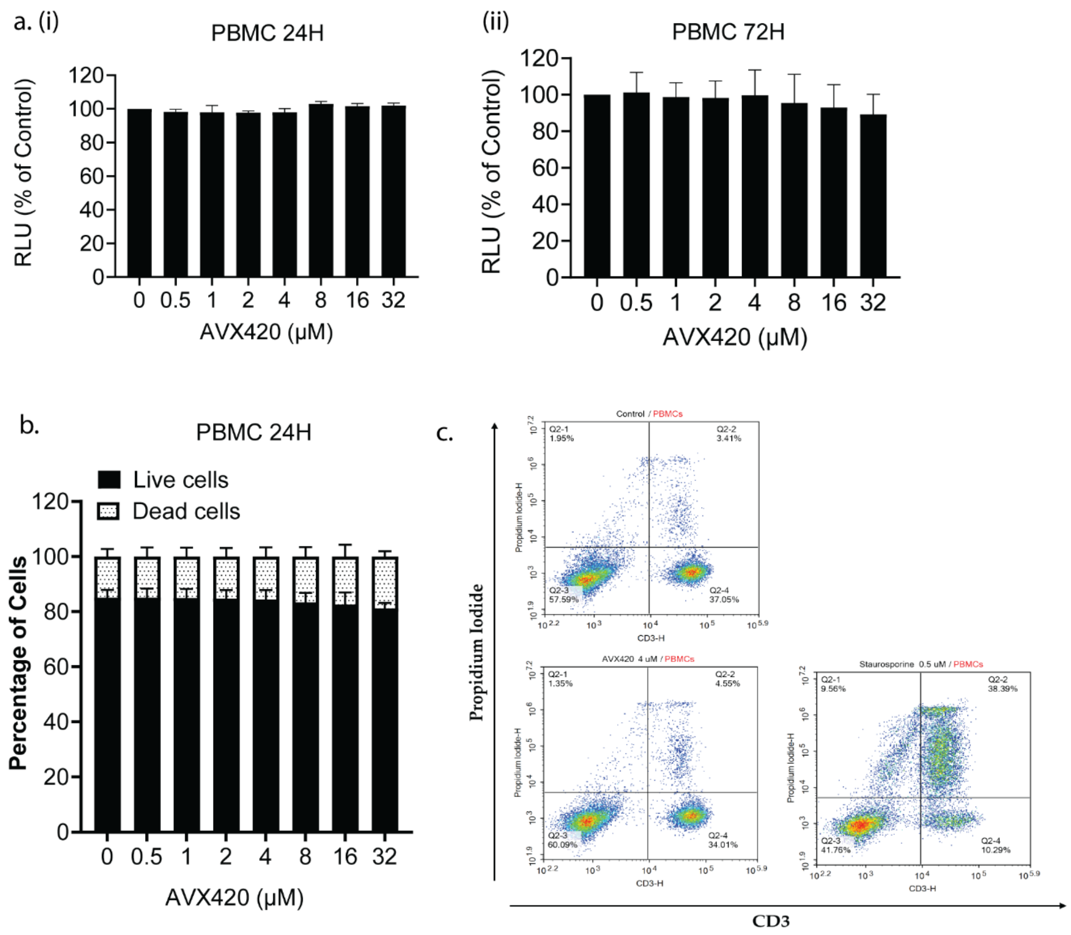

Figure S8. AVX420 does not affect the viability of peripheral blood mononuclear cells. **a**. CTG assays were used to assess viability of PBMCs isolated from human blood after treatment with vehicle control (DMSO) or AVX420 at the indicated doses for either (i) 24 hours or (ii) 72 hours. Data are the mean ±stdev of 4 or 5 biological replicates respectively. **b**. The propidium iodide exclusion assay was used to assess viability of ungated PBMCs treated with AVX420 at the indicated doses for 24 hours. Data are the mean ±stdev from 8 biological replicates (1-16 µM) and 5 biological replicates (32 µM). **c**. Representative dots plots to demonstrate the analysis of propidium iodide exclusion in CD3+ lymphocytes (T-cells). Cells appearing in the bottom right quadrant are PI-negative (live) T-cells while cells in the top right quadrant are PI-positive (dead) T-cells.

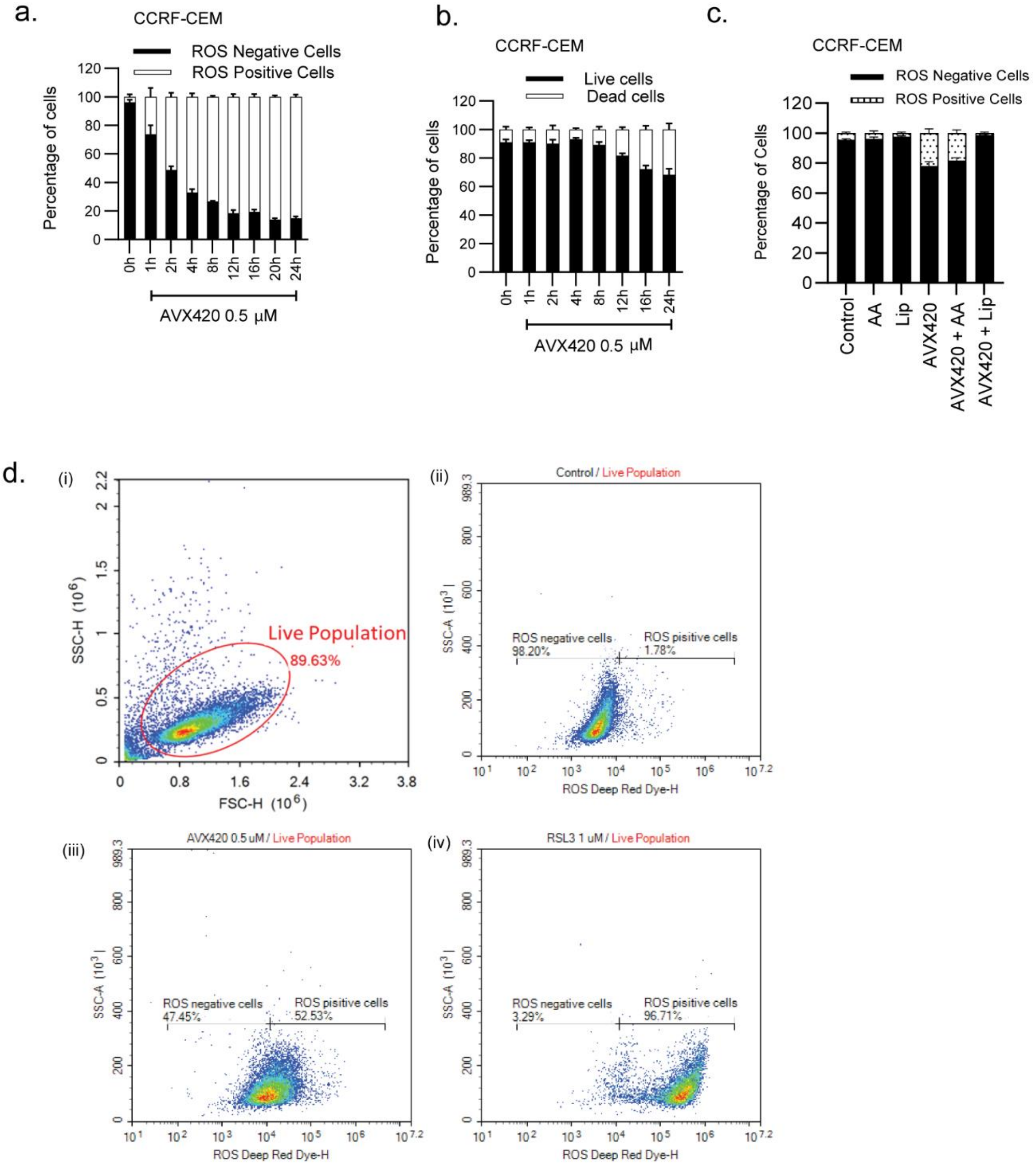

Figure S9. Intracellular ROS accumulation and cell death. **a.** Time dependency of the increase in iROS following treatment with AVX420 in CCRF-CEM cells as measured by ROS-red staining. Data are the mean ±stdev from ≥2 biological replicates **b.** Time-dependency of cell death induced by AVX420 in CCRF-CEM cells as measured by PI exclusion and flow cytometry. Data are the mean ±stdev of ≥2 biological replicates **c.** intracellular ROS measurements in CCRF-CEM cells treated with 0.5 µM AVX420 in the presence of 20 µM arachidonic acid (AA) or 5 µM liproxstatin (Lip) for 4 hours. Data are the mean ± stdev of 2 biological replicates. d. Example dots plots to demonstrate the analysis of ROS Red staining by flow cytometry. (i) Gating of live cells using FSC-H versus SSC-H. (ii) Gating based on ROS Deep Red staining under control conditions and after treatment with (iii) AVX420 or (iv) RLS3.

Table S6. Differentially expressed genes after 16 hours of treatment with 0.5 μM AVX420 using a P-value cut off of 0.01 after multiple testing adjustment (padj) and a log2 fold change >1 or <-1. The top 20 up-regulated and all down-regulated genes are shown, while a list of all differentially expressed genes can be found in the source data file.

Gene	log2FoldChange	padj
CHAC1	3.51	1.63E-43
LINC02970	3.49	7.91E-04
GPT2	3.48	2.51E-14
SLC7A11	3.42	2.17E-105
OSGIN1	3.08	1.67E-10
L3MBTL2-AS1	3.01	5.22E-07
SLC6A9	2.93	3.47E-16
HRG	2.92	9.16E-04
NMRAL2P	2.89	1.56E-06
RFPL1S	2.61	4.30E-03
MIR22HG	2.58	6.78E-03
SESN2	2.53	4.17E-41
TM6SF1	2.49	4.76E-18
VLDLR-AS1	2.44	4.42E-15
B4GALNT1	2.37	1.06E-06
ADM2	2.31	3.43E-22
NOL3	2.29	2.34E-05
ENSG00000260498	2.28	6.66E-04
NDUFA4L2	2.19	2.47E-04
IFRD1	2.08	1.01E-03
RPL23AP42	-2.03	8.39E-03
THOC3	-1.74	7.46E-03
CAMKK1	-1.18	8.94E-03
MYT1	-1.13	1.82E-05
RAG1	-1.12	8.71E-13
LRRTM1	-1.11	3.01E-03
AQP3	-1.1	6.96E-04
CAMKV	-1.07	2.92E-05
DUSP2	-1.04	7.53E-07
PNMA1	-1.02	6.74E-03

Table S7. Differential ribosomal occupancy after 1 hour treatment with 100 nM Torin1 using a P-value cut off of < 0.01 after multiple testing adjustment (padj) and a log2(FC) of >1 or <-1. Log2 fold change of differentially expressed genes from the IP library (log2fc.Ribo), RNA-seq library (log2fc.RNA) and the ribosomal occupancy (log2fc.RO) are shown.

gene_name	log2fc.Ribo	log2fc.RNA	log2fc.RO	padj
ENSG00000226484	-2.12	0.00	-2.12	3.32E-03
ENSG00000260983	-1.00	0.36	-1.36	4.33E-04
RPS3	-1.24	0.11	-1.35	3.83E-08
EEF1G	-1.37	-0.03	-1.34	3.04E-14
RPS15A	-1.15	0.15	-1.29	2.31E-07
RPL34	-1.31	-0.04	-1.27	1.12E-09
RPS13	-1.25	0.01	-1.26	3.71E-07
KIZ-AS1	-1.19	0.05	-1.24	1.51E-05
RPL26	-1.15	0.07	-1.23	5.36E-09
RPS29	-1.16	0.07	-1.23	7.12E-05
RPS4X	-1.03	0.10	-1.13	2.66E-07
GAPDH	-0.94	0.19	-1.13	1.01E-06
RPL10A	-1.05	0.07	-1.12	8.31E-07
AMBRA1	-1.05	0.06	-1.11	7.94E-07
RPL27	-0.91	0.18	-1.10	8.07E-04
RPS9	-1.07	0.01	-1.09	6.12E-08
UBA52	-1.02	0.06	-1.07	8.31E-06
RPL27A	-1.08	-0.04	-1.04	9.50E-07
EEF1D	-1.00	0.03	-1.03	3.16E-08
RPS21	-0.98	0.03	-1.00	9.77E-04

Table S8. Gene set enrichment analysis performed on ribosomal occupancy data from CCRF-CEM cells treated with Torin1 for 1 hour. The adjusted p-value (padj) for the gene set enrichment analysis (GSEA) and the average fold change of all genes included in the analysis (Log2FC)) are shown.

Ribosomal Occupancy gene enrichment	Log2FC	padj
**GO-cellular component**		
**cytosolic ribosome**	-1.17	7.80E-23
**ribosomal subunit**	-1.17	2.00E-19
**ribosome**	-1.17	8.20E-19
**cytosolic small ribosomal subunit**	-1.18	4.30E-15
**small ribosomal subunit**	-1.18	5.10E-13
**GO- molecular function**		
**structural constituent of ribosome**	-1.17	6.70E-20
**structural molecule activity**	-1.17	2.20E-14
**translation regulator activity**	-1.15	6.90E-03
**rRNA binding**	-1.21	4.10E-05
**translation elongation factor activity**	-1.19	4.40E-03
**GO - biological processes**		
**cytoplasmic translation**	-1.17	4.70E-19
**regulation of cellular amide metabolic processes**	-1.18	2.60E-02
**regulation of translation**	-1.18	2.60E-02
**regulation of cellular macromolecule biosynthetic process**	-1.18	2.60E-02
**rRNA processing**	-1.11	2.60E-02
**KEGG**		
**coronavirus disease**	-1.17	7.50E-17
**ribosome**	-1.17	9.80E-18

Table S9. Gene set enrichment analysis performed on differential expression data from CCRF-CEM cells treated with AVX420 (0.5 µM) for 16 hours. The adjusted p-value (padj) for the enrichment analysis and the average fold change of all genes included in the analysis (Log2FC) are shown.

RNA-seq gene enrichment	Log2FC	padj
GO-Biological Process		
response to ER stress	1.64	2.80E-03
intrinsic apoptotic signaling in response to endoplasmic reticulum stress	1.82	5.70E-03
response to unfolded protein	1.63	1.10E-02
endoplasmic reticulum unfolded protein response	1.25	1.10E-02
negative regulation of protein processing	2.51	1.80E-02
GO- molecular function		
organic acid binding	1.64	4.20E-02
amino acid binding	1.81	4.20E-02
cysteine-type endopeptidase inhibitor activity	2.31	4.20E-02

Supplemental Methods

Synthesis of Inhibitors

Chromatographic purification of products was accomplished using Merck Silica Gel 60 (70-230 or 230-400 mesh). Thin-layer chromatography (TLC) was performed on Silica Gel 60 F254 aluminum plates. Visualization of the developed TLC was carried out by UV light and/or phosphomolybdic acid in EtOH. Melting points were determined using a Büchi 530 apparatus and were uncorrected. ^{1}H, ^{13}C and ^{19}F NMR spectra were recorded on a Varian Mercury (200 MHz, 50 MHz and 188 MHz, respectively) and on a Bruker AVANCE III (400 MHz, 100 MHz and 377 MHz, respectively) in $CDCl_3$. ^{1}H and ^{13}C NMR spectra of inhibitors **17d**, **h-j** were recorded on a Bruker AVANCE III (600 MHz and 150 MHz, respectively) in $CDCl_3$. Chemical shifts are given in ppm, and coupling constants (*J*) in Hz. Peak multiplicities are described as follows: s, singlet, d, doublet, t, triplet and m, multiplet. Electron spray ionization (ESI) mass spectra were recorded on a Finnigan, Surveyor MSQ Plus spectrometer. Dichloromethane, diethylether and toluene were dried by standard procedures and stored over molecular sieves. All other solvents and chemicals were reagent grade and used without further purification. The purity of all compounds subjected to biological tests was determined by analytical HPLC, and was found to be ≥95%. HRMS spectra were recorded on a Bruker Maxis Impact QTOF Spectrometer.

Compounds **10k**,[2] **11a**,[3] **12a**,[3] **12d**,[3] and **13a**[3] were prepared according to the literature.

Ethyl 2-(4-(heptyloxy)phenoxy)acetate (11b)

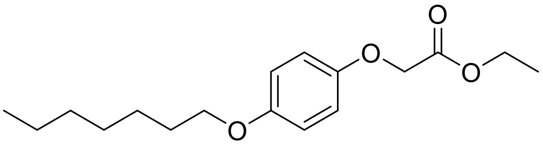

Yield 96% (282 mg); White oil; **^{1}H-NMR** (400 MHz, $CDCl_3$): δ 6.85 (d, J = 9.2 Hz, 2H, ArH), 6.81 (d, J = 9.2 Hz, 2H, ArH), 4.56 (s, 2H, OCH_2), 4.26 (q, J = 7.1 Hz, 2H, OCH_2), 3.89 (t, J = 6.6 Hz, 2H, OCH_2), 1.82-1.62 (m, 2H, CH_2), 1.52-1.11 (m, 11H, 4 x CH_2 and CH_3), 0.88 (t, J = 6.6 Hz, 3H, CH_3); **^{13}C-NMR** (100 MHz, $CDCl_3$): δ 169.0, 153.9, 151.7, 115.6, 115.1, 68.3, 66.1, 61.0, 31.6, 29.2, 28.9, 25.9, 22.5, 14.0, 13.9; **HRMS (ESI^{+})** *m/z* calcd for $C_{17}H_{27}O_4^{+}$: 295.1904; $[M + H]^{+}$ found: 295.1904.

Ethyl 2-(4-phenoxyphenoxy)acetate (11c)

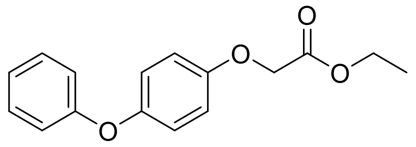

Commercially Available.

Ethyl 2-(4-(4-fluorophenoxy)phenoxy)acetate (11d)

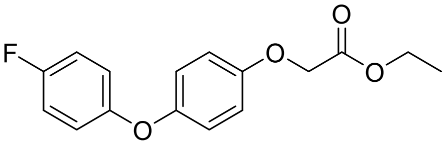

Yield 94% (273 mg); White oil; **^{1}H-NMR** (400 MHz, $CDCl_3$): δ 6.97-6.91 (m, 2H, ArH), 6.90-6.82 (m, 6H, ArH), 4.55 (s, 2H, OCH_2), 4.21 (q, J = 7.1 Hz, 2H, OCH_2), 1.25 (t, J = 7.1 Hz, 3H, CH_3); **^{13}C-NMR** (100 MHz, $CDCl_3$): δ 168.6, 158.2 (d, J = 240.7 Hz), 153.8, 153.6, 153.6, 151.3, 119.7, 119.2 (d, J = 8.1 Hz), 115.9 (d, J = 23.3 Hz), 115.7, 65.6, 61.0, 13.8; **^{19}F-NMR** (377 MHz, $CDCl_3$): δ -120.80; **HRMS (ESI$^+$)** *m/z* calcd for $C_{16}H_{16}FO_4^+$: 291.1027; $[M + H]^+$ found: 291.1020.

Ethyl 2-(4-(hexylthio)phenoxy)acetate (11e)

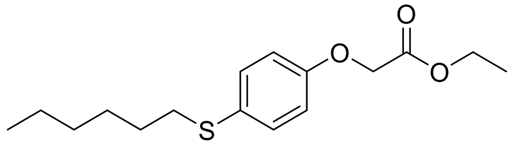

Yield 92% (272 mg); White oil; **^{1}H-NMR** (200 MHz, $CDCl_3$): δ 7.29 (d, J = 8.9 Hz, 2H, ArH), 6.81 (d, J = 8.9 Hz, 2H, ArH), 4.57 (s, 2H, OCH_2), 4.24 (q, J = 7.1 Hz, 2H, OCH_2), 2.79 (t, J = 7.0 Hz, 2H, $PhSCH_2$), 1.64-1.46 (m, 2H, CH_2), 1.45-1.14 (m, 9H, 3 x CH_2 and CH_3), 0.84 (t, J = 7.1 Hz, 3H, CH_3); **^{13}C-NMR** (50 MHz, $CDCl_3$): δ 168.7, 156.5, 132.2, 128.2, 115.0, 65.3, 61.3, 35.2, 31.3, 29.1, 28.3, 22.4, 14.0, 14.0; **HRMS (ESI$^+$)** *m/z* calcd for $C_{16}H_{24}NaO_3S^+$: 319.1338; $[M + Na]^+$ found: 319.1340.

Ethyl 2-(4-(hexyloxy)phenylthio)acetate (11f)

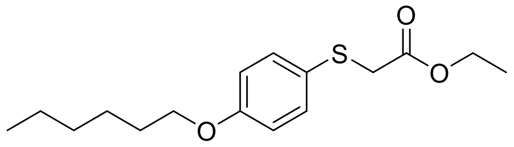

Yield 95% (281 mg); White oil; **^{1}H-NMR** (400 MHz, $CDCl_3$): δ 7.39 (d, J = 9.0 Hz, 2H, ArH), 6.81 (d, J = 9.0 Hz, 2H, ArH), 4.11 (q, J = 7.2 Hz, 2H, OCH_2), 3.90 (t, J = 6.4 Hz, 2H, OCH_2), 3.48 (s, 2H, $PhSCH_2$), 1.89-1.63 (m, 2H, CH_2), 1.52-1.20 (m, 9H, 3 x CH_2 and CH_3), 0.88 (t, J = 7.2 Hz, 3H, CH_3); **^{13}C-NMR** (100 MHz, $CDCl_3$): δ

169.9, 159.3, 134.2, 124.6, 115.2, 68.1, 61.3, 38.7, 31.5, 29.1, 25.7, 22.6, 14.1, 14.0; **HRMS (ESI$^+$)** *m/z* calcd for $C_{16}H_{24}NaO_3S^+$: 319.1338; $[M + Na]^+$ found: 319.1339.

Ethyl 2-(4-octylphenoxy)propanoate (11g)

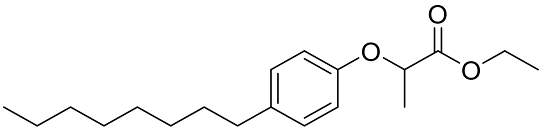

Yield 98% (303 mg); White oil; **^{1}H-NMR** (400 MHz, $CDCl_3$): δ 7.07 (d, J = 8.4 Hz, 2H, ArH), 6.80 (d, J = 8.4 Hz, 2H,ArH), 4.71 (q, J = 6.8 Hz, 1H, OCH), 4.22 (q, J = 7.1 Hz, 2H, OCH_2), 2.54 (t, J = 8.0 Hz, 2H, OCH_2), 1.70-1.43 (m, 5H, CH_2 and CH_3), 1.41-1.09 (m, 13H, 5 x CH_2 and CH_3), 0.88 (t, J = 7.1 Hz, 3H, CH_3); **^{13}C-NMR** (100 MHz, $CDCl_3$): δ 172.4, 155.6, 136.0, 129.2, 114.9, 72.8, 61.1, 35.0, 31.8, 31.6, 29.4, 29.2, 22.6, 18.5, 14.1, 14.0; **HRMS (ESI$^+$)** *m/z* calcd for $C_{19}H_{31}O_3^+$: 307.2268; $[M + H]^+$ found: 307.2268.

Ethyl 2-(4-(heptyloxy)phenoxy)propanoate (11h)

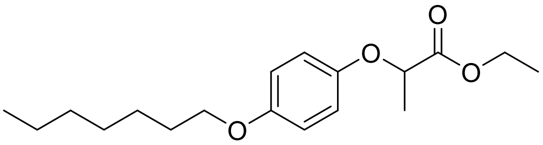

Yield 97% (299 mg); White oil; **^{1}H-NMR** (200 MHz, $CDCl_3$): δ 6.86-6.69 (m, 4H, ArH), 4.63 (q, J = 6.8 Hz, 1H, OCH), 4.18 (q, J = 7.1 Hz, 2H, OCH_2), 3.86 (t, J = 6.5 Hz, 2H, OCH_2), 1.84-1.64 (m, 2H, CH_2), 1.57 (d, J = 6.6 Hz, 3H, CH_3), 1.46-1.13 (m, 11H, 4 x CH_2 and CH_3), 0.87 (t, J = 6.3 Hz, 3H, CH_3); **^{13}C-NMR** (50 MHz, $CDCl_3$): δ 172.3, 153.8, 151.4, 116.2, 115.1, 73.4, 68.2, 61.0, 31.7, 29.2, 28.9, 25.9, 22.5, 18.4, 13.9; **HRMS (ESI$^+$)** *m/z* calcd for $C_{18}H_{29}O_4^+$: 309.2060; $[M + H]^+$ found: 309.2064.

Ethyl 2-(4-phenoxyphenoxy)propanoate[4] (11i)

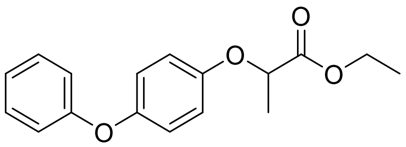

Yield 96% (275 mg); White oil; **^{1}H-NMR** (400 MHz, $CDCl_3$): δ 7.29 (t, J = 7.4 Hz, 2H, ArH), 7.05 (t, J = 7.4 Hz, 1H, ArH), 7.00-6.93 (m, 4H, ArH), 6.91-6.86 (m, 2H, ArH), 4.71 (q, J = 6.8 Hz, 1H, OCH), 4.24 (q, J = 7.1 Hz, 2H, OCH_2), 1.63 (d, J = 6.8 Hz, 3H, CH_3), 1.27 (t, J = 7.1 Hz, 3H, CH_3); **^{13}C-NMR** (100 MHz, $CDCl_3$): δ 172.0, 158.0, 153.6, 150.8, 129.5, 122.5, 120.4, 117.7, 116.3, 73.1, 61.0, 18.4, 14.0; **HRMS (ESI$^+$)** *m/z* calcd for $C_{17}H_{19}O_4^+$: 287.1278; $[M + H]^+$ found: 287.1280.

Ethyl 2-(4-(hexylthio)phenoxy)propanoate (11j)

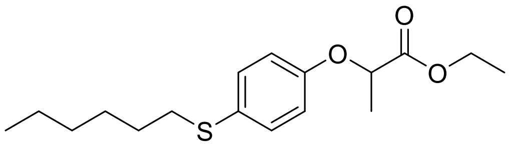

Yield 95% (295 mg); White oil; **^{1}H-NMR** (200 MHz, $CDCl_3$): δ 7.22 (d, J = 9.0 Hz, 2H, ArH), 6.74 (d, J = 9.0 Hz, 2H, ArH), 4.64 (q, J = 6.8 Hz, 1H, OCH), 4.14 (q, J = 7.1 Hz, 2H, OCH_2), 2.75 (t, J = 7.0 Hz, 2H, $PhSCH_2$), 1.59-1.42 (m, 5H, CH_2 and CH_3), 1.39-1.10 (m, 9H, 3 x CH_2 and CH_3), 0.80 (t, J = 7.1 Hz, 3H, CH_3); **^{13}C-NMR** (50 MHz, $CDCl_3$): δ 171.7, 156.3, 132.1, 127.9, 115.4, 72.4, 61.0, 35.1, 31.1, 29.0, 28.1, 22.3, 18.3, 13.9, 13.8; **HRMS (ESI$^+$)** *m/z* calcd for $C_{17}H_{27}O_3S^+$: 311.1675; $[M + H]^+$ found: 311.1679.

2-(4-(Heptyloxy)phenoxy)ethanol (12b)

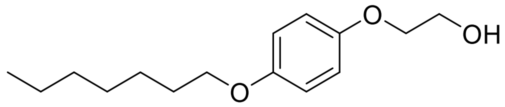

Yield 88% (222 mg); White solid m.p.: 77-78 ºC; **^{1}H-NMR** (400 MHz, $CDCl_3$): δ 6.93-6.76 (m, 4H, ArH), 4.06-3.99 (m, 2H, OCH_2), 3.96-3.86 (m, 4H, 2 x OCH_2), 2.25 (br s, 1H, OH), 1.81-1.70 (m, 2H, CH_2), 1.51-1.23 (m, 8H, 4 x CH_2), 0.89 (t, J = 6.1 Hz, 3H, CH_3); **^{13}C-NMR** (100 MHz, $CDCl_3$): δ 153.6, 152.6, 115.5, 115.4, 69.9, 68.6, 61.5, 31.7, 29.3, 29.0, 26.0, 22.6, 14.0; **HRMS (ESI$^+$)** *m/z* calcd for $C_{15}H_{24}NaO_3^+$: 275.1618; $[M + Na]^+$ found: 275.1618.

2-(4-Phenoxyphenoxy)ethanol (12c)

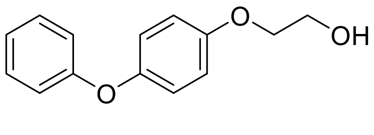

Commercially available.

2-(4-(Hexylthio)phenoxy)ethanol (12e)

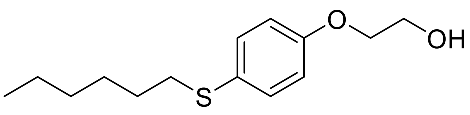

Yield 80% (203 mg); White solid m.p.: 48-49 ºC; **^{1}H-NMR** (200 MHz, $CDCl_3$): δ 7.30 (d, J = 7.8 Hz, 2H, ArH), 6.82 (d, J = 7.8 Hz, 2H, ArH), 4.16-3.80 (m, 4H, 2 x OCH_2), 2.79 (t, J = 7.2 Hz, 2H, SCH_2), 2.58 (br s, 1H, OH), 1.67-1.47 (m, 2H, CH_2), 1.46-1.16 (m, 6H, 3 x CH_2), 0.86 (t, J = 6.3 Hz, 3H, CH_3); **^{13}C-NMR** (50 MHz, $CDCl_3$): δ 157.5, 132.6, 127.4, 115.0, 69.2, 61.2, 35.5, 31.3, 29.1, 28.3, 22.4, 13.9; **HRMS (ESI$^+$)** *m/z* calcd for $C_{14}H_{22}NaO_2S^+$: 277.1233; $[M + Na]^+$ found: 277.1233.

2-(4-(Hexyloxy)phenylthio)ethanol (12f)

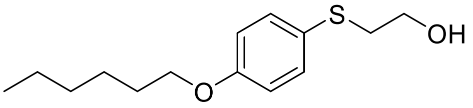

Yield 81% (206 mg); White solid low m.p.; **^{1}H-NMR** (200 MHz, $CDCl_3$): δ 7.37 (d, J = 8.8 Hz, 2H, ArH), 6.84 (d, J = 8.8 Hz, 2H, ArH), 3.93 (t, J = 6.6 Hz, 2H, OCH_2), 3.73-3.57 (m, 2H, OCH_2), 2.98 (t, J = 6.0 Hz, 2H, $PhSCH_2$), 2.39 (br s, 1H, OH), 1.86-1.66 (m, 2H, CH_2), 1.55-1.15 (m, 6H, 3 x CH_2), 0.91 (t, J = 6.4 Hz, 3H, CH_3); **^{13}C-NMR** (50 MHz, $CDCl_3$): δ 158.8, 133.9, 124.2, 115.1, 68.0, 59.9, 39.1, 31.5, 29.0, 25.6, 22.5, 13.9; **HRMS (ESI$^+$)** *m/z* calcd for $C_{14}H_{22}NaO_2S^+$: 277.1233; $[M + Na]^+$found: 277.1233.

2-(4-Octylphenoxy)propan-1-ol (12g)

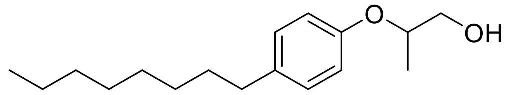

Yield 87% (230 mg); White oil; **^{1}H-NMR** (400 MHz, $CDCl_3$): δ 7.10 (d, J = 8.2 Hz, 2H, ArH), 6.86 (d, J = 8.2 Hz, 2H, ArH), 4.52-4.39 (m, 1H, OCH), 3.78-3.65 (m, 2H, OCH_2), 2.56 (t, J = 7.7 Hz, 2H, $PhCH_2$), 2.47 (br s, 1H, OH), 1.66-1.54 (m, 2H, CH_2), 1.40-1.22 (m, 13H, 5 x CH_2 and CH_3), 0.91 (t, J = 6.7 Hz, 3H, CH_3); **^{13}C-NMR** (100 MHz, $CDCl_3$): δ 155.6, 135.6, 129.3, 116.0, 74.9, 66.1, 35.0, 31.8, 31.6, 29.4, 29.2, 29.2, 22.6, 15.8, 14.0; **HRMS (ESI$^+$)** *m/z* calcd for $C_{17}H_{28}NaO_2^+$: 287.1982; $[M + Na]^+$ found: 287.1982.

2-(4-(Heptyloxy)phenoxy)propan-1-ol (12h)

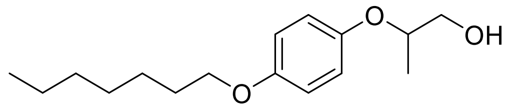

Yield 92% (245 mg); White solid m.p.: 81-83 °C; **^{1}H-NMR** (200 MHz, $CDCl_3$): δ 6.85 (d, J = 9.4 Hz, 2H, ArH), 6.79 (d, J = 9.4 Hz, 2H, ArH), 4.40-4.21 (m, 1H, OCH), 3.88 (t, J = 6.5 Hz, 2H, 2 x OCH_2), 3.71-3.59 (m, 2H, OCH_2), 2.74 (s, 1H, OH), 1.82-1.55 (m, 2H, CH_2), 1.49-1.24 (m, 8H, 4 x CH_2), 1.20 (d, J = 6.2 Hz, 3H, CH_3), 0.89 (t, J = 6.4 Hz, 3H, CH_3); **^{13}C-NMR** (50 MHz, $CDCl_3$): δ 153.7, 151.3, 117.6, 115.2, 75.9, 68.4, 66.0, 31.7, 29.2, 29.0, 25.9, 22.5, 15.8, 14.0; **HRMS (ESI$^+$)** *m/z* calcd for $C_{16}H_{26}NaO_3^+$: 289.1774; $[M + Na]^+$ found: 289.1777.

2-(4-Phenoxyphenoxy)propan-1-ol (12i)

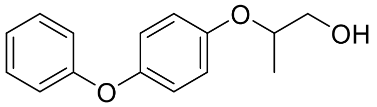

Yield 83% (203 mg); White solid m.p.:79-81 ºC; **^{1}H-NMR** (400 MHz, $CDCl_3$): δ 7.31 (t, J = 7.5 Hz, 2H, ArH), 7.05 (t, J = 7.5 Hz, 1H, ArH), 7.01-6.89 (m, 6H, ArH), 4.49-4.38 (m, 1H, OCH), 3.80-3.66 (m, 2H, OCH_2), 2.06 (br s, 1H, OH), 1.28 (d, J = 6.1 Hz, 3H, CH_3); **^{13}C-NMR** (100 MHz, $CDCl_3$): δ 158.2, 153.8, 150.8, 129.6, 122.6, 120.7, 117.8, 117.5, 75.6, 66.3, 15.8; **HRMS (ESI$^+$)** *m/z* calcd for $C_{15}H_{16}NaO_3^+$: 267.0992; $[M + Na]^+$ found: 267.0993.

2-(4-(Hexylthio)phenoxy)propan-1-ol (12j)

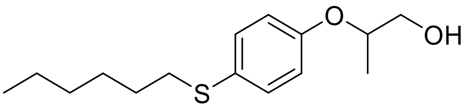

Yield 85% (228 mg); White oil; **^{1}H-NMR** (200 MHz, $CDCl_3$): δ 7.27 (d, J = 8.8 Hz, 2H, ArH), 6.82 (d, J = 8.8 Hz, 2H, ArH), 4.51-4.32 (m, 1H, OCH), 3.72-3.60 (m, 2H, OCH_2), 2.79 (t, J = 7.0 Hz, 2H, SCH_2), 2.67 (s, 1H, OH), 1.65-1.47 (m, 2H, CH_2), 1.45-1.14 (m, 9H, 3 x CH_2 and CH_3), 0.85 (t, J = 6.5 Hz, 3H, CH_3); **^{13}C-NMR** (50 MHz, $CDCl_3$): δ 156.5, 132.5, 127.4, 116.4, 74.8, 65.9, 35.4, 31.2, 29.1, 28.3, 22.4, 15.6, 13.9; **HRMS (ESI$^+$)** *m/z* calcd for $C_{15}H_{24}NaO_2S^+$: 291.1389; $[M + Na]^+$ found: 291.1391.

2-(*tert*-Butyldimethylsilyloxy)-3-(4-(heptyloxy)phenoxy)propanenitrile (13b)

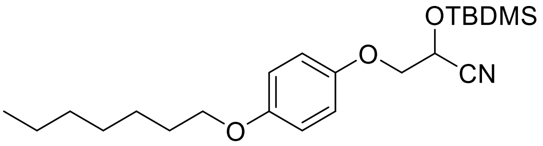

Yield 73% (286 mg); White oil; **^{1}H-NMR** (200 MHz, $CDCl_3$): δ 6.90-6.73 (m, 4H, ArH), 4.76 (t, J = 6.2 Hz, 1H, CHCN), 4.20-4.02 (m, 2H, OCH_2), 3.90 (t, J = 6.5 Hz, 2H, OCH_2), 1.84-1.66 (m, 2H, CH_2), 1.49-1.23 (m, 8H, 4 x CH_2), 0.97-0.80 (m, 12H, $C(CH_3)_3$ and CH_3), 0.19 (d, J = 8.7 Hz, 6H, 2 x $SiCH_3$); **^{13}C-NMR** (50 MHz, $CDCl_3$): δ 154.1, 151.7, 118.2, 115.8, 115.4, 70.4, 68.5, 61.5, 31.8, 29.3, 29.1, 26.0, 25.7, 25.4, 22.6, 18.1, 14.1, -5.3; **HRMS (ESI$^+$)** *m/z* calcd for $C_{22}H_{37}NNaO_3Si^+$: 414.2435; $[M + Na]^+$ found: 414.2438.

2-(*tert*-Butyldimethylsilyloxy)-3-(4-phenoxyphenoxy)propanenitrile (13c)

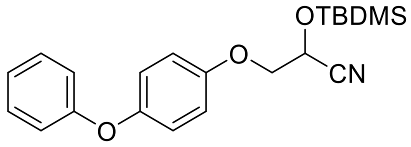

Yield 28% (103 mg); White oil; **^{1}H-NMR** (200 MHz, $CDCl_3$): *δ* 7.38-7.24 (m, 2H, ArH), 7.12-6.85 (m, 7H, ArH), 4.84-4.75 (m, 1H, CHCN), 4.21-4.10 (m, 2H, OCH_2), 0.94 (s, 9H, $C(CH_3)_3$), 0.24 (s, 3H, $SiCH_3$), 0.19 (s, 3H, $SiCH_3$); **^{13}C-NMR** (50 MHz, $CDCl_3$): *δ* 158.1, 153.9, 151.1, 129.6, 122.6, 120.7, 118.0, 117.7, 115.8, 70.1, 61.5, 25.4, 18.1, -5.2, -5.3; **HRMS (ESI$^+$)** *m/z* calcd for $C_{21}H_{27}NNaO_3Si^+$: 392.1652; [M + Na]$^+$ found: 392.1652.

2-((*tert*-Butyldimethylsilyl)oxy)-3-(4-(4-fluorophenoxy)phenoxy)propanenitrile (13d)

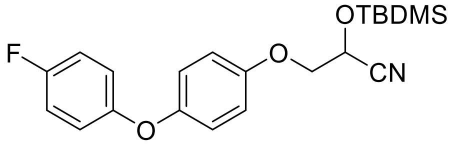

Yield 82% (318 mg); Pale yellow syrup; **^{1}H-NMR** (400 MHz, $CDCl_3$): *δ* 7.04-6.85 (m, 8H, ArH), 4.80 (t, *J* = 6.0 Hz, 1H, CHCN), 4.21-4.10 (m, 2H, OCH_2), 0.96 (s, 9H, $C(CH_3)_3$), 0.25 (s, 3H, $SiCH_3$), 0.21 (s, 3H, $SiCH_3$); **^{13}C-NMR** (100 MHz, $CDCl_3$): *δ* 158.3 (d, *J* = 240.1 Hz), 153.8, 153.7, 151.5, 120.0, 119.5 (d, *J* = 7.8 Hz), 117.8, 116.1, 115.8 (d, *J* = 22.6 Hz), 70.1, 61.4, 25.3, 17.9, -4.8, -5.4; **HRMS (ESI$^+$)** *m/z* calcd for $C_{21}H_{26}FNNaO_3Si^+$: 410.1558; [M + Na]$^+$ found: 410.1562.

2-(*tert*-Butyldimethylsilyloxy)-3-(4-(hexylthio)phenoxy)propanenitrile (13e)

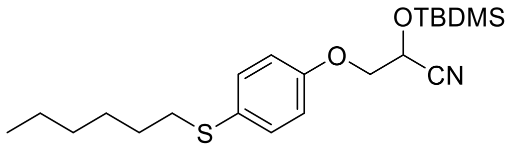

Yield 36% (142 mg); White oil; **^{1}H-NMR** (200 MHz, $CDCl_3$): *δ* 7.28 (d, *J* = 7.0 Hz, 2H, ArH), 6.79 (d, *J* = 7.0 Hz, 2H, ArH), 4.90-4.61 (m, 1H, CHCN), 4.28-3.87 (m, 2H, OCH_2), 3.02-2.57 (m, 2H, SCH_2), 1.67-1.06 (m, 8H, 4 x CH_2), 1.03-0.63 (m, 12H, $C(CH_3)_3$ and CH_3), 0.29-(-0.04) (m, 6H, $Si(CH_3)_2$); **^{13}C-NMR** (50 MHz, $CDCl_3$): *δ* 156.6, 132.4, 128.4, 117.9, 115.1, 69.5, 61.3, 35.3, 31.3, 29.6, 29.1, 28.3, 25.4, 22.5, 14.0, -5.3; **HRMS (ESI$^+$)** *m/z* calcd for $C_{21}H_{35}NNaO_2SSi^+$: 416.2050; [M + Na]$^+$ found: 416.2053.

2-(*tert*-Butyldimethylsilyloxy)-3-(4-(hexyloxy)phenylthio)propanenitrile (13f)

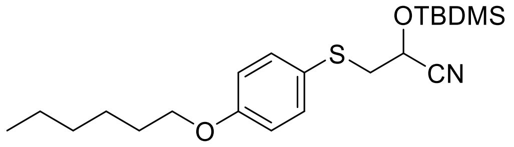

Yield 30% (118 mg); White oil; **^{1}H-NMR** (200 MHz, $CDCl_3$): *δ* 7.41 (d, *J* = 8.7 Hz, 2H, ArH), 6.87 (d, *J* = 8.7 Hz, 2H, ArH), 4.44-4.35 (m, 1H, CHCN), 4.02-3.87 (m, 2H, OCH_2), 3.20-3.03 (t, m, 2H, $PhSCH_2$), 1.86-170 (m, 2H, CH_2), 1.55-1.18 (m, 6H, 3 x CH_2), 0.96-0.79 (m, 12H, $C(CH_3)_3$ and CH_3), 0.18-(-0.05) (m, 6H, $Si(CH_3)_2$); **^{13}C-NMR** (50 MHz, $CDCl_3$): *δ* 159.4, 134.3, 124.0, 116.3, 115.4, 68.2, 62.0, 41.9, 31.5, 29.1, 25.6, 25.4, 22.6, 18.0, 14.0, -5.3, -5.4; **HRMS (ESI$^+$)** *m/z* calcd for $C_{21}H_{35}NNaO_2SSi^{+\ +}$: 416.2050; $[M + Na]^+$ found: 416.2047.

2-(*tert*-Butyldimethylsilyloxy)-3-(4-octylphenoxy)butanenitrile (mixture of diastereomers) (13g)

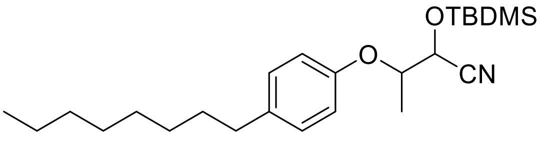

Yield 51% (206 mg); Pale yellow oil; **^{1}H-NMR** (200 MHz, $CDCl_3$): *δ* 7.19-7.01 (m, 2H, ArH), 6.93-6.70 (m, 2H, ArH), 4.81-4.35 (m, 2H, CHCN and OCH), 2.66-2.44 (m, 2H, $PhCH_2$), 1.72-1.15 (m, 15H, 6 x CH_2 and CH_3), 1.05-0.76 (m, 12H, $C(CH_3)_3$ and CH_3), 0.25-0.15 (m, 6H, $Si(CH_3)_2$); **^{13}C-NMR** (50 MHz, $CDCl_3$): *δ* 155.6, 154.9, 136.3, 135.6, 129.4, 129.2, 118.4, 114.6, 75.5, 74.6, 65.4, 65.1, 35.0, 31.9, 31.6, 29.5, 29.3, 29.1, 25.6, 25.4, 25.3, 22.6, 18.1, 18.0, 15.4, 15.1, 14.1, -5.3, -5.4; **HRMS (ESI$^+$)** *m/z* calcd for $C_{24}H_{41}NNaO_2Si^+$: 426.2799; $[M + Na]^+$ found: 426.2802.

2-((tert-butyldimethylsilyl)oxy)-3-(4-(heptyloxy)phenoxy)butanenitrile (mixture of diastereomers) (13h)

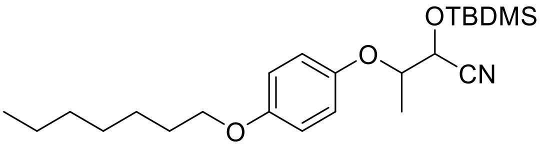

Yield 78% (316 mg); White oil; **^{1}H-NMR** (200 MHz, $CDCl_3$): *δ* 6.94-6.78 (m, 4H, ArH), 4.65-4.48 (m, 1H, CHCN), 4.47-4.24 (m, 1H, OCH), 3.92 (t, *J* = 6.5 Hz, 2H, OCH_2), 1.89-1.64 (m, 2H, CH_2), 1.54-1.25 (m, 11H, 4 x CH_2 and CH_3), 1.01-0.84 (m, 12H, $C(CH_3)_3$ and CH_3), 0.29-0.09 (m, 6H, $Si(CH_3)_2$); **^{13}C-NMR** (50 MHz, $CDCl_3$): *δ* 154.2, 150.8, 117.7, 115.3, 76.7, 75.7, 68.4, 65.3, 65.1, 31.7, 29.3, 29.0, 25.9, 25.4, 22.5, 15.5, 15.1, 14.0, -5.3, -5.4; **HRMS (ESI$^+$)** *m/z* calcd for $C_{23}H_{39}NNaO_3Si^+$: 428.2591; $[M + Na]^+$ found: 428.2594.

2-(*tert*-Butyldimethylsilyloxy)-3-(4-phenoxyphenoxy)butanenitrile (mixture of diastereomers) (13i)

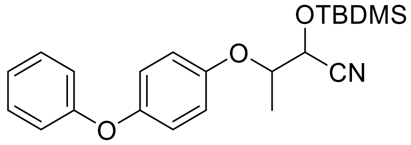

Yield 19% (73 mg); Pale yellow oil; **^{1}H-NMR** (200 MHz, $CDCl_3$): δ 7.46-7.22 (m, 2H, ArH), 7.20-6.85 (m, 7H, ArH), 4.66-4.32 (m, 2H, CHCN and OCH), 1.55-1.36 (m, 3H, CH_3), 0.96-0.89 (m, 9H, $C(CH_3)_3$), 0.29-0.11 (m, 6H, $Si(CH_3)_2$); **^{13}C-NMR** (50 MHz, $CDCl_3$): δ 157.3, 153.0, 151.1, 132.6, 131.5, 129.6, 127.8, 122.6, 120.6, 117.8, 117.7, 117.6, 117.3, 76.1, 75.2, 65.4, 65.2, 25.7, 25.4, 18.0, 17.8, 15.6, 15.1, -5.3, -5.4; **HRMS (ESI$^+$)** *m/z* calcd for $C_{22}H_{29}NNaO_3Si^+$: 406.1809; $[M + Na]^+$ found: 406.1812.

2-((*tert*-Butyldimethylsilyl)oxy)-3-(4-(hexylthio)phenoxy)butanenitrile (mixture of diastereomers) (13j)

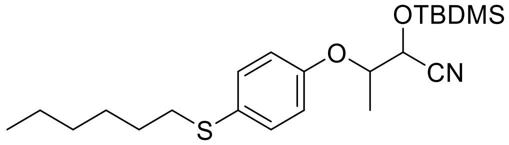

Yield 34% (138 mg); White oil; **^{1}H-NMR** (200 MHz, $CDCl_3$): δ 7.31 (d, J = 8.8 Hz, 2H, ArH), 6.85 (d, J = 8.8 Hz, 2H, ArH), 5.32-5.28 (m, 1H, CHCN), 4.63-4.38 (m, 1H, OCH), 2.82 (t, J = 7.2 Hz, 2H, SCH_2), 1.71-1.19 (m, 11H, 4 x CH_2 and CH_3), 1.00-0.82 (m, 12H, $C(CH_3)_3$ and CH_3), 0.28-0.09 (m, 6H, $Si(CH_3)_2$); **^{13}C-NMR** (50 MHz, $CDCl_3$): δ 156.9, 132.4, 132.3, 128.6, 116.7, 116.5, 75.5, 74.7, 65.5, 65.2, 35.4, 33.2, 31.3, 29.2, 28.4, 25.4, 22.5, 18.0, 15.4, 15.1, 14.0, -5.3, -5.3; **HRMS (ESI$^+$)** *m/z* calcd for $C_{22}H_{37}NNaO_2SSi^+$: 430.2206; $[M + Na]^+$ found: 430.2209.

Ethyl 2-(1-(*tert*-butyldimethylsilyloxy)-2-(4-octylphenoxy)ethyl)-4,5-dihydrothiazole-4-carboxylate (mixture of diastereomers) (14a)

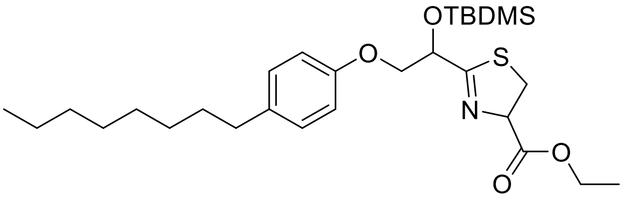

Yield 92% (480 mg); White oil; **^{1}H-NMR** (200 MHz, $CDCl_3$): δ 7.06 (d, J = 8.4 Hz, 2H, ArH), 6.81 (d, J = 8.4 Hz, 2H, ArH), 5.14 (t, J = 9.3 Hz, 1H, OCH), 5.05-4.91 (m, 1H, NCH), 4.37-4.16 (m, 3H, 3 x OC*H*H), 4.15-3.99 (m, 1H, OC*H*H), 3.61-3.33 (m,

2H, SCH_2), 2.53 (t, J = 7.8 Hz, 2H, $PhCH_2$), 1.67-1.47 (m, 2H, CH_2), 1.38-1.19 (m, 13H, 5 x CH_2 and CH_3), 0.99-0.81 (m, 12H, $C(CH_3)_3$ and CH_3), 0.26-0.05 (m, 6H, $Si(CH_3)_2$); **^{13}C-NMR** (50 MHz, $CDCl_3$): δ 178.4, 178.0, 170.6, 156.4, 135.2, 129.1, 114.3, 78.4, 72.5, 72.4, 71.6, 71.5, 61.7, 35.0, 33.8, 33.8, 31.8, 31.7, 29.4, 29.2, 25.7, 25.7, 22.6, 18.2, 14.1, 14.1, -4.7, -5.2, -5.2; **HRMS (ESI$^+$)** *m/z* calcd for $C_{28}H_{48}NO_4SSi^+$: 522.3068; $[M + H]^+$ found: 522.3060.

Methyl 2-(1-(*tert*-butyldimethylsilyloxy)-2-(4-(heptyloxy)phenoxy)ethyl)-4,5-dihydrothiazole-4-carboxylate (mixture of diastereomers) (14b)

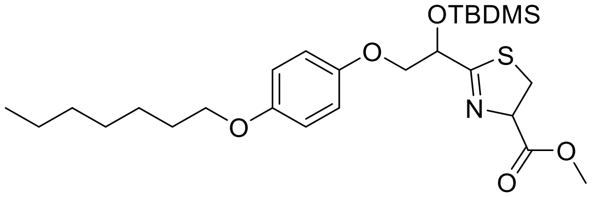

Yield 63% (321 mg); Pale yellow oil; **^{1}H-NMR** (200 MHz, $CDCl_3$): δ 6.89-6.67 (m, 4H, ArH), 5.23-5.07 (m, 1H, OCH), 5.02-4.89 (m, 1H, NCH), 4.28-3.94 (m, 2H, OCH_2), 3.88 (t, J = 6.6 Hz, 2H, OCH_2), 3.80 (s, 3H, OCH_3), 3.57-3.39 (m, 2H, SCH_2), 1.83-1.64 (m, 2H, CH_2), 1.52-1.18 (m, 8H, 4 x CH_2), 1.00-0.72 (m, 12H, $C(CH_3)_3$ and CH_3), 0.24-0.03 (m, 6H, $Si(CH_3)_2$); **^{13}C-NMR** (50 MHz, $CDCl_3$): δ 178.8, 178.3, 171.1, 153.4, 152.5, 115.4, 115.2, 78.3, 78.2, 72.5, 72.5, 72.1, 68.5, 52.8, 33.7, 31.7, 29.3, 29.0, 26.0, 25.6, 22.6, 18.2, 14.1, -4.7, -5.2; **HRMS (ESI$^+$)** *m/z* calcd for $C_{26}H_{43}NNaO_5SSi^+$: 532.2523; $[M + Na]^+$found: 532.2523.

Methyl 2-(1-(*tert*-butyldimethylsilyloxy)-2-(4-phenoxyphenoxy)ethyl)-4,5-dihydrothiazole-4-carboxylate (mixture of diastereomers) (14c)

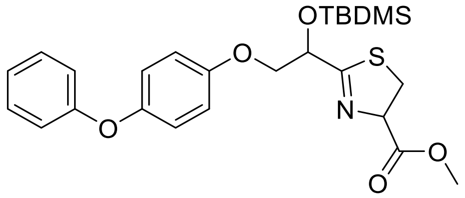

Yield 80% (390 mg); White oil; **^{1}H-NMR** (200 MHz, $CDCl_3$): δ 7.32-7.19 (m, 2H, ArH), 7.07-6.78 (m, 7H, ArH), 5.22-5.05 (m, 1H, OCH), 5.01-4.89 (m, 1H, NCH), 4.30-4.14 (m, 1H, OC*H*H), 4.11-3.92 (m, 1H, OC*H*H), 3.77 (s, 3H, OCH_3), 3.54-3.38 (m, 2H, SCH_2), 0.90 (s, 9H, $C(CH_3)_3$), 0.29-(-0.02) (m, 6H, $Si(CH_3)_2$); **^{13}C-NMR** (50 MHz, $CDCl_3$): δ 178.6, 178.0, 171.0, 158.3, 154.7, 150.2, 129.5, 122.3, 120.7, 117.5, 115.6, 78.3, 78.2, 72.4, 72.0, 53.2, 52.6, 33.7, 25.6, 18.2, -4.7, -5.2; **HRMS (ESI$^+$)** *m/z* calcd for $C_{25}H_{34}NO_5SSi^+$: 488.1921; $[M + H]^+$ found: 488.1922.

Methyl 2-(1-((*tert*-butyldimethylsilyl)oxy)-2-(4-(4-fluorophenoxy)phenoxy)ethyl)-4,5-dihydrothiazole-4-carboxylate (mixture of diastereomers) (14d)

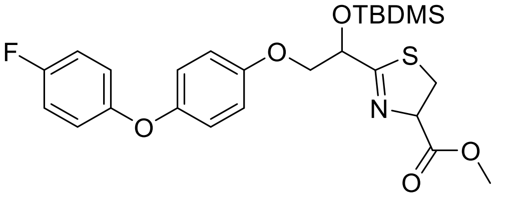

Yield 35% (177 mg); White oil; **^{1}H-NMR** (400 MHz, $CDCl_3$): δ 7.14-6.82 (m, 8H, ArH), 5.24-5.09 (m, 1H, OCH), 5.04-4.90 (m, 1H, NCH), 4.35-4.17 (m, 1H, OC*H*H), 4.14-4.01 (m, 1H, OC*H*H), 3.81 (s, 3H, OCH_3), 3.60-3.41 (m, 2H, $PhSCH_2$), 0.93 (s, 9H, $C(CH_3)_3$), 0.33-(-0.00) (m, 6H, $Si(CH_3)_2$); **^{13}C-NMR** (100 MHz, $CDCl_3$) δ 178.6, 178.0, 171.1, 158.3 (d, J = 240.6 Hz), 154.8, 154.1, 150.9, 146.4, 120.1, 119.2 (d, J = 8.2 Hz), 116.1 (d, J = 23.3 Hz), 115.8, 115.7, 78.4, 78.3, 72.5, 72.5, 72.2, 52.7, 33.8, 25.7, 18.3, -4.7, -5.1; **^{19}F-NMR** (377 MHz, $CDCl_3$): δ -121.31; **HRMS (ESI$^+$)** *m/z* calcd for $C_{25}H_{33}FNO_5SSi^+$: 506.1827; $[M + Na]^+$ found: 506.1825.

Methyl 2-(1-(*tert*-butyldimethylsilyloxy)-2-(4-(hexylthio)phenoxy)ethyl)-4,5-dihydrothiazole-4-carboxylate (mixture of diastereomers) (14e)

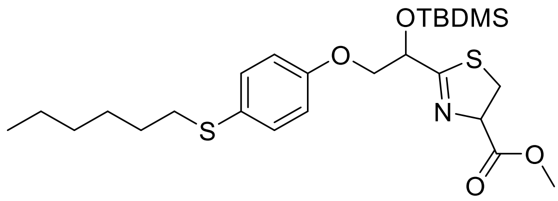

Yield 37% (189 mg); Pale yellow oil; **^{1}H-NMR** (200 MHz, $CDCl_3$): δ 7.25 (d, J = 8.3 Hz, 2H, ArH), 6.78 (d, J = 8.3 Hz, 2H, ArH), 5.26-5.03 (m, 1H, OCH), 5.01-4.84 (m, 1H, NCH), 4.29-4.10 (m, 1H, OC*H*H), 4.09-3.89 (m, 1H, OC*H*H), 3.75 (s, 3H, OCH_3), 3.59-3.29 (m, 2H, SCH_2), 2.75 (t, J = 7.0 Hz, 2H, SCH_2), 1.63-1.13 (m, 8H, 4 x CH_2), 1.01-0.68 (m, 12H, $C(CH_3)_3$ and CH_3), 0.23-(-0.06) (m, 6H, $Si(CH_3)_2$); **^{13}C-NMR** (50 MHz, $CDCl_3$): δ 178.6, 178.1, 171.0, 157.4, 132.6, 127.1, 115.0, 78.3, 78.2, 72.3, 71.5, 52.8, 35.6, 33.7, 31.3, 29.2, 28.3, 25.6, 22.5, 18.2, 14.0, -4.7, -5.2; **HRMS (ESI$^+$)** *m/z* calcd for $C_{25}H_{42}NO_4S_2Si^+$: 512.2319; $[M + H]^+$ found: 512.2321.

Methyl 2-(1-(*tert*-butyldimethylsilyloxy)-2-(4-(hexyloxy)phenylthio)ethyl)-4,5-dihydrothiazole-4-carboxylate (mixture of diastereomers) (14f)

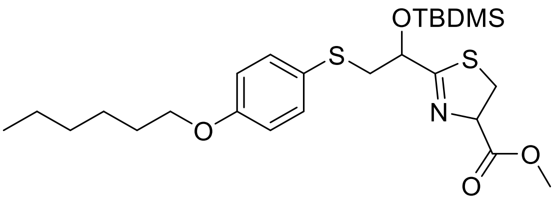

Yield 42% (215 mg); White oil; **^{1}H-NMR** (400 MHz, $CDCl_3$): δ 7.36 (d, J = 8.1 Hz, 2H, ArH), 6.82 (d, J = 8.1 Hz, 2H, ArH), 5.07 (t, J = 9.2 Hz, 1H, OCH), 4.80-4.68 (m, 1H, NCH), 3.97-3.90 (m, 2H, OCH_2), 3.84-3.74 (m, 3H, OCH_3), 3.55-3.29 (m, 2H, SCH_2), 3.23-3.13 (m, 2H, $PhSCH_2$), 1.82-1.73 (m, 2H, CH_2), 1.51-1.31 (m, 6H, 3 x CH_2), 0.92 (s, 12H, $C(CH_3)_3$ and CH_3), 0.17-0.05 (m, 6H, $Si(CH_3)_2$); **^{13}C-NMR** (100 MHz, $CDCl_3$): δ 179.8, 179.1, 171.0, 158.5, 133.1, 126.2, 115.1, 77.9, 77.8, 72.9, 72.5, 68.1, 52.7, 43.0, 42.9, 33.9, 31.6, 29.2, 25.7, 22.6, 18.2, 14.0, -3.6, -4.6; **HRMS (ESI$^+$)** *m/z* calcd for $C_{25}H_{42}NO_4S_2Si^+$: 512.2319; $[M + H]^+$ found: 512.2320.

Methyl 2-(1-(*tert*-butyldimethylsilyloxy)-2-(4-octylphenoxy)propyl)-4,5-dihydrothiazole-4-carboxylate (mixture of diastereomers) (14g)

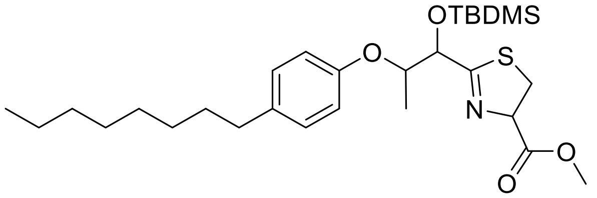

Yield 31% (162 mg); White oil; **^{1}H-NMR** (200 MHz, $CDCl_3$): δ 7.04 (d, J = 8.6 Hz, 2H, ArH), 6.82 (d, J = 8.6 Hz, 2H, ArH), 5.24-5.03 (m, 1H, OCH), 4.87-4.78 (m, 1H, NCH), 4.72-4.57 (m, 1H, CH), 3.85-3.70 (m, 3H, OCH_3), 3.55-3.28 (m, 2H, SCH_2), 2.51 (t, J = 7.8 Hz, 2H, $PhCH_2$), 1.62-1.46 (m, 2H, CH_2), 1.38-1.17 (m, 13H, 5 x CH_2 and CH_3), 1.01-0.78 (m, 12H, $C(CH_3)_3$ and CH_3), 0.21-(-0.06) (m, 6H, $Si(CH_3)_2$); **HRMS (ESI$^+$)** *m/z* calcd for $C_{28}H_{47}NNaO_4SSi^+$: 544.2887; $[M + Na]^+$ found: 544.2887.

Methyl 2-(1-((*tert*-butyldimethylsilyl)oxy)-2-(4-(heptyloxy)phenoxy)propyl)-4,5-dihydrothiazole-4-carboxylate (mixture of diastereomers)[3] (14h)

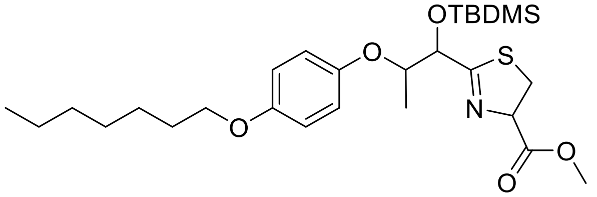

Yield 26% (120 mg); Pale yellow oil; **^{1}H-NMR** (400 MHz, $CDCl_3$): δ 6.92-6.71 (m, 4H, ArH), 5.23-5.05 (m, 1H, OCH), 4.87-4.77 (m, 1H, NCH), 4.64-4.50 (m, 1H, OCH), 3.89 (t, J = 6.6 Hz, 2H, OCH_2), 3.83-3.75 (m, 3H, OCH_3), 3.54-3.31 (m, 2H, SCH_2), 1.82-1.68 (m, 2H, CH_2), 1.50-1.21 (m, 11H, 4 x CH_2 and CH_3), 1.02-0.84 (m, 12H, $C(CH_3)_3$ and CH_3), 0.23-(-0.03) (m, 6H, $Si(CH_3)_2$). **^{13}C-NMR** (100 MHz, $CDCl_3$): δ 179.5, 171.2, 153.5, 153.3, 151.4, 151.4, 149.4, 117.6, 116.9, 116.8, 116.0, 115.6, 115.4, 115.2, 78.4, 78.2, 75.1, 74.9, 68.7, 68.6, 52.6, 52.6, 33.8, 33.7, 31.9, 31.8, 29.7,

29.4, 29.1, 26.0, 25.9, 25.7, 25.7, 22.6, 18.3, 14.2, 14.1, 14.1, -4.6, -4.7, -5.2, -5.2; **HRMS (ESI$^+$)** *m/z* calcd for $C_{27}H_{45}NNaO_5SSi^+$: 546.2680; $[M + Na]^+$ found: 546.2683.

Methyl 2-(1-(*tert*-butyldimethylsilyloxy)-2-(4-phenoxyphenoxy)propyl)-4,5-dihydrothiazole-4-carboxylate (mixture of diastereomers) (14i)

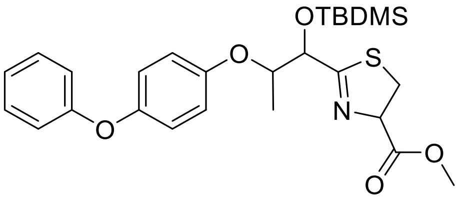

Yield 23% (115 mg); Pale yellow oil; **^{1}H-NMR** (200 MHz, $CDCl_3$): δ 7.37-7.22 (m, 2H, ArH), 7.09-6.75 (m, 7H, ArH), 5.25-5.05 (m, 1H, OCH), 4.91-4.80 (m, 1H, NCH), 4.72-4.56 (m, 1H, OCH), 3.87-3.73 (m, 3H, OCH_3), 3.52-3.29 (m, 2H, SCH_2), 1.35-1.20 (m, 3H, CH_3), 0.95 (s, 9H, $C(CH_3)_3$), 0.15-0.04 (m, 6H, $Si(CH_3)_2$); **^{13}C-NMR** (50 MHz, $CDCl_3$): δ 178.5, 170.4, 156.7, 153.6, 150.2, 129.6, 122.4, 120.8, 117.6, 116.9, 78.3, 78.0, 75.0, 74.7, 74.4, 52.7, 33.8, 25.7, 18.3, 14.1, -4.6, -5.2; **HRMS (ESI$^+$)** *m/z* calcd for $C_{26}H_{35}NNaO_5SSi^+$: 524.1897; $[M + Na]^+$ found: 524.1899.

Methyl 2-(1-((*tert*-butyldimethylsilyl)oxy)-2-(4-(hexylthio)phenoxy)propyl)-4,5-dihydrothiazole-4-carboxylate (mixture of diastereomers)[3] (14j)

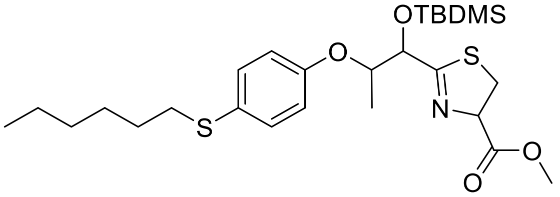

Yield 19% (100 mg); White oil; **^{1}H-NMR** (200 MHz, $CDCl_3$): δ 7.41-7.14 (m, 2H, ArH), 6.96-6.61 (m, 2H, ArH), 5.31-5.02 (m, 1H, OCH), 4.89-4.77 (m, 1H, NCH), 4.76-4.57 (m, 1H, OCH), 3.79 (s, 3H, OCH_3), 3.56-3.29 (m, 2H, SCH_2), 2.79 (t, $J = 7.0$ Hz, 2H, SCH_2), 1.73-1.15 (m, 11H, 4 x CH_2 and CH_3), 1.06-0.68 (m, 12H, $C(CH_3)_3$ and CH_3), 0.24-(-0.10) (m, 6H, $Si(CH_3)_2$); **^{13}C-NMR** (50 MHz, $CDCl_3$): δ 179.8, 171.2, 156.4, 133.1, 132.7, 116.1, 116.1, 116.0, 78.3, 78.0, 76.4, 74.8, 74.6, 52.7, 35.9, 35.6, 33.8, 33.6, 31.4, 29.3, 28.4, 25.7, 22.5, 18.2, 14.0, 13.9, -4.7, -5.3; **HRMS (ESI$^+$)** *m/z* calcd for $C_{26}H_{43}NNaO_4S_2Si^+$: 548.2295; $[M + Na]^+$ found: 548.2299.

Ethyl 2-(1-(*tert*-butyldimethylsilyloxy)-2-(4-octylphenoxy)ethyl)thiazole-4-carboxylate (15a)

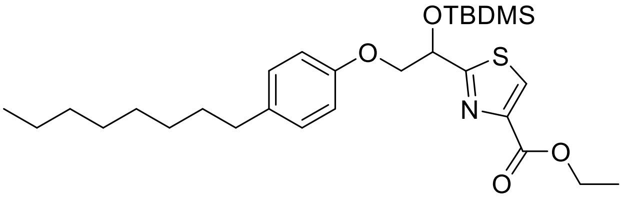

Yield 92% (478 mg); White oil; **^{1}H-NMR** (200 MHz, $CDCl_3$): δ 8.15 (s, 1H, ArH), 7.06 (d, J = 8.4 Hz, 2H, ArH), 6.80 (d, J = 8.4 Hz, 2H, ArH), 5.45 (dd, J = 7.9, 2.2 Hz, 1H, OCH), 4.55-4.30 (m, 3H, 3 x OC*H*H), 4.08-3.94 (m, 1H, OC*H*H), 2.52 (t, J = 7.8 Hz, 2H, $PhCH_2$), 1.66-1.46 (m, 2H, CH_2), 1.40 (t, J = 7.1 Hz, 3H, CH_3), 1.35-1.17 (m, 10H, 5 x CH_2), 1.00-0.79 (m, 12H, CH_3 and $C(CH_3)_3$), 0.24-(-0.04) (m, 6H, $Si(CH_3)_2$); **^{13}C-NMR** (50 MHz, $CDCl_3$): δ 174.3, 161.4, 156.4, 147.2, 135.3, 129.1, 127.6, 114.2, 72.7, 72.5, 61.4, 35.0, 31.8, 31.7, 29.4, 29.2, 25.7, 22.6, 18.2, 14.3, 14.0, -4.5, -5.2; **HRMS (ESI$^+$)** *m/z* calcd for $C_{28}H_{45}NNaO_4SSi^+$: 542.2731; $[M + Na]^+$ found: 542.2733.

Methyl 2-(1-(*tert*-butyldimethylsilyloxy)-2-(4-(heptyloxy)phenoxy)ethyl)thiazole-4-carboxylate (15b)

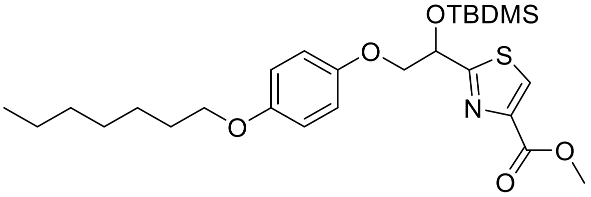

Yield 43% (218 mg); White oil; **^{1}H-NMR** (200 MHz, $CDCl_3$): δ 8.15 (s, 1H, ArH), 6.99-6.53 (m, 4H, ArH), 5.56-5.13 (m, 1H, OCH), 4.53-4.22 (m, 1H, OC*H*H), 4.12-3.60 (m, 6H, 3 x OC*H*H and OCH_3), 1.88-1.58 (m, 2H, CH_2), 1.51-1.10 (m, 8H, 4 x CH_2), 1.05-0.68 (m, 12H, $C(CH_3)_3$ and CH_3), 0.36-(-0.14) (m, 6H, $Si(CH_3)_2$); **^{13}C-NMR** (50 MHz, $CDCl_3$): δ 174.5, 161.9, 153.4, 152.4, 146.7, 128.0, 115.3, 115.2, 73.3, 72.5, 68.4, 52.4, 31.7, 29.3, 29.0, 25.9, 25.6, 22.5, 18.2, 14.0, -4.6, -5.3; **HRMS (ESI$^+$)** *m/z* calcd for $C_{26}H_{41}NNaO_5SSi^+$: 530.2367; $[M + Na]^+$ found: 530.2367.

Methyl 2-(1-(*tert*-butyldimethylsilyloxy)-2-(4-phenoxyphenoxy)ethyl)thiazole-4-carboxylate (15c)

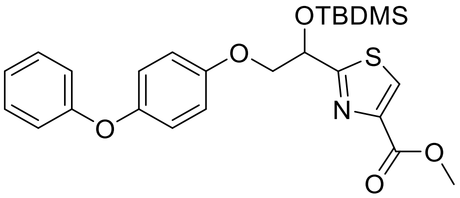

Yield 91% (442 mg); White oil; **^{1}H-NMR** (200 MHz, $CDCl_3$): δ 8.18 (s, 1H, ArH), 7.36-7.21 (m, 2H, ArH), 7.10-6.79 (m, 7H, ArH), 5.45 (dd, J = 7.8, 2.5 Hz, 1H, OCH), 4.43 (dd, J = 9.8, 2.5 Hz, 1H, OC*H*H), 4.14-4.01 (m, 1H, OC*H*H), 3.94 (s, 3H, OCH_3), 0.95 (s, 9H, $C(CH_3)_3$), 0.17 (s, 3H, $SiCH_3$), 0.15 (s, 3H, $SiCH_3$); **^{13}C-NMR** (50 MHz,

$CDCl_3$): δ 174.3, 161.8, 158.3, 154.6, 150.3, 146.8, 129.5, 128.0, 122.4, 120.7, 117.5, 115.6, 73.1, 72.5, 52.4, 25.6, 18.2, -4.5, -5.2; **HRMS (ESI$^+$)** *m/z* calcd for $C_{25}H_{31}NNaO_5SSi^+$: 508.1584; $[M + Na]^+$ found: 508.1583.

Methyl 2-(1-((*tert*-butyldimethylsilyl)oxy)-2-(4-(4-fluorophenoxy)phenoxy)ethyl)thiazole-4-carboxylate (15d)

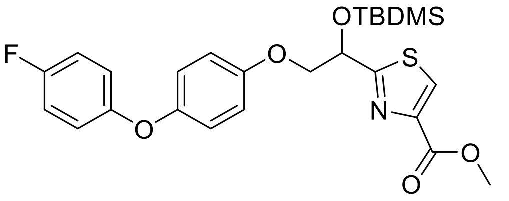

Yield 86% (433 mg); White oil; **^{1}H-NMR** (400 MHz, $CDCl_3$): δ 8.18 (s, 1H, ArH), 7.04-6.82 (m, 8H, ArH), 5.45 (dd, J = 7.8, 2.5 Hz, 1H, OCH), 4.42 (dd, J = 9.8, 2.5 Hz, 1H, OC*H*H), 4.08-4.01 (m, 1H, OC*H*H), 3.96 (s, 3H, OCH_3), 0.95, (s, 9H, $C(CH_3)_3$), 0.17 (s, 3H, $SiCH_3$), 0.15 (s, 3H, $SiCH_3$); **^{13}C-NMR** (100 MHz, $CDCl_3$): δ 174.4, 162.9 (d, J = 238.5 Hz), 154.7, 151.0, 150.4, 146.9, 128.0, 120.1, 119.2 (d, J = 8.0 Hz), 116.1 (d, J = 23.3 Hz), 73.3, 72.6, 52.5, 25.7, 18.3, -4.5, -5.1; **^{19}F-NMR** (377 MHz, $CDCl_3$): δ -121.26; **HRMS (ESI$^+$)** *m/z* calcd for $C_{25}H_{30}FNNaO_5SSi^+$: 526.1490; $[M + Na]^+$ found: 526.1494.

Methyl 2-(1-(*tert*-butyldimethylsilyloxy)-2-(4-(hexylthio)phenoxy)ethyl)thiazole-4-carboxylate (15e)

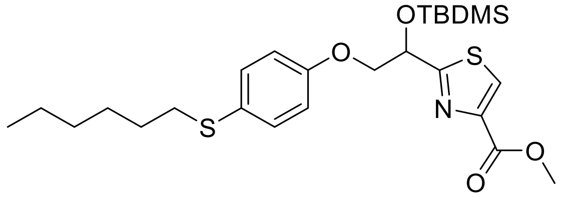

Yield 90% (459 mg); White oil; **^{1}H-NMR** (200 MHz, $CDCl_3$): δ 8.17 (s, 1H, ArH), 7.28 (d, J = 8.7 Hz, 2H, ArH), 6.81 (d, J = 8.7 Hz, 2H, ArH), 5.42 (dd, J = 7.7, 2.1 Hz, 1H, OCH), 4.41 (dd, J = 9.7, 2.2 Hz, 1H, OC*H*H), 4.11-3.98 (m, 1H, OC*H*H), 3.93 (s, 3H, OCH_3), 2.79 (t, J = 7.0 Hz, 2H, SCH_2), 1.64-1.44 (m, 2H, CH_2), 1.31-1.12 (m, 6H, 3 x CH_2), 0.97-0.72 (m, 12H, $C(CH_3)_3$ and CH_3), 0.21-(-0.02) (m, 6H, $Si(CH_3)_2$); **^{13}C-NMR** (50 MHz, $CDCl_3$): δ 174.2, 161.8, 157.4, 146.8, 132.6, 128.0, 127.4, 115.0, 72.7, 72.4, 52.5, 35.6, 31.3, 29.2, 28.3, 25.6, 22.7, 22.5, 18.2, 14.0, -4.5, -5.2; **HRMS (ESI$^+$)** *m/z* calcd for $C_{25}H_{39}NNaO_4S_2Si^+$: 532.1982; $[M + Na]^+$ found: 532.1982.

Methyl 2-(1-(*tert*-butyldimethylsilyloxy)-2-(4-(hexyloxy)phenylthio)ethyl)thiazole-4-carboxylate (15f)

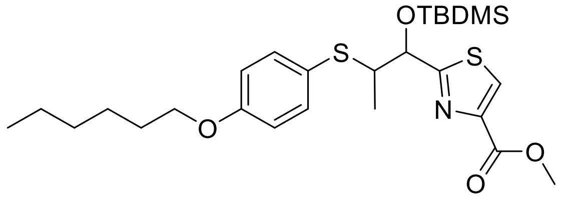

Yield 83% (435 mg); White oil; **^{1}H-NMR** (200 MHz, $CDCl_3$): δ 8.13 (s, 1H, ArH), 7.29 (d, J = 8.8 Hz, 2H, ArH), 6.77 (d, J = 8.8 Hz, 2H, ArH), 5.28 (dd, J = 6.4, 4.0 Hz, 1H, OCH), 3.99-3.82 (m, 5H, OCH_2 and OCH_3), 3.42-3.22 (m, 2H, 2 x SC*H*H), 1.87-1.68 (m, 2H, CH_2), 1.40-1.19 (m, 6H, 3 x CH_2), 0.99-0.76 (m, 12H, $C(CH_3)_3$ and CH_3), 0.14 (s, 3H, $SiCH_3$), 0.01 (s, 3H, $SiCH_3$); **^{13}C-NMR** (50 MHz, $CDCl_3$): δ 176.2, 161.8, 158.4, 146.4, 133.1, 127.8, 126.1, 115.0, 72.2, 68.0, 52.4, 45.2, 31.5, 29.6, 29.1, 25.7, 22.6, 18.1, 14.0, -4.7, -4.9; **HRMS (ESI$^+$)** *m/z* calcd for $C_{226}H_{41}NNaO_4S_2Si^+$: 546.2138; $[M + Na]^+$ found: 546.2138.

Methyl 2-(1-(*tert*-butyldimethylsilyloxy)-2-(4-octylphenoxy)propyl)thiazole-4-carboxylate (15g)

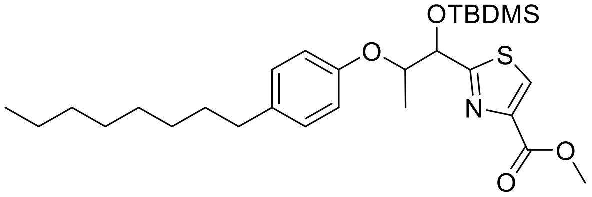

Yield 67% (348 mg); White oil; **^{1}H-NMR** (200 MHz, $CDCl_3$): δ 8.16 (s, 1H, ArH), 7.07 (d, J = 8.6 Hz, 2H, ArH), 6.87 (d, J = 8.6 Hz, 2H, ArH), 5.38 (d, J = 2.5 Hz, 1H, OCH), 4.90-4.75 (m, 1H, OCH), 3.96 (s, 3H, OCH_3), 2.53 (t, J = 7.8 Hz, 2H, $PhCH_2$), 1.63-1.49 (m, 2H, CH_2), 1.38-1.22 (m, 10H, 5 x CH_2), 1.14 (d, J = 6.2 Hz, 3H, CH_3), 0.96 (s, 9H, $C(CH_3)_3$), 0.87 (t, J = 6.4 Hz, 3H, CH_3), 0.18 (s, 3H, $SiCH_3$), 0.04 (s, 3H, $SiCH_3$); **HRMS (ESI$^+$)** *m/z* calcd for $C_{28}H_{45}NNaO_4SSi^+$: 542.2731; $[M + Na]^+$ found: 542.2735.

Methyl 2-(1-((*tert*-butyldimethylsilyl)oxy)-2-(4-(heptyloxy)phenoxy)propyl)thiazole-4-carboxylate (mixture of diastereomers) (15h)

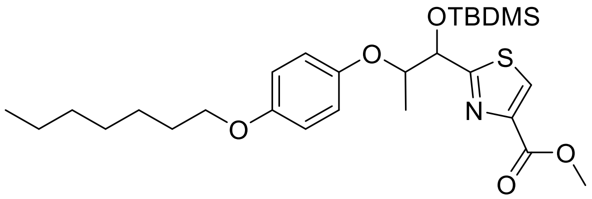

Yield 65% (339 mg); White oil; **^{1}H-NMR** (400 MHz, $CDCl_3$): δ 8.15 (s, 1H, ArH), 6.89 (d, J = 8.9 Hz, 2H, ArH), 6.81 (d, J = 8.9 Hz, 2H, ArH), 5.40-5.33 (m, 1H, OCH),

4.80-4.70 (m, 1H, OCH), 3.96 (s, 3H, OCH_3), 3.90 (t, J = 6.5 Hz, 2H, OCH_2), 1.81-1.70 (m, 2H, CH_2), 1.38-1.23 (m, 8H, 4 x CH_2), 1.14 (d, J = 6.2 Hz, 3H, CH_3), 0.97 (s, 9H, $C(CH_3)_3$), 0.89-0.86 (m, 3H, CH_3), 0.18 (s, 3H, $SiCH_3$), 0.04z (s, 3H, $SiCH_3$); **^{13}C-NMR** (100 MHz, $CDCl_3$): δ 175.3, 171.5, 161.2, 153.6, 151.3, 146.9, 127.8, 117.0, 115.5, 77.2, 75.0, 68.6, 52.4, 31.8, 29.4, 29.1, 26.0, 25.8, 22.6, 18.3, 14.1, 13.0, -4.5, -5.3; **HRMS (ESI$^+$)** *m/z* calcd for $C_{27}H_{43}NNaO_5SSi^+$: 544.2523; $[M + Na]^+$ found: 544.2526.

Methyl 2-(1-(*tert*-butyldimethylsilyloxy)-2-(4-phenoxyphenoxy)propyl)thiazole-4-carboxylate (15i)

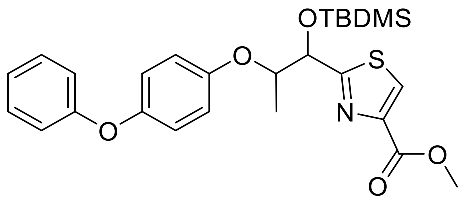

Yield 54% (270 mg); White oil; **^{1}H-NMR** (200 MHz, $CDCl_3$): δ 8.16 (s, 1H, ArH), 7.38-7.22 (m, 2H, ArH), 7.14-6.80 (m, 7H, ArH), 5.39 (d, J = 2.4 Hz, 1H, OCH), 4.88-4.78 (m, 1H, OCH), 3.95 (s, 3H, OCH_3), 1.18 (d, J = 6.2 Hz, 3H, CH_3), 0.97 (s, 9H, $C(CH_3)_3$), 0.19 (s, 3H, $SiCH_3$), 0.05 (s, 3H, $SiCH_3$); **^{13}C-NMR** (50 MHz, $CDCl_3$): δ 175.0, 161.9, 158.2, 153.6, 150.3, 146.8, 129.6, 127.9, 122.4, 120.8, 117.6, 116.9, 77.3, 74.9, 52.4, 25.7, 18.3, 14.1, -4.5, -5.3; **HRMS (ESI$^+$)** *m/z* calcd for $C_{26}H_{34}NO_5SSi^+$: 500.1921; $[M + H]^+$ found: 500.1922.

Methyl 2-(1-(*tert*-butyldimethylsilyloxy)-2-(4-(hexylthio)phenoxy)propyl)thiazole-4-carboxylate (mixture of diastereomers) (15j)

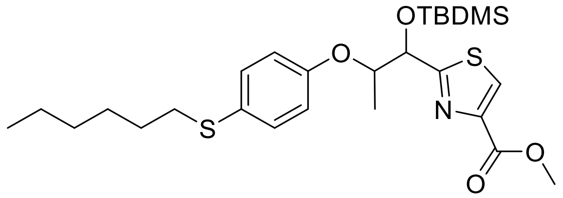

Yield 92% (482 mg); White oil; **^{1}H-NMR** (200 MHz, $CDCl_3$): δ 8.16 (s, 1H, ArH), 7.31 (d, J = 7.3 Hz, 2H, ArH), 6.89 (d, J = 7.3 Hz, 2H, ArH), 5.48-5.27 (m, 1H, OCH), 5.02-4.73 (m, 1H, OCH), 3.94 (s, 3H, OCH_3), 2.94-2.71 (m, 2H, SCH_2), 1.72-1.21 (m, 11H, 4 x CH_2 and CH_3), 1.11-0.74 (m, 12H, $C(CH_3)_3$ and CH_3), 0.09 (d, J = 26.5 Hz, 6H, $Si(CH_3)_2$); **^{13}C-NMR** (50 MHz, $CDCl_3$): δ 174.9, 160.2, 156.4, 146.9, 133.1, 132.8, 127.8, 116.2, 76.7, 74.7, 52.4, 35.6, 31.3, 29.3, 28.4, 25.7, 22.5, 18.2, 14.0, 12.8, -4.5, -5.3; **HRMS (ESI$^+$)** *m/z* calcd for $C_{26}H_{41}NNaO_4S_2Si^+$: 546.2138; $[M + Na]^+$ found: 546.2140.

Ethyl 2-(1-hydroxy-2-(4-octylphenoxy)ethyl)thiazole-4-carboxylate (16a)

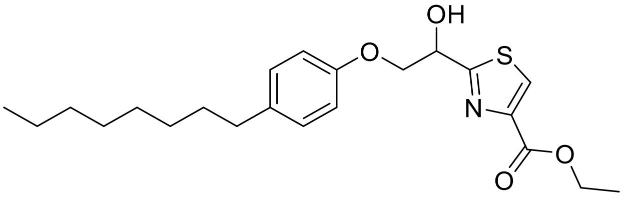

Yield 91% (369 mg); White solid m.p.: 55-57 °C; **^{1}H-NMR** (200 MHz, $CDCl_3$): δ 8.15 (s, 1H, ArH), 7.06 (d, J = 8.2 Hz, 2H, ArH), 6.81 (d, J = 8.2 Hz, 2H, ArH), 5.44 (dd, J = 6.0, 3.5 Hz, 1H, OCH), 4.54-4.31 (m, 3H, 3 x OC*H*H), 4.26-4.14 (m, 1H, OC*H*H), 4.09 (br s, 1H, OH), 2.52 (t, J = 7.6 Hz, 1H, $PhCH_2$), 1.66-1.45 (m, 2H, CH_2), 1.38 (t, J = 7.1, CH_3), 1.33-1.17 (m, 10H, 5 x CH_2), 0.87 (t, J = 6.0 Hz, 3H, CH_3); **^{13}C-NMR** (50 MHz, $CDCl_3$): δ 172.3, 161.3, 155.9, 146.9, 136.0, 129.2, 127.9, 114.4, 71.2, 70.6, 61.4, 35.0, 31.8, 31.6, 29.4, 29.2, 22.6, 14.3, 14.0; **HRMS (ESI$^+$)** *m/z* calcd for $C_{22}H_{31}NNaO_4S^+$: 428.1866; $[M + Na]^+$ found: 428.1870.

Methyl 2-(2-(4-(heptyloxy)phenoxy)-1-hydroxyethyl)thiazole-4-carboxylate (16b)

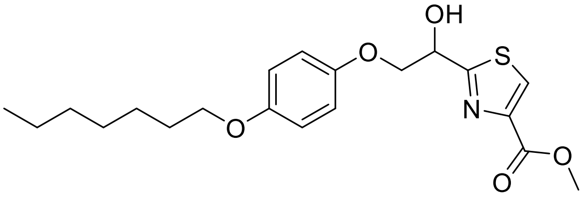

Yield 93% (366 mg); White solid m.p.: 111-113 °C; **^{1}H-NMR** (200 MHz, $CDCl_3$): δ 8.20 (s, 1H, ArH), 6.99-6.63 (m, 4H, ArH), 5.40 (dd, J = 6.3, 4.0 Hz, 1H, OCH), 4.44

(dd, J = 9.4, 3.7 Hz, 1H, OC*H*H), 4.24-4.10 (m, 1H, OC*H*H), 4.00-3.82 (m, 4H, OCH_3 and OC*H*H), 3.54-3.41 (m, 1H, OC*H*H), 2.71 (br s, 1H, OH), 1.87-1.60 (m, 2H, CH_2), 1.51-1.11 (m, 8H, 4 x CH_2), 0.88 (t, J = 6.3 Hz, 3H, CH_3); **^{13}C-NMR** (50 MHz, $CDCl_3$): δ 172.3, 171.8, 161.7, 157.2, 153.9, 151.8, 128.2, 115.8, 115.3, 72.0, 70.8, 68.5, 52.5, 31.7, 29.3, 29.0, 25.9, 22.6, 14.1; **HRMS (ESI$^+$)** *m/z* calcd for $C_{20}H_{27}NNaO_5S^+$: 416.1502; $[M + Na]^+$ found: 416.1500.

Methyl 2-(1-hydroxy-2-(4-phenoxyphenoxy)ethyl)thiazole-4-carboxylate (16c)

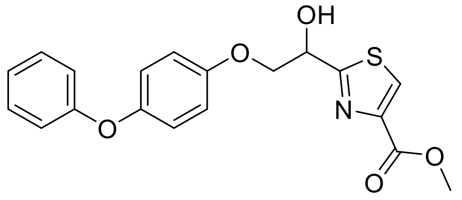

Yield 75% (279 mg); White solid m.p.: 130-132 ºC; **^{1}H-NMR** (400 MHz, $CDCl_3$): δ 8.21 (s, 1H, ArH), 7.34-7.28 (m, 2H, ArH), 7.12-6.80 (m, 7H, ArH), 5.50-5.38 (m, 1H, OCH), 4.48 (dd, J = 9.6, 3.7 Hz, 1H, OC*H*H), 4.24 (dd, J = 9.6, 6.8 Hz, 1H, OC*H*H), 3.96 (s, 3H, OCH_3), 3.39 (br s, 1H, OH); **^{13}C-NMR** (100 MHz, $CDCl_3$): δ 171.9, 161.8, 158.2, 154.1, 151.2, 146.8, 129.7, 128.2, 122.7, 120.7, 117.9, 116.0, 71.8, 70.8, 52.5; **HRMS (ESI$^+$)** *m/z* calcd for $C_{19}H_{17}NNaO_5S^+$: 394.0720; $[M + Na]^+$ found: 394.0720.

Methyl 2-(2-(4-(4-fluorophenoxy)phenoxy)-1-hydroxyethyl)thiazole-4-carboxylate[3] (16d)

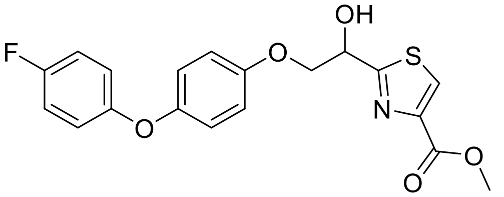

Yield 95% (370 mg); White solid m.p.: 110-112 ºC; **^{1}H-NMR** (200 MHz, $CDCl_3$): δ 8.19 (s, 1H, ArH), 7.09-6.79 (m, 8H, ArH), 5.43 (dd, J = 6.3, 3.0 Hz, 1H, OCH), 4.46 (dd, J = 9.6, 2.7 Hz, 1H, OC*H*H), 4.28-4.14 (m, 1H, OC*H*H), 3.92 (s, 3H OCH_3), 3.62 (br s, 1H, OH); **^{13}C-NMR** (50 MHz, $CDCl_3$): δ 172.3, 161.8, 154.0, 153.7 (d, J = 233.8 Hz), 146.0, 136.2, 128.2, 120.0, 119.3 (d, J = 8.2 Hz), 116.1 (d, J = 23.1 Hz), 115.9, 71.8, 70.7, 52.5; **^{19}F-NMR** (377 MHz, $CDCl_3$): δ -120.92; **HRMS (ESI$^+$)** *m/z* calcd for $C_{19}H_{16}FNNaO_5S^+$: 412.0625; $[M + Na]^+$ found: 412.0628.

Methyl 2-(2-(4-(hexylthio)phenoxy)-1-hydroxyethyl)thiazole-4-carboxylate (16e)

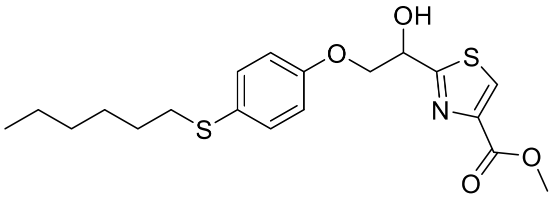

Yield 75% (279 mg); White solid m.p.: 75-77 °C; **^{1}H-NMR** (200 MHz, $CDCl_3$): δ 8.18 (s, 1H, ArH), 7.28 (d, J = 8.6 Hz, 2H, ArH), 6.82 (d, J = 8.6 Hz, 2H, ArH), 5.51-5.32 (m, 1H, OCH), 4.46 (d, J = 9.0 Hz, 1H, OC*H*H), 4.31-4.11 (m, 1H, OC*H*H), 3.92 (s, 3H, OCH_3), 3.60 (br s, 1H, OH), 2.79 (t, J = 7.3 Hz, 2H, SCH_2), 1.66-1.12 (m, 8H, 4 x CH_2), 0.85 (t, J = 6.6 Hz, 3H, CH_3), **^{13}C-NMR** (50 MHz, $CDCl_3$): δ 172.2, 161.7, 156.8, 146.5, 132.3, 128.2, 115.2, 71.3, 70.6, 52.5, 35.3, 31.3, 29.1, 28.3, 22.4, 13.9; **HRMS (ESI$^+$)** *m/z* calcd for $C_{19}H_{25}NNaO_4S_2^+$: 418.1117; $[M + Na]^+$ found: 418.1122.

Methyl 2-(2-(4-(hexyloxy)phenylthio)-1-hydroxyethyl)thiazole-4-carboxylate (16f)

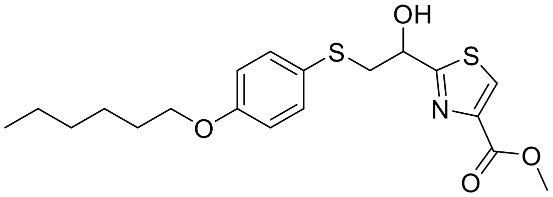

Yield 90% (356 mg); White solid m.p.: 66-68 °C; **^{1}H-NMR** (200 MHz, $CDCl_3$): δ 8.05 (s, 1H, ArH), 7.32 (d, J = 8.2 Hz, 2H, ArH), 6.74 (d, J = 8.2 Hz, 2H, ArH), 4.90 (dd, J = 9.0, 3.6 Hz, 1H, OCH), 3.93-3.72 (m, 5H, OCH_2 and OCH_3), 3.46 (dd, J = 14.0, 3.6 Hz, 1H, SC*H*H), 2.97 (dd, J = 14.0, 9.0 Hz, 1H, SC*H*H), 1.80-1.60 (m, 2H, CH_2), 1.48-1.20 (m, 6H, 3 x CH_2), 0.83 (t, J = 6.2 Hz, 3H, CH_3); **^{13}C-NMR** (50 MHz, $CDCl_3$): δ 175.5, 174.0, 161.7, 159.4, 156.7, 146.6, 134.8, 127.8, 122.6, 115.4, 69.2, 68.1, 52.4, 44.4, 31.5, 29.1, 25.6, 22.6, 14.0; **HRMS (ESI$^+$)** *m/z* calcd for $C_{19}H_{26}NO_4S_2^+$ $[M + H]^+$: 396.1298. Found: 396.1298.

Methyl 2-(1-hydroxy-2-(4-octylphenoxy)propyl)thiazole-4-carboxylate (16g)

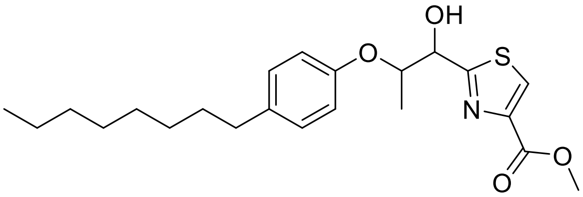

Yield 73% (296 mg); White solid m.p.: 87-89 °C; **^{1}H-NMR** (400 MHz, $CDCl_3$): δ 8.18 (s, 1H, ArH), 7.09 (d, J = 8.5 Hz, 2H, ArH), 6.89 (d, J = 8.5 Hz, 2H, ArH), 5.39-5.25 (m, 1H, OCH), 4.94-4.76 (m, 1H, OCH), 3.95 (s, 3H, OCH_3), 2.54 (t, J = 8.0 Hz, 2H, $PhCH_2$), 1.69-1.47 (m, 2H, CH_2), 1.41-1.09 (m, 13H, 5 x CH_2 and CH_3), 0.88 (t, J = 6.0

Hz, 3H, CH_3); **^{13}C-NMR** (100 MHz, $CDCl_3$): δ 171.7, 161.8, 154.6, 146.5, 136.5, 129.5, 129.4, 128.2, 116.3, 115.0, 76.3, 73.5, 52.5, 35.1, 31.9, 31.6, 29.5, 29.3, 22.7, 14.1, 13.7; **HRMS (ESI$^+$)** *m/z* calcd for $C_{22}H_{31}NNaO_4S^+$: 428.1866; [M + Na]$^+$ found: 428.1866.

Methyl 2-(2-(4-(heptyloxy)phenoxy)-1-hydroxypropyl)thiazole-4-carboxylate (mixture of diastereomers) (16h)

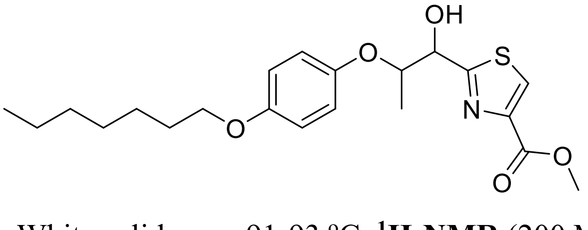

Yield 76% (310 mg); White solid m.p.: 91-93 °C; **^{1}H-NMR** (200 MHz, $CDCl_3$): δ 8.17 (s, 1H, ArH), 6.92 (d, J = 8.9 Hz, 2H, ArH), 6.83 (d, J = 8.9 Hz, 2H, ArH), 5.35-5.29 (m, 1H, OCH), 5.11-5.06 (m, 1H, OCH), 3.97 (s, 3H, OCH_3), 3.91 (t, J = 6.4 Hz, 2H, OCH_2), 1.81-1.72 (m, 2H, CH_2), 1.50-1.25 (m, 11H, 4 x CH_2 and CH_3), 0.91 (t, J = 6.2 Hz, 3H, CH_3); **^{13}C-NMR** (50 MHz, $CDCl_3$): δ 174.5, 161.8, 153.4, 152.4, 146.7, 127.8, 116.0, 115.6, 76.2, 73.8, 68.7, 60.4, 51.0, 31.8, 29.1, 26.0, 25.8, 22.6, 14.1; **HRMS (ESI$^+$)** *m/z* calcd for $C_{21}H_{29}NNaO_5S^+$: $4^30.1659$; [M + Na]$^+$ found: 430.1657.

Methyl 2-(1-hydroxy-2-(4-phenoxyphenoxy)propyl)thiazole-4-carboxylate (16i)

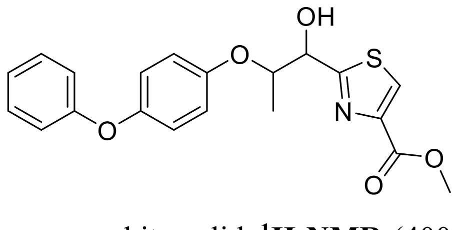

Yield 69% (266 mg); Low m.p. white solid; **^{1}H-NMR** (400 MHz, $CDCl_3$): δ 8.19 (s, 1H, ArH), 7.37-7.25 (m, 2H, ArH), 7.12-6.90 (m, 7H, ArH), 5.33 (d, J = 4.0 Hz, 1H, OCH), 4.90-4.77 (m, 1H, OCH), 3.95 (s, 3H, OCH_3), 1.27-1.24 (m, 3H, CH_3), 1.22 (br s, 1H, OH); **^{13}C-NMR** (100 MHz, $CDCl_3$): δ 174.8, 170.7, 161.8, 158.0, 151.4, 146.5, 129.7, 129.6, 128.1, 122.8, 122.5, 121.0, 120.6, 118.0, 117.6, 117.0, 116.3, 74.9, 52.6, 23.0; **HRMS (ESI$^+$)** *m/z* calcd for $C_{20}H_{19}NNaO_5S^+$: 408.0876; [M + Na]$^+$ found: 408.0874.

Methyl 2-(2-(4-(hexylthio)phenoxy)-1-hydroxypropyl)thiazole-4-carboxylate (mixture of diastereomers) (16j)

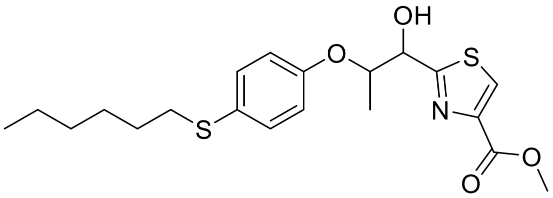

Yield 87% (356 mg); White oil; **^{1}H-NMR** (200 MHz, $CDCl_3$): δ 8.18 (s, 1H, ArH), 7.31 (d, J = 8.8 Hz, 2H, ArH), 6.90 (d, J = 8.8 Hz, 2H, ArH), 5.31 (d, J = 4.0 Hz, 1H, OCH), 4.95-4.79 (m, 1H, OCH), 3.94 (s, 3H, OCH_3), 2.82 (t, J = 7.2 Hz, 2H, SCH_2), 1.68-1.49 (m, 2H, CH_2), 1.49-1.17 (m, 9H, 3 x CH_2 and CH_3), 0.87 (t, J = 6.6 Hz, 3H, CH_3). **^{13}C-NMR** (50 MHz $CDCl_3$): δ 171.6, 161.9, 154.2, 146.7, 127.8, 115.8, 76.6, 72.8, 51.9, 35.4, 31.5, 29.1, 25.6, 22.6, 14.0; **HRMS (ESI$^+$)** *m/z* calcd for $C_{20}H_{27}NNaO_4S_2^+$: 432.1274; [M + Na]$^+$ found: 432.1277.

Ethyl 2-(2-(4-octylphenoxy)acetyl)thiazole-4-carboxylate (17a, GK401)

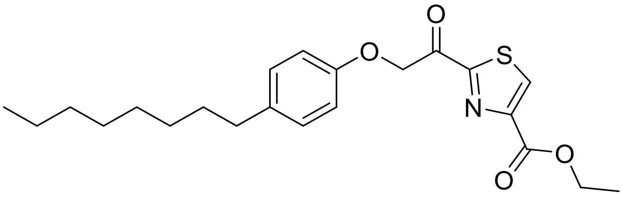

Yield 78% (315 mg); White waxy solid m.p.: 63-65 °C; **^{1}H-NMR** (200 MHz, $CDCl_3$): δ 8.49 (s, 1H, ArH), 7.10 (d, J = 7.2 Hz, 2H, ArH), 6.91 (d, J = 7.2 Hz, 2H, ArH), 5.57 (s, 2H, OCH_2), 4.46 (q, J = 7.1 Hz, 2H, OCH_2), 2.53 (t, J = 7.5 Hz, 2H, $PhCH_2$), 1.66-1.51 (m, 2H, CH_2), 1.43 (t, J = 7.1 Hz, 3H, CH_3), 1.36-1.20 (m, 10H, 5 x CH_2), 0.87 (t, J = 7.0 Hz, 3H, CH_3); **^{13}C-NMR** (50 MHz, $CDCl_3$): δ 187.7, 164.4, 160.5, 155.7, 149.0, 136.3, 133.4, 129.3, 114.7, 70.3, 62.0, 35.0, 31.8, 31.6, 29.4, 29.2, 22.6, 14.3, 14.1; **HRMS (ESI$^+$)** *m/z* calcd for $C_{22}H_{29}NNaO_4S^+$: 426.1710; [M + Na]$^+$ found: 426.1709.

Methyl 2-(2-(4-(heptyloxy)phenoxy)acetyl)thiazole-4-carboxylate (17b, GK420)

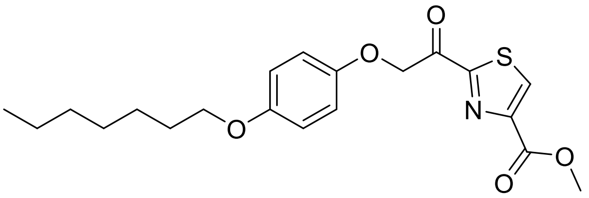

Yield 79% (309 mg); Pale yellow solid m.p.: 120-122 °C; **^{1}H-NMR** (200 MHz, $CDCl_3$): δ 8.50 (s, 1H, ArH), 6.93 (d, J = 9.0 Hz, 2H, ArH), 6.81 (d, J = 9.0 Hz, 2H, ArH), 5.53 (s, 2H, OCH_2), 3.98 (s, 3H, OCH_3), 3.88 (t, J = 6.5 Hz, 2H, OCH_2), 1.84-1.64 (m, 2H, CH_2), 1.50-1.20 (m, 8H, 4 x CH_2), 0.87 (t, J = 6.1 Hz, 3H, CH_3); **^{13}C-NMR** (50 MHz, $CDCl_3$): δ 187.8, 164.4, 161.0, 154.1, 151.7, 148.5, 133.6, 116.2, 115.3, 71.1, 68.4,

52.8, 31.7, 29.3, 29.0, 26.0, 22.6, 14.1; **HRMS (ESI$^+$)** *m/z* calcd for $C_{20}H_{25}NNaO_5S^+$: 414.1346; $[M + Na]^+$ found: 414.1357.

Methyl 2-(2-(4-phenoxyphenoxy)acetyl)thiazole-4-carboxylate (17c, GK403)

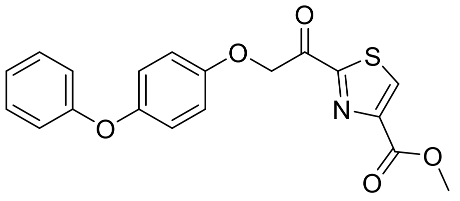

Yield 71% (262 mg); White solid m.p.: 148-150 °C; **^{1}H-NMR** (200 MHz, $CDCl_3$): δ 8.51 (s, 1H, ArH), 7.38-7.23 (m, 2H, ArH), 7.13-6.85 (m, 7H, ArH), 5.58 (s, 2H, OCH_2), 3.99 (s, 3H OCH_3); **^{13}C-NMR** (50 MHz, $CDCl_3$): δ 187.5, 164.3, 160.9, 158.0, 153.9, 151.2, 148.6, 133.7, 129.6, 122.6, 120.6, 117.8, 116.2, 70.8, 52.8; **HRMS (ESI$^+$)** *m/z* calcd for $C_{19}H_{15}NNaO_5S^+$: 392.0563; $[M + Na]^+$ found: 392.0562.

Methyl 2-(2-(4-(4-fluorophenoxy)phenoxy)acetyl)thiazole-4-carboxylate (17d, GK440)

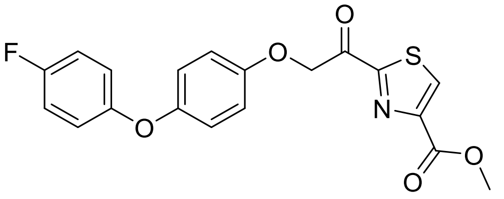

Yield 85% (329 mg); White solid m.p.: 139-141 °C; **^{1}H-NMR** (600 MHz, $CDCl_3$): δ 8.51 (s, 1H, ArH), 7.02-6.95 (m, 4H, ArH), 6.95-6.89 (m, 4H, ArH), 5.58 (s, 2H, OCH_2), 4.00 (s, 3H, OCH_3); **^{13}C-NMR** (150 MHz, $CDCl_3$): δ 187.5, 164.4, 161.0, 158.5 (d, J = 241.0 Hz), 153.9, 153.8, 151.8, 148.7, 133.6, 120.0, 119.5 (d, J = 8.2 Hz), 116.4, 116.1 (d, J = 23.3 Hz), 70.9, 52.8; **HRMS (ESI$^+$)** *m/z* calcd for $C_{19}H_{14}FNNaO_5S^+$: 410.0469; $[M + Na]^+$ found: 410.0477.

Methyl 2-(2-(4-(hexylthio)phenoxy)acetyl)thiazole-4-carboxylate (17e, GK427)

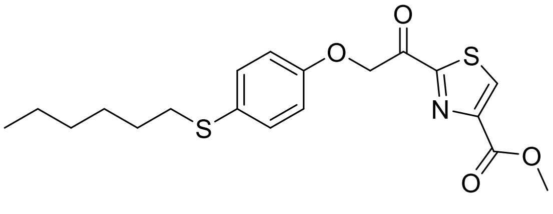

Yield 69% (271 mg); Pale yellow solid m.p.: 89-90 °C; **^{1}H-NMR** (200 MHz, $CDCl_3$): δ 8.52 (s, 1H, ArH), 7.32 (d, J = 8.6 Hz, 2H, ArH), 6.93 (d, J = 8.6 Hz, 2H, ArH), 5.58 (s, 2H, OCH_2), 4.01 (s, 3H, OCH_3), 2.82 (t, J = 7.2 Hz, 2H, SCH_2), 1.65-1.48 (m, 2H, CH_2), 1.32-1.18 (m, 6H, 3 x CH_2), 0.87 (t, J = 6.6 Hz, 3H, CH_3); **^{13}C-NMR** (50 MHz, $CDCl_3$): δ 187.3, 164.3, 161.0, 156.6, 148.6, 133.7, 132.4, 128.6, 115.5, 70.2, 52.9,

35.4, 31.3, 29.2, 28.4, 22.5, 14.0; **HRMS (ESI$^+$)** *m/z* calcd for $C_{19}H_{23}NNaO_4S_2^+$: 416.0961; $[M + Na]^+$ found: 416.0961.

Methyl 2-(2-(4-(hexyloxy)phenylthio)acetyl)thiazole-4-carboxylate (17f, GK419)

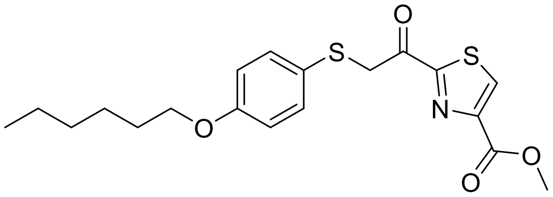

Yield 77% (303 mg); Pale yellow solid m.p.: 74-76 ºC; **^{1}H-NMR** (200 MHz, $CDCl_3$): δ 8.46 (s, 1H, ArH), 7.39 (d, J = 8.8 Hz, 2H, ArH), 6.81 (d, J = 8.8 Hz, 2H, ArH), 4.32 (s, 2H, OCH_2), 3.98 (s, 3H, OCH_3), 3.92 (t, J = 6.6 Hz, 2H, SCH_2), 1.86-1.68 (m, 2H, CH_2), 1.52-1.27 (m, 6H, 3 x CH_2), 0.90 (t, J = 6.6 Hz, 3H, CH_3); **^{13}C-NMR** (50 MHz, $CDCl_3$): δ 187.0, 166.2, 161.2, 159.5, 148.3, 135.0, 133.7, 123.6, 115.2, 68.0, 52.7, 42.0, 31.5, 29.1, 25.7, 22.6, 14.0; **HRMS (ESI$^+$)** *m/z* calcd for $C_{19}H_{23}NNaO_4S_2^+$: 416.0961; $[M + Na]^+$ found: 416.0961.

Methyl 2-(2-(4-octylphenoxy)propanoyl)thiazole-4-carboxylate (17g, GK402)

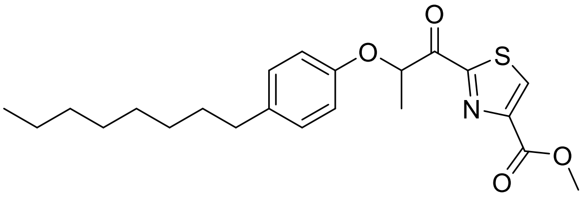

Yield 84% (339 mg); White solid m.p.: 97-99 ºC; **^{1}H-NMR** (200 MHz, $CDCl_3$): δ 8.51 (s, 1H, ArH), 7.04 (d, J = 8.6 Hz, 2H, ArH), 6.83 (d, J = 8.6 Hz, 2H, ArH), 6.03 (q, J = 6.8 Hz, 1H, OCH), 4.01 (s, 3H, OCH_3), 2.50 (t, J = 7.8 Hz, 2H, $PhCH_2$), 1.75 (d, J = 6.8 Hz, 3H, CH_3), 1.60-1.44 (m, 2H, CH_2), 1.36-1.20 (m, 10H, 5 x CH_2), 0.87 (t, J = 6.8 Hz, 3H, CH_3); **^{13}C-NMR** (50 MHz, $CDCl_3$): δ 191.8, 164.7, 161.1, 155.1, 148.7, 136.1, 133.9, 129.3, 115.2, 74.9, 52.8, 35.0, 31.9, 31.6, 29.4, 29.2, 22.6, 18.7, 14.1; **HRMS (ESI$^+$)** *m/z* calcd for $C_{22}H_{29}NNaO_4S^+$: 426.1710; $[M + Na]^+$ found: 426.1716.

Methyl 2-(2-(4-(heptyloxy)phenoxy)propanoyl)thiazole-4-carboxylate (17h, GK439)

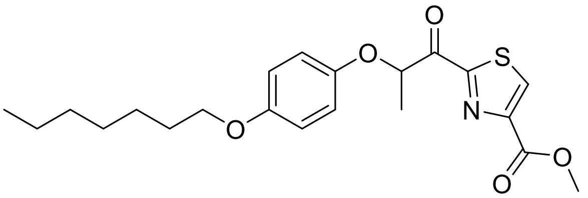

Yield 87% (352 mg); Pale yellow solid m.p.: 119-121 ºC; **^{1}H-NMR** (600 MHz, $CDCl_3$): δ 8.48 (s, 1H, ArH), 6.87 (d, J = 9.0 Hz, 2H, ArH), 6.77 (d, J = 9.0 Hz, 2H, ArH), 5.96

(q, J = 6.6 Hz, 1H, OCH), 4.00 (s, 3H, OCH_3), 3.86 (t, J = 6.6 Hz, 2H, OCH_2), 1.76-1.69 (m, 5H, CH_2 and CH_3), 1.64-1.53 (m, 2H, CH_2), 1.45-1.38 (m, 2H, CH_2), 1.35-1.27 (m, 4H, 2 x CH_2), 0.88 (t, J = 6.6 Hz, 3H, CH_3); **^{13}C-NMR** (150 MHz, $CDCl_3$): δ 191.9, 164.9, 161.1, 154.2, 151.2, 148.7, 133.7, 117.0, 115.4, 76.0, 68.5, 52.7, 31.8, 29.3, 29.0, 26.0, 22.6, 18.6, 14.1; **HRMS (ESI$^+$)** *m/z* calcd for $C_{21}H_{27}NNaO_5S^+$: 428.1502; $[M + Na]^+$ found: 428.1514.

Methyl 2-(2-(4-phenoxyphenoxy)propanoyl)thiazole-4-carboxylate (17i, GK428)

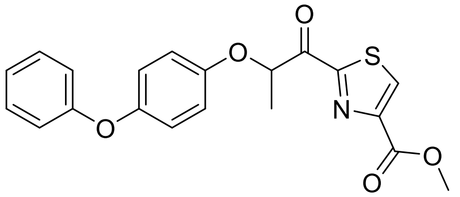

Yield 87% (333 mg); Off-white solid m.p.: 55-57 ºC ; **^{1}H-NMR** (600 MHz, $CDCl_3$): δ 8.50 (s, 1H, ArH), 7.28 (t, J = 9.0 Hz, 2H, ArH), 7.04 (t, J = 9.0 Hz, 1H, ArH), 6.96-6.86 (m, 6H, ArH), 6.02 (q, J = 6.8 Hz, 1H, OCH), 4.00 (s, 3H, OCH_3), 1.76 (d, J = 6.8 Hz, 3H, CH_3); **^{13}C-NMR** (150 MHz, $CDCl_3$): δ 191.6, 164.8, 161.1, 158.1, 153.4, 151.2, 148.8, 133.8, 129.6, 122.7, 120.5, 117.9, 116.9, 75.6, 52.7, 18.6; **HRMS (ESI$^+$)** *m/z* calcd for $C_{20}H_{17}NNaO_5S^+$: 406.0720; $[M + Na]^+$ found: 406.0720.

Methyl 2-(2-(4-(hexylthio)phenoxy)propanoyl)thiazole-4-carboxylate (17j, GK449)

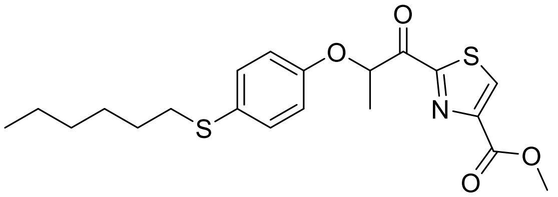

Yield 89% (362 mg); Yellow solid m.p.: 64-66 ºC; **^{1}H-NMR** (600 MHz, $CDCl_3$): δ 8.44 (s, 1H, ArH), 7.19 (d, J = 9.0 Hz, 2H, ArH), 6.77 (d, J = 9.0 Hz, 2H, ArH), 5.97 (q, J = 7.2 Hz, 1H, OCH), 3.93 (s, 3H, OCH_3), 2.72 (t, J = 7.2 Hz, 2H, SCH_2), 1.68 (d, J = 7.2 Hz, 3H, CH_3), 1.53-1.45 (m, 2H, CH_2), 1.35-1.26 (m, 2H, CH_2), 1.25-1.13 (m, 4H, 2 x CH_2), 0.80 (t, J = 6.6 Hz, 3H, CH_3); **^{13}C-NMR** (150 MHz, $CDCl_3$): δ 191.4, 164.7, 161.1, 156.2, 148.8, 133.9, 132.4, 128.5, 116.1, 75.0, 52.7, 35.4, 31.3, 29.3, 28.4, 22.5, 18.6, 14.0; **HRMS (ESI$^+$)** *m/z* calcd for $C_{20}H_{26}NO_4S_2^+$: 408.1298; $[M + H]^+$ found: 408.1291.

3-(4-(Heptyloxy)phenoxy)-2-hydroxypropanenitrile (18a)

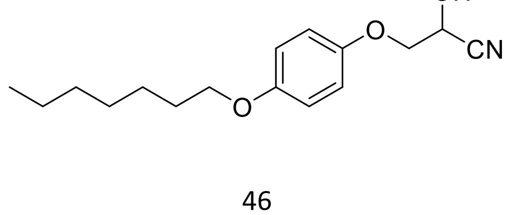

Yield 67% (186 mg); White solid; m.p.: 78-80 °C; **^{1}H-NMR** (400 MHz, $CDCl_3$): *δ* 6.98-6.70 (m, 4H, ArH), 4.83-4.68 (m, 1H, CHCN), 4.21-4.08 (m, 2H, OCH_2), 3.90 (t, *J* = 6.6 Hz, 2H, OCH_2), 3.75 (br s, 1H, OH), 1.82-1.70 (m, 2H, CH_2), 1.51-1.25 (m, 8H, 4 x CH_2), 0.90 (t, *J* = 6.5 Hz, 3H, CH_3); **^{13}C-NMR** (100 MHz, $CDCl_3$): *δ* 154.3, 151.5, 117.9, 116.0, 115.5, 69.6, 68.6, 60.5, 31.7, 29.2, 29.0, 25.9, 22.5, 14.0; **HRMS (ESI$^+$)** *m/z* calcd for $C_{16}H_{23}NNaO_3^+$: 300.1570; $[M + Na]^+$ found: 300.1569.

2-Hydroxy-3-(4-octylphenoxy)butanenitrile (18b)

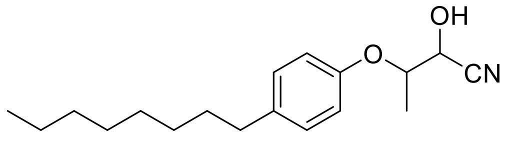

Yield 66% (191 mg); Colorless oil; **^{1}H-NMR** (400 MHz, $CDCl_3$): *δ* 7.11 (d, *J* = 8.2 Hz, 2H, ArH), 6.87 (d, *J* = 8.2 Hz, 2H, ArH), 4.61-4.47 (m, 2H, CHCN and OCH), 2.55 (t, *J* = 7.7 Hz, 2H, $PhCH_2$), 1.63 (br s, 1H, OH), 1.61-1.53 (m, 2H, CH_2), 1.44 (t, *J* = 6.9 Hz, 3H, CH_3), 1.35-1.24 (m, 10H, 5 x CH_2), 0.88 (t, *J* = 6.6 Hz, 3H, CH_3); **^{13}C-NMR** (100 MHz, $CDCl_3$): *δ* 154.5, 154.4, 137.2, 129.6, 117.3, 116.6, 116.5, 75.1, 74.8, 65.3, 65.0, 35.1, 31.9, 31.6, 29.4, 29.2, 22.7, 15.5, 15.3, 14.1; **HRMS (ESI$^+$)** *m/z* calcd for $C_{18}H_{27}NNaO_2^+$: 312.1934; $[M + Na]^+$ found: 312.1934.

3-(4-(Heptyloxy)phenoxy)-2-hydroxybutanenitrile (18c)

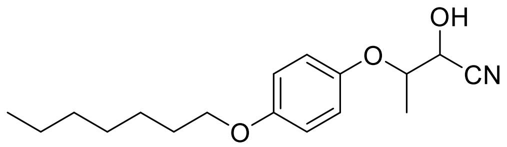

Yield 62% (181 mg); Colorless oil; **^{1}H-NMR** (400 MHz, $CDCl_3$): *δ* 6.89 (d, *J* = 9.1 Hz, 2H, ArH), 6.83 (d, *J* = 9.1 Hz, 2H, ArH), 4.58-4.51 (m, 1H, OCH), 4.46-4.36 (m, 1H, OC*H*), 3.91 (t, *J* = 6.6 Hz, 2H, OCH_2), 1.81-1.71 (m, 2H, CH_2), 1.50-1.26 (m, 12H, 4 x CH_2, CH_3 and OH), 0.90 (t, *J* = 6.6 Hz, 3H, CH_3); **^{13}C-NMR** (100 MHz, $CDCl_3$): *δ* 154.8, 150.3, 150.2, 118.4, 118.4, 115.5, 115.5, 76.1, 76.0, 68.6, 65.2, 64.8, 31.7, 29.3, 29.0, 26.0, 22.6, 15.3, 14.0; **HRMS (ESI$^+$)** *m/z* calcd for $C_{17}H_{25}NNaO_3^+$: 314.1727; $[M + Na]^+$ found: 314.1728.

2-Hydroxy-3-(4-phenoxyphenoxy)butanenitrile (18d)

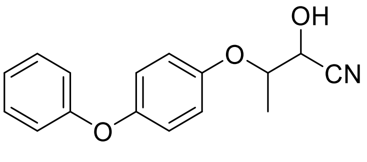

Yield 50% (135 mg); Colorless oil; **^{1}H-NMR** (400 MHz, $CDCl_3$): δ 7.30 (t, J = 6.9 Hz, 2H, ArH), 7.11 (t, J = 6.9 Hz, 1H, ArH), 7.01-6.80 (m, 6H, ArH), 4.68-4.39 (m, 2H, CHCN and OH), 4.25 (t, J = 6.1 Hz, 1H, OCH), 1.55-1.23 (m, 3H, CH_3); **^{13}C-NMR** (100 MHz, $CDCl_3$): δ 157.6, 152.5, 151.4, 129.5, 122.7, 120.4, 117.9, 117.8, 75.5, 75.0, 64.9, 64.3, 15.3, 15.0; **HRMS (ESI$^+$)** *m/z* calcd for $C_{16}H_{15}NNaO_3^+$: 292.0944; [M + Na]$^+$ found: 292.0948.

Cancer cell line screening

Cell proliferation assays

All cell lines were from the American Type Culture Collection (ATCC) Manassas, Virginia (US) and maintained under ATCC-recommended protocols and within 9 passages of the ATCC vial.

AVX420/GK420, AVX235 and AVX002 were stored as 31.6 µM solutions in 100% DMSO at -80°C until one hour before use. Cells exposed within one hour after thawing of the compound stocks. Final compound concentration range in the assay was 10 µM to 10 nM Reference compounds (list provided in Table S1) were stored as indicated by supplier. Compounds were dissolved in 100 % DMSO and stored at room temperature. Final concentration of the reference compounds in the assay was $3.16x10^{-5}$ M to $3.16x10^{-9}$ M. The final DMSO concentration during incubation was 0.4 % in all wells.

Cell lines were plated in Advanced RPMI 1640 (Gibco, ref 12633-012) cell culture medium, containing 1% Glutamax solution (Gibco, ref 35050-038), 0.4% Pen Strep solution (Gibco, ref 15140-122) and bovine serum. The $cPLA_2\alpha$ inhibitors were tested in 0.5% bovine serum (HyClone, ref SH30072.03) with the exception of769-P, A-204, BT-549, SR, T98G, which were tested in the presence of 1.0% serum and A-704 and LNCaP FGS, which were tested in the in the presence of 10% serum. Reference compounds were all tested in the presence of 10% bovine serum.

Cells were dispensed in a 384-well plate, at a concentration of 200 - 3200 cells per well, depending on the cell line, in 45 µL medium. Plated cells were incubated in a humidified atmosphere of 5% CO_2 at 37 ºC. After 24 hours, 5 µL of compound dilution was added and plates were further incubated for another 72 hours. After 72 hours, 25 µL of ATPlite 1Step™ (PerkinElmer) solution was added to each well, and subsequently shaken for 2 minutes. After 10 minutes of incubation in the dark, the luminescence was recorded on an Envision multimode reader (PerkinElmer).

Cell growth control. The cellular doubling times of all cell lines are calculated from the t = 0 hours and t = 72 hours growth signals of the untreated cells. If the doubling time is out of specification (0.5 – 2.0 times deviating from historic average) the assay is invalidated.

IC_{50}s were calculated by non-linear regression using IDBS XLfit 5. The percentage growth after 72h (*%-growth*) was calculated as follows: 100% x ($luminescence_{t = 72h}$ / $luminescence_{untreated,t=72h}$). This was fitted to the ^{10}log compound concentration (*conc*) by a 4-parameter logistics curve: *%-growth* = *bottom* + (*top* – *bottom*) / (1+ $10^{(logIC50 - conc)*hill)}$), where *hill* is the Hill-coefficient, and *bottom* and *top* the asymptotic minimum and maximum cell growth that the compound allows in that assay.

All analysis were performed in the statistical software package R and visualized using GraphPad Prism version 10.0.0

Compound activity profiling

Correlations were calculated by the Pearson methods where the $^{10}\log IC_{50}$ (nM) data of the cPLA$_2\alpha$ inhibitors were used to calculate correlations with all reference compounds. The significance of correlation coefficients was estimated by a p-value based on converting the Pearson correlation to a t-statistic according to equation (1) where *n* indicates the number of cell lines. Values for *t* were subsequently translated into p-values using the Student t distribution function.

$$t = \frac{r.\sqrt{n-2}}{\sqrt{1-r^2}} \qquad (1)$$

*Hierarchical clustering***.** Compounds were clustered using unsupervised hierarchical clustering. The Ward-method was used, distances were set to 1 - corr(*a,b*), where corr(*a,b*) is the Pearson correlation between the $^{10}\log IC_{50}$ (nM) profiles of compounds *a* and *b*, defined by the covariance of datasets *a* and *b*, and the standard deviations (σ) of datasets *a* and *b* (equation 2).

$$\mathrm{corr}(a,b) = \frac{cov(a,b)}{\sigma_a \sigma_b} \qquad (2)$$

The resulting clustering tree was validated using bootstrapping, as implemented in the R-package *pvclust*. This analysis provides data on reliability of clustering by re-clustering subsamples of the data. It reports the values AU (approximate unbiased) and BP (bootstrapping probability). The quantity 1 - AU gives an approximate p-value for cluster reliability. Clusters associated with AU > 95 ($p < 0.05$) were considered validated clusters.

The correlation matrix shows all correlations between all compounds analyzed. Pearson correlations corr(*a,b*) were calculated for all compound pairs, and displayed using the R-package *corrplot*. Compounds in rows and columns were ordered according to the results of the hierarchical clustering.

The network tree connects compounds with correlations exceeding 0.5, thus providing a visual interpretation of compound similarities. In contrast to the clustering tree, compounds can have multiple neighbors. Data were analyzed and plotted in R, using the *igraph* package.

Identification of genetic modifications associated with inhibitor sensitivity.
The mutation status of cell lines was established from a combination of public and proprietary data. Based on public data (COSMIC Cancer Genome Project), genetic changes were required to be observed at least once in one patient tumor sample in COSMIC. Cell lines were classified as having a 'wild type' or a 'mutated' genotype, where 'mutated' means: at least one allele changed by point mutation, insertion, deletion, amplification or copy number variation. Analysis was performed on genes that were mutated in at least three different cell lines (114 in total).

The $^{10}\log IC_{50}$ differences between the 'mutated' and 'wild type' groups were analyzed by a two-sided homoscedastic t-test in R. To compute significance, p-values were subjected to a Benjamin-Hochberg multiple testing correction, and genetic associations with a <20% false discovery rate (FDR) were considered significant. (adj. p. <0.2).

Correlation of gene expression with drug response.
Gene expression data as reported by CCLE2 (CCLE_Expression_ Entrez_2012-10-18.res) were found for 55 of the 66 cell lines. Data from cell lines A-704 and LNCaP FGS were not used because they did not proliferate in low serum. Data for 18900 genes were used to investigate whether high expression of a certain gene is correlated to either sensitivity or resistance to inhibitor.

The Pearson correlations between the $^{10}\log IC_{50}$ (nM) values of the inhibitors in the cell lines, and gene expression values in the corresponding cell lines were calculated and filtered based on specific gene subsets to avoid false-positive correlations. The first was 361 genes experimentally determined to be frequently mutated, amplified or deleted in cancer (Cancer genes) [5-7] the second was 87 phospholipase genes from the drug gene interaction database (DGIdb)[8] and the third was 405 genes that are involved in drug resistance based on expert- and non-expert curated databases[8] (drug resistance genes). Pearson p-values were subjected to multiple testing correction using the Benjamini-Hochberg correction. Pearson correlations with a false discovery rate <20% were considered significant.

Genes which are often correlated to response, irrespective of which compound is used ('frequent hitters') were filtered out in a second approach, using the reference library of 120 compounds. First, Pearson correlations were calculated between each of these compounds and expression of the 18,900 genes. After including the correlations of the inhibitor, all correlations were subjected to a Fisher's z-transformation. This way, the correlation data assume a normal distribution, which is necessary for further analysis. Subsequently, the average Fisher's z (μ) and standard deviation (σ) were calculated for every gene across all compounds. A sigma score was then assigned to every Fisher's z value (X), according to equation (1).

$Sigma\ score = X - \mu\sigma$ (1)

The sigma score indicates the number of standard deviations a particular gene-compound correlation stands out above or below the mean of the compound library, that is, how unique its association is compared to other compounds. The significance of the sigma score was estimated by a p-value as determined in R and a p-value < 0.001. was considered significant.

Gene set enrichment and overlap analyses.
Gene set enrichment analysis was performed in R using the Piano package10 using the pre-ranked list of 18,900 correlations as input. Gene sets mainly enriched in sensitivity markers get a highly positive ES assigned. Lists were queried against the Oncogenic Signatures collection (c6) in the Molecular Signatures Database (MSigDB) which had 189 gene sets representing signatures of cellular pathways which are often deregulated in cancer, and which are directly derived from microarray experiments. Only gene sets containing a minimum number of 10, and a maximum number of 1000 genes were used in the analysis. The significance of enrichment of a specific gene set was determined by performing a permutation test with a total of 10000 permutations. This significance indicates the probability that a random list of genes shows a higher enrichment than the provided list of genes. P-values were subjected to multiple testing

correction using the Benjamini-Hochberg correction to control the rate of false-positives. Gene sets with false discovery rate <20% were considered significant.

Gene set overlaps with the MSigDB human collections were calculated using the online toolset (https://www.gsea-msigdb.org/) available via the Broad institute, UC San Diego. Gene sets with and FDR q value < 0.05 were considered significant and the top ten overlapping gene sets are shown for each analysis.

RNA-Seq and ribosome-associated RNA(Ribo)-Seq

RNA-Seq and ribosome-associated RNA(Ribo)-Seq was performed by Eclipsebio (eclipsebio.com). The cytosolic fraction of crosslinked cell pellets was isolated and RNA quality and quantity was assessed before 250 ng of the isolated RNA was used to build 3' RNA-Seq libraries. 10 µg RNA was used for immunoprecipitation (IP) for Ribo-SEQ analysis. Non-crosslinked RNA was removed from the IP through multiple wash steps and the remaining RNA was released via proteinase digestion. RNA from both sources was fragmented and libraries were generated from the poly(A) tails. Libraries were sequenced on a NextSeq 2000. After sequencing, samples were processed with Eclipsebio's proprietary analysis pipeline (v1). Unique molecular identifiers (UMIs) were pruned from read sequences using umitools (v1.1.1). Next 3' adapters were trimmed from reads using cutadapt (v3.2). Reads were then mapped to a custom, curated database of repetitive elements and rRNA sequences. All non-repeat mapped reads were mapped to the genome hg38 using STAR (v2.7.7a) and PCR duplicates were removed using umi_tools (v1.1.1). Gene coverage was calculated using Eclipsebio's proprietary feature counting algorithm and ribosome occupancy was calculated by dividing coverage in the ribosome-associated (IP) library by the RNA-Seq library. Differentially expressed and occupied genes were identified using DESeq2. Significant differential expression was defined using a cutoff on p.value < 0.01 (after multiple testing adjustment, "padj" metric) and requiring the fold change in gene coverage to be greater than 1 or less than -1 ($\log_2$ scale). Significance of the ribosomal occupancy (RO) difference was determined by extending DESeq2 models to include RO fold change as an interaction between the experimental condition and the library type. Genes with a RO log2 (fold change) > 1 or <-1 and an adjusted p-value < 0.01 were considered significant. Only genes annotated as protein-coding or lncRNA are included.

Volcano plots were produced using plotyl.js (v2.12.2). GO and KEGG analyses were performed with clusterProfiler's enrichGO and enrichKEGG functions available through the Bioconductor package clusterProfiler. For each term, the adjusted p-value for the enrichment analysis and the average fold change of all genes included are calculated and shown in the relevant tables.

HPLC chromatograms of inhibitors

GK401 (**17a**)

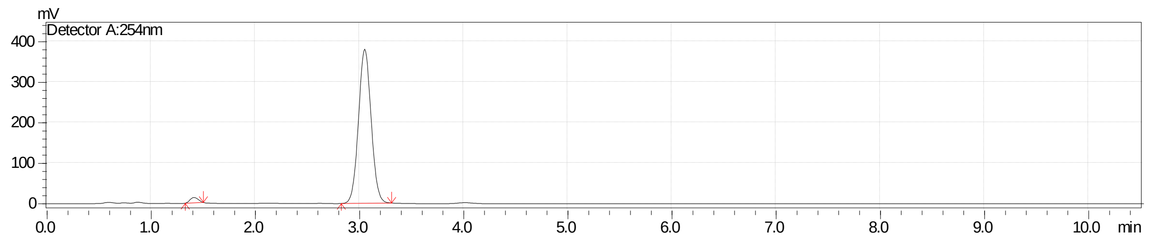

Peak#	Ret. Time	Area	Peak Start	Peak End	Area%
1	1.406	73061	1.325	1.500	2.2
2	3.045	3213349	2.825	3.308	97.8

Figure S9. HPLC chromatogram of inhibitor **17a** [ACN/water 80:20, 1 mL/min, column: Phenomenex, Luna 5 µm C18(2) 100 Å 150 x 2 mm, 254 nm].

GK420 (**17b**)

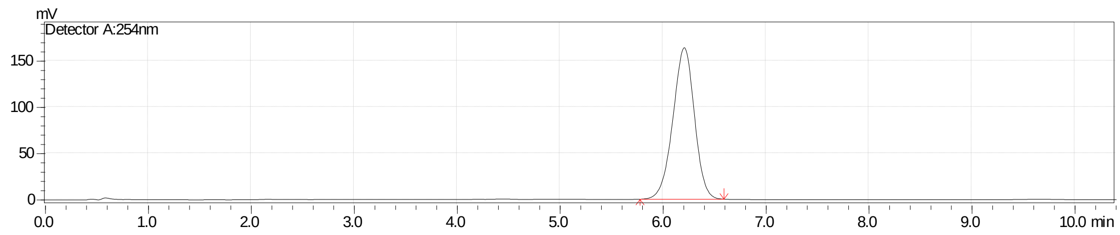

Peak#	Ret. Time	Area	Peak Start	Peak End	Area%
1	6.201	2388555	5.775	6.592	100.0

Figure S10. HPLC chromatogram of inhibitor 17b [ACN/water 60:40, 1 mL/min, column: Phenomenex, Luna 5 µm C18(2) 100 Å 150 x 2 mm, 254 nm].

GK403 (**17c**)

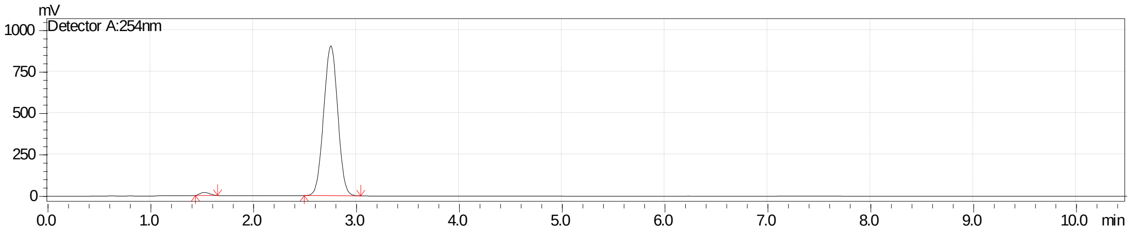

Peak#	Ret. Time	Area	Peak Start	Peak End	Area%
1	1.517	123769	1.433	1.650	1.4
2	2.746	8613241	2.492	3.042	98.6

Figure S11. HPLC chromatogram of inhibitor 17c [ACN/water 55:45, 1 mL/min, column: Phenomenex, Luna 5 µm C18(2) 100 Å 150 x 2 mm, 254 nm].

GK440 (**17d**)

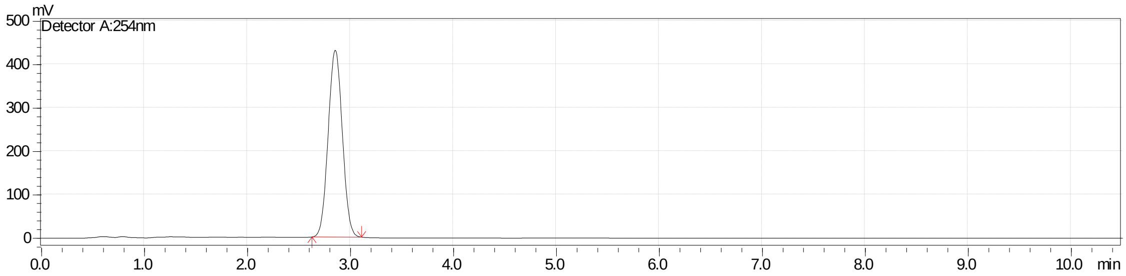

Peak#	Ret. Time	Area	Peak Start	Peak End	Area%
1	2.847	4121723	2.625	3.108	100.0

Figure S12. HPLC chromatogram of inhibitor **17d** [ACN/water 55:45, 1 mL/min, column: Phenomenex, Luna 5 µm C18(2) 100 Å 150 x 2 mm, 254 nm].

GK402 (**17g**)

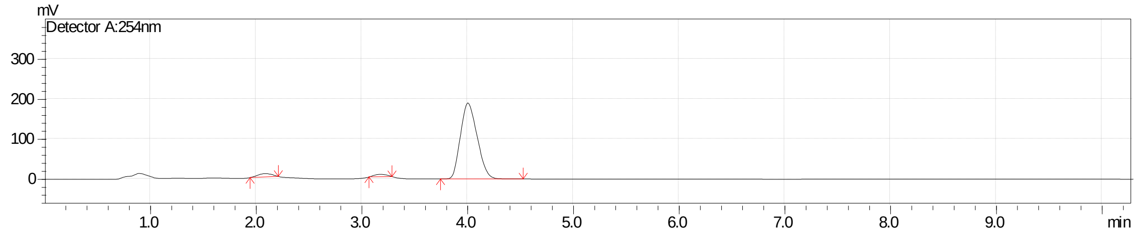

Peak#	Ret. Time	Area	Peak Start	Peak End	Area%
1	2.081	80024	1.942	2.208	3.6
2	3.170	50875	3.067	3.283	2.3
3	3.997	2094418	3.742	4.525	94.1

Figure S13. HPLC chromatogram of inhibitor **17g** [ACN/water 75:25, 1 mL/min, column: Phenomenex, Luna 5 µm C18(2) 100 Å 150 x 2 mm, 254 nm].

GK439 (**17h**)

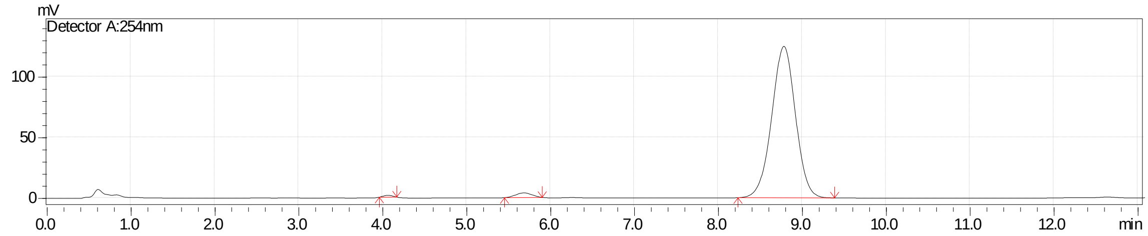

Peak#	Ret. Time	Area	Peak Start	Peak End	Area%
1	4.057	11113	3.958	4.167	0.4
2	5.676	50978	5.450	5.900	2.0
3	8.775	2466346	8.233	9.383	97.6

Figure S14. HPLC chromatogram of inhibitor **17h** [ACN/water 60:40, 1 mL/min, column: Phenomenex, Luna 5 µm C18(2) 100 Å 150 x 2 mm, 254 nm].

GK428 (**17i**)

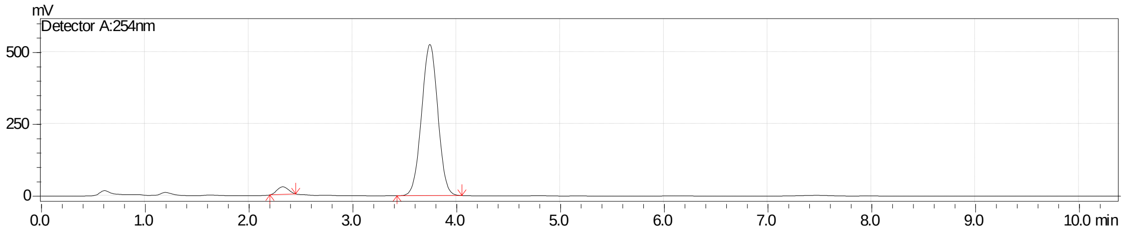

Peak#	Ret. Time	Area	Peak Start	Peak End	Area%
1	2.320	204649	2.200	2.450	3.5
2	3.737	5726156	3.425	4.050	96.5

Figure S15. HPLC chromatogram of inhibitor **17i** [ACN/water 55:45, 1 mL/min, column: Phenomenex, Luna 5 µm C18(2) 100 Å 150 x 2 mm, 254 nm].

^{1}H and ^{13}C NMR Traces

Figure S16. 11b

a. ^{1}H-NMR (400 MHz) of 11b in $CDCl_3$

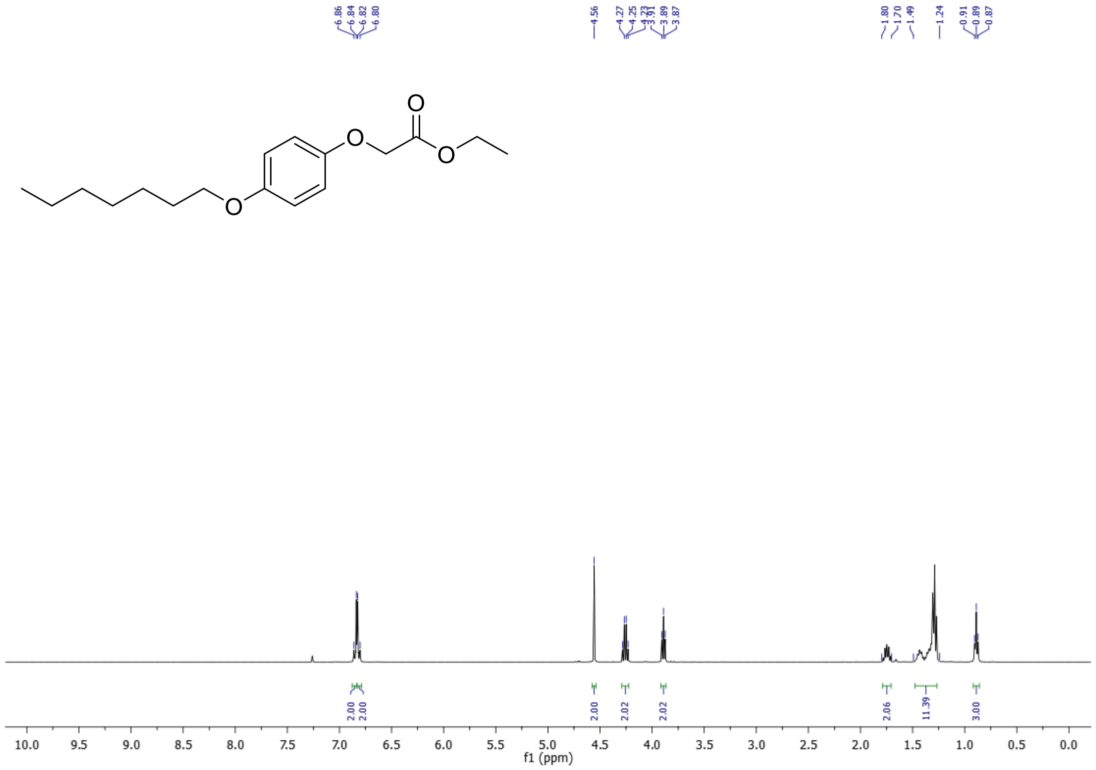

b.^{13}C-NMR (100 MHz) of 11b in $CDCl_3$

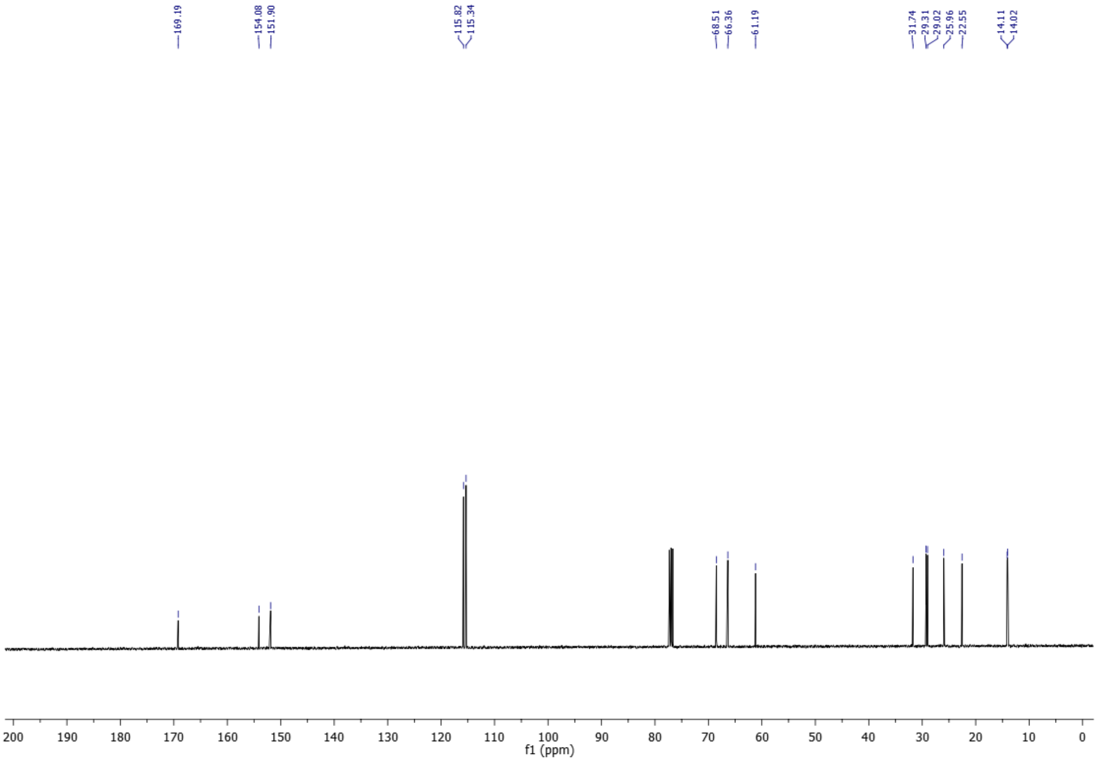

Figure S17. 11d

a. ^{1}H-NMR (400 MHz) of 11d in $CDCl_3$

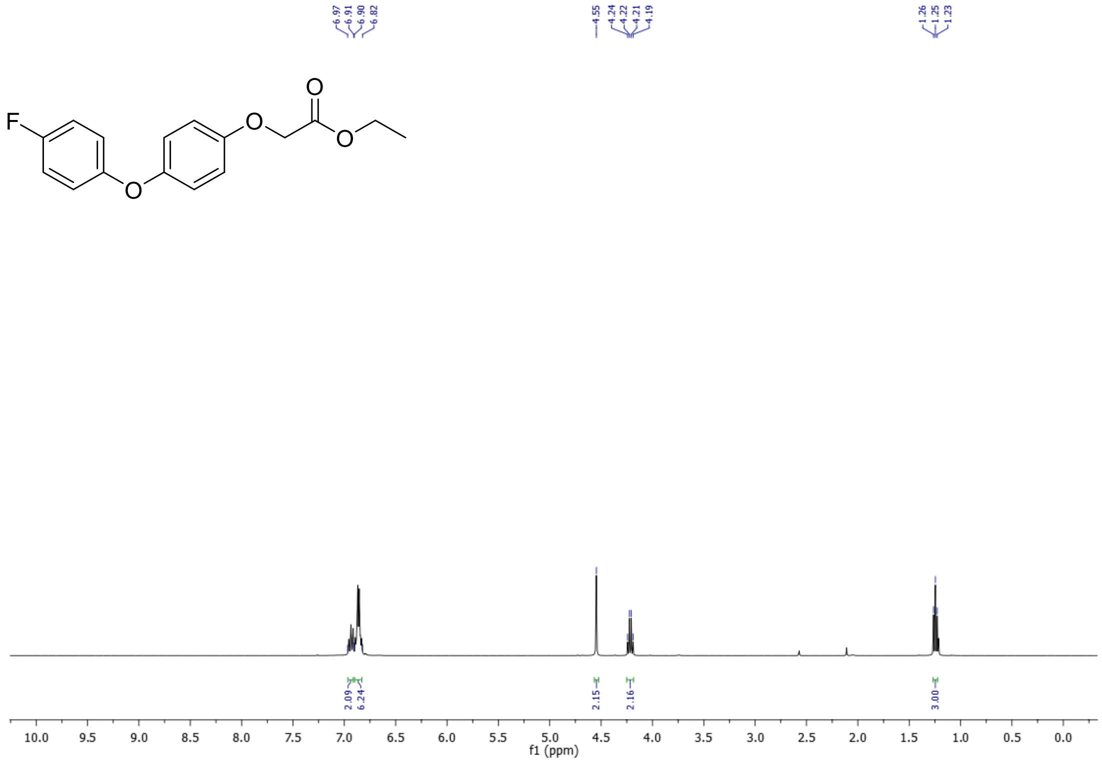

b. ^{13}C-NMR (100 MHz) of 11d in $CDCl_3$

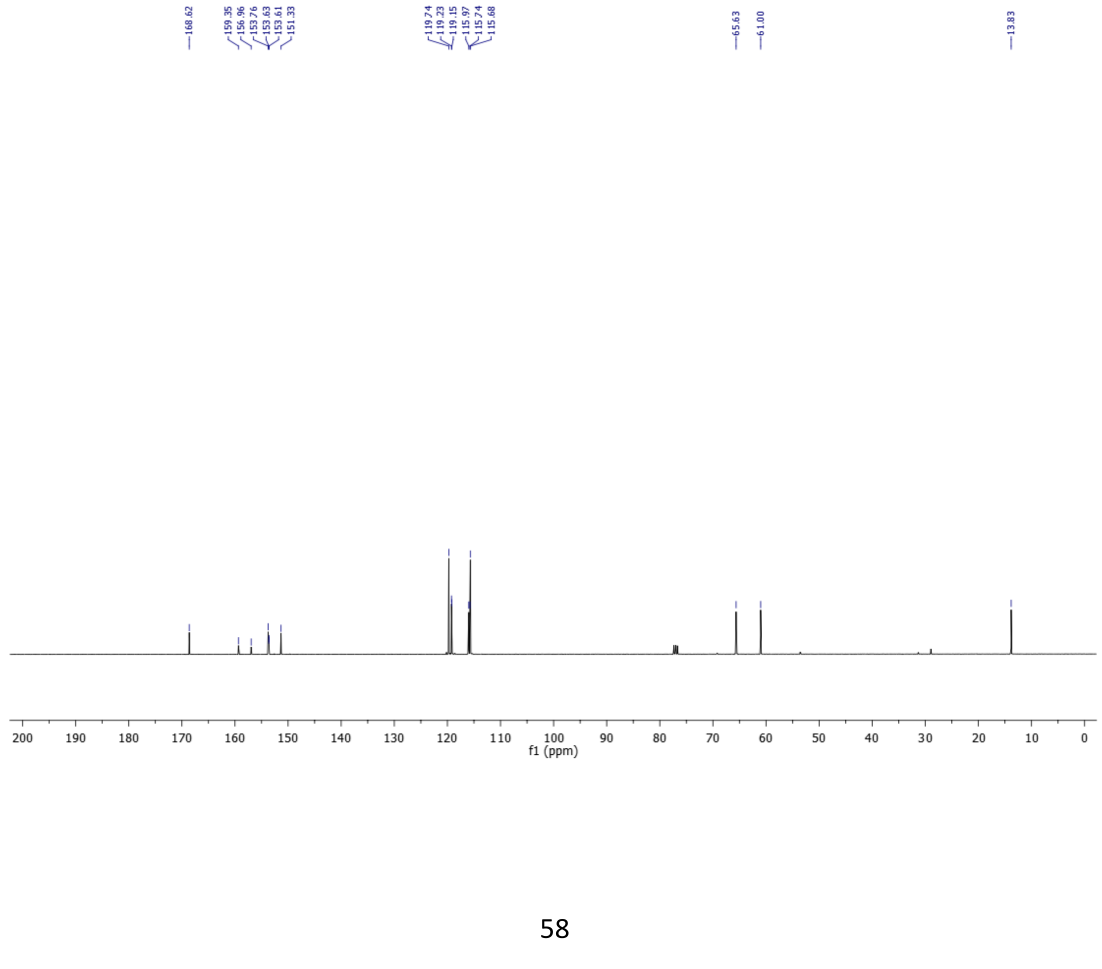

c. ^{19}F-NMR (377 MHz) of 11d in $CDCl_3$

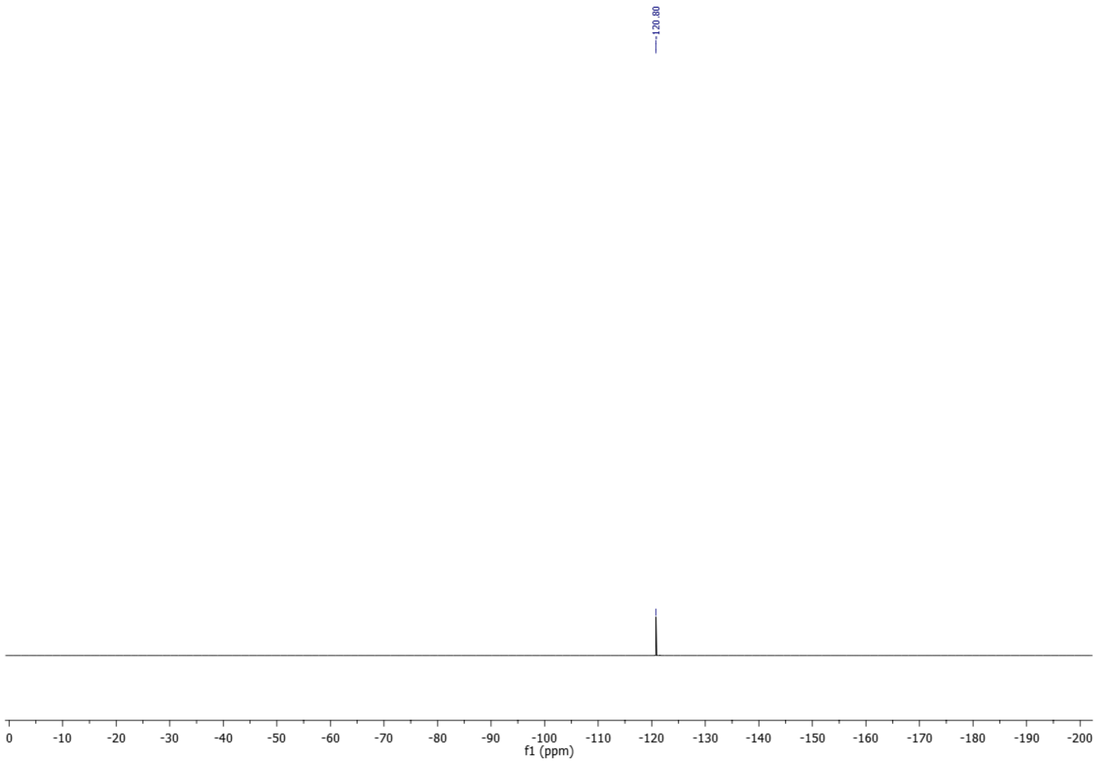

Figure S18. 11e

a. ^{1}H-NMR (200 MHz) of 11e in $CDCl_3$

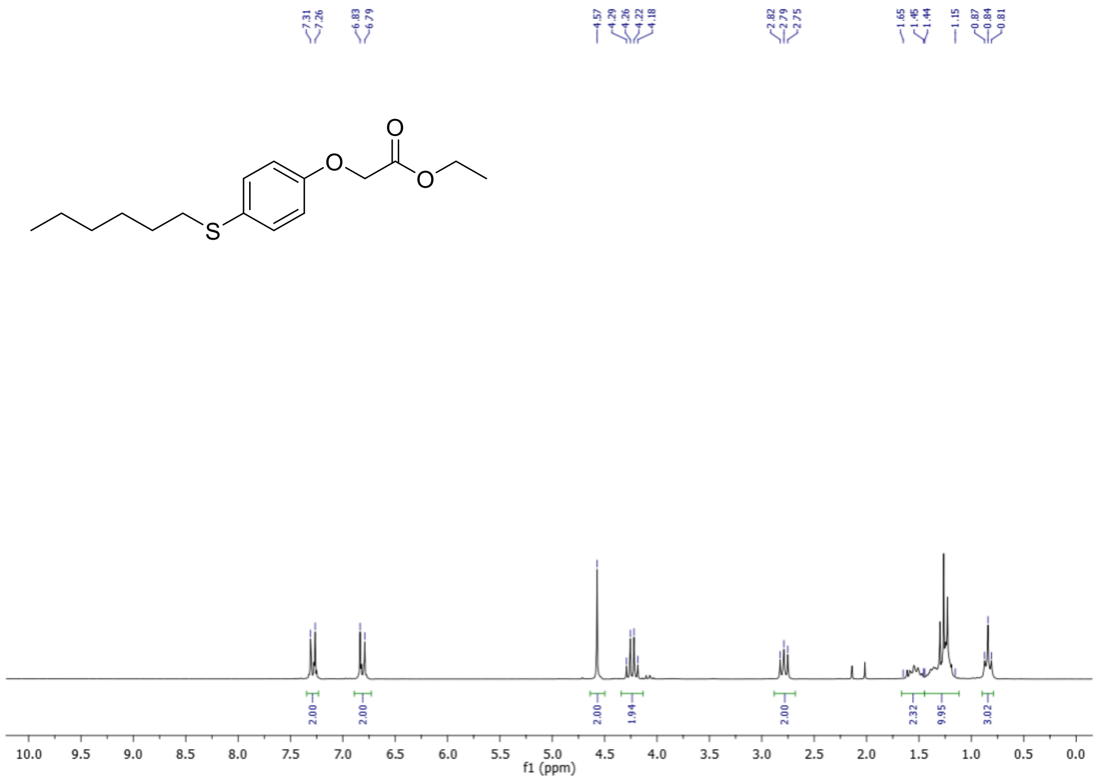

b. ^{13}C-NMR (50 MHz) of 11e in $CDCl_3$

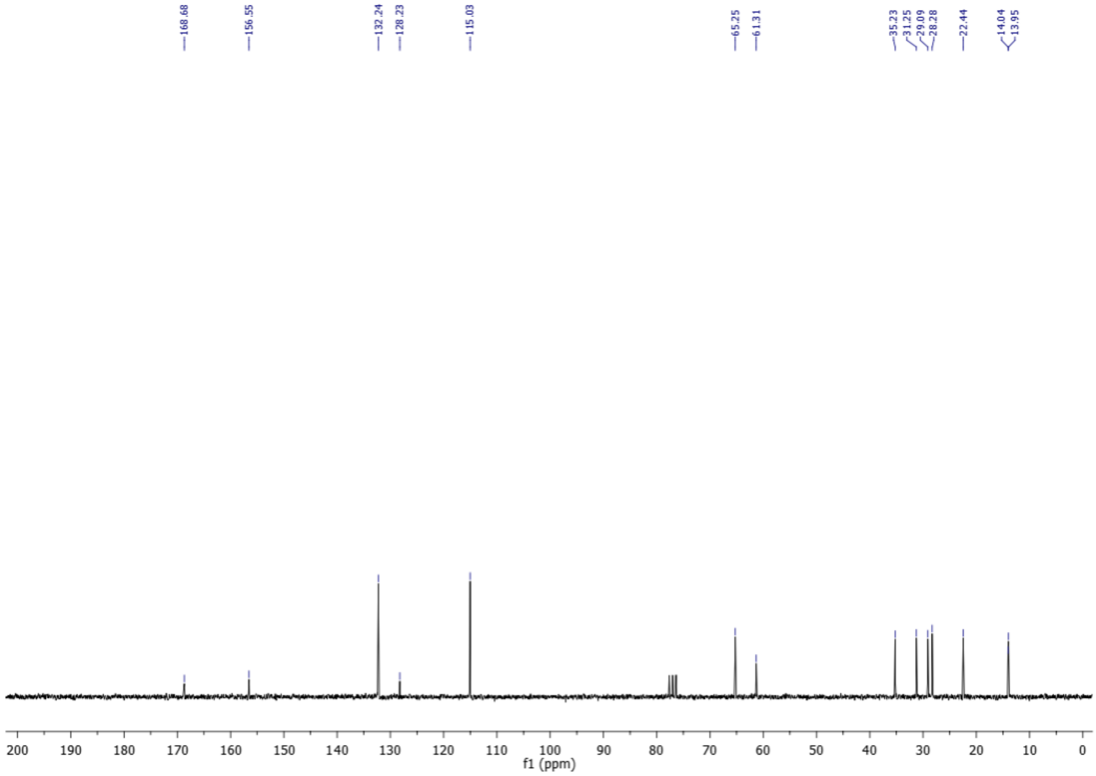

Figure S19. 11f

a. ^{1}H-NMR (400 MHz) of 11f in $CDCl_3$

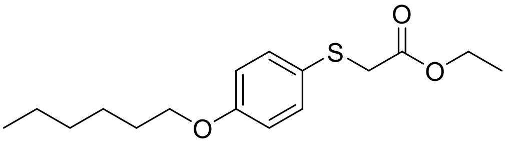

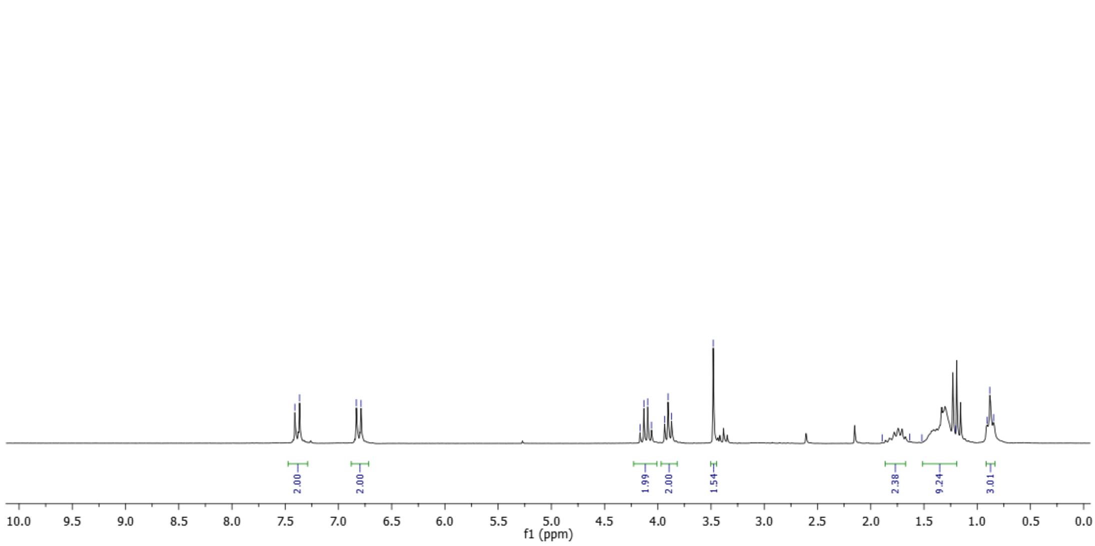

b. ^{13}C-NMR (100 MHz) of 11f in $CDCl_3$

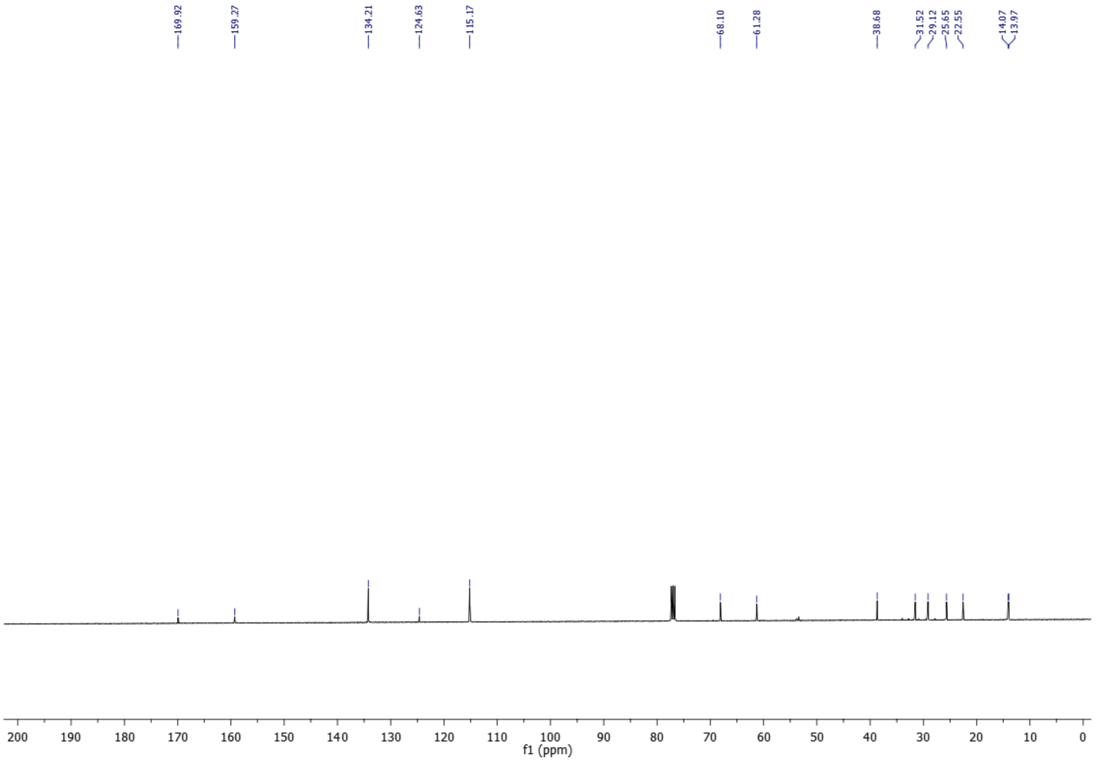

Figure S20. 11g

a. ^{1}H-NMR (400 MHz) of 11g in $CDCl_3$

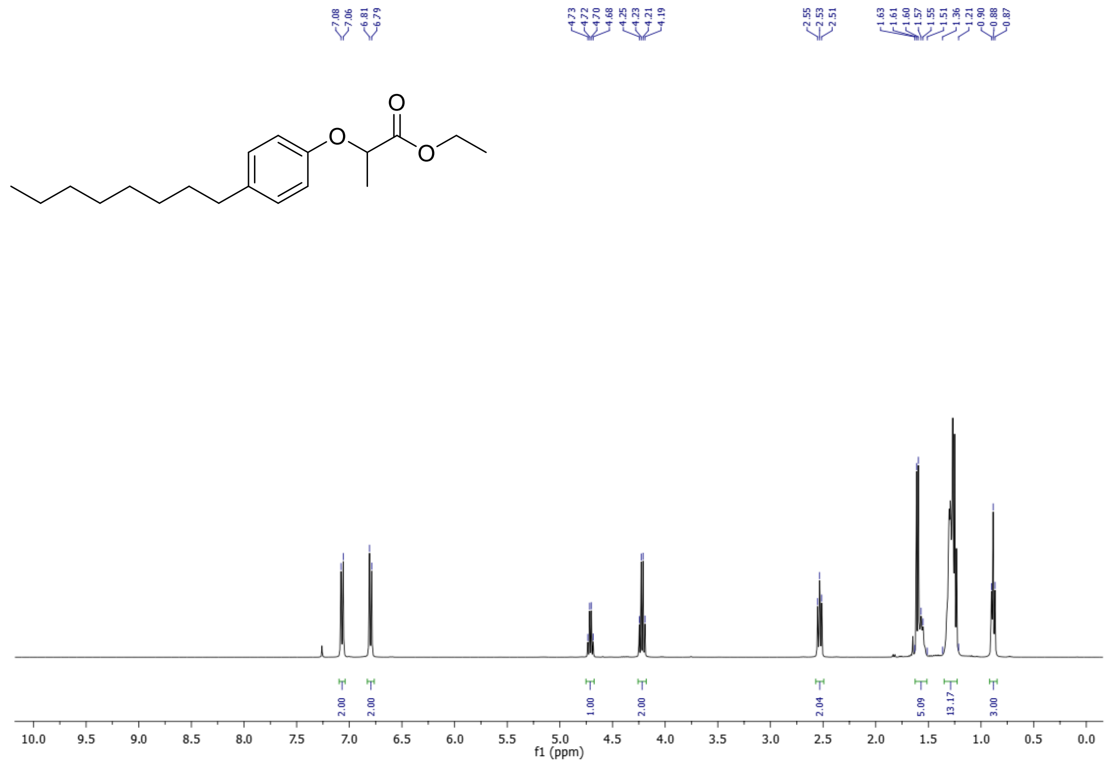

b. ^{13}C-NMR (100 MHz) of 11g in $CDCl_3$

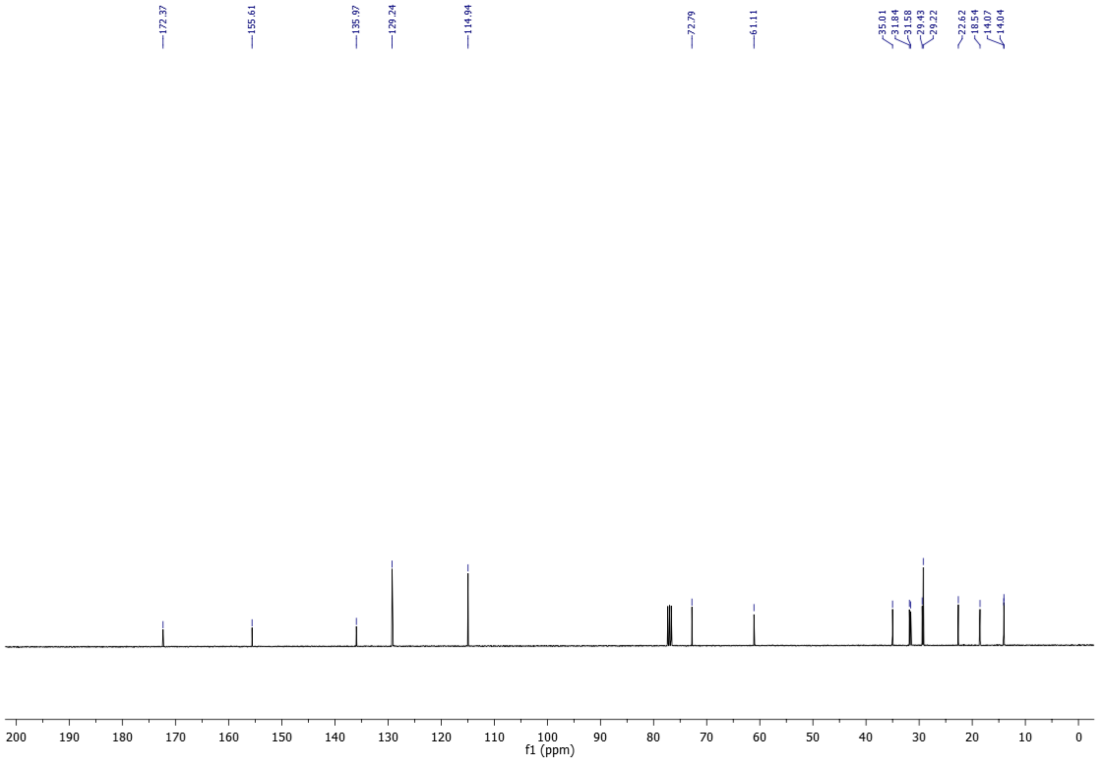

Figure S21. 11h

a. ^{1}H-NMR (200 MHz) of 11h in $CDCl_3$

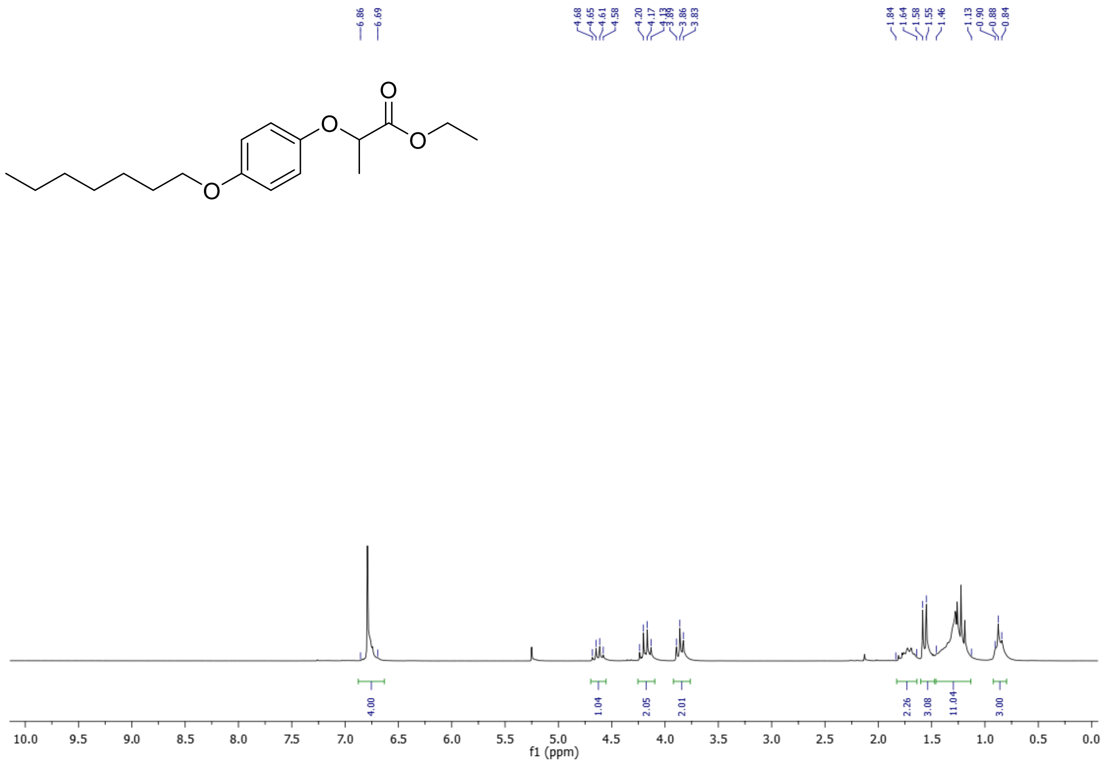

b. ^{13}C-NMR (50 MHz) of 11h in $CDCl_3$

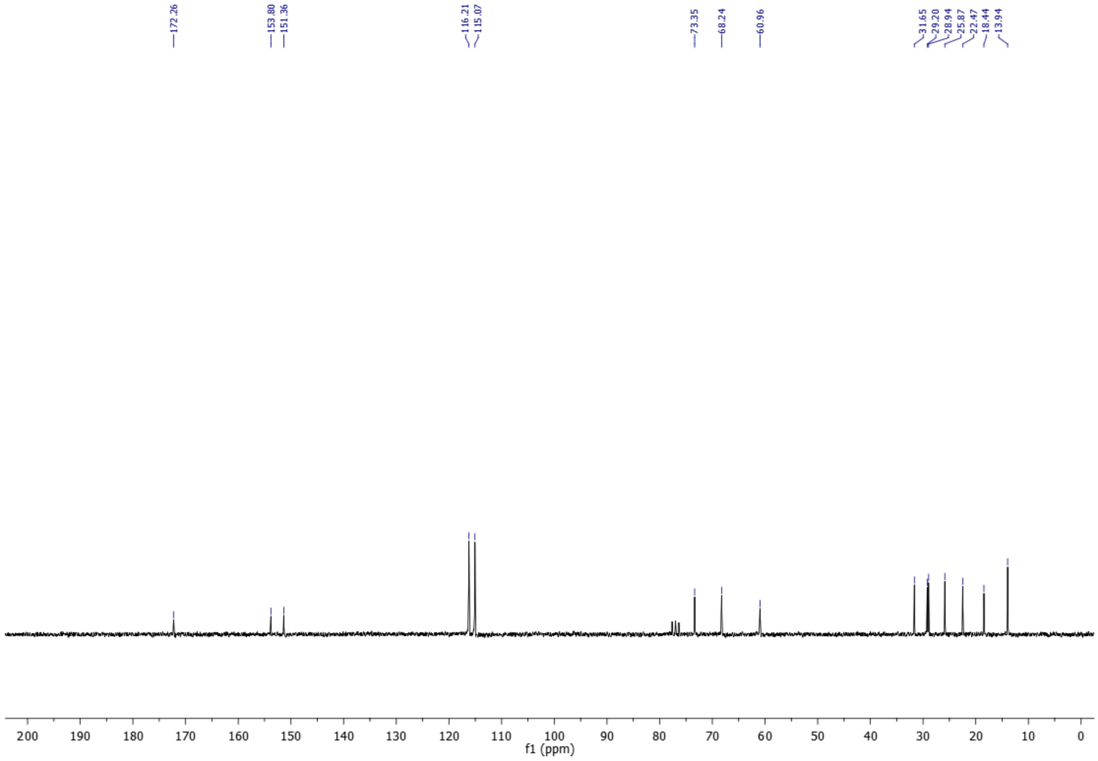

Figure S22. 11i

a. ^{1}H-NMR (400 MHz) of 11i in $CDCl_3$

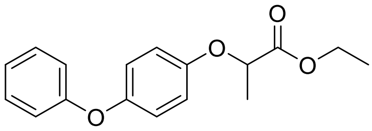

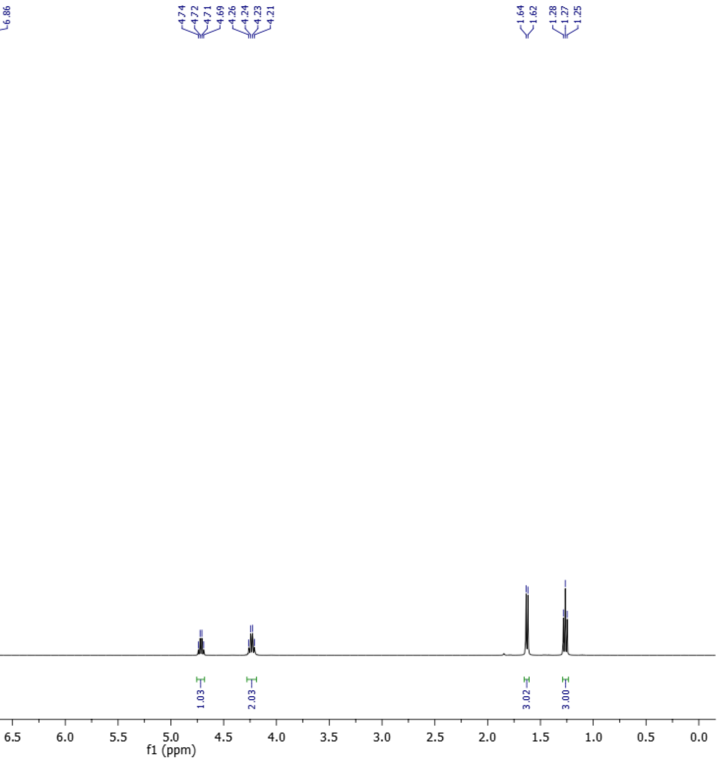

b. ^{13}C-NMR (100 MHz) of 11i in $CDCl_3$

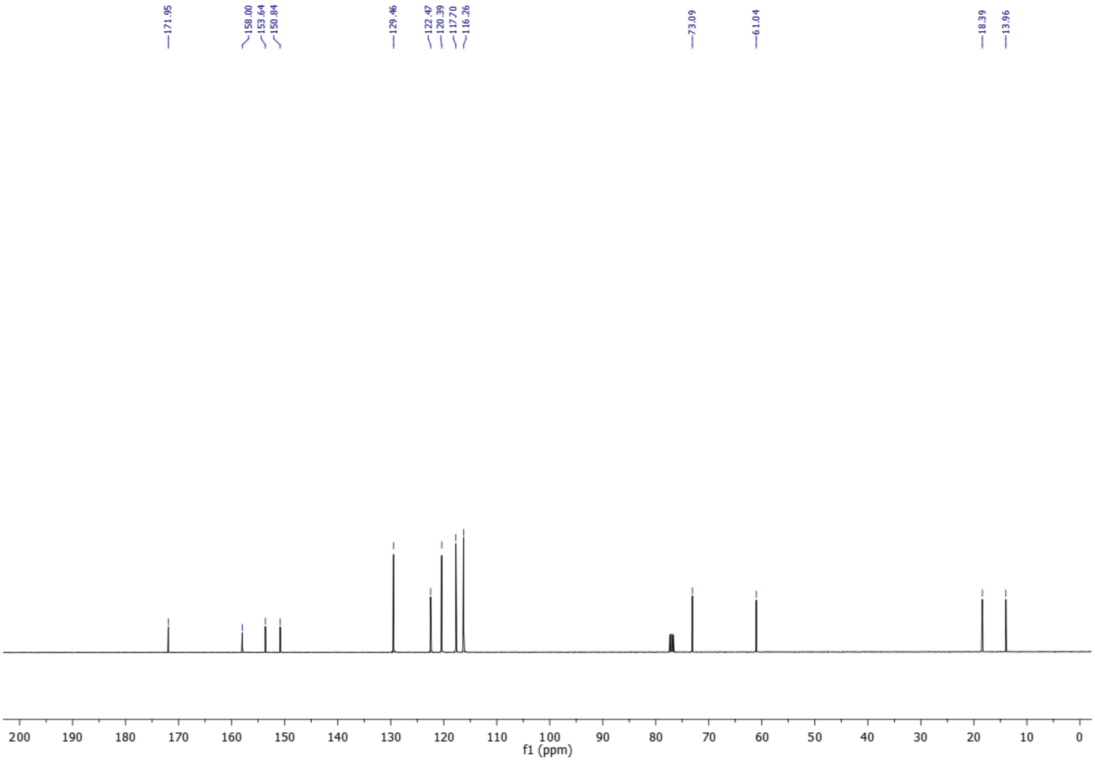

Figure S23. 11j

a. ^{1}H-NMR (200 MHz) of 11j in $CDCl_3$

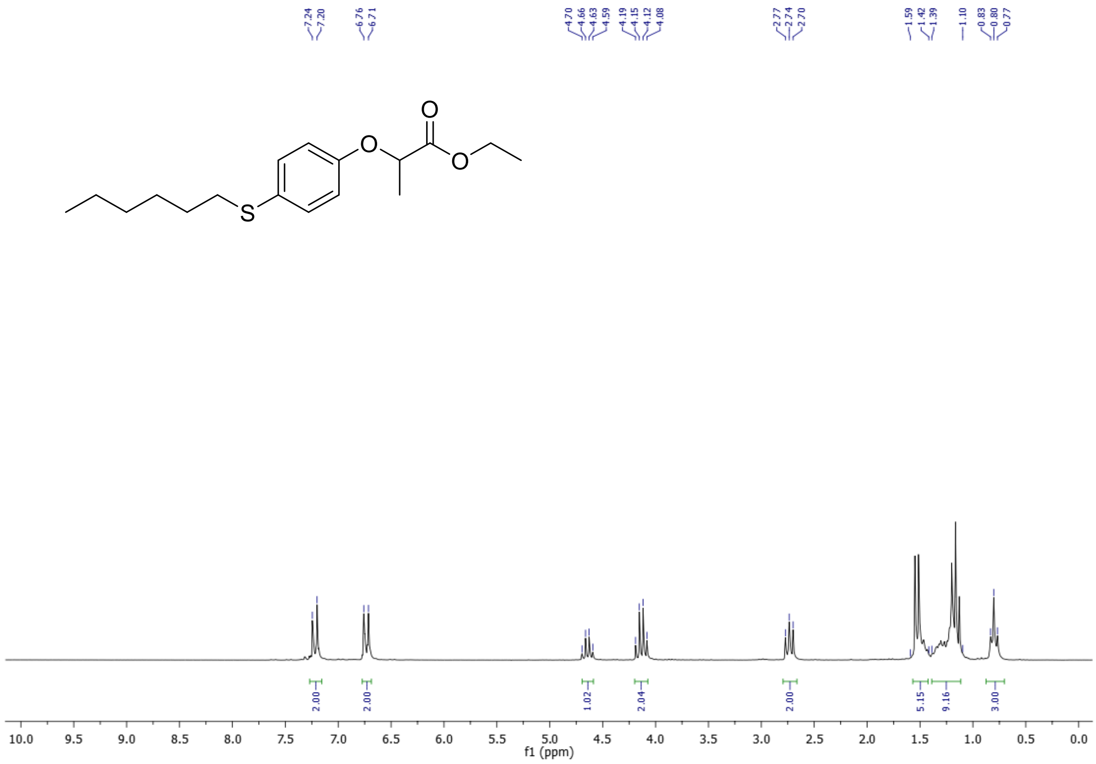

b. ^{13}C-NMR (50 MHz) of 11j in $CDCl_3$

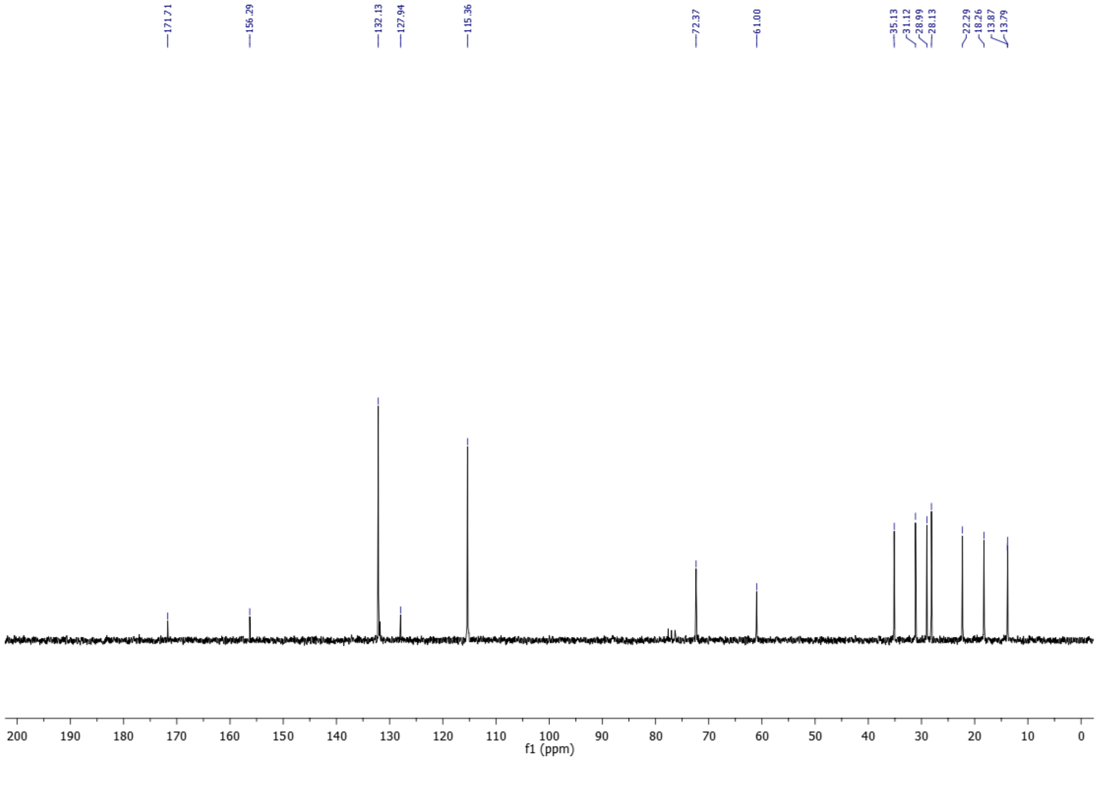

Figure S24. 11b

a. ^{1}H-NMR (400 MHz) of 12b in $CDCl_3$

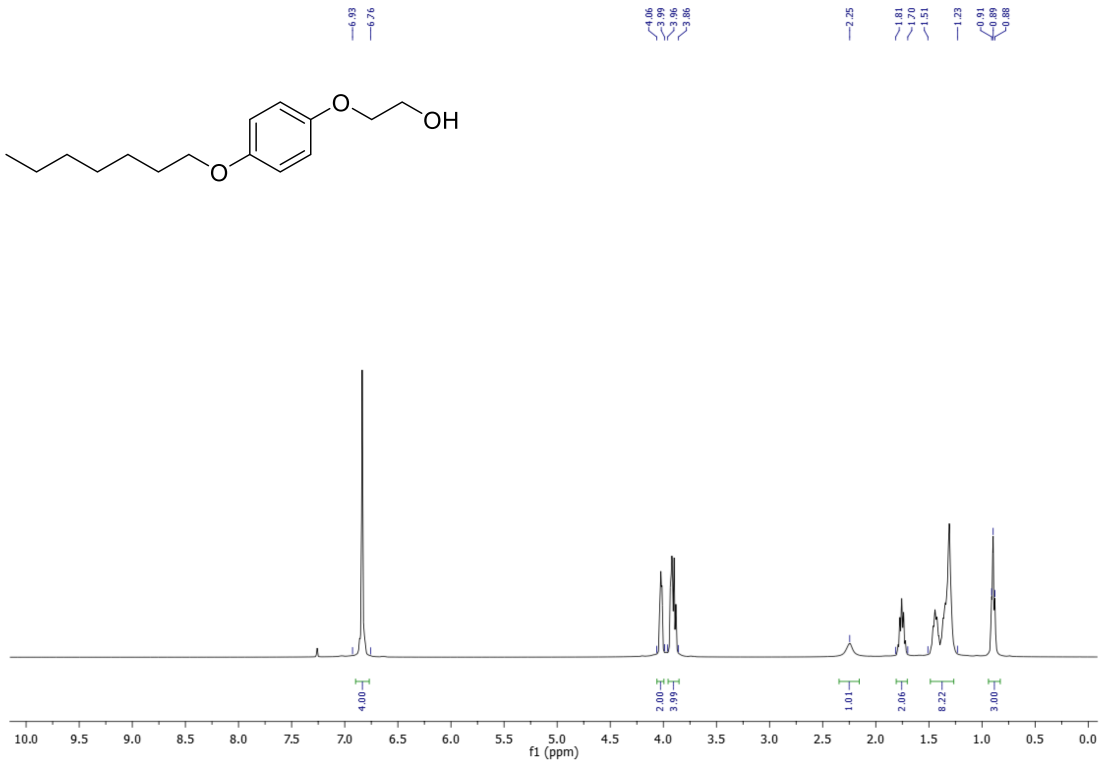

b. ^{13}C-NMR (100 MHz) of 12b in $CDCl_3$

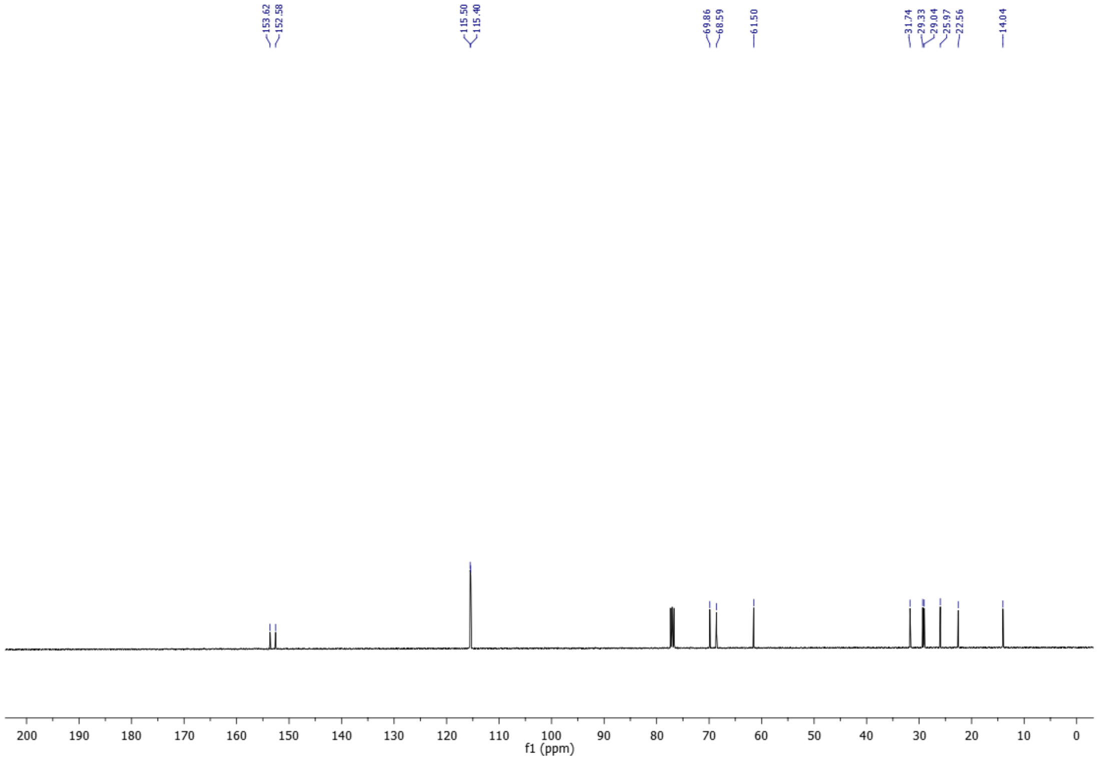

Figure S25. 12d

a. ^{1}H-NMR (200 MHz) of 12d in $CDCl_3$

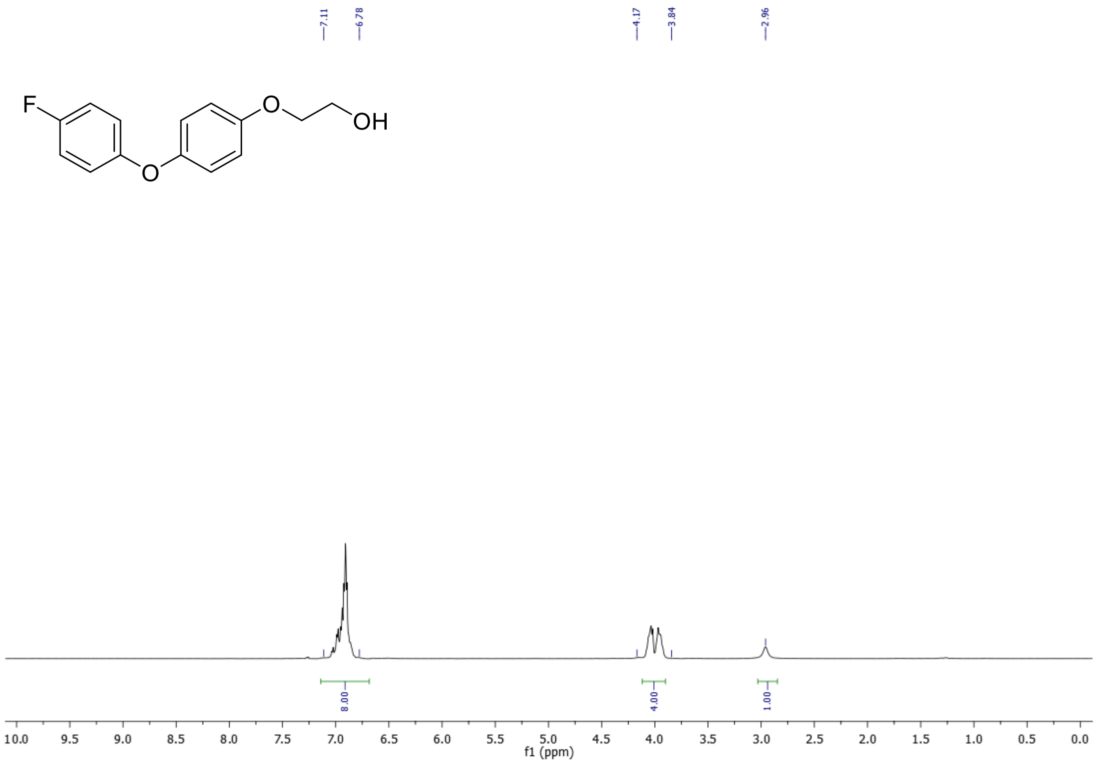

b. ^{13}C-NMR (50 MHz) of 12d in $CDCl_3$

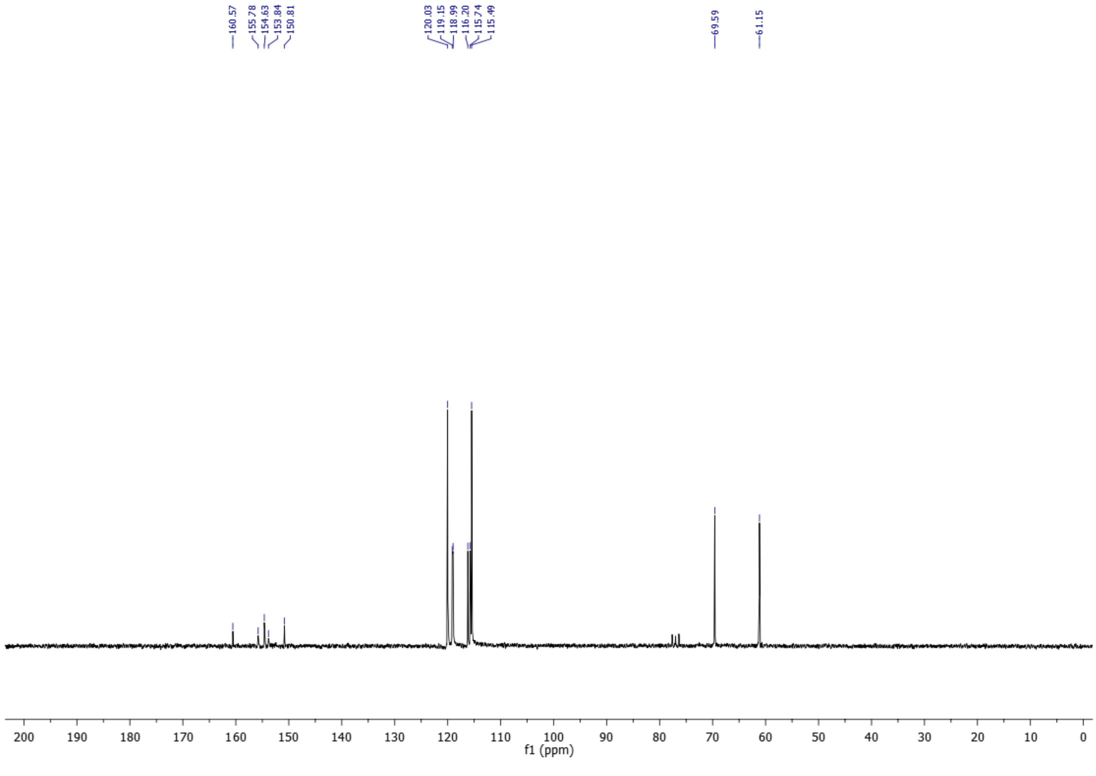

c. ^{19}F-NMR (177 MHz) of 12d in $CDCl_3$

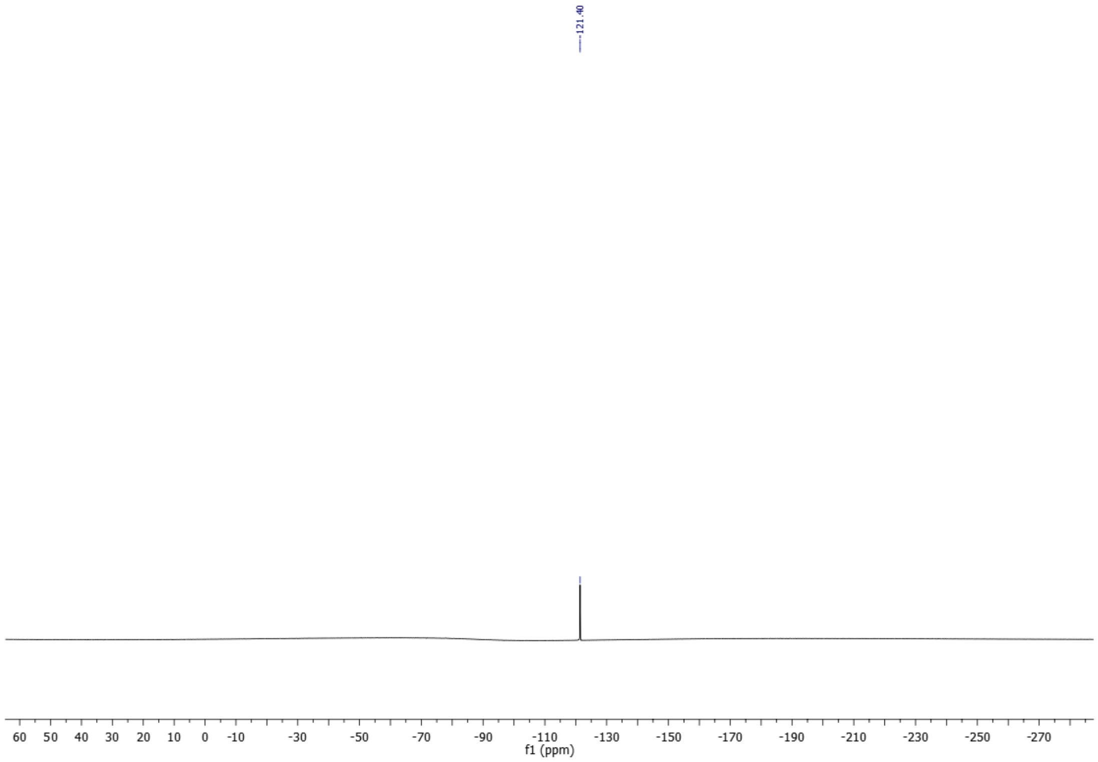

Figure S26. 12e

a. ^{1}H-NMR (200 MHz) of 12e in $CDCl_3$

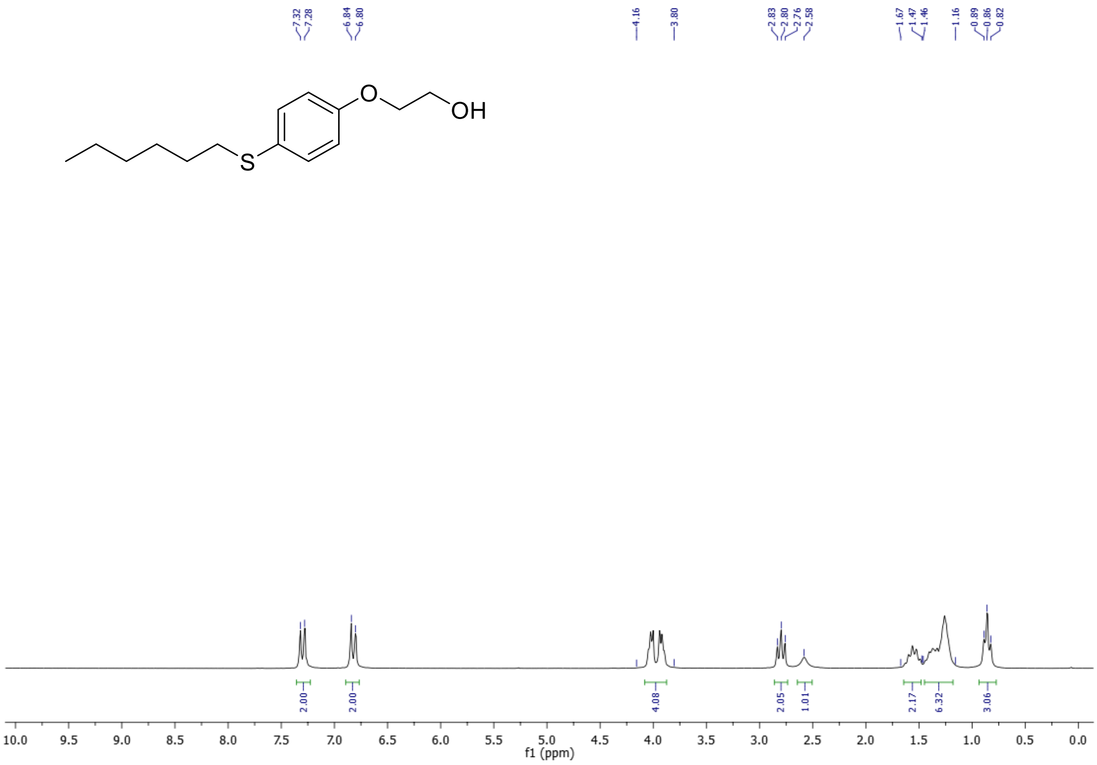

b. ^{13}C-NMR (50 MHz) of 12e in $CDCl_3$

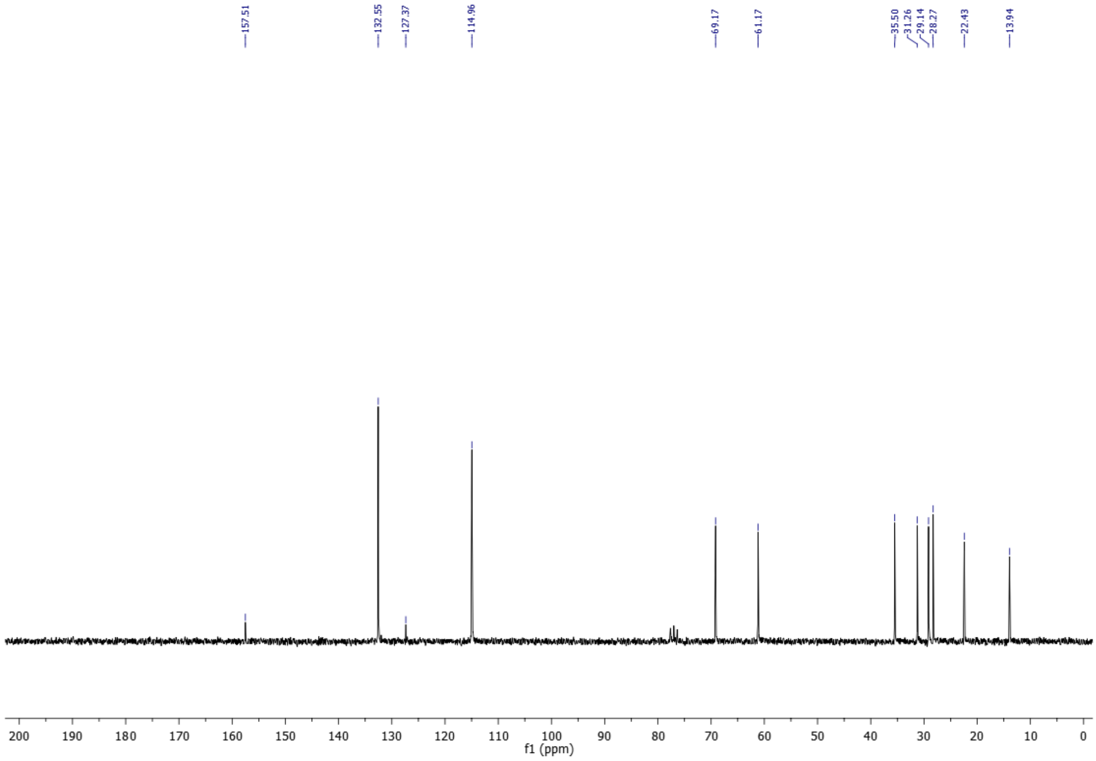

Figure S27. 12g

a. ^{1}H-NMR (400 MHz) of 12g in $CDCl_3$

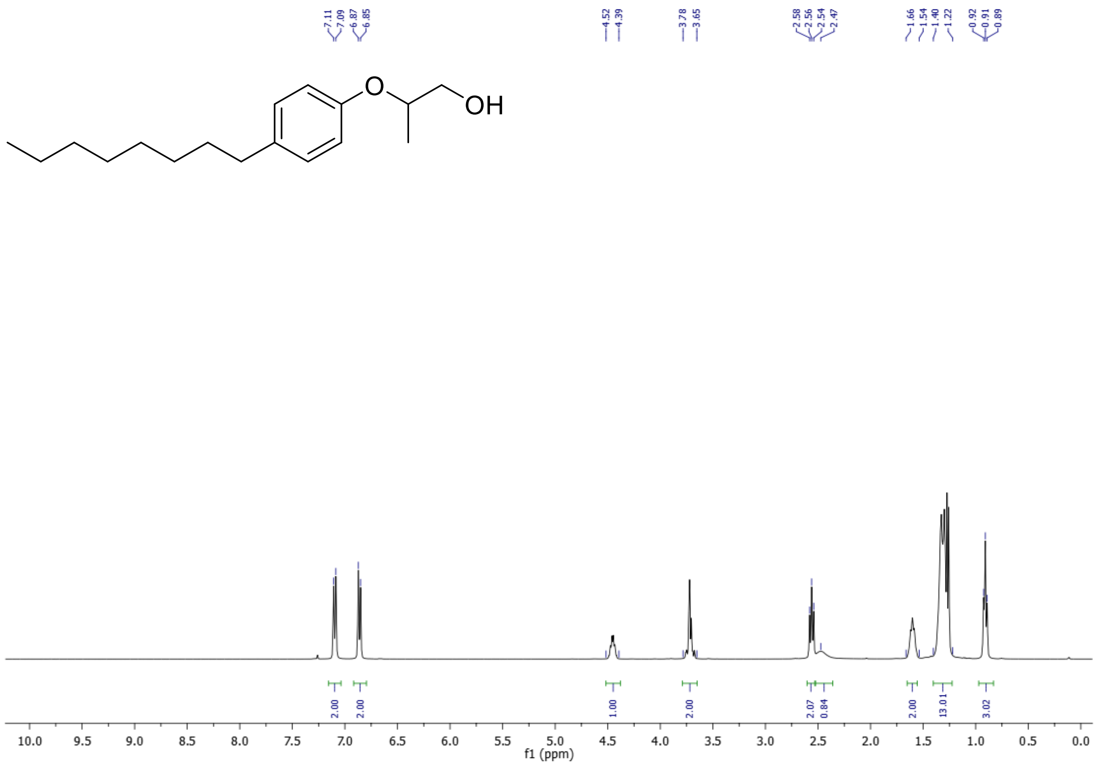

b. ^{13}C-NMR (100 MHz) of 12g in $CDCl_3$

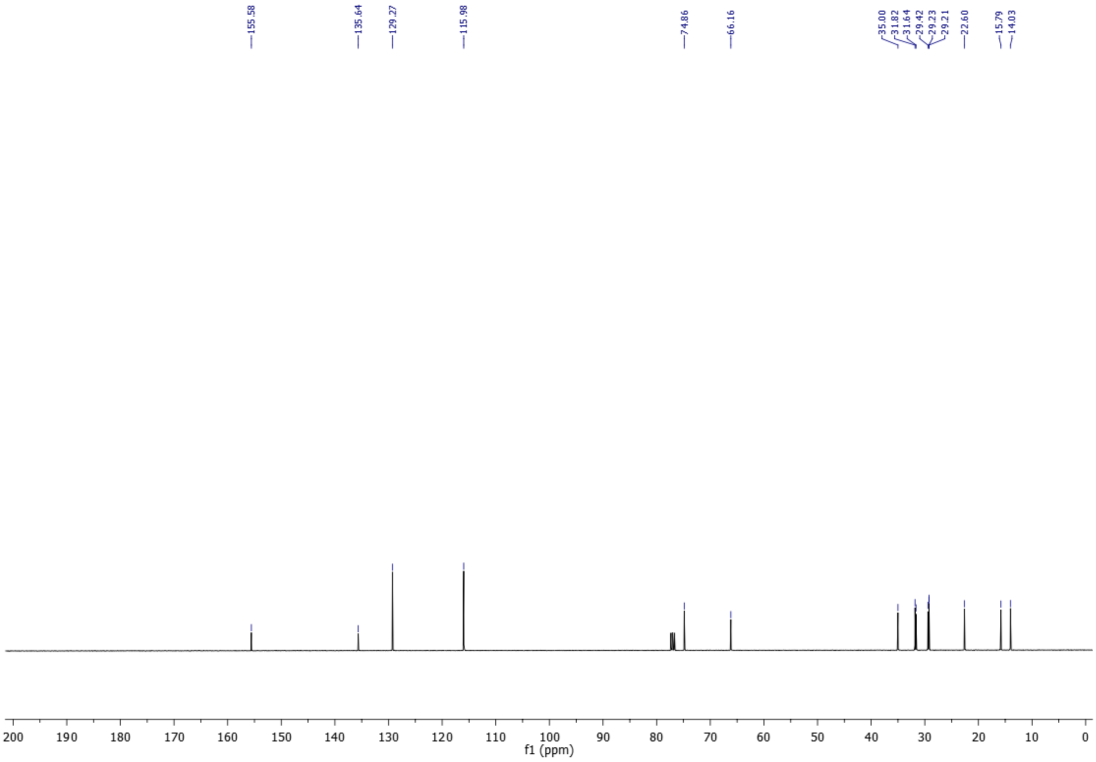

Figure S28. 12h

a. ^{1}H-NMR (200 MHz) of 12h in $CDCl_3$

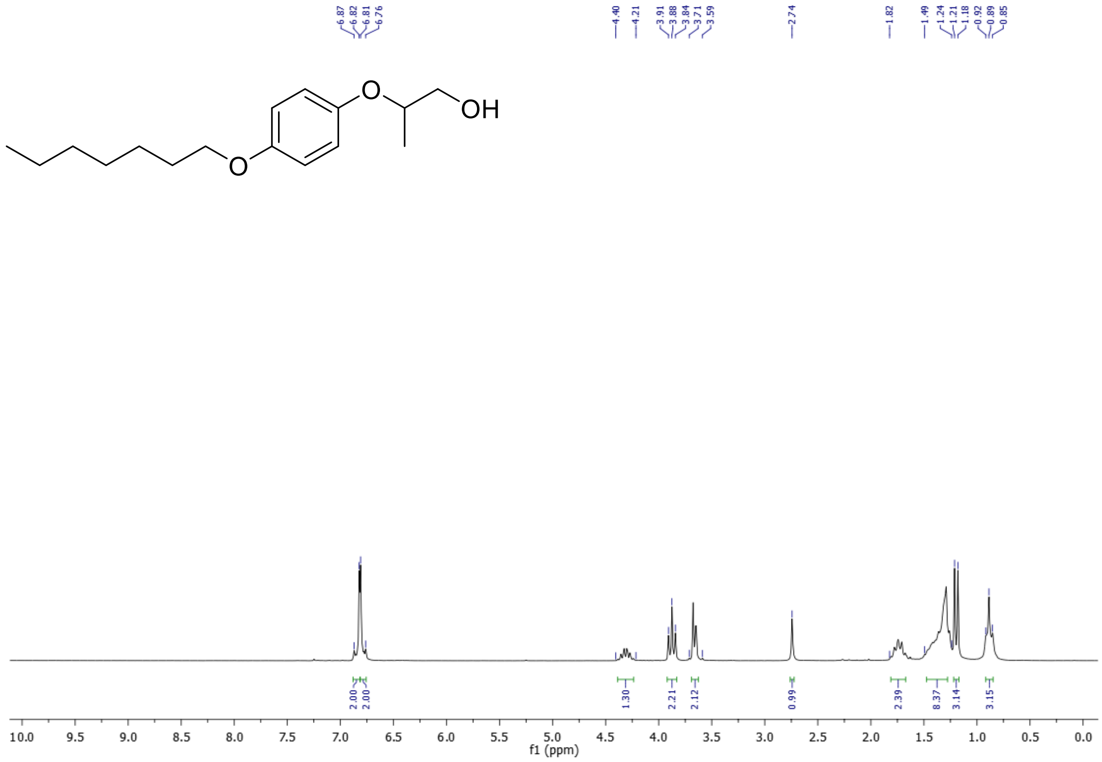

b. ^{13}C-NMR (50 MHz) of 12h in $CDCl_3$

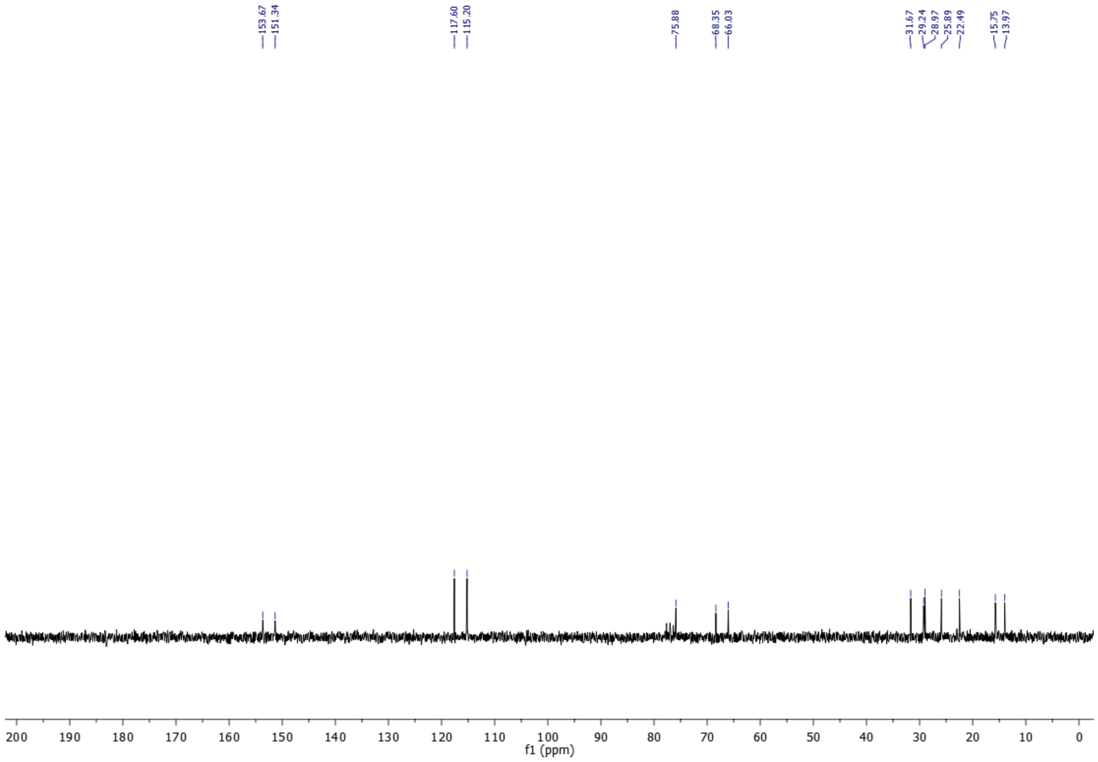

Figure S29. 12i

a. ^{1}H-NMR (400 MHz) of 12i in $CDCl_3$

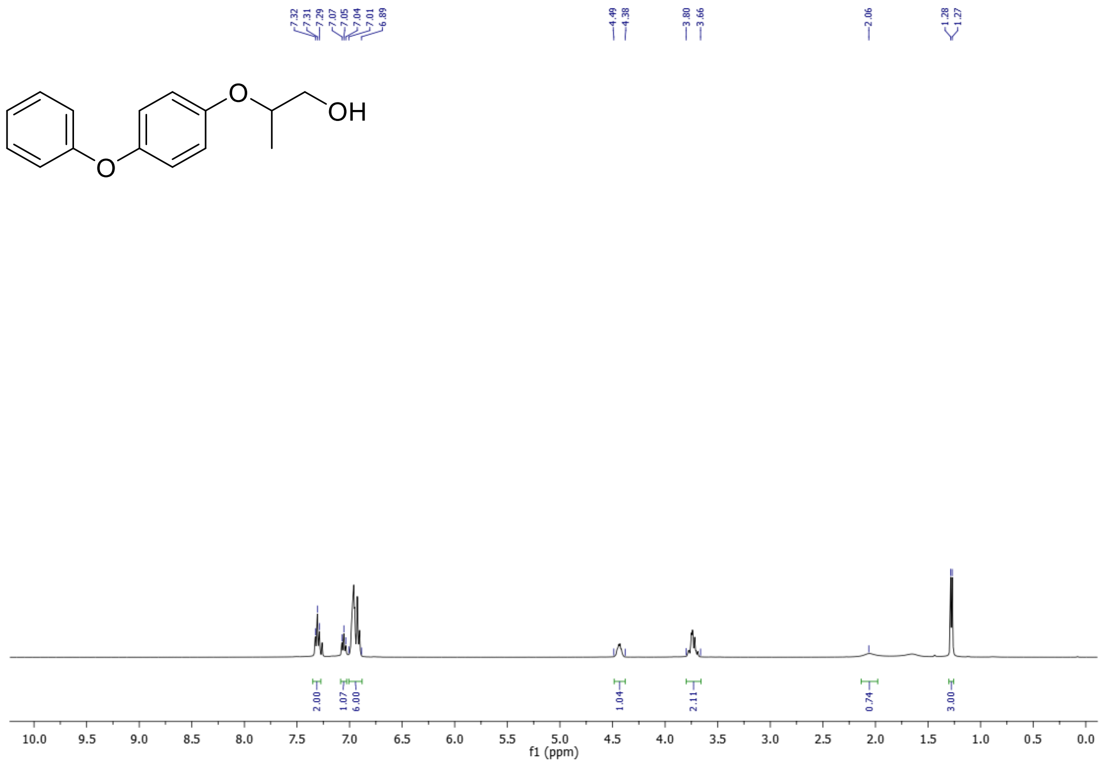

b. ^{13}C-NMR (100 MHz) of 12i in $CDCl_3$

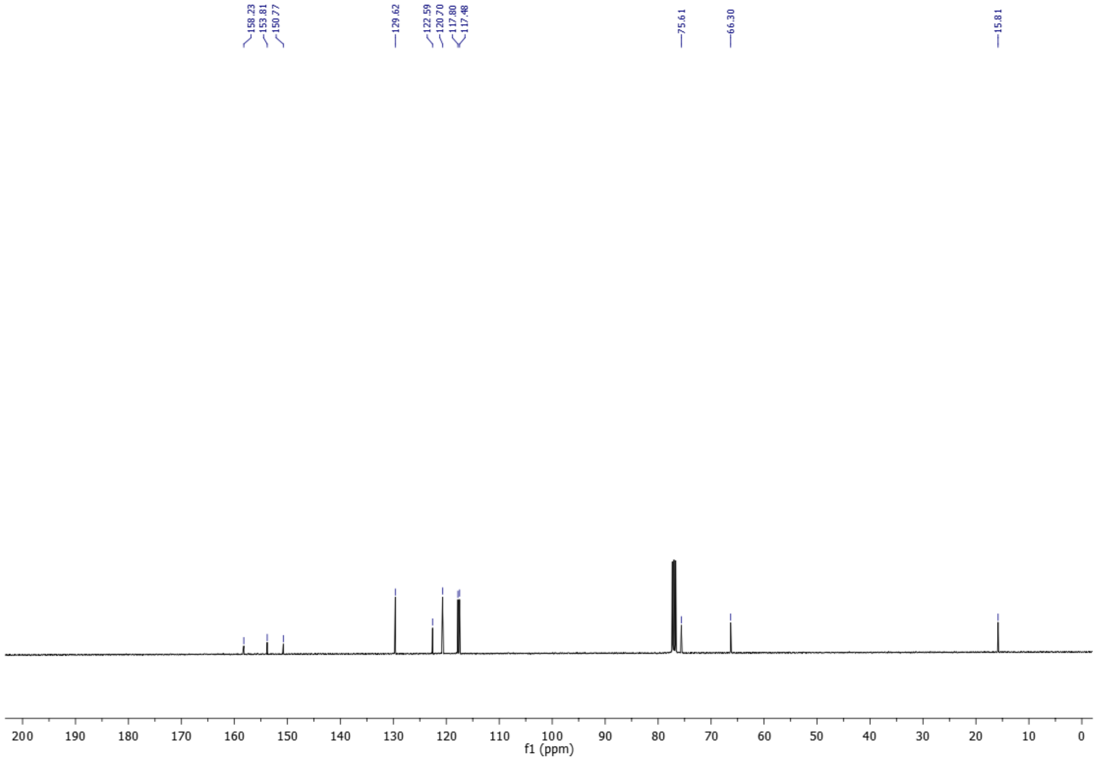

Figure S30. 12j

a.^{1}H-NMR (200 MHz) of 12j in $CDCl_3$

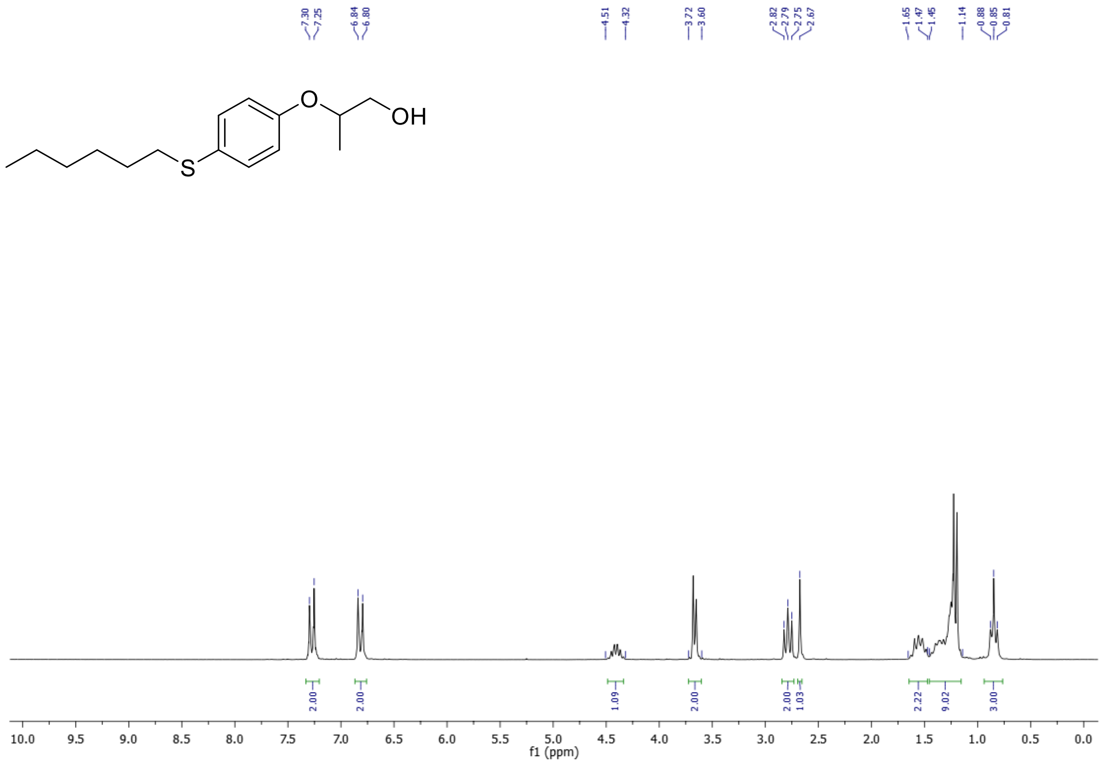

b.^{13}C-NMR (50 MHz) of 12j in $CDCl_3$

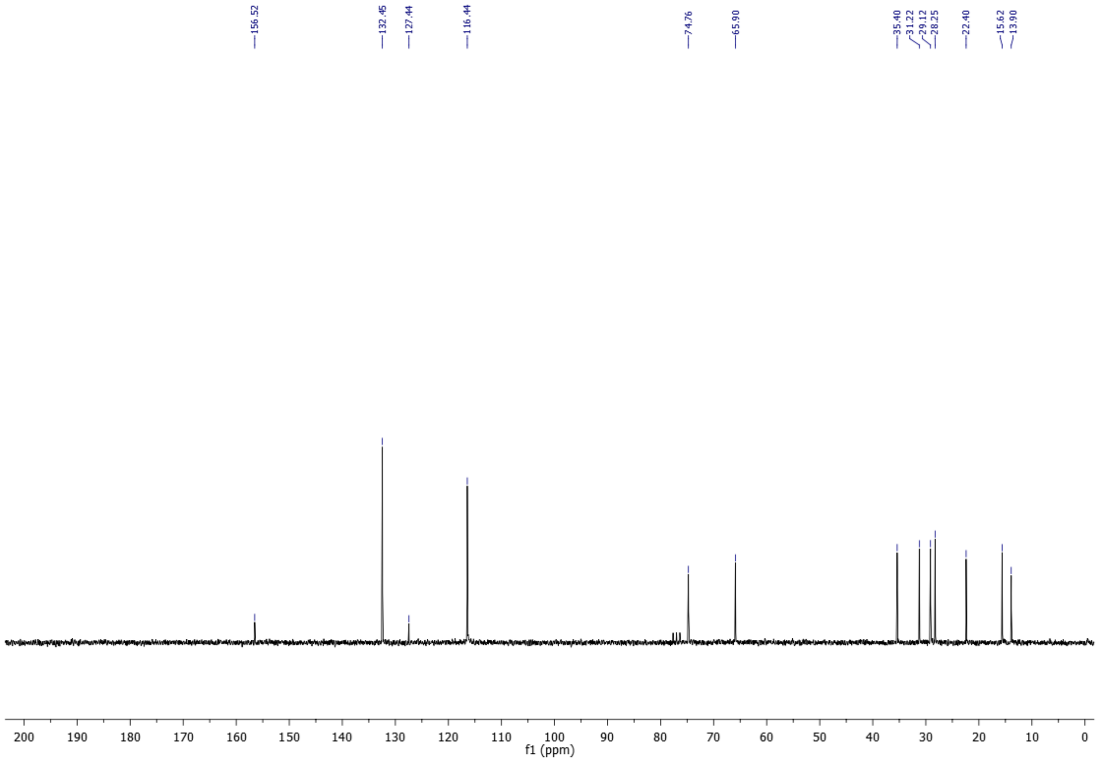

Figure S31. 13b

a. ^{1}H-NMR (200 MHz) of 13b in $CDCl_3$

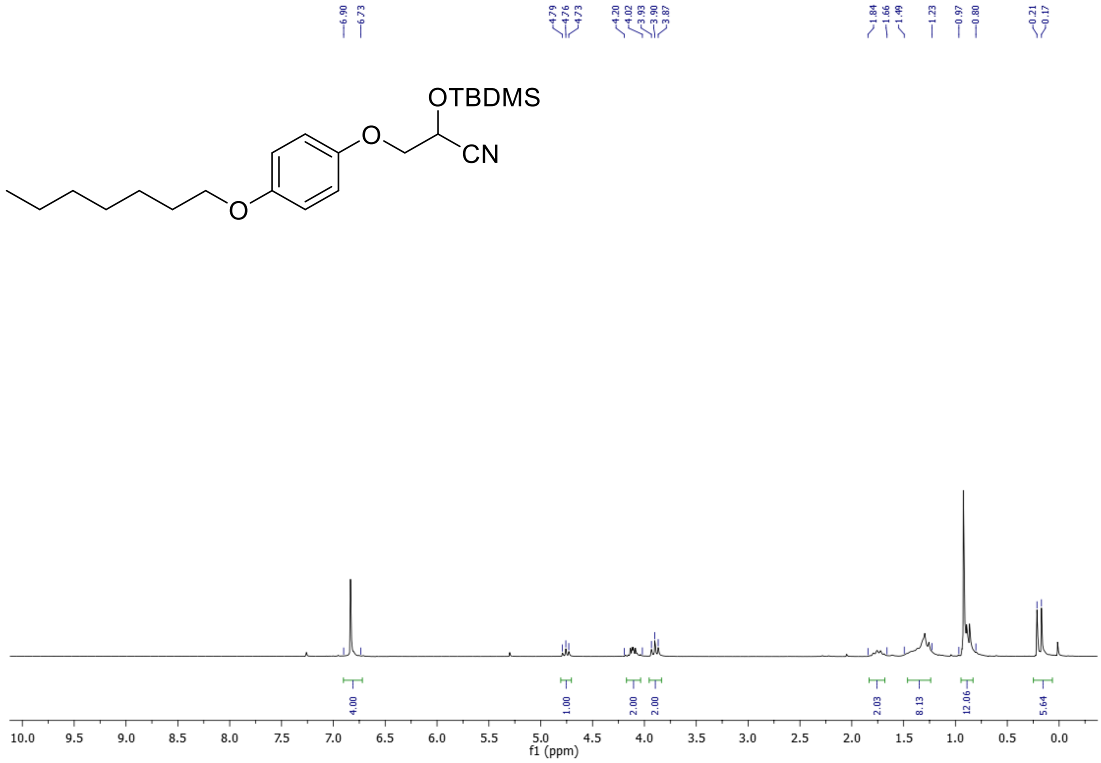

b. ^{13}C-NMR (50 MHz) of 13b in $CDCl_3$

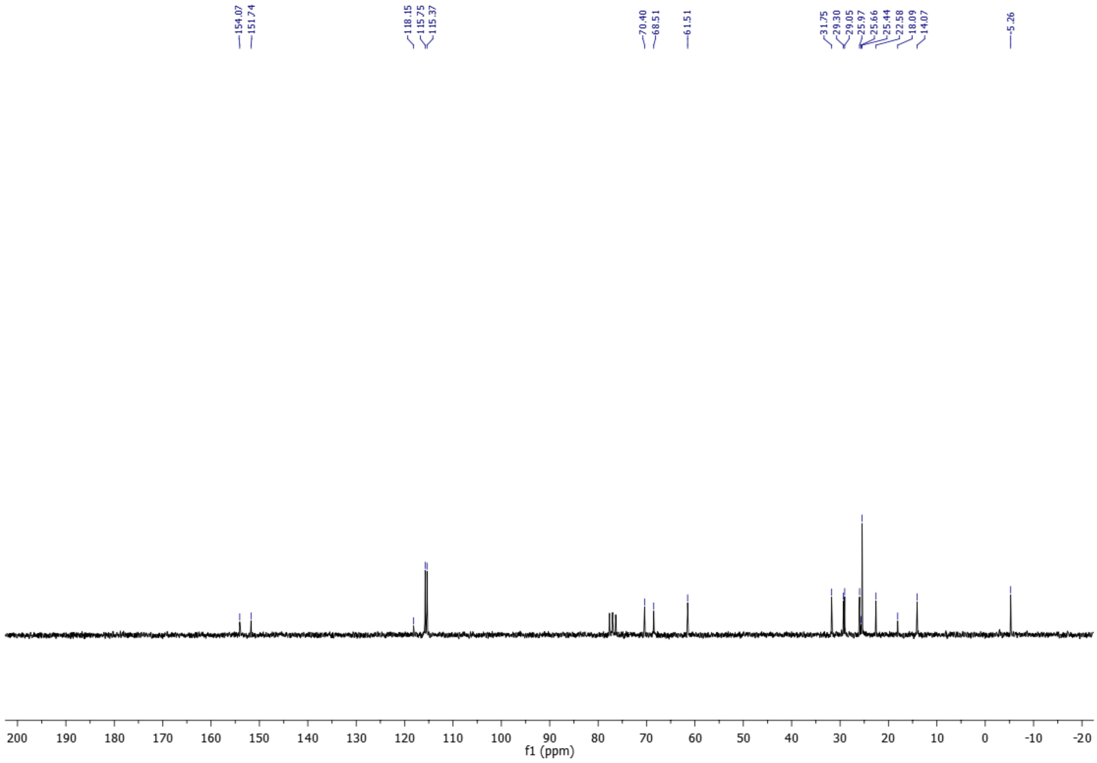

Figure S32. 13h

a. ^{1}H-NMR (200 MHz) of 13h in $CDCl_3$

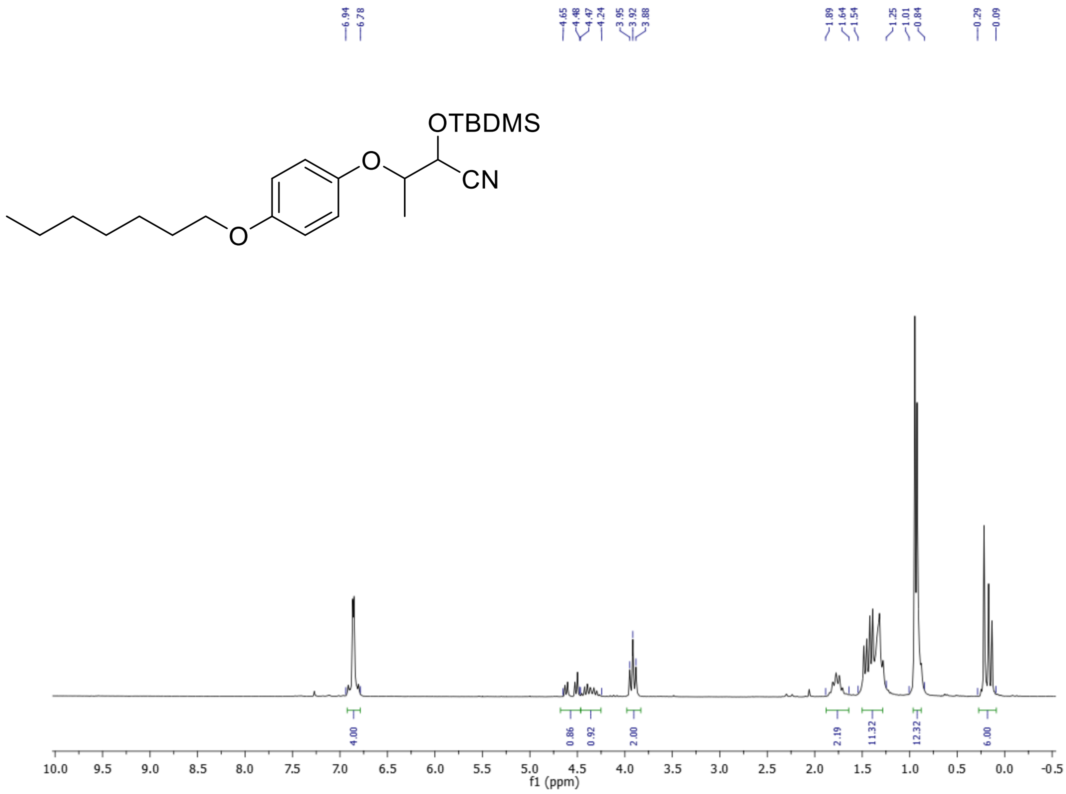

b. ^{13}C-NMR (50 MHz) of 13h in $CDCl_3$

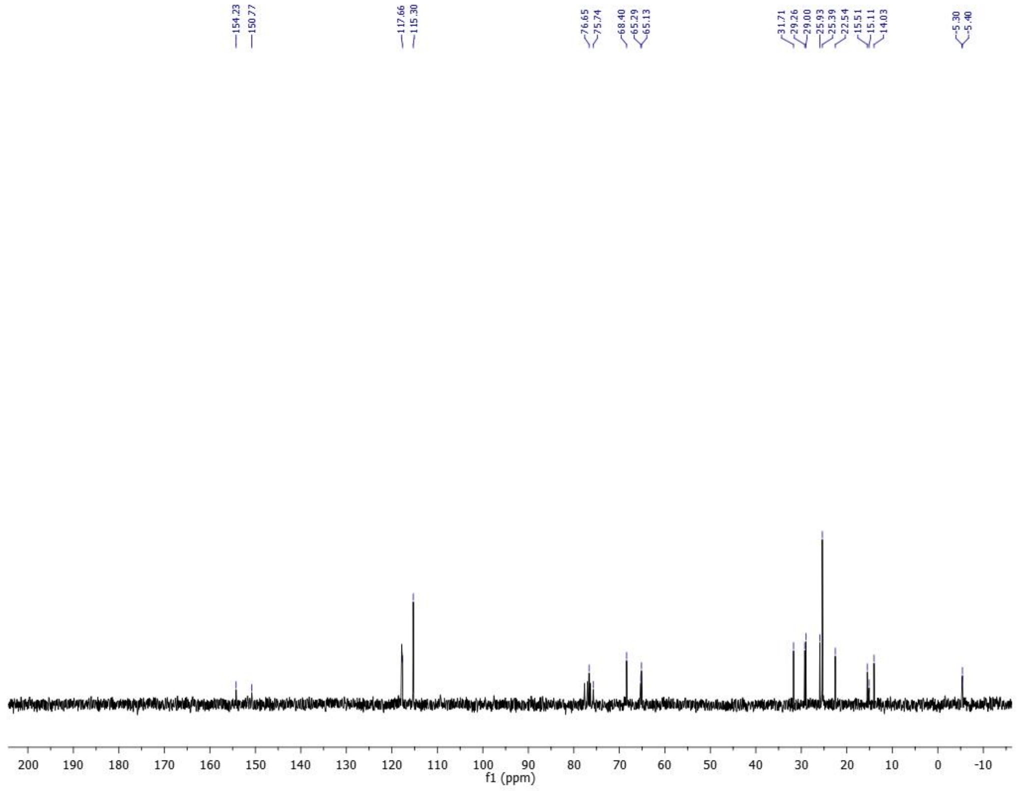

Figure S33. 13j

a. ^{1}H-NMR (200 MHz) of 13j in $CDCl_3$

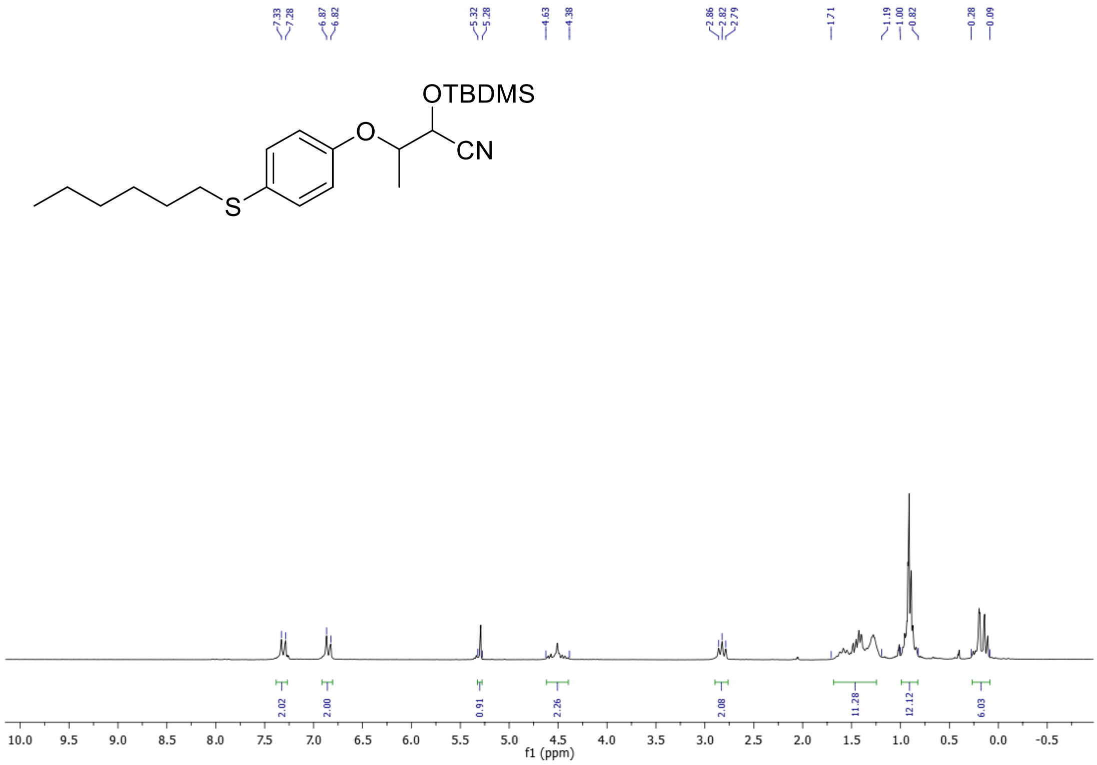

b. ^{13}C-NMR (50 MHz) of 13j in $CDCl_3$

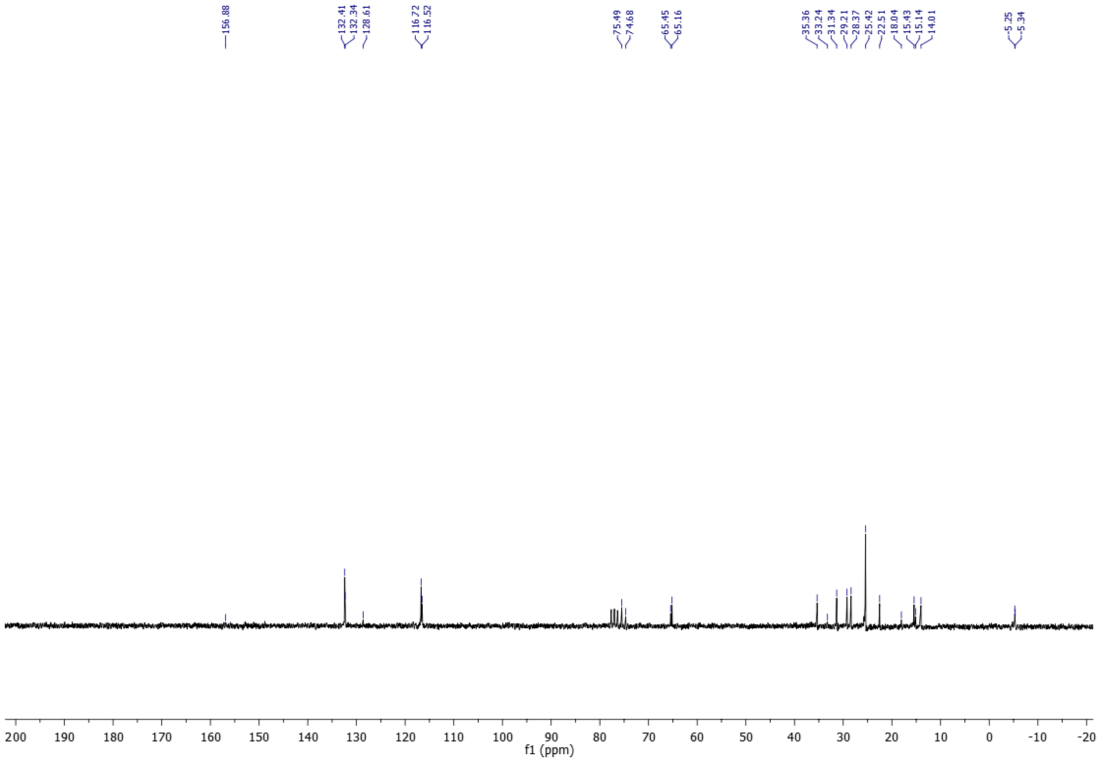

Figure S34. 14a

a. ^{1}H-NMR (200 MHz) of 14a in $CDCl_3$

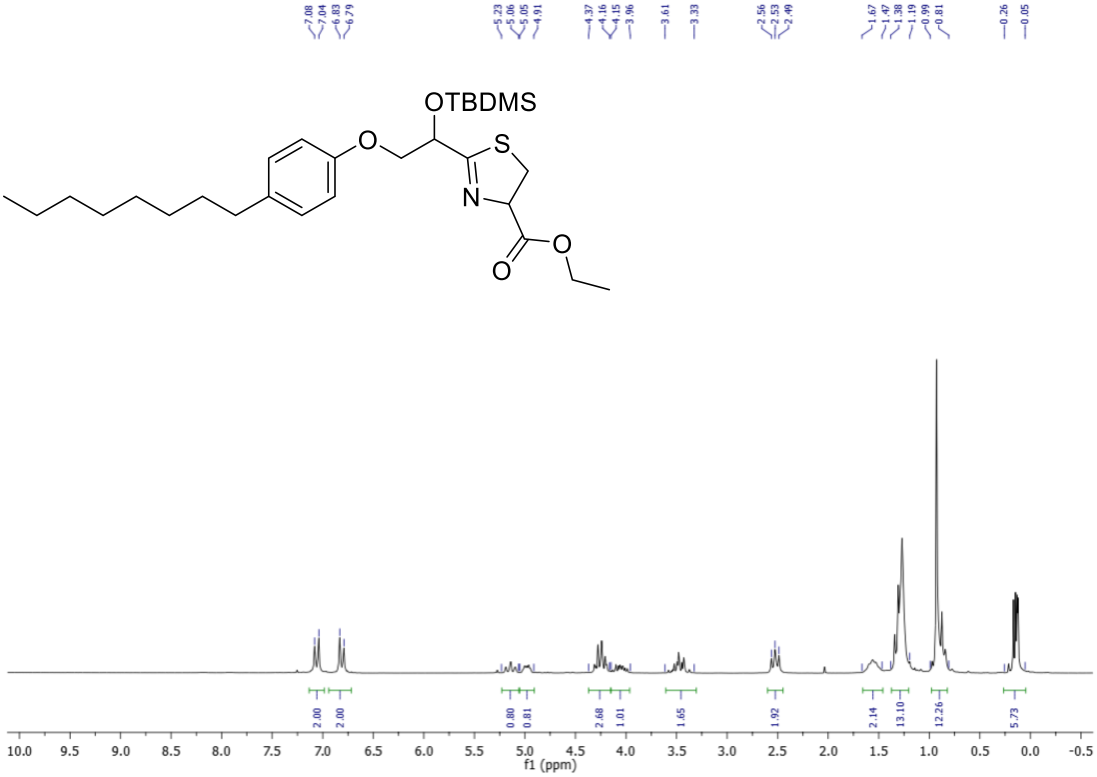

b. ^{13}C-NMR (50 MHz) of 14a in $CDCl_3$

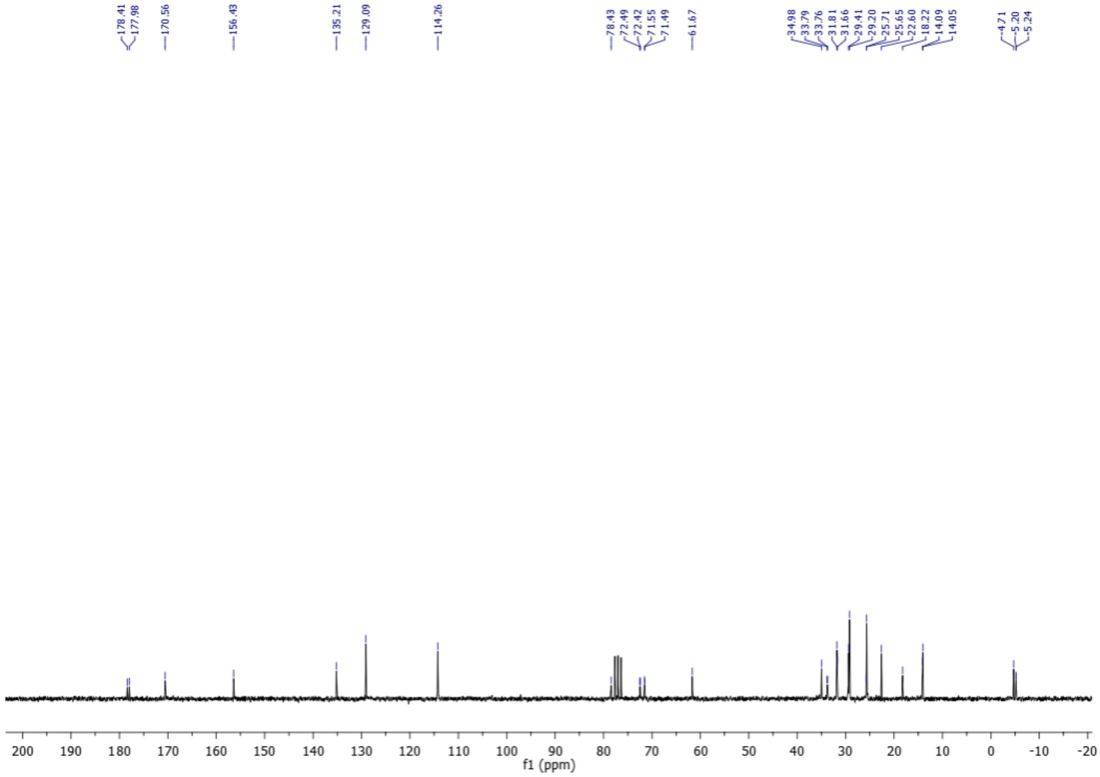

Figure S35. 14b

a. ^{1}H-NMR (200 MHz) of 14b in CDCl$_3$

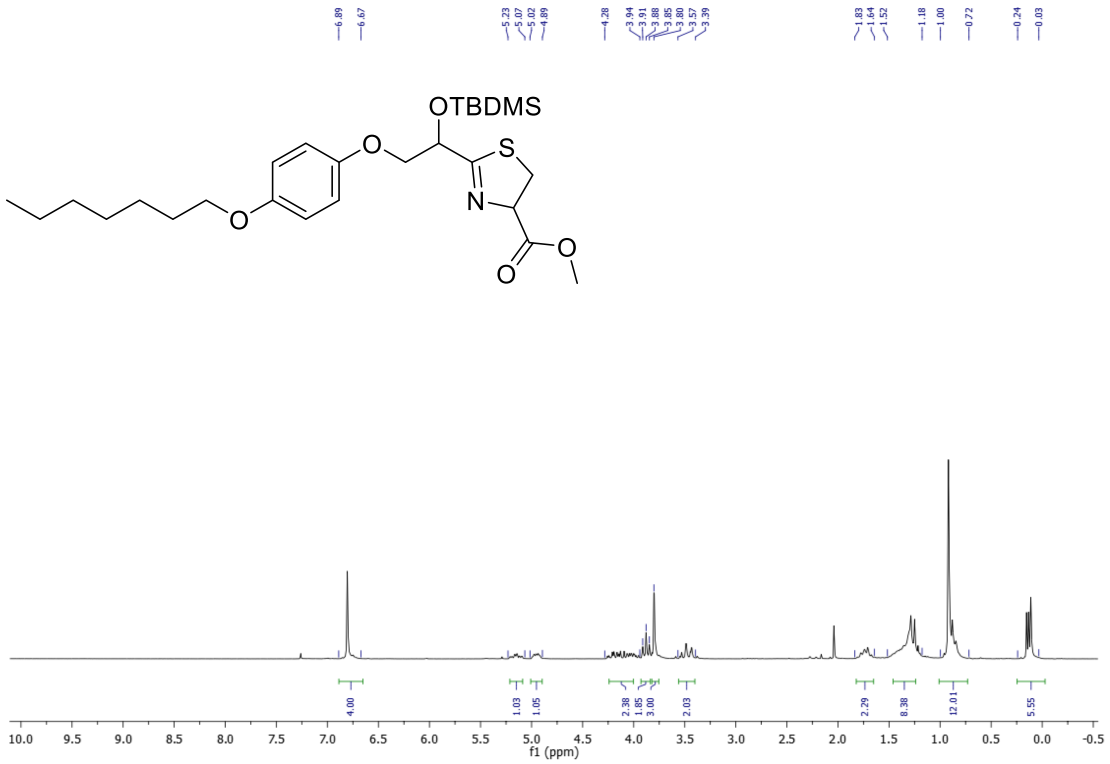

b. ^{13}C-NMR (50 MHz) of 14b in CDCl$_3$

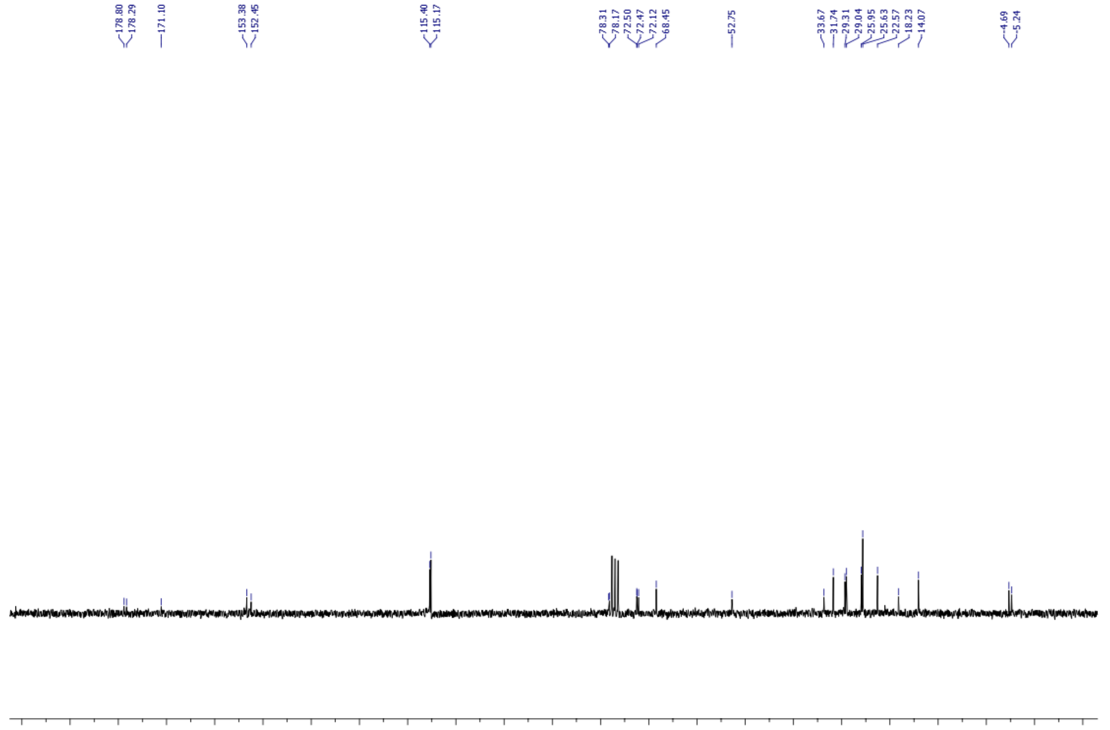

Figure S36. 14d

a. ^{1}H-NMR (400 MHz) of 14d in $CDCl_3$

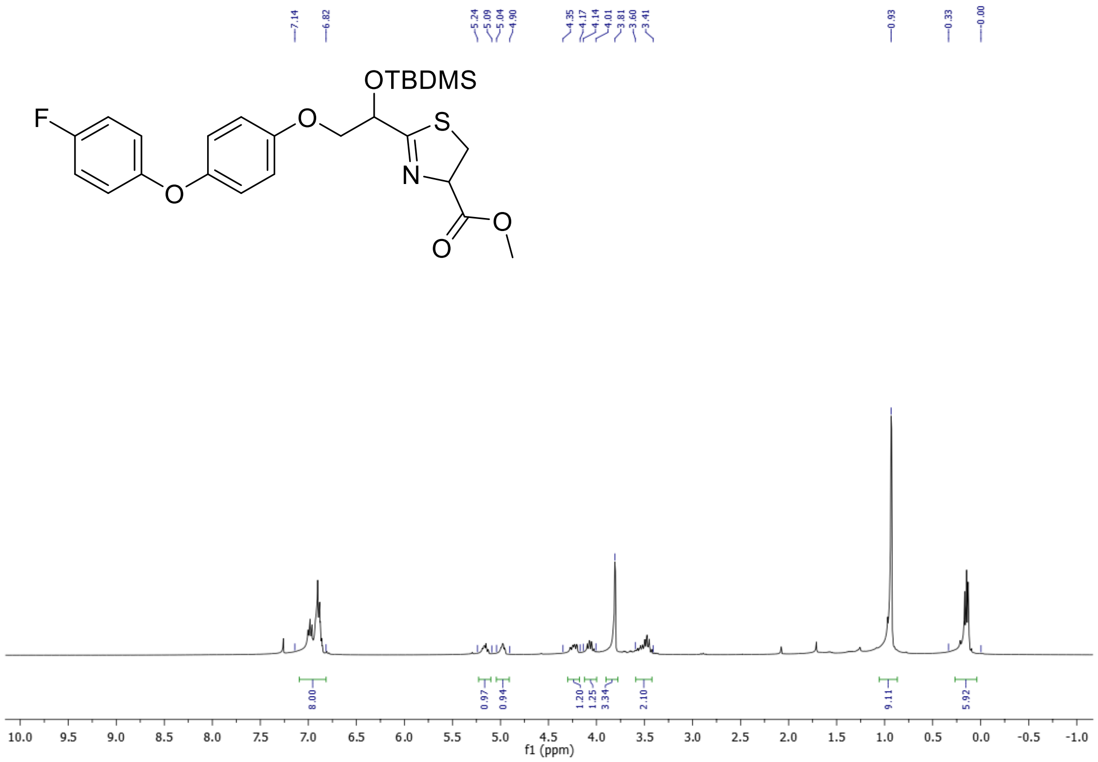

b. ^{13}C-NMR (100 MHz) of 14d in $CDCl_3$

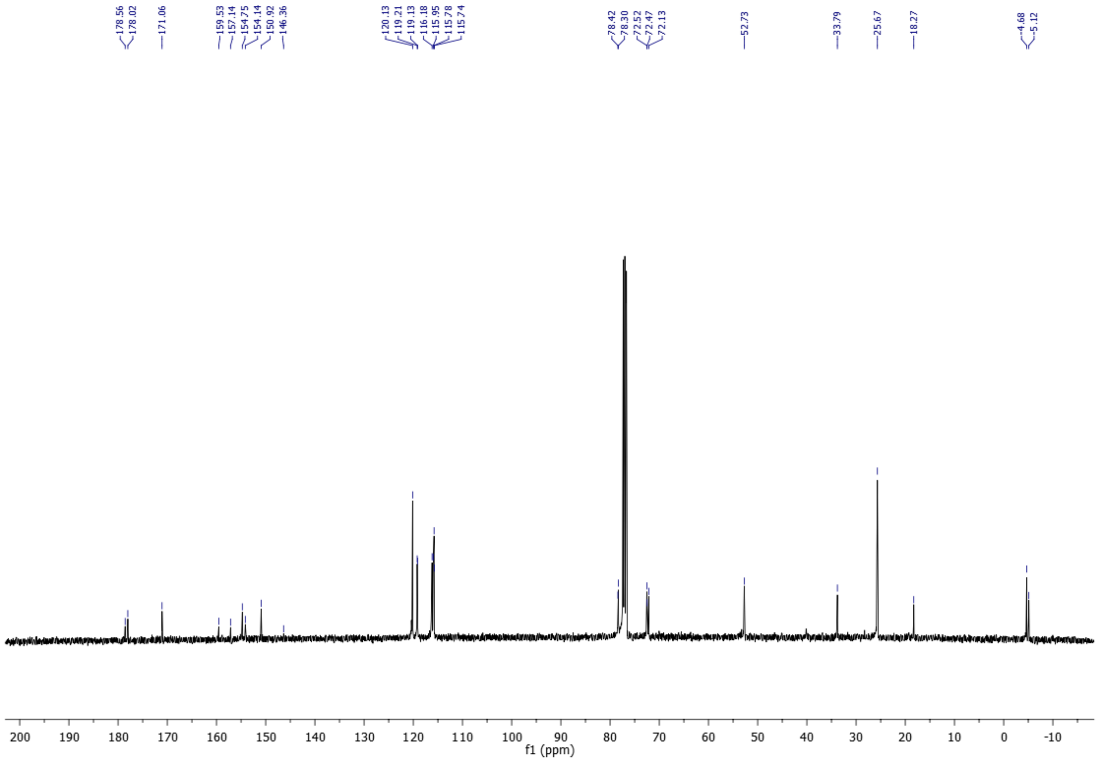

c. ^{19}F-NMR (377 MHz) of 14d in $CDCl_3$

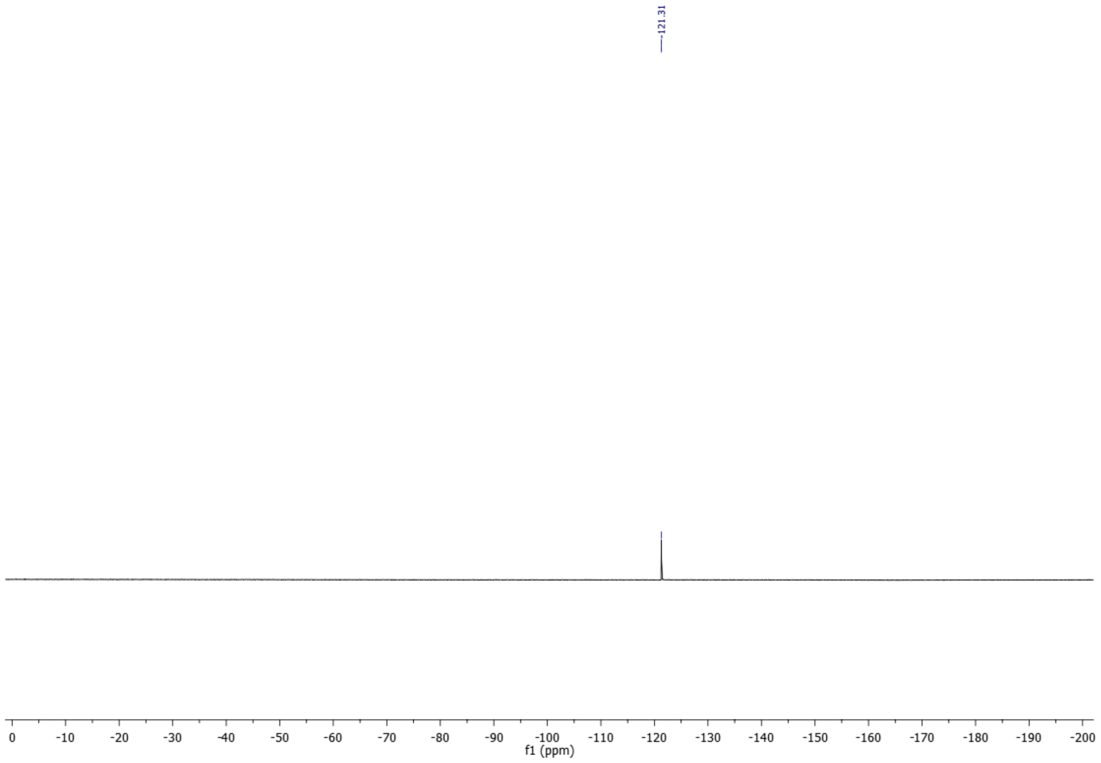

Figure S37. 14e

a. ^{1}H-NMR (200 MHz) of 14e in $CDCl_3$

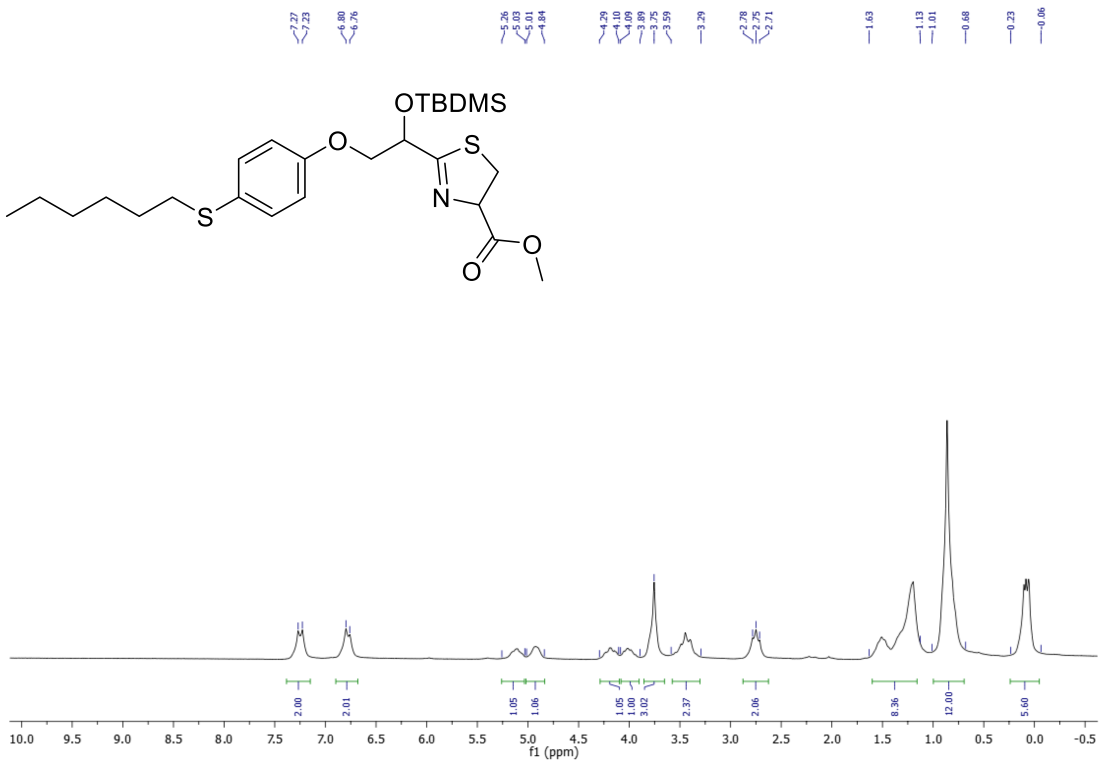

b. ^{13}C-NMR (50 MHz) of 14e in $CDCl_3$

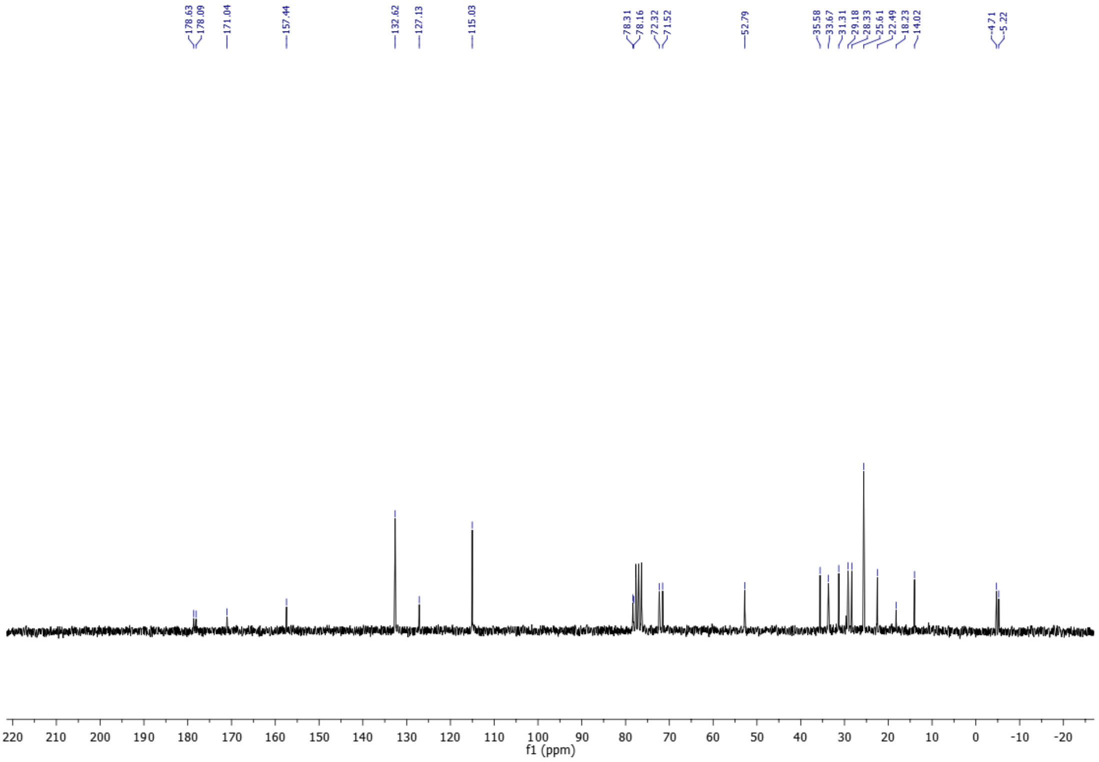

Figure S38. 14h

a. ^{1}H-NMR (400 MHz) of 14h in $CDCl_3$

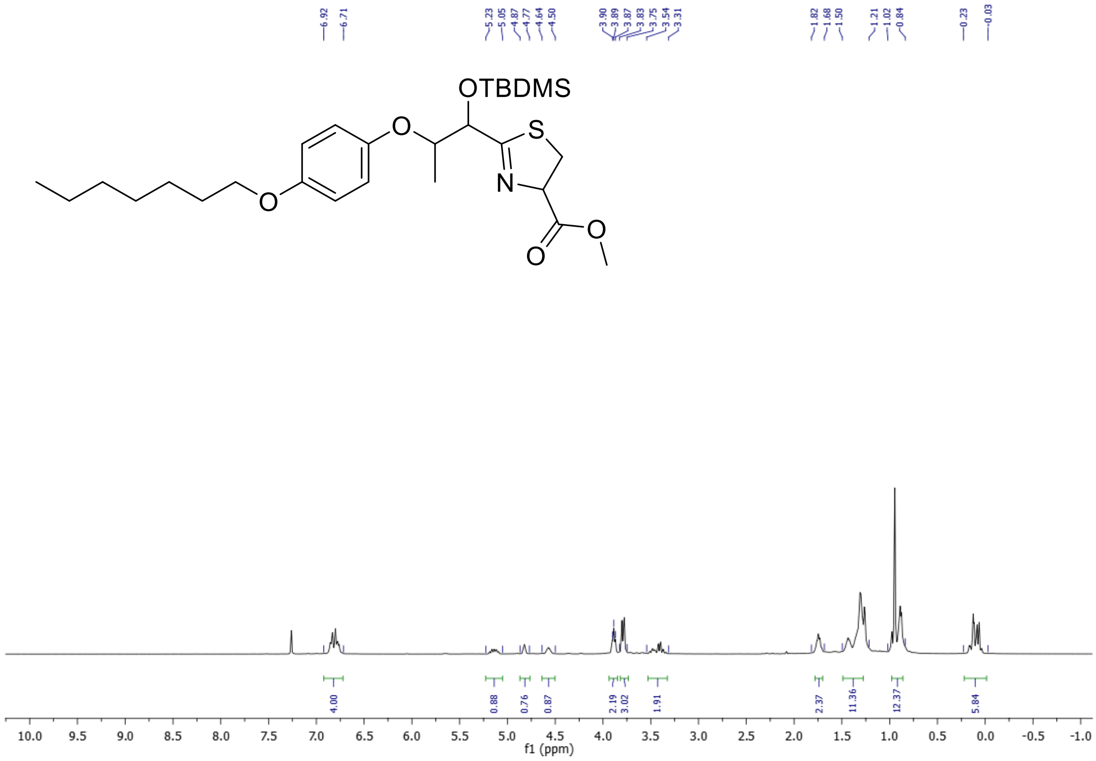

b. ^{13}C-NMR (100 MHz) of 14h in $CDCl_3$

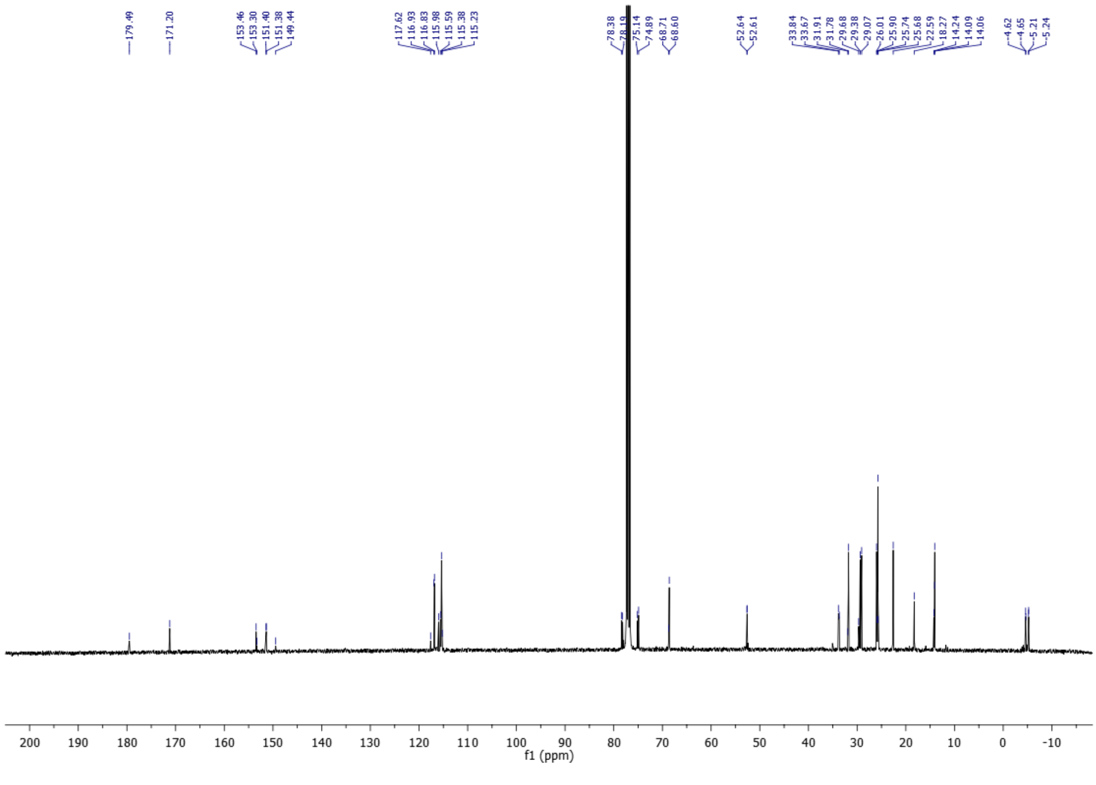

Figure S39. 14j

a. ^{1}H-NMR (200 MHz) of 14j in $CDCl_3$

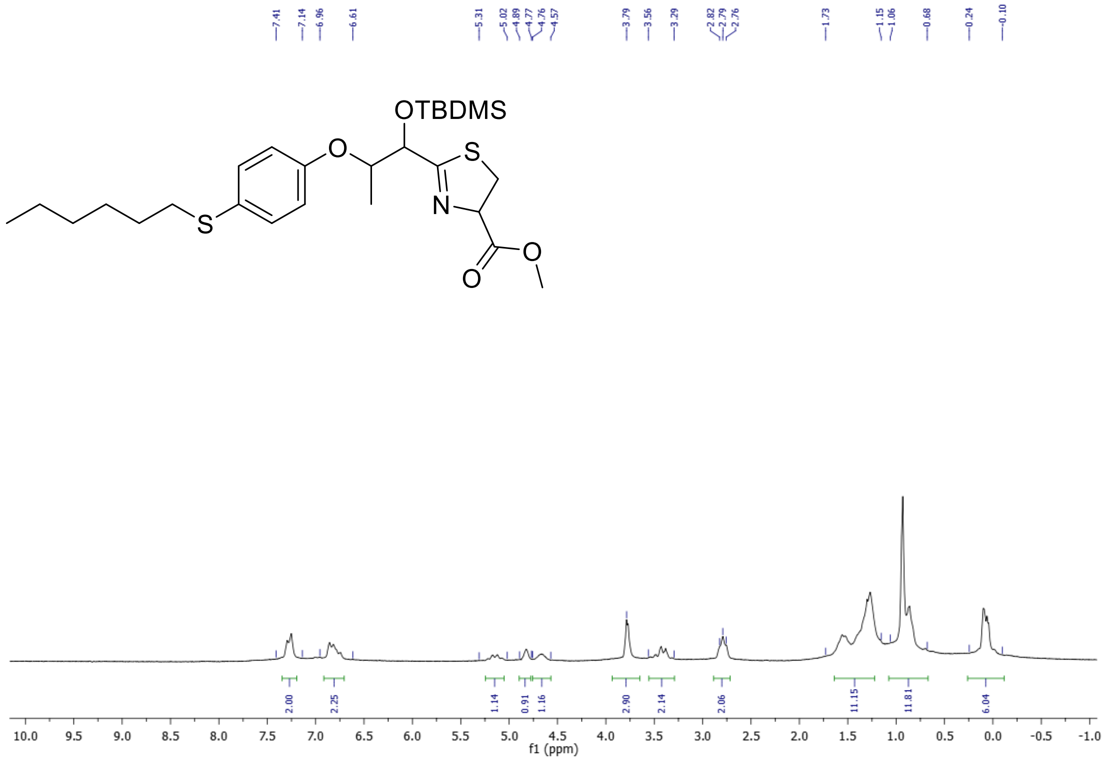

b. ^{13}C-NMR (50 MHz) of 14j in $CDCl_3$

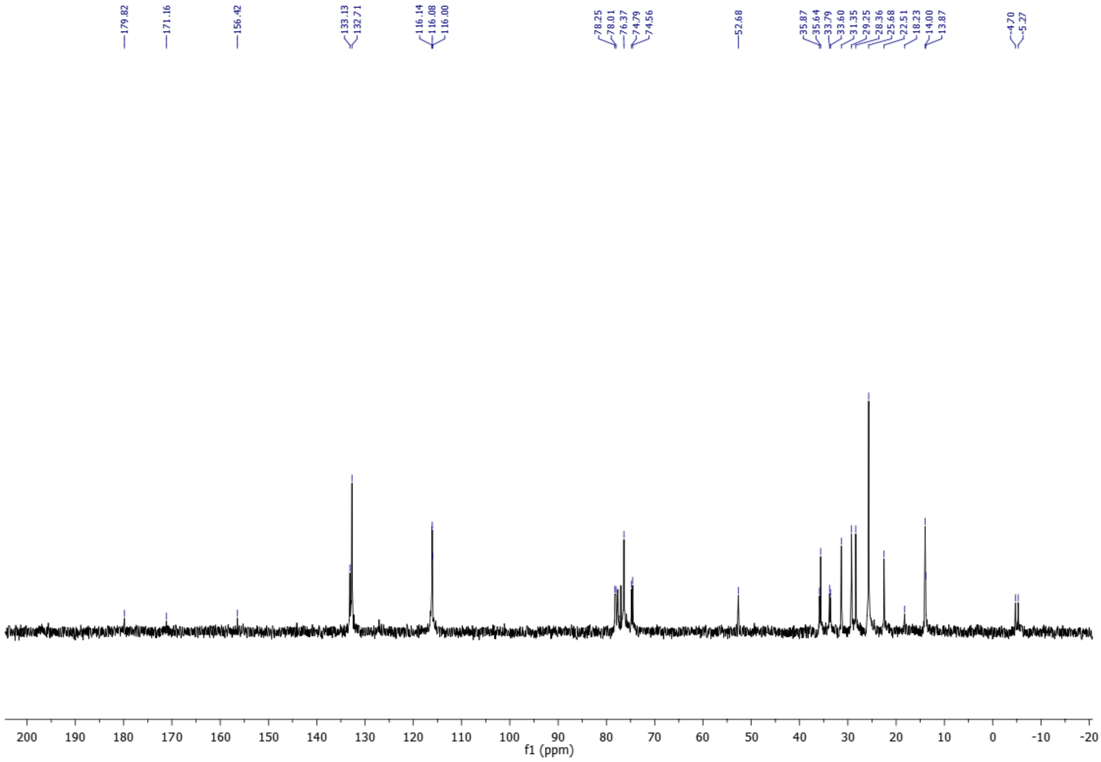

Figure S40. 15a

a. ^{1}H-NMR (200 MHz) of 15a in $CDCl_3$

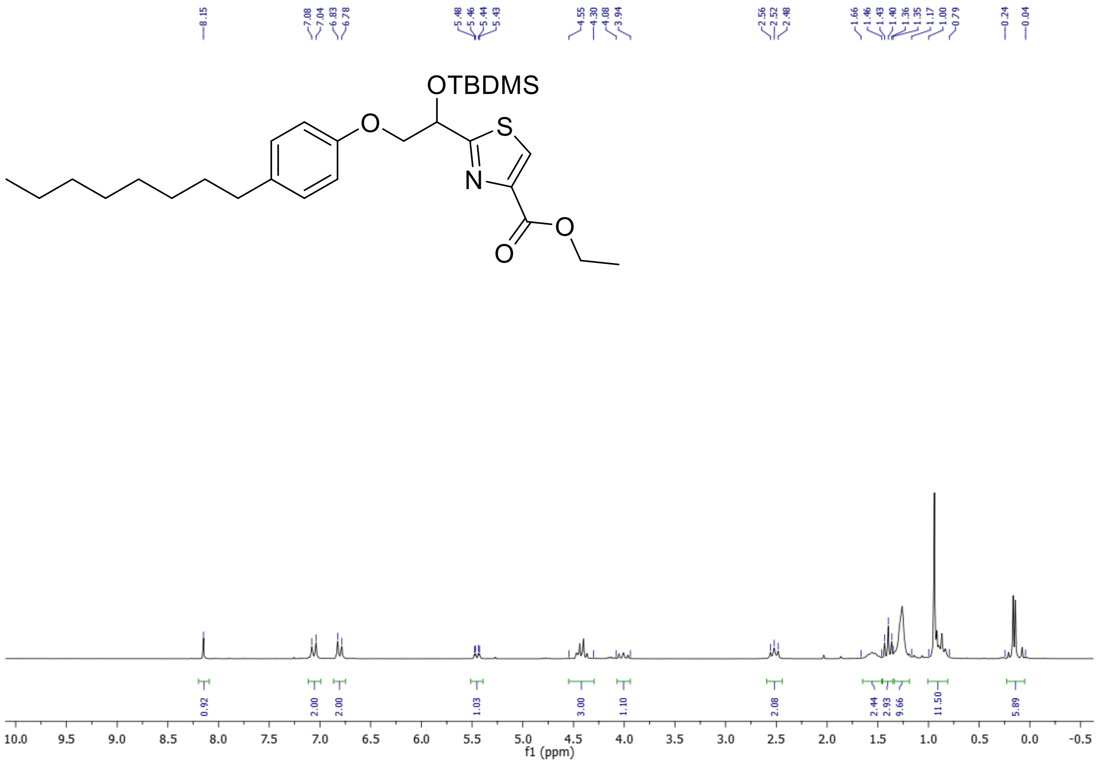

b. ^{13}C-NMR (50 MHz) of 15a in $CDCl_3$

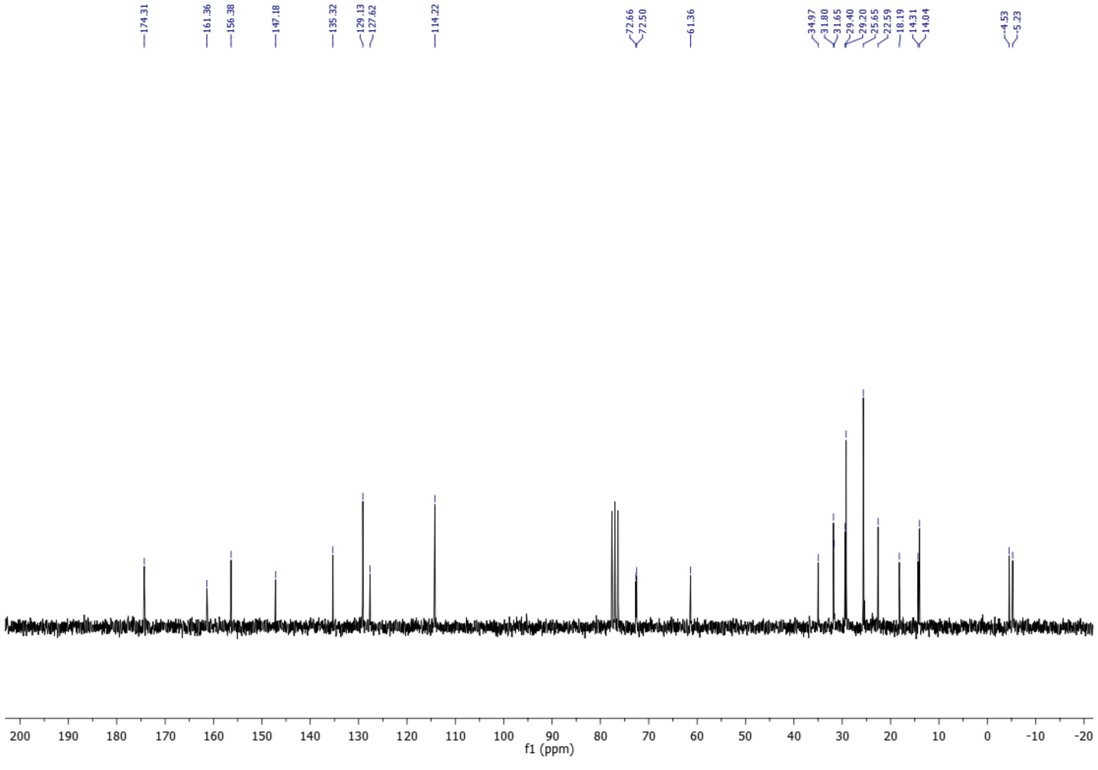

Figure S41. 15b

a. ^{1}H-NMR (200 MHz) of 15b in $CDCl_3$

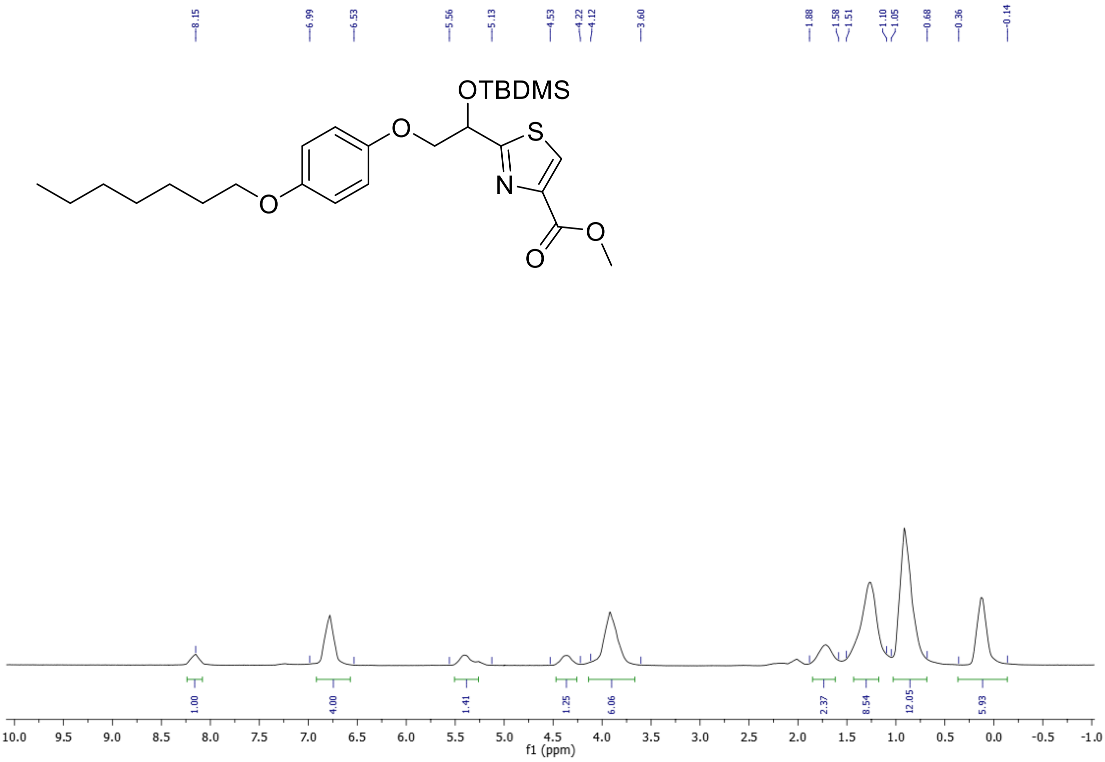

b. ^{13}C-NMR (50 MHz) of 15b in $CDCl_3$

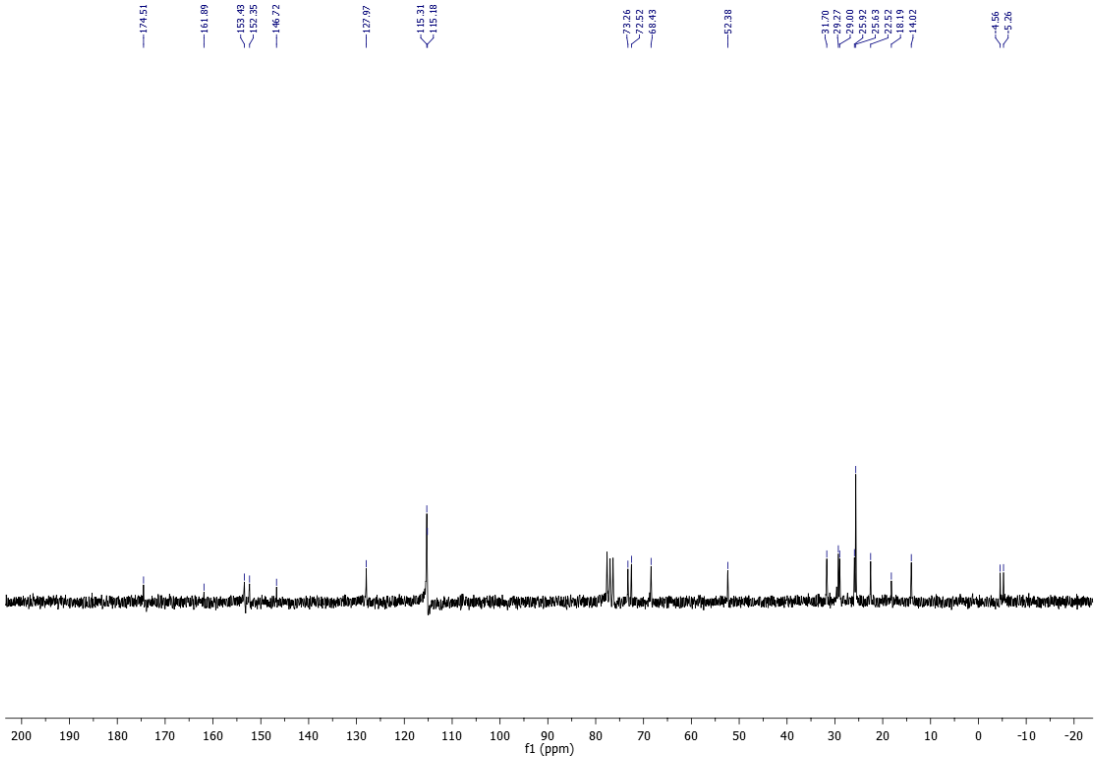

Figure S42. 15c

a. ^{1}H-NMR (200 MHz) of 15c in $CDCl_3$

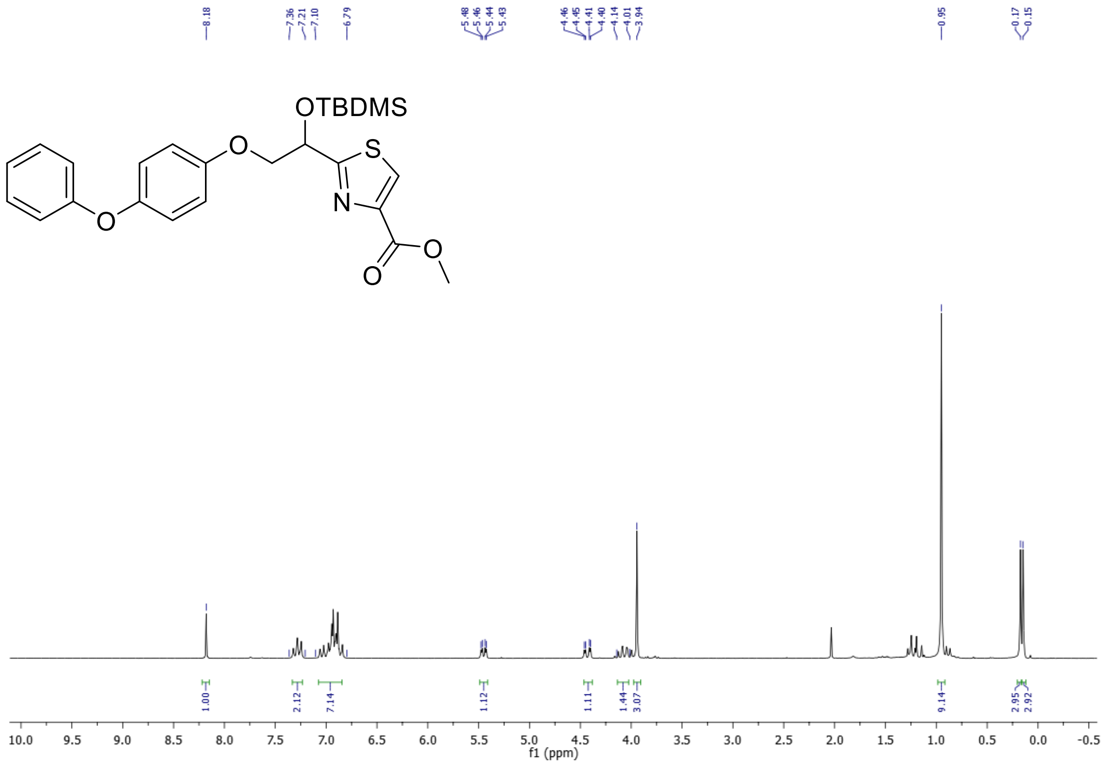

b. ^{13}C-NMR (50 MHz) of 15c in $CDCl_3$

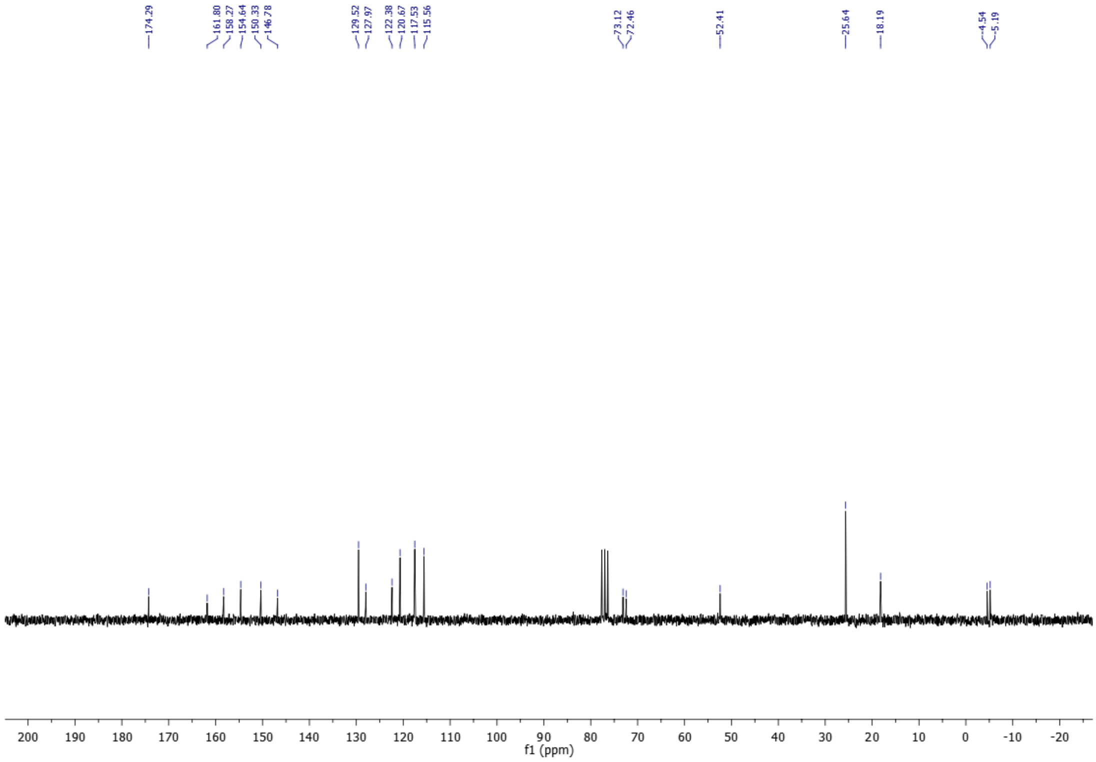

Figure S43. 15d

a. ^{1}H-NMR (400 MHz) of 15d in $CDCl_3$

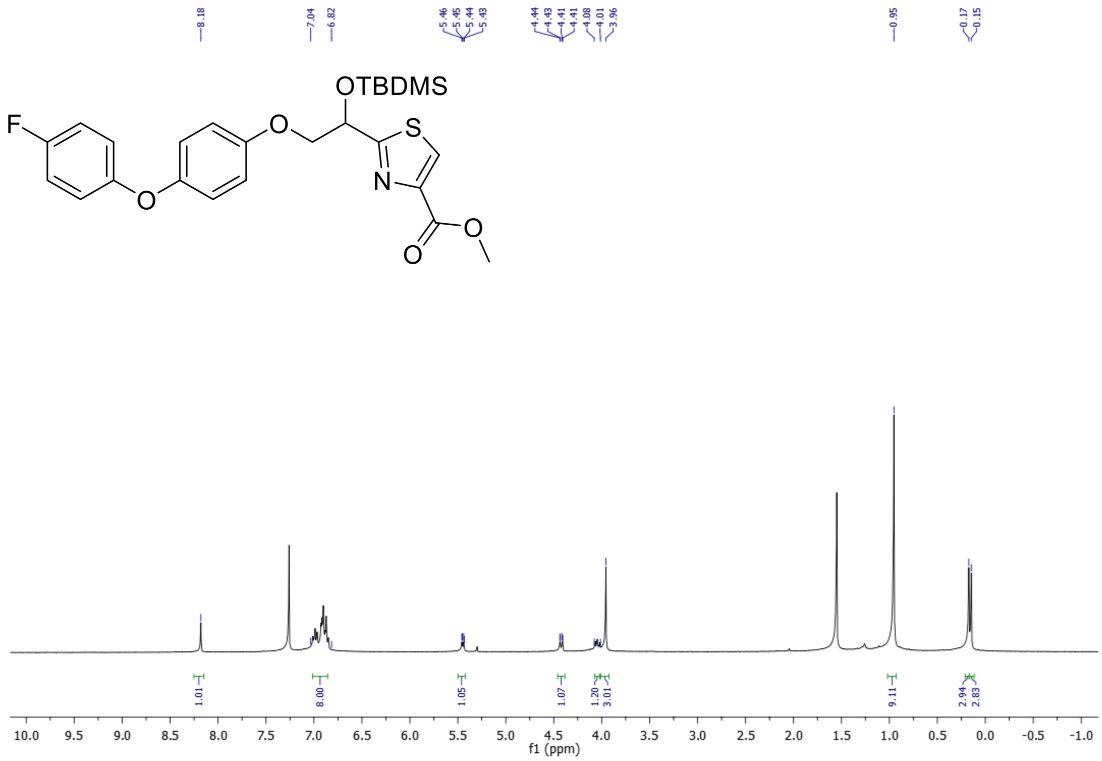

b. ^{13}C-NMR (100 MHz) of 15d in $CDCl_3$

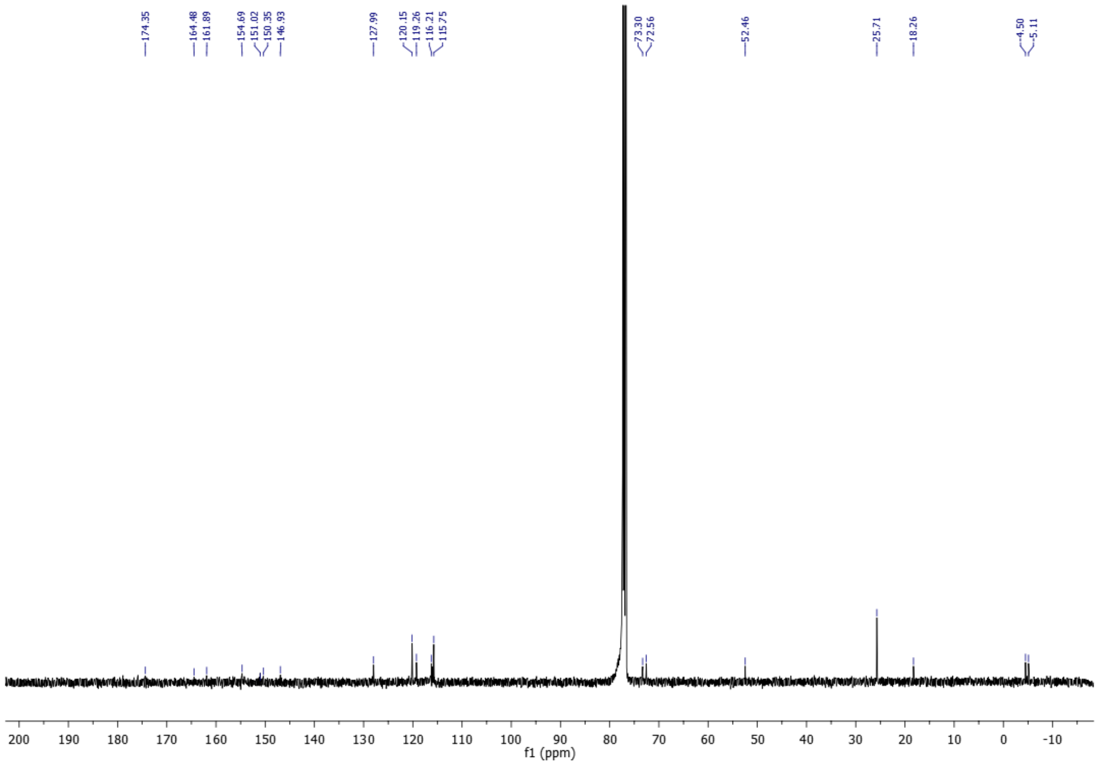

c. ^{19}F-NMR (377 MHz) of 15d in $CDCl_3$

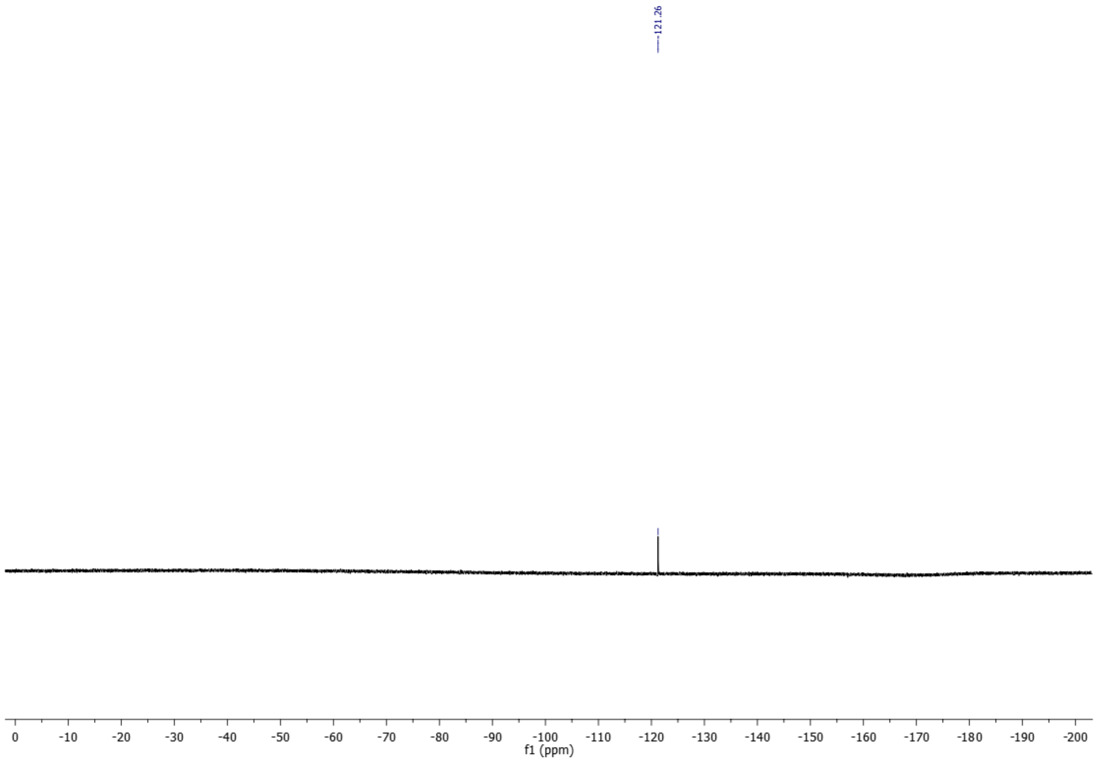

Figure S44. 15e

a. ^{1}H-NMR (200 MHz) of 15e in $CDCl_3$

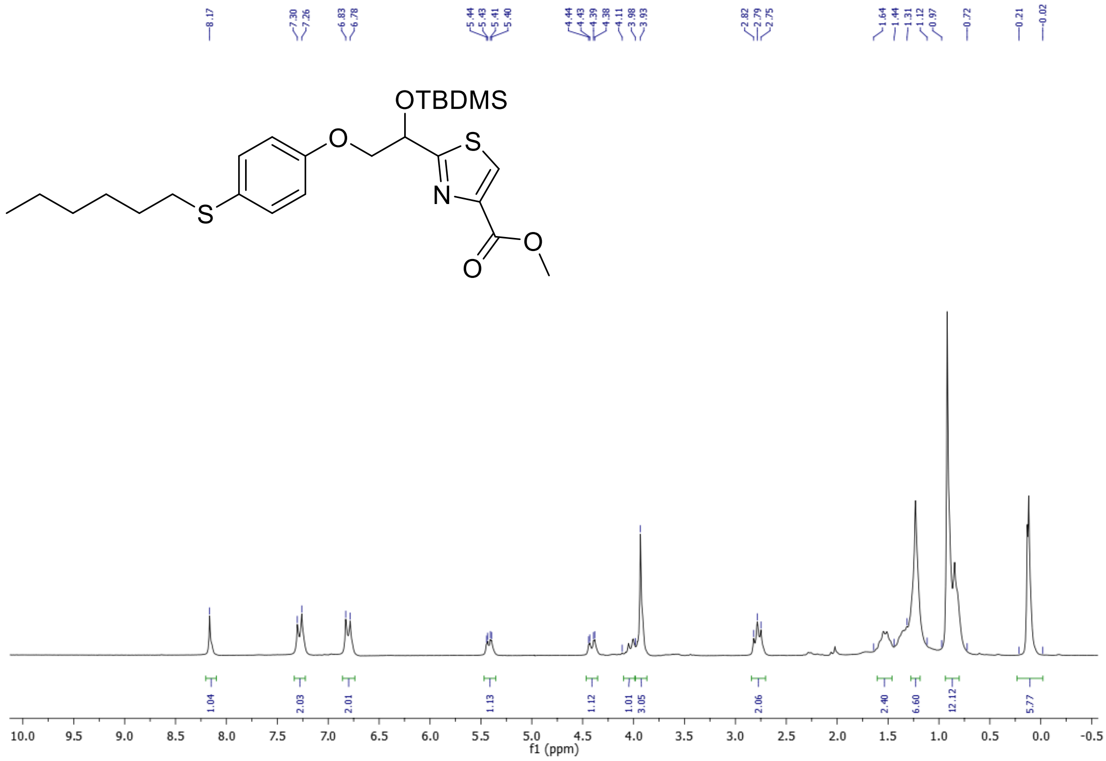

b. ^{13}C-NMR (50 MHz) of 15e in $CDCl_3$

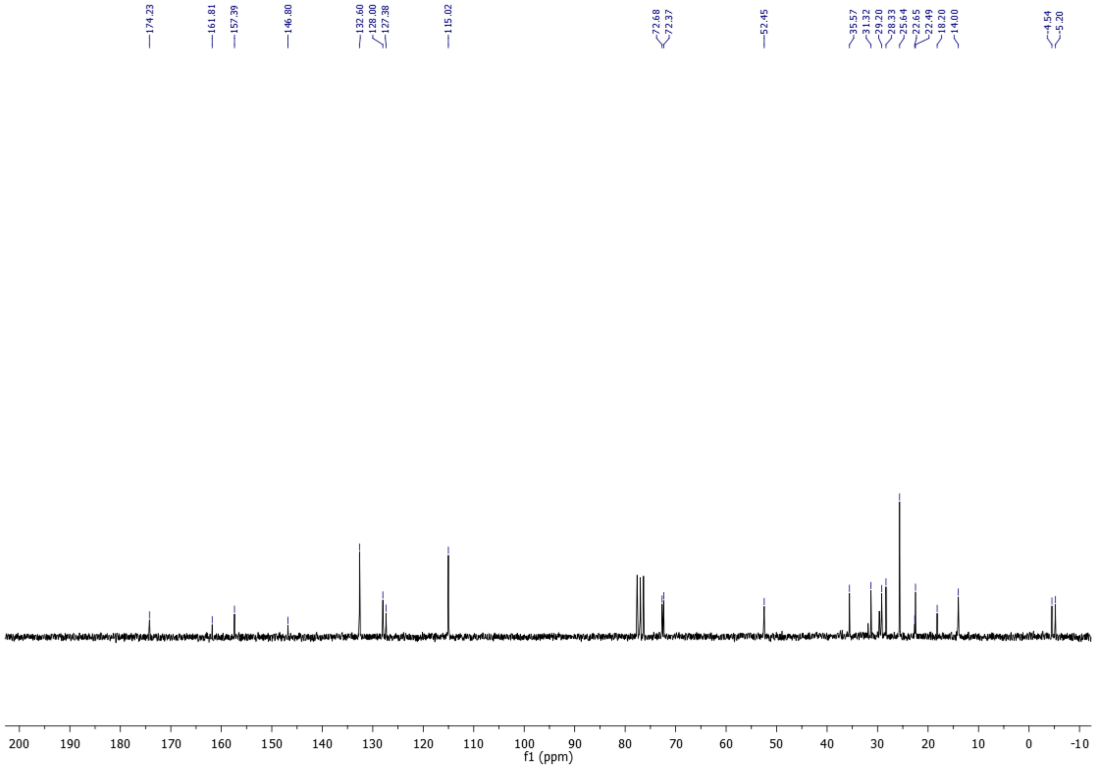

Figure S45. 15f

a. ^{1}H-NMR (200 MHz) of 15f in $CDCl_3$

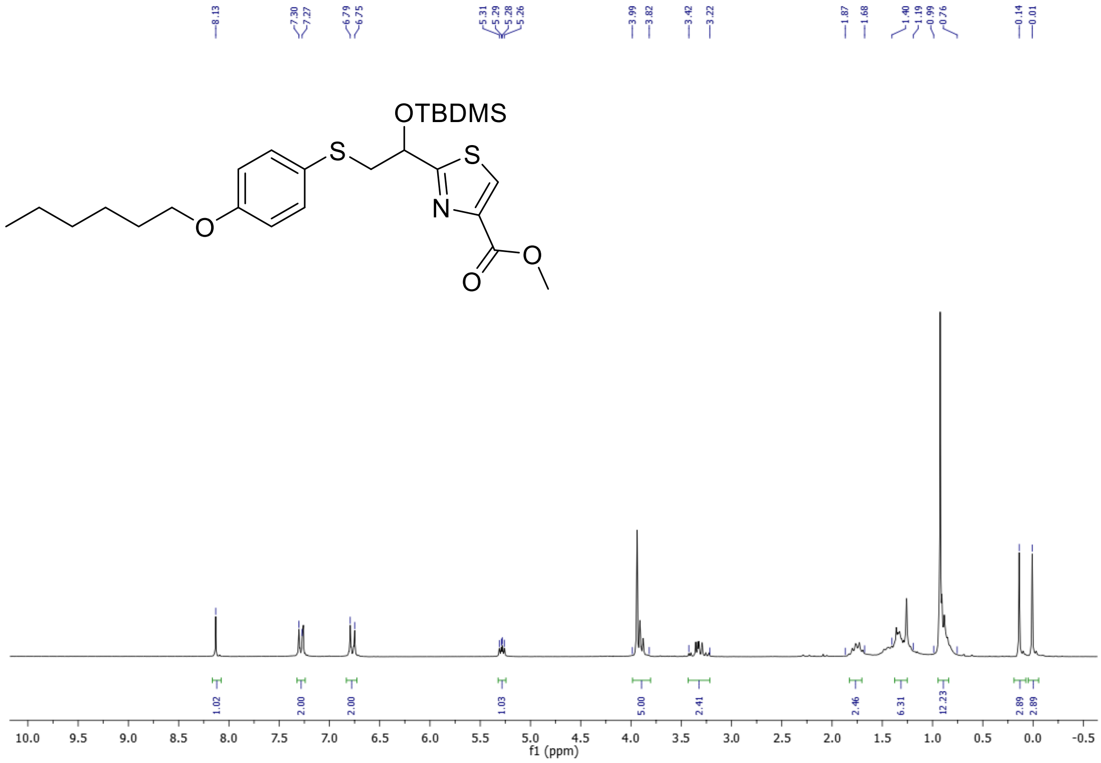

b. ^{13}C-NMR (50 MHz) of 15f in $CDCl_3$

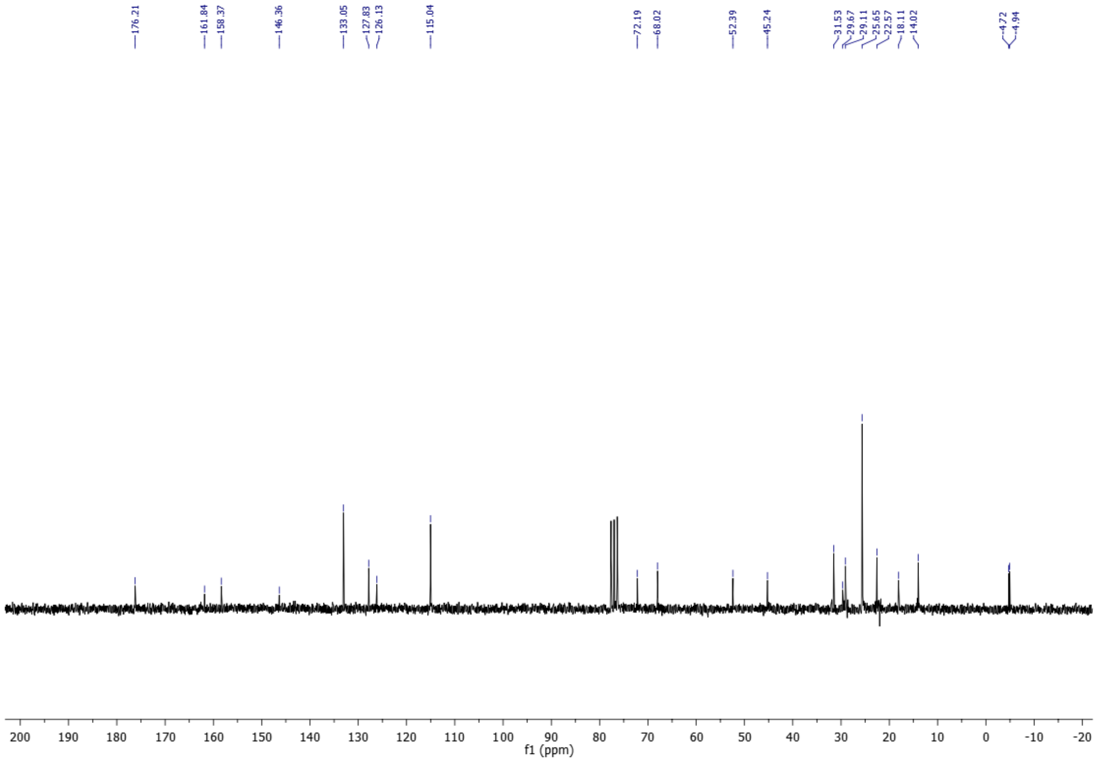

Figure S46. 15g

a. ^{1}H-NMR (200 MHz) of 15g in $CDCl_3$

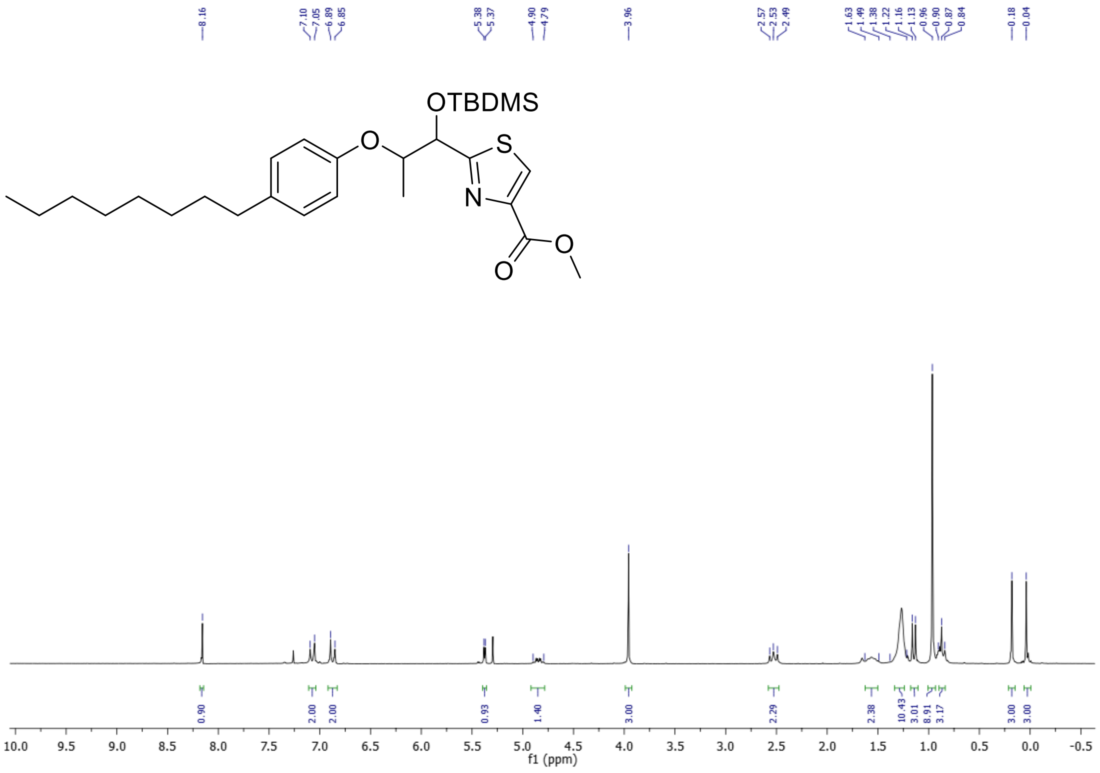

Figure S47. 15h

a. ^{1}H-NMR (400 MHz) of 15h in $CDCl_3$

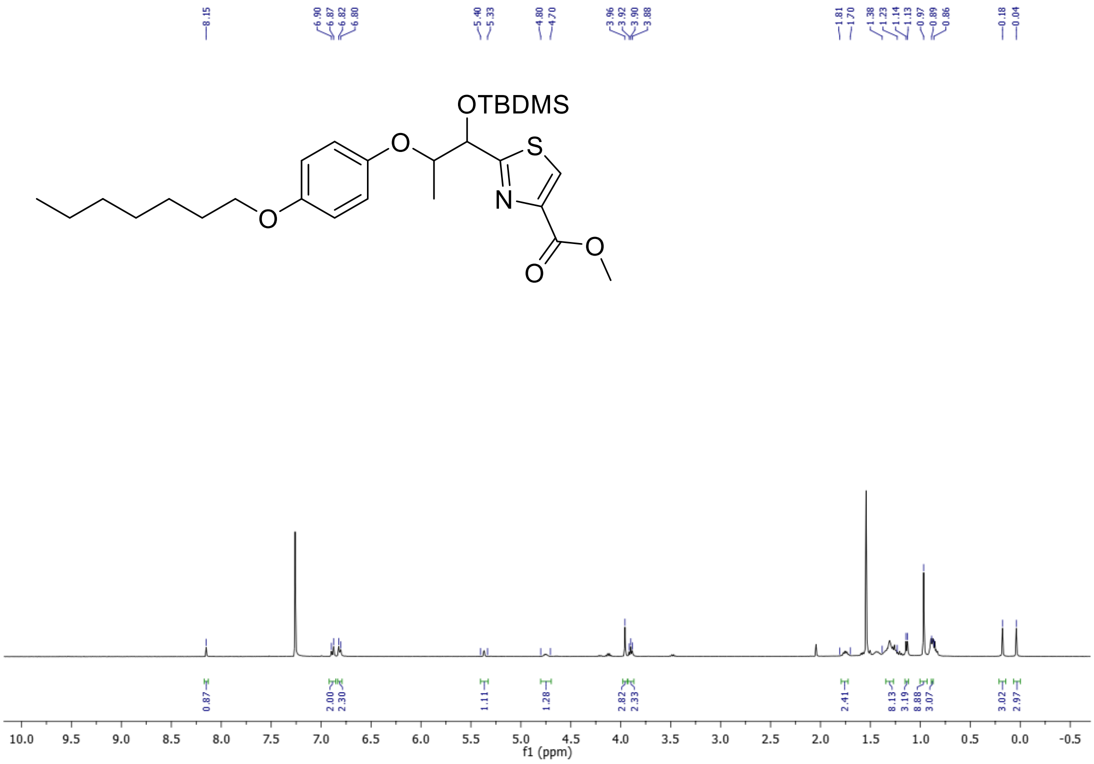

b. ^{13}C-NMR (100 MHz) of 15h in $CDCl_3$

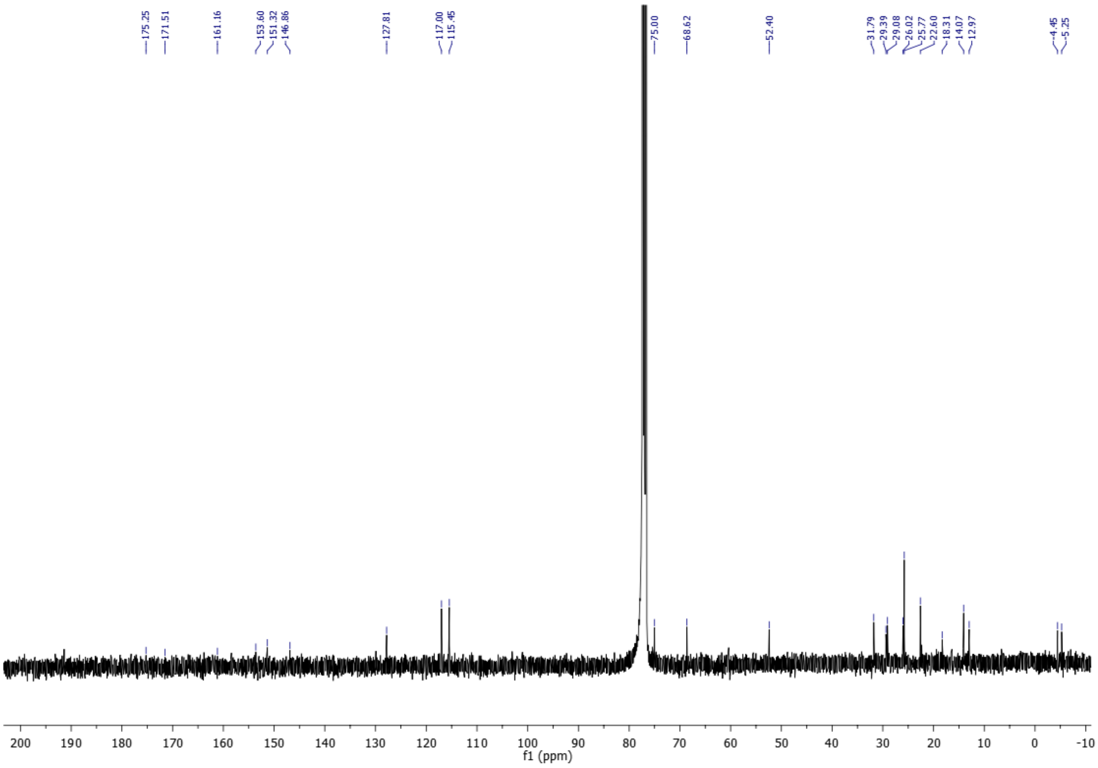

Figure S48. 15j

a. ^{1}H-NMR (200 MHz) of 15j in $CDCl_3$

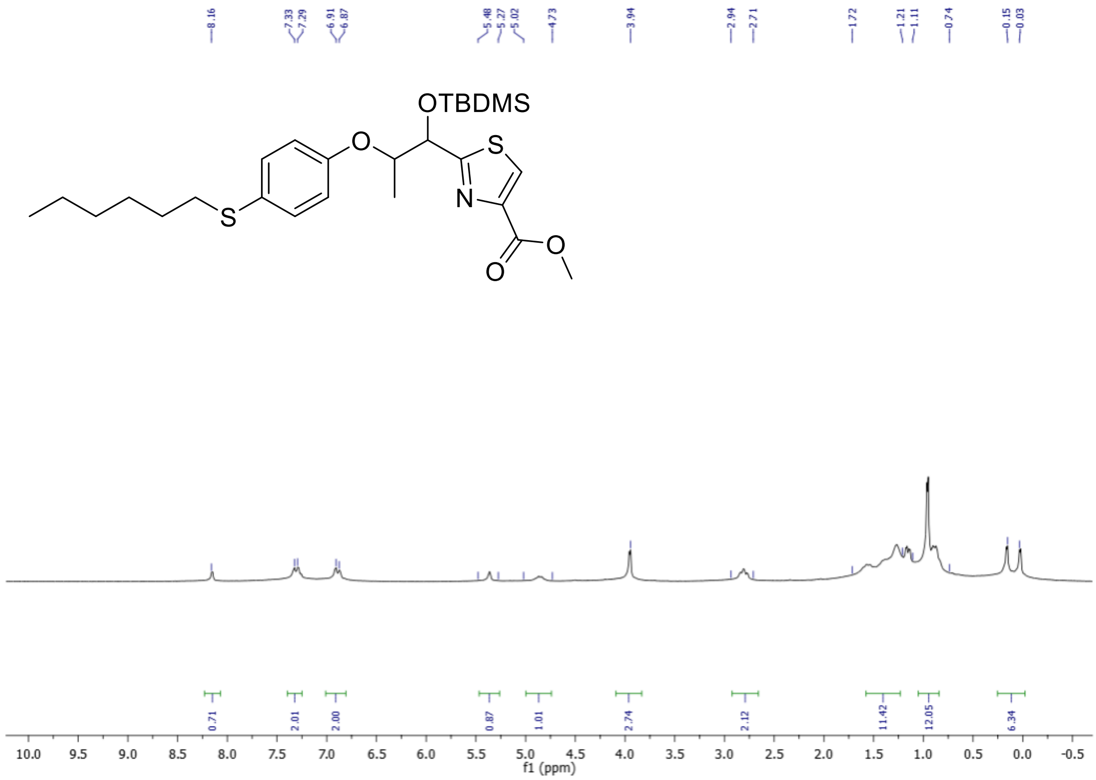

b. ^{13}C-NMR (50 MHz) of 15j in $CDCl_3$

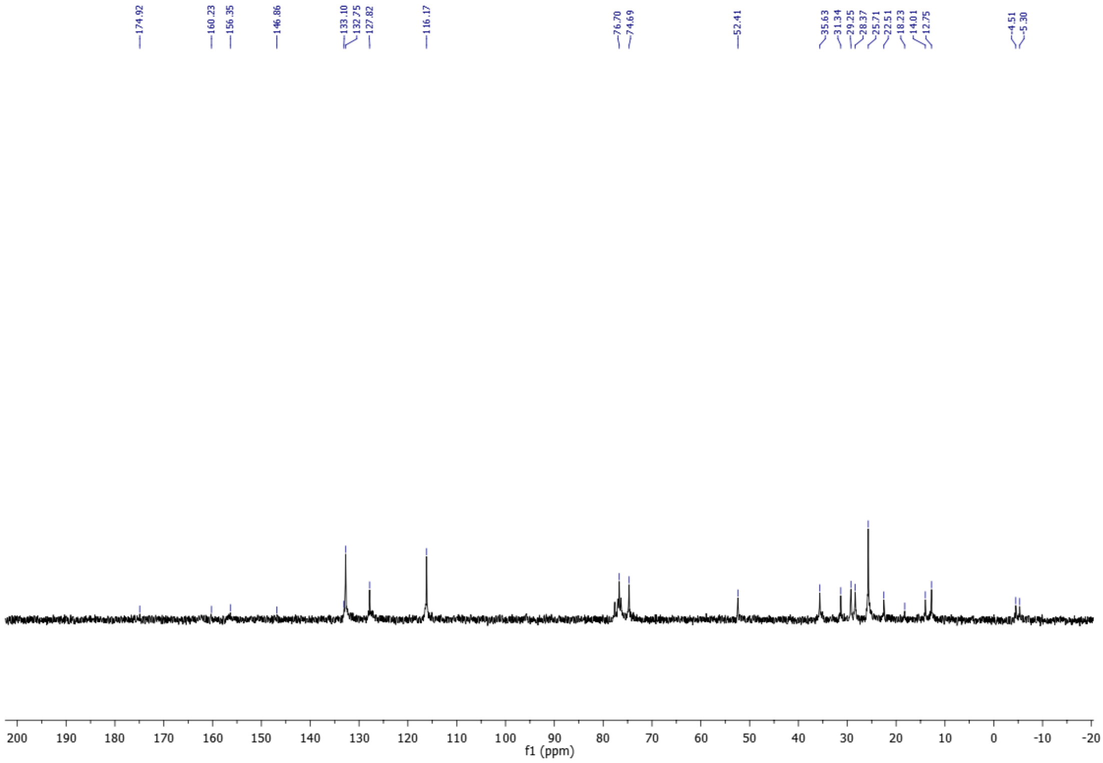

Figure S49. 16a

a. ^{1}H-NMR (200 MHz) of 16a in $CDCl_3$

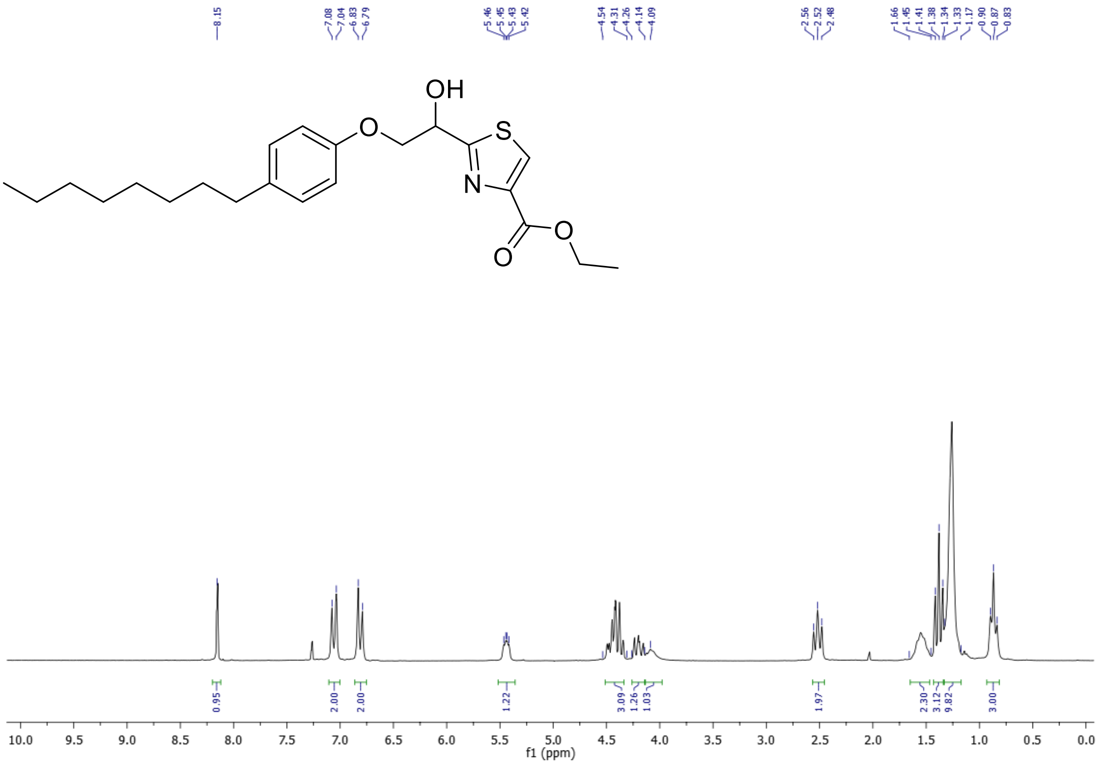

b. ^{13}C-NMR (50 MHz) of 16a in $CDCl_3$

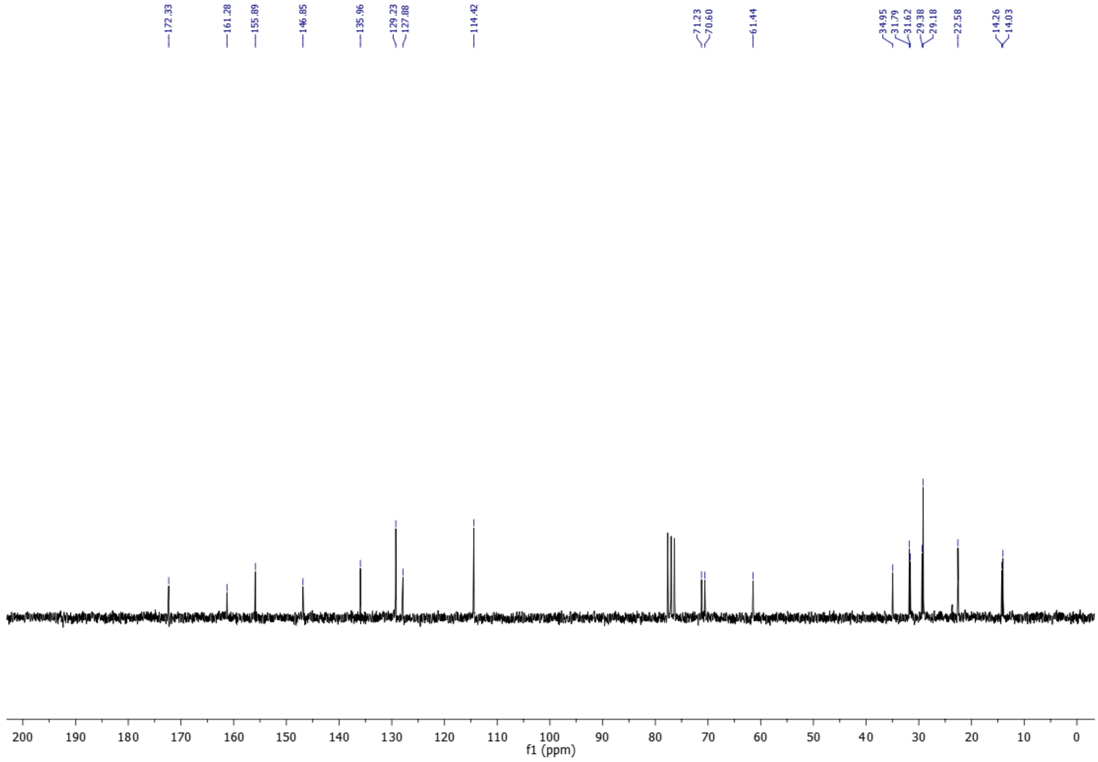

Figure S50. 16c

a. ^{1}H-NMR (400 MHz) of 16c in $CDCl_3$

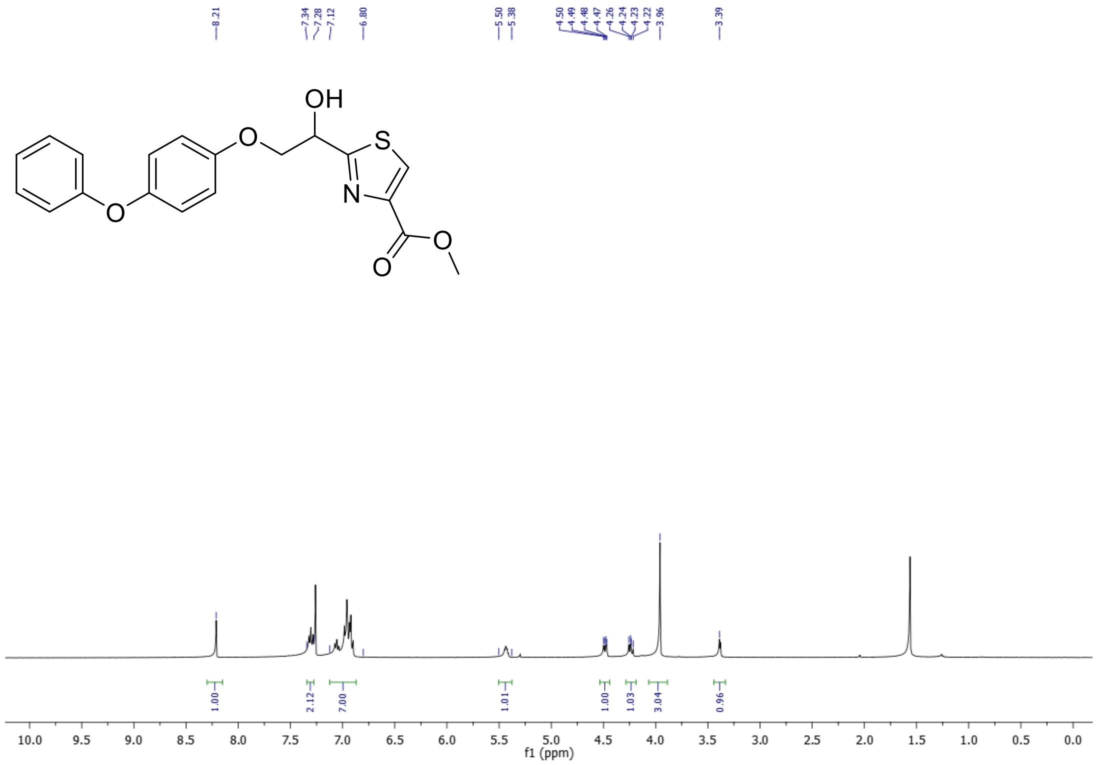

b. ^{13}C-NMR (100 MHz) of 16c in $CDCl_3$

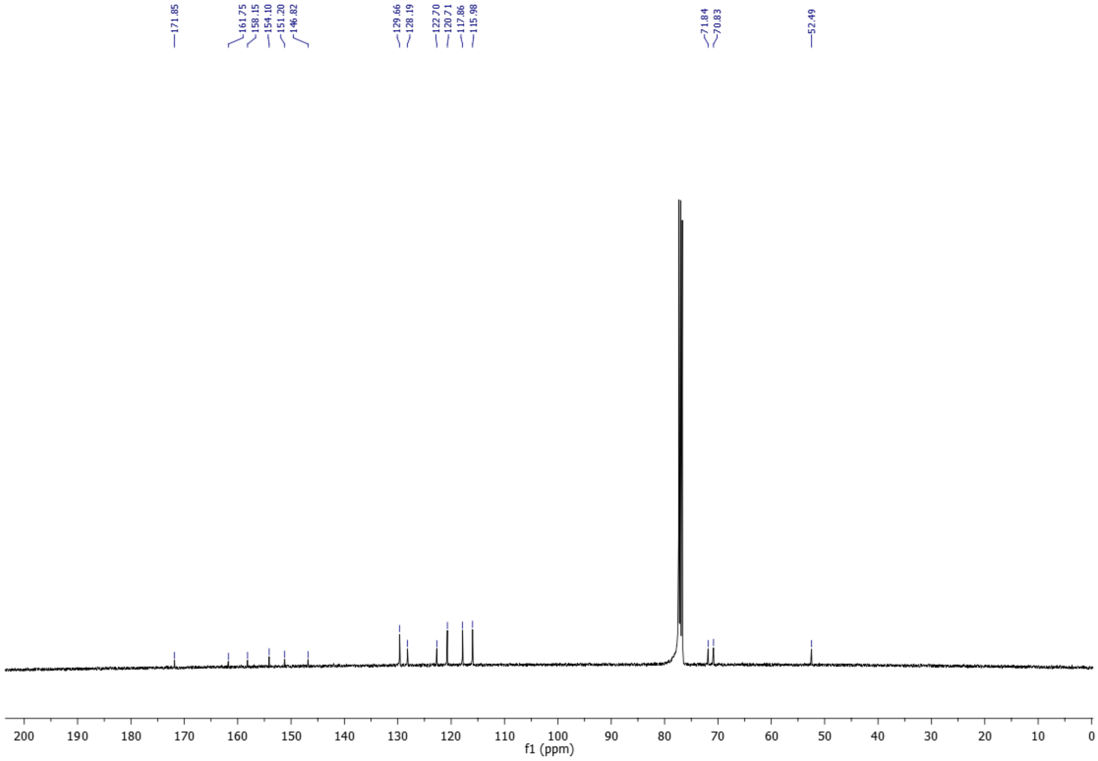

Figure S51. 16d

a. ^{1}H-NMR (200 MHz) of 16d in $CDCl_3$

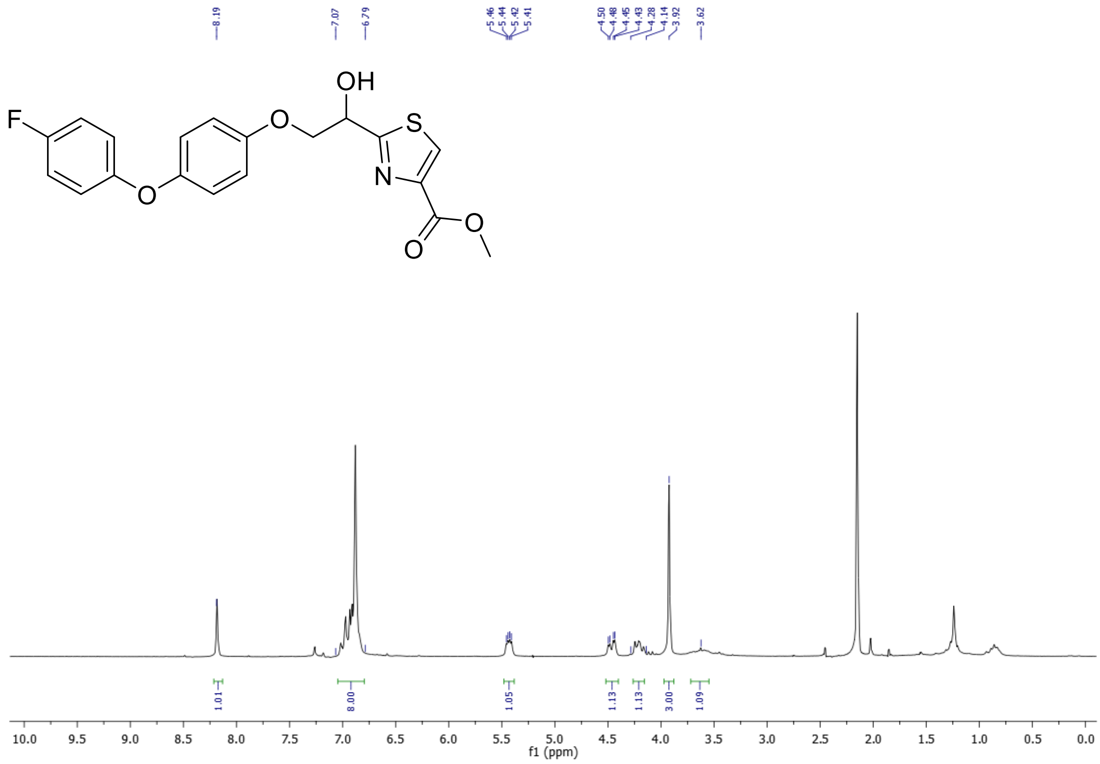

b. ^{13}C-NMR (50 MHz) of 16d in $CDCl_3$

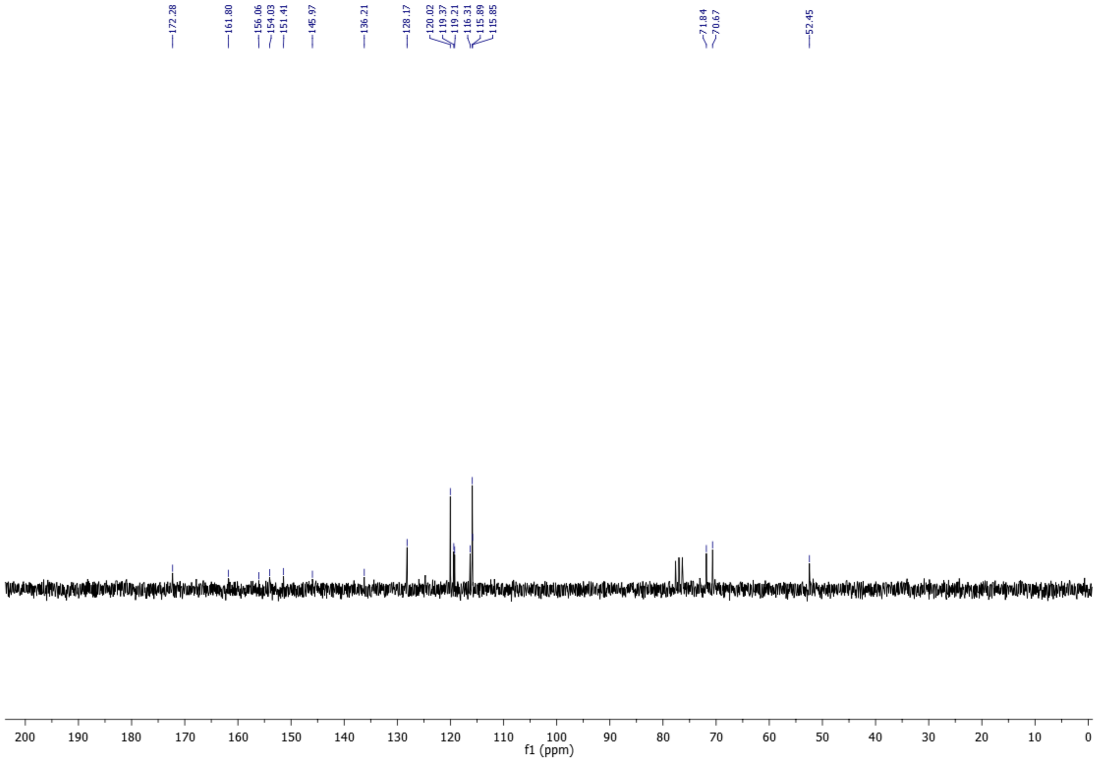

c. ^{19}F-NMR (377 MHz) of 16d in $CDCl_3$

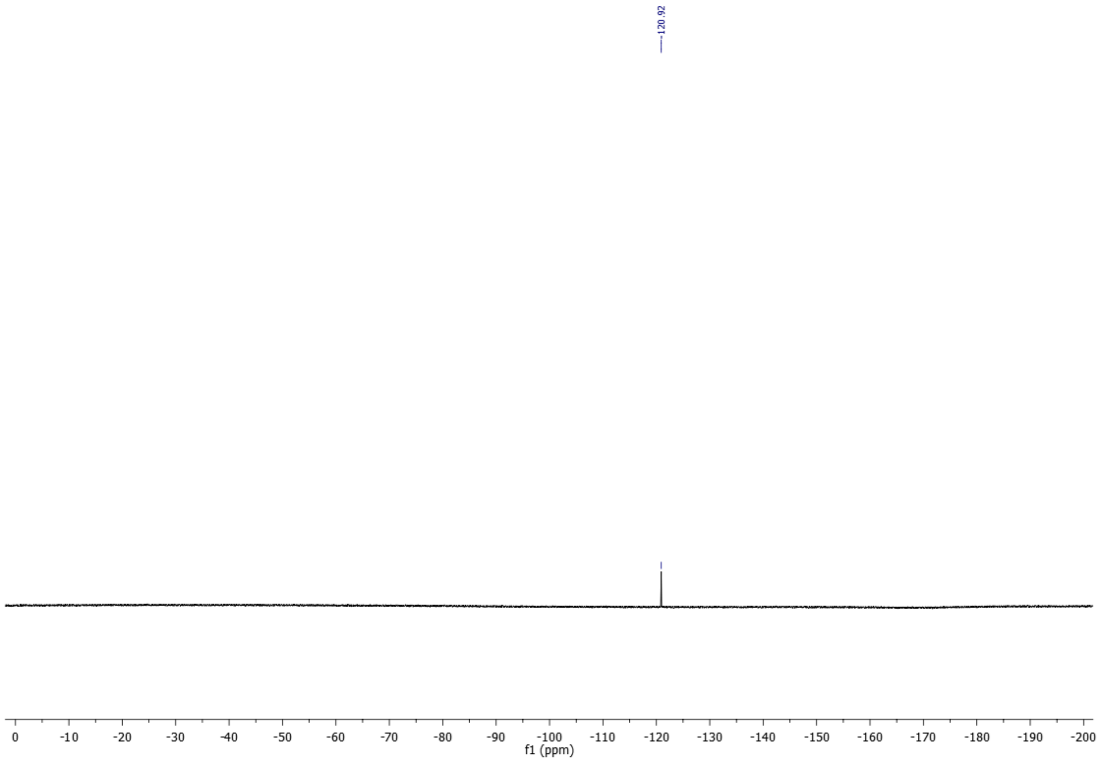

Figure S52. 16e

a. ^{1}H-NMR (200 MHz) of 16e in $CDCl_3$

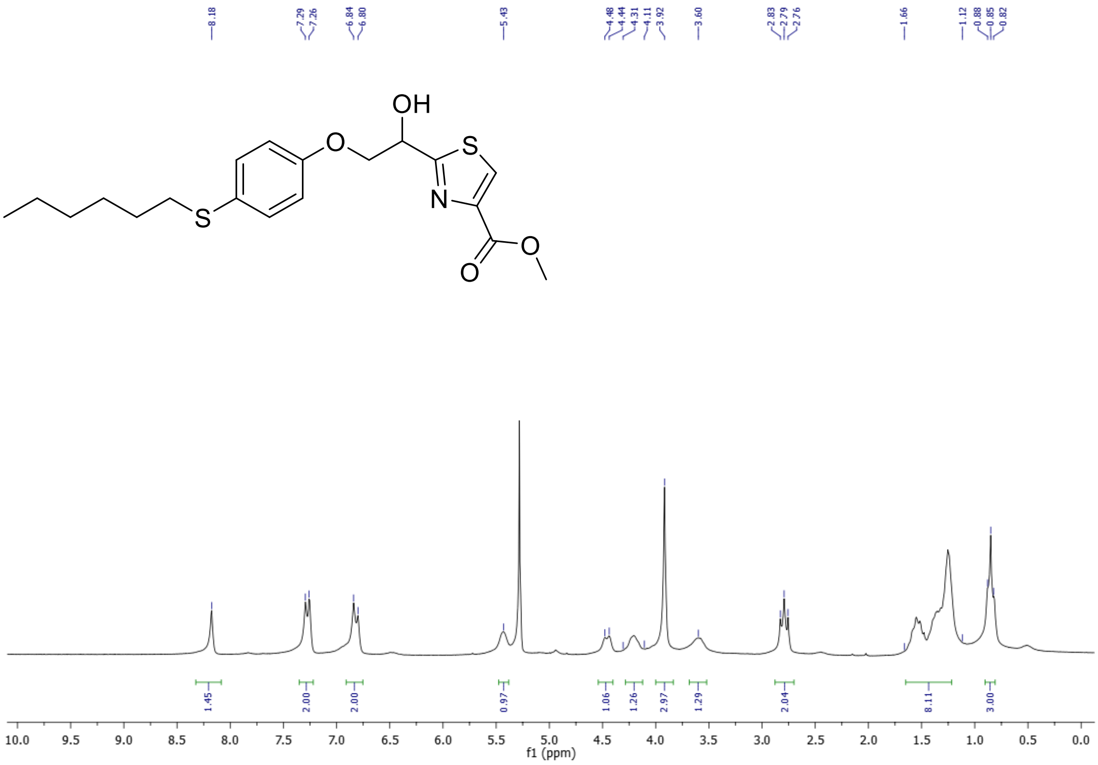

b. ^{13}C-NMR (50 MHz) of 16e in $CDCl_3$

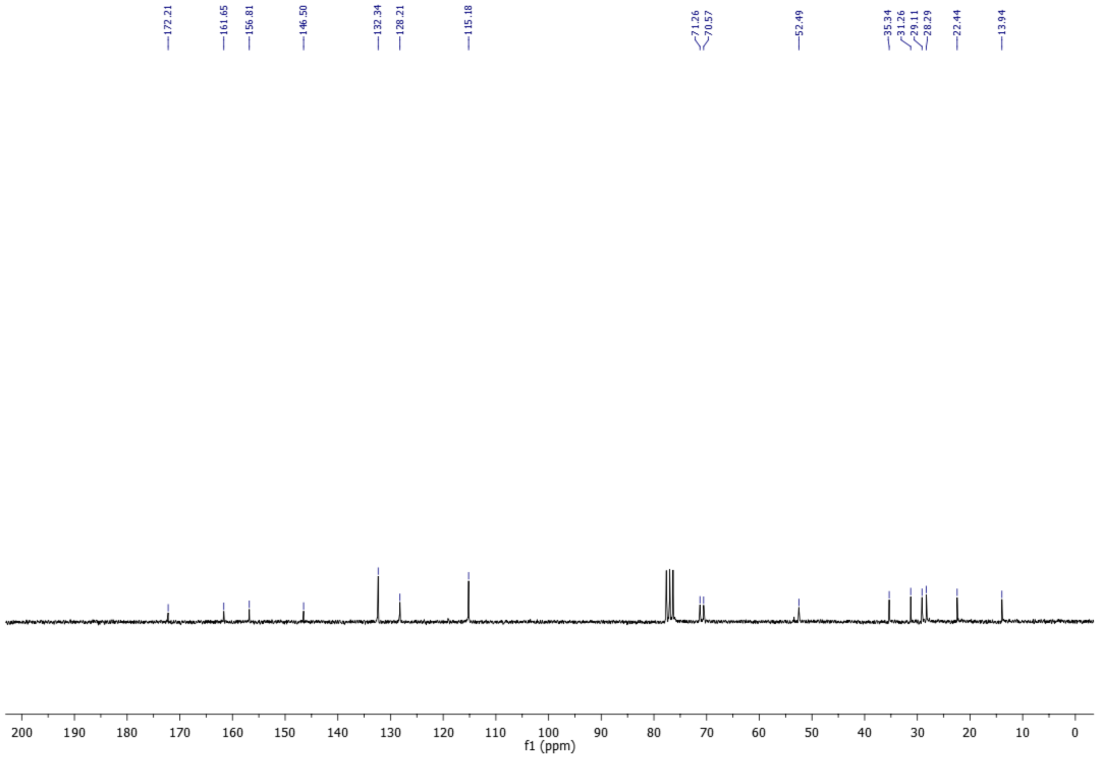

Figure S53. 16f

a. ^{1}H-NMR (200 MHz) of 16f in $CDCl_3$

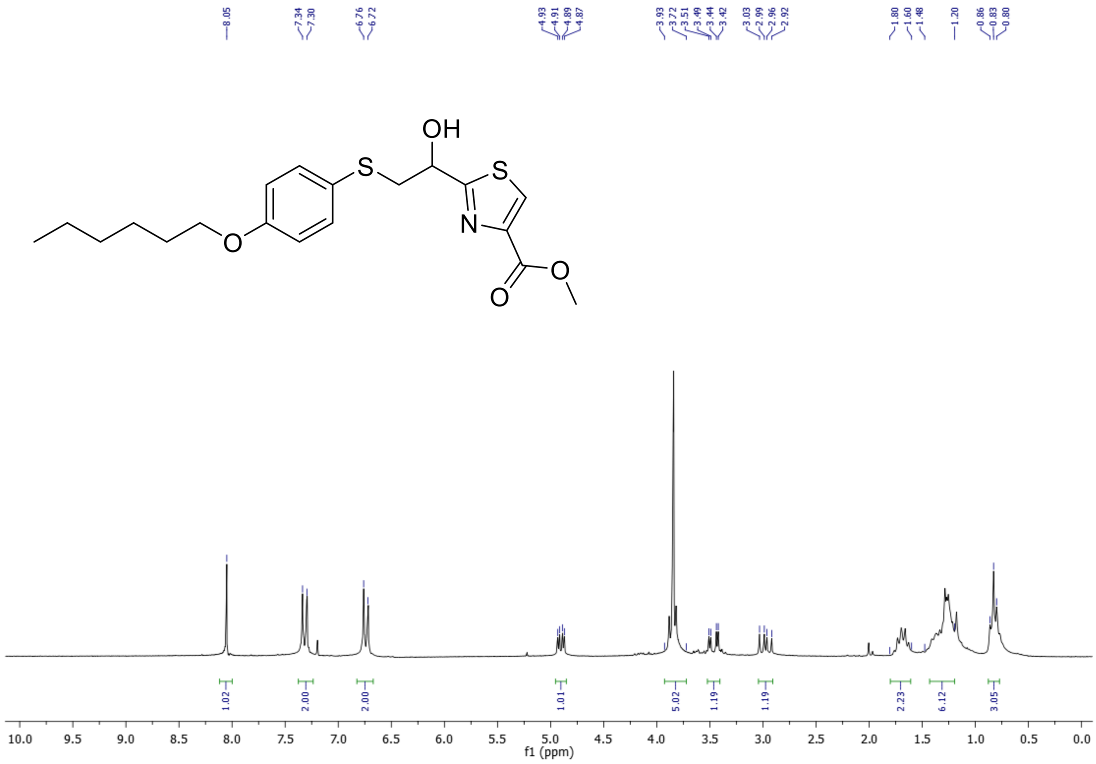

b. ^{13}C-NMR (100 MHz) of 16f in $CDCl_3$

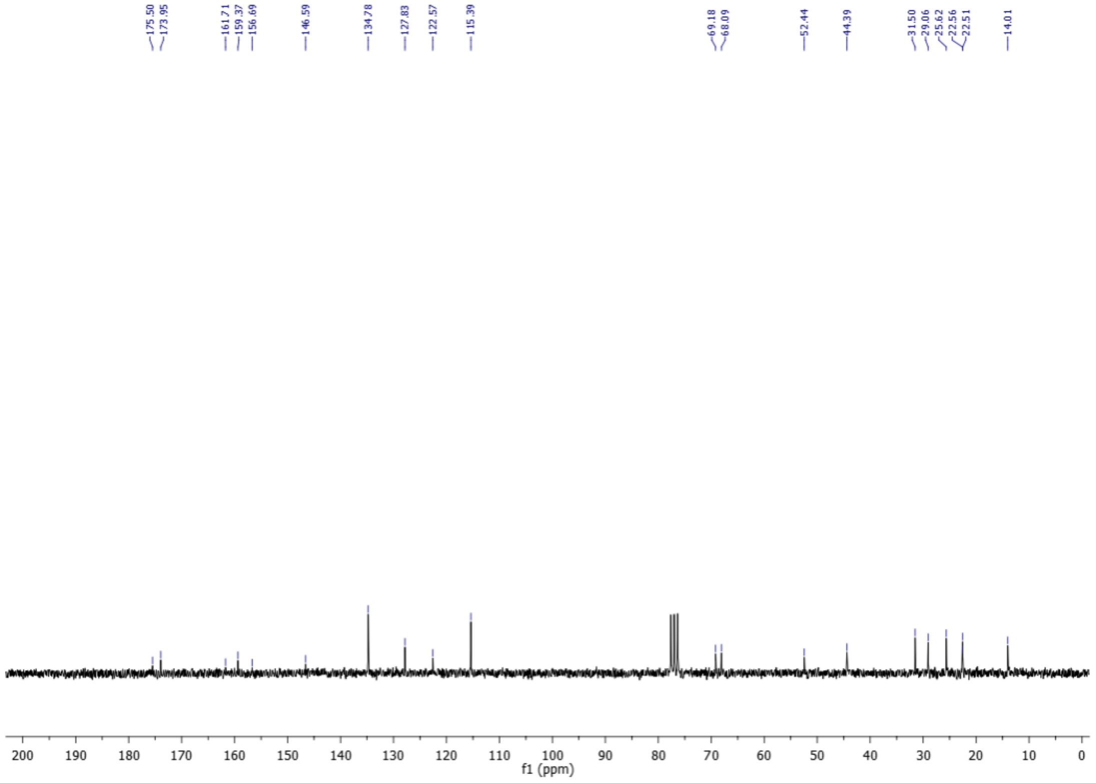

Figure S54. 16g

a. ^{1}H-NMR (400 MHz) of 16g in $CDCl_3$

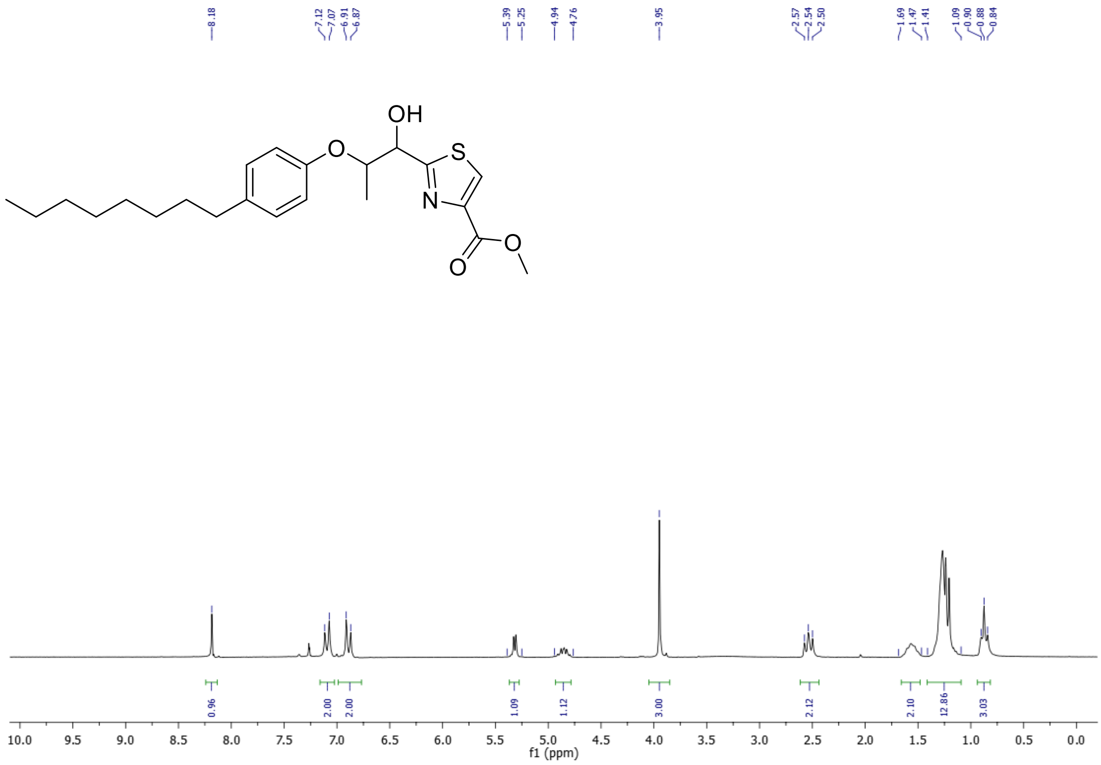

b. ^{13}C-NMR (100 MHz) of 16g in $CDCl_3$

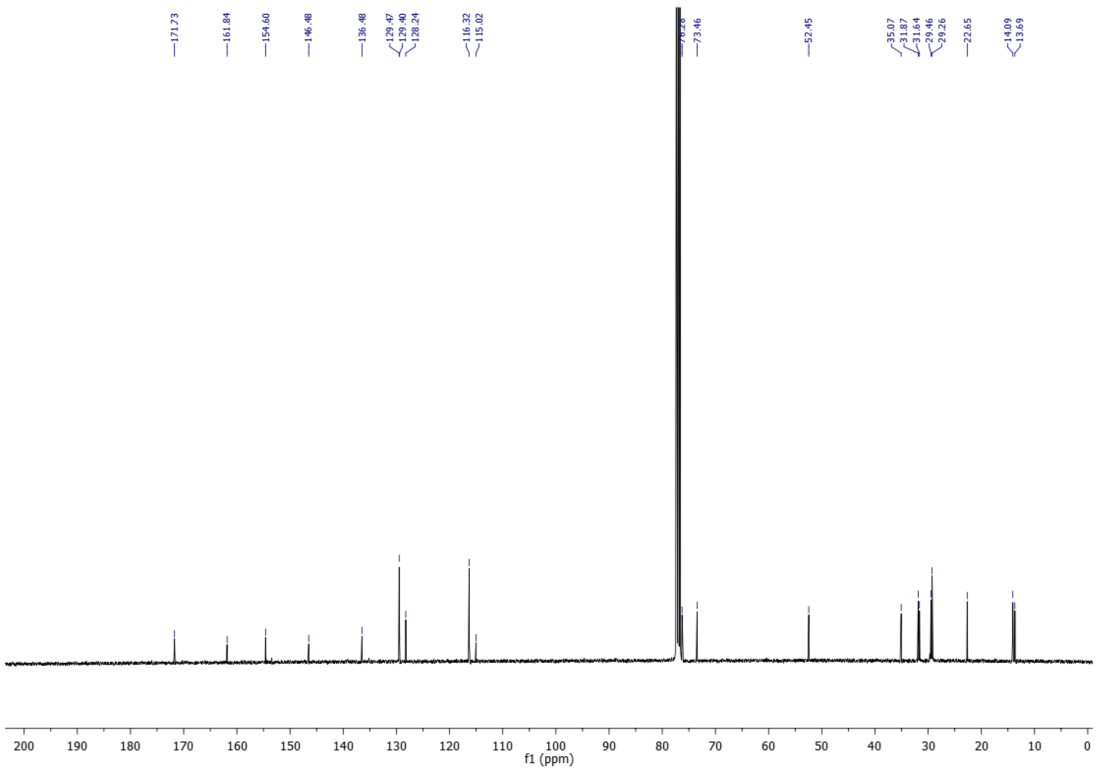

Figure S55. 16i

a. ^{1}H-NMR (400 MHz) of 16i in $CDCl_3$

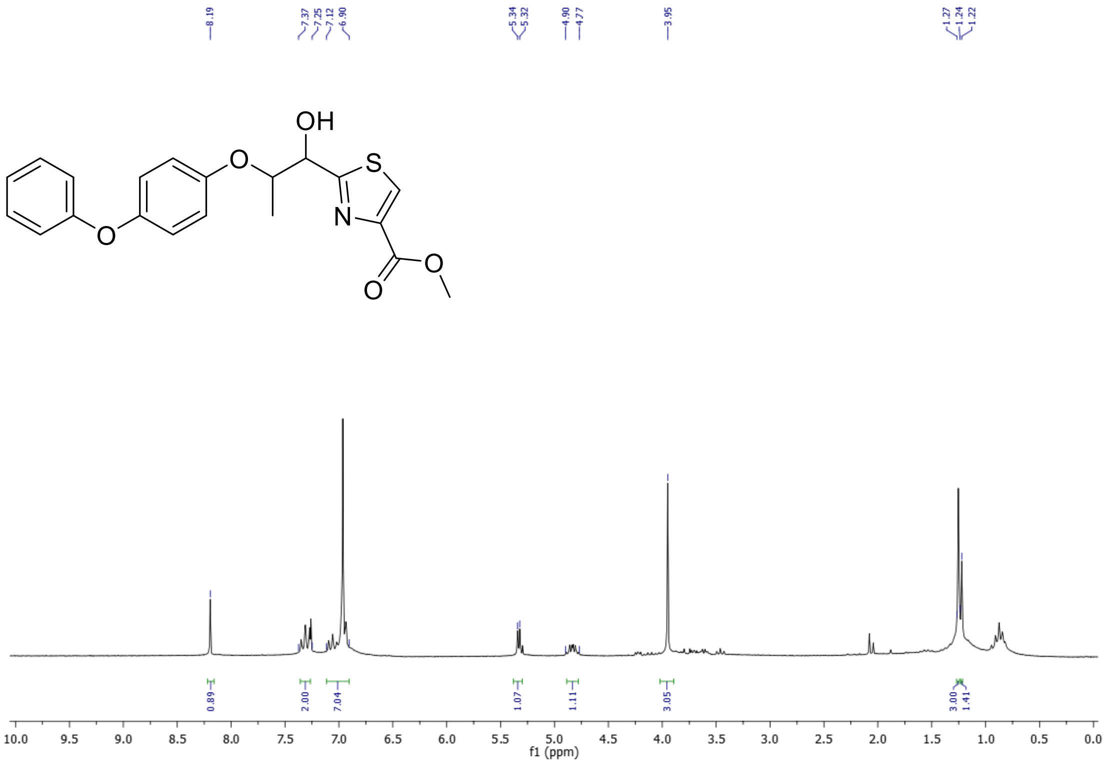

b. ^{13}C-NMR (100 MHz) of 16i in $CDCl_3$

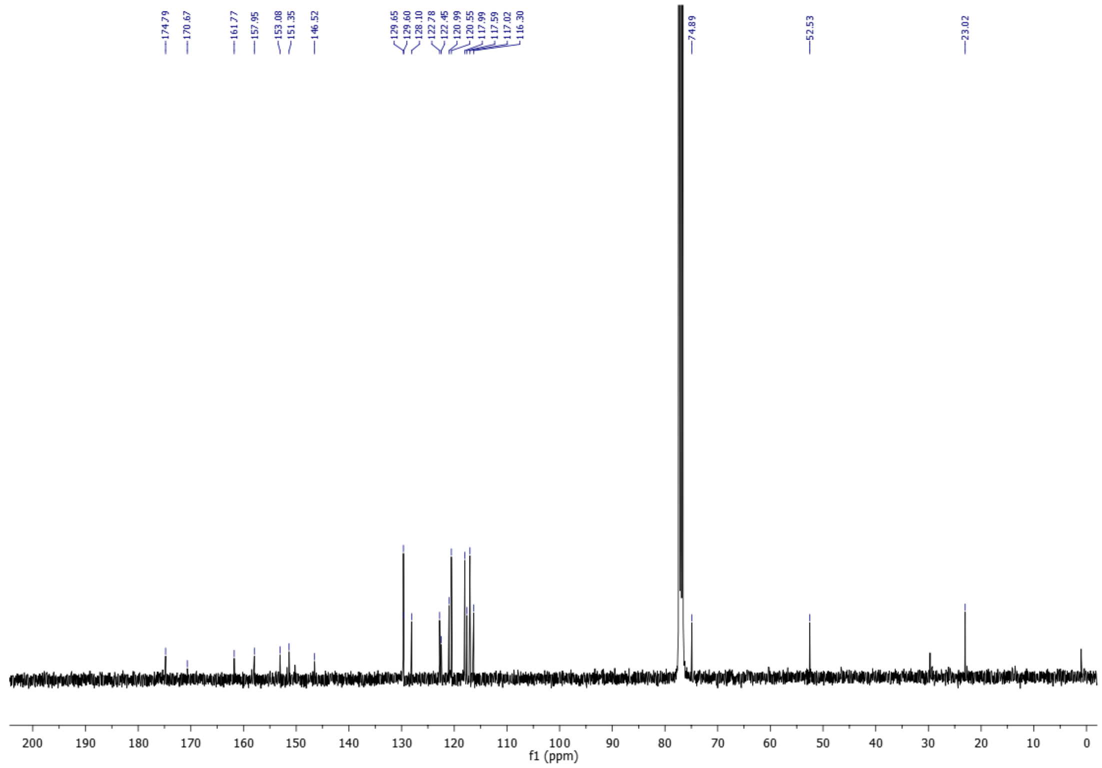

Figure S56. 16j

a. ^{1}H-NMR (200 MHz) of 16j in $CDCl_3$

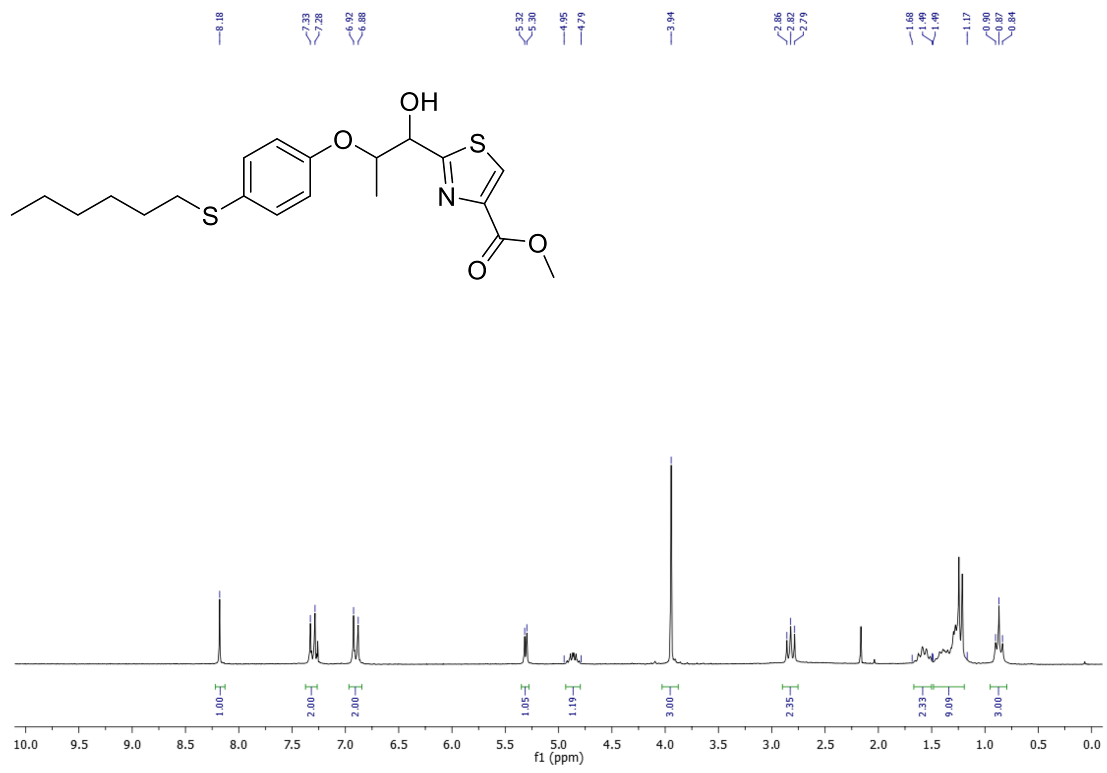

Figure S57. 17a

a. ^{1}H-NMR (200 MHz) of 17a in $CDCl_3$

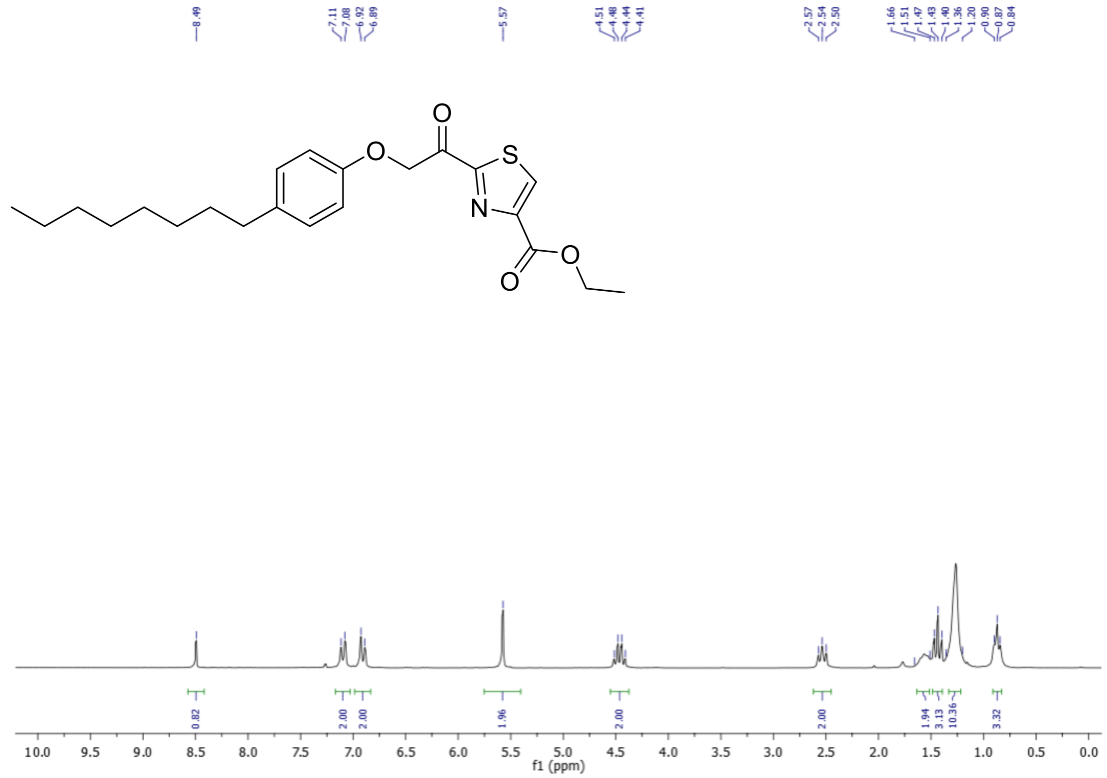

b. ^{13}C-NMR (50 MHz) of 17a in $CDCl_3$

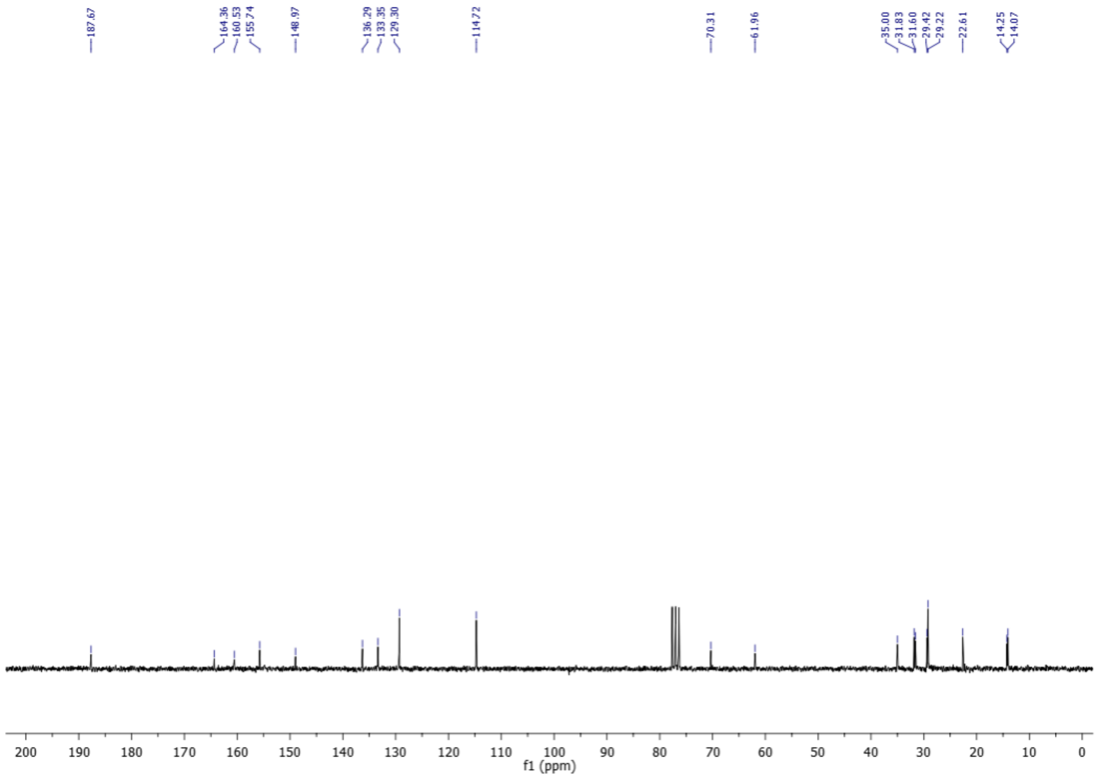

Figure S58. 17b

a. ^{1}H-NMR (200 MHz) of 17b in $CDCl_3$

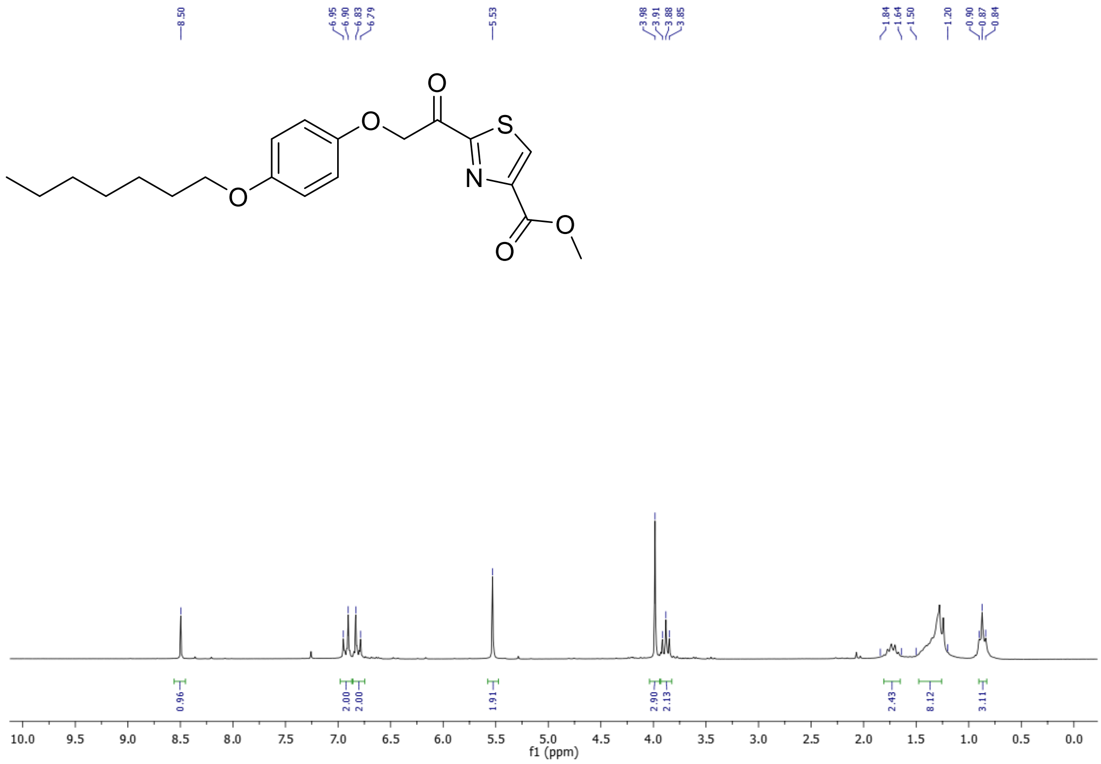

b. ^{13}C-NMR (50 MHz) of 17b in $CDCl_3$

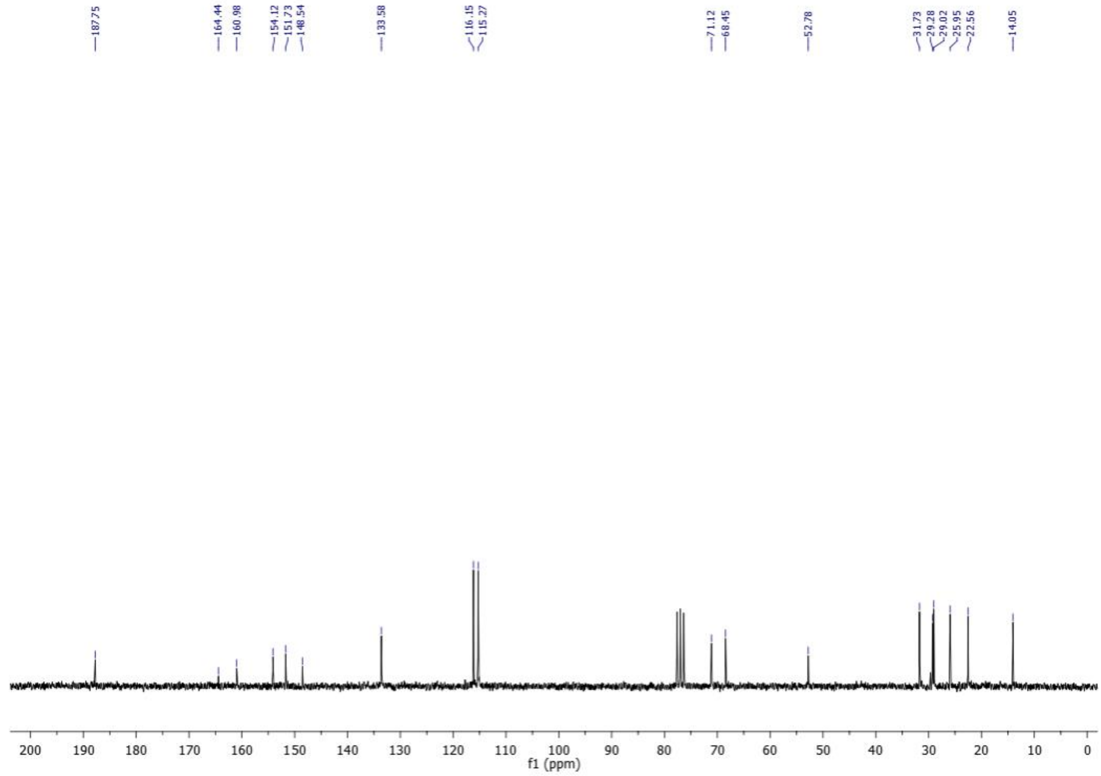

Figure S59. 17c

a. ^{1}H-NMR (200 MHz) of 17c in $CDCl_3$

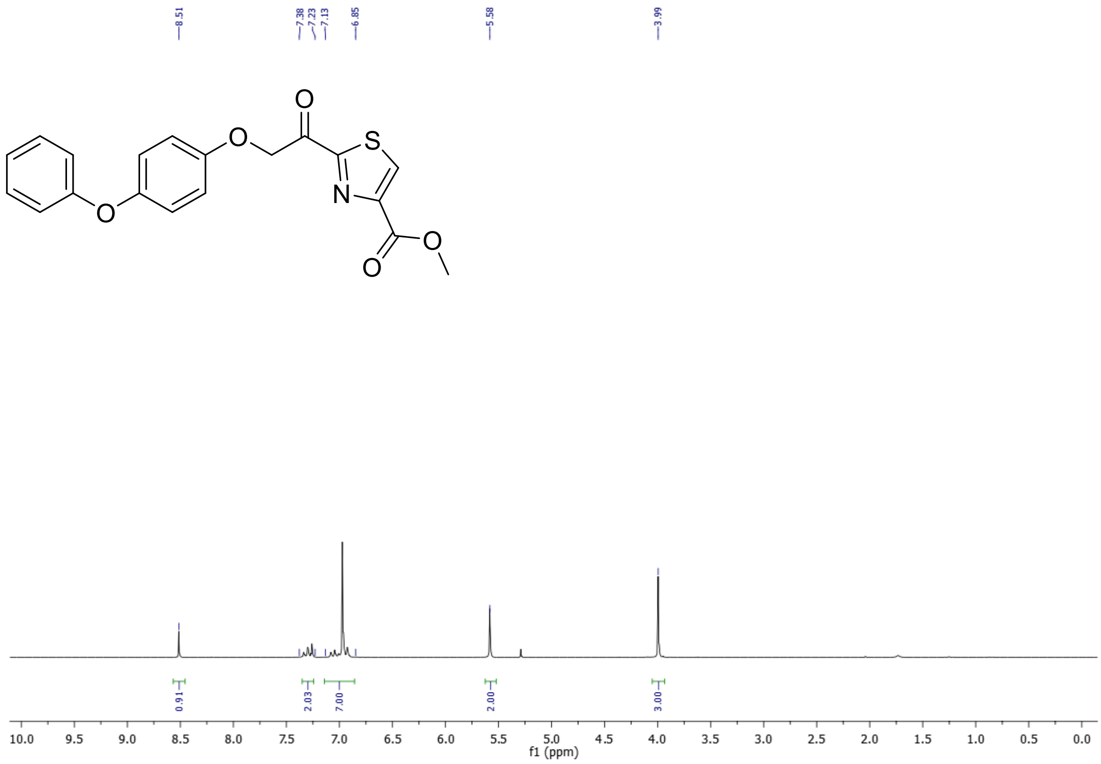

b. ^{13}C-NMR (100 MHz) of 17c in $CDCl_3$

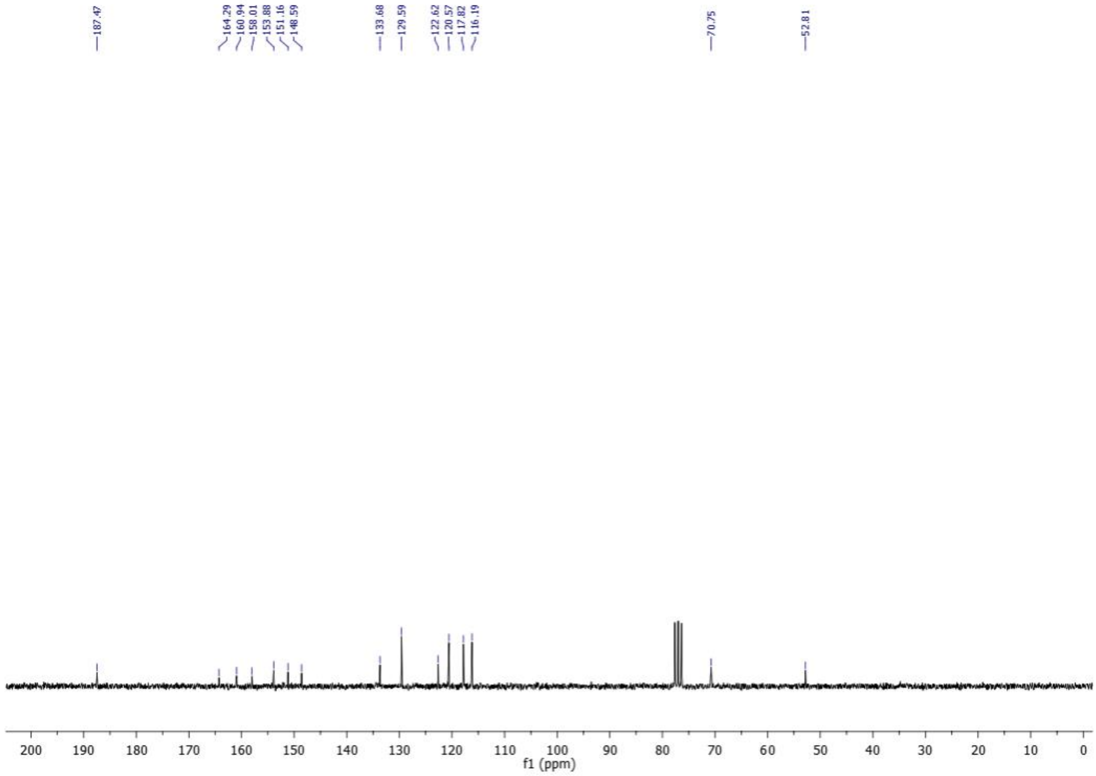

Figure S60. 17d

a. ^{1}H-NMR (600 MHz) of 17d in $CDCl_3$

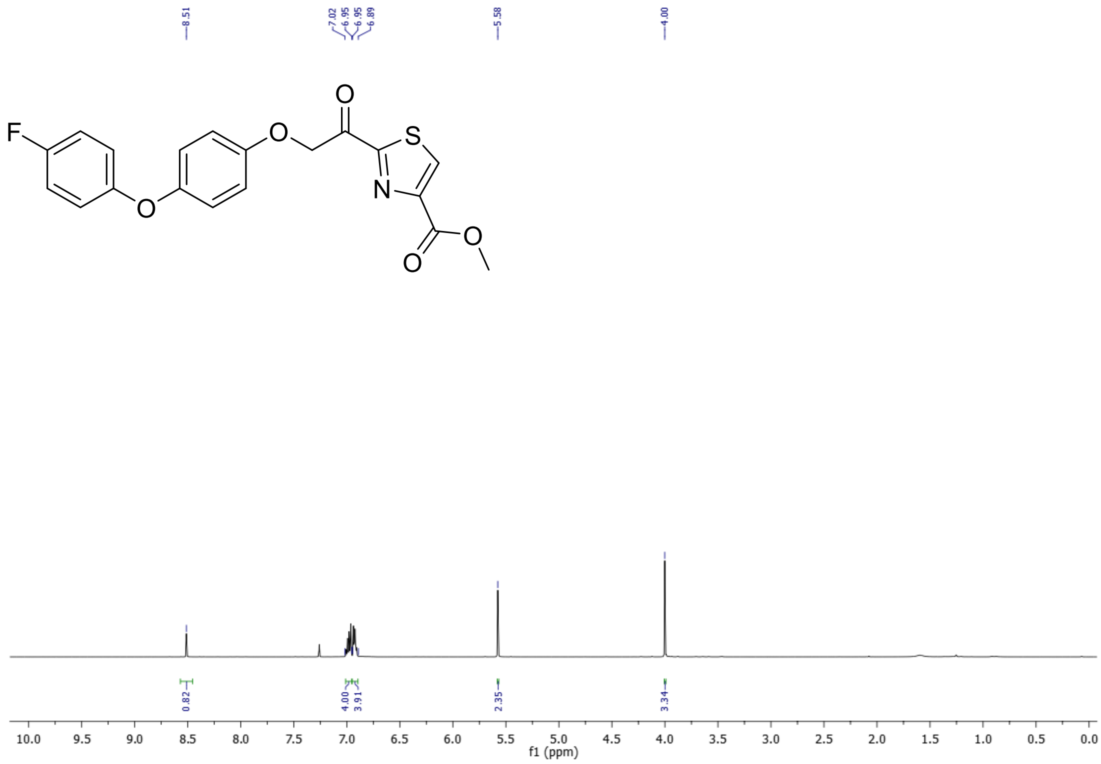

b. ^{13}C-NMR (150 MHz) of 17d in $CDCl_3$

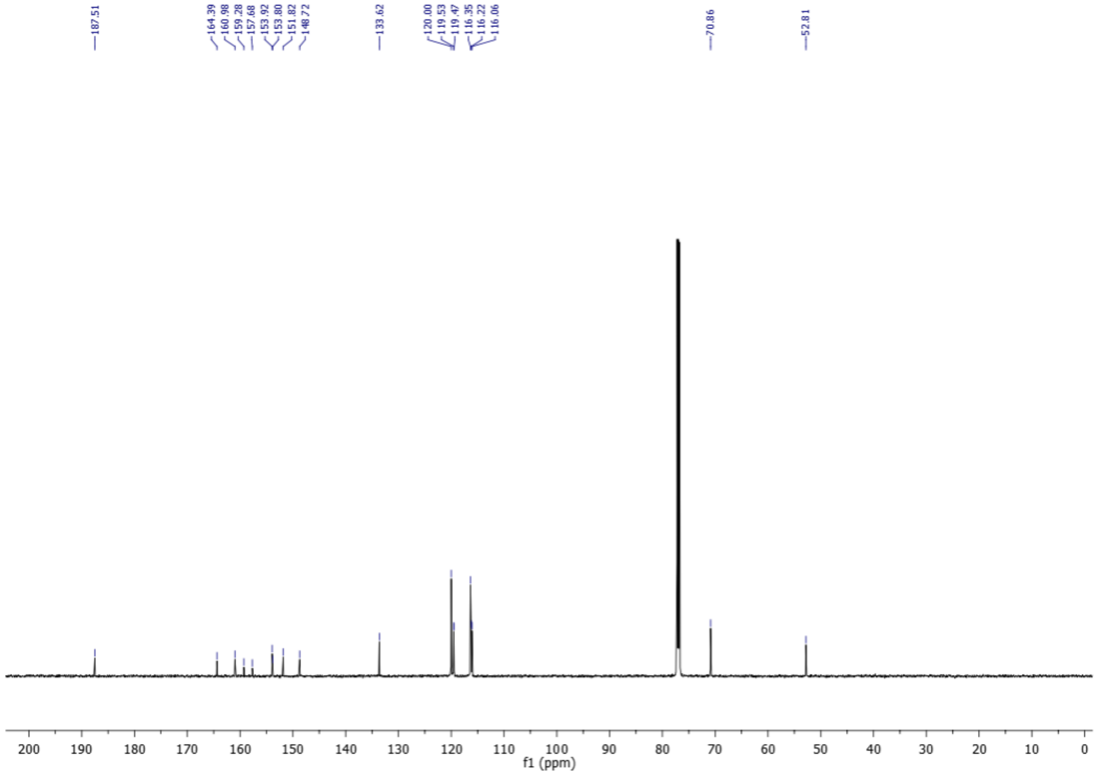

Figure S61. 17e

a. ^{1}H-NMR (200 MHz) of 17e in $CDCl_3$

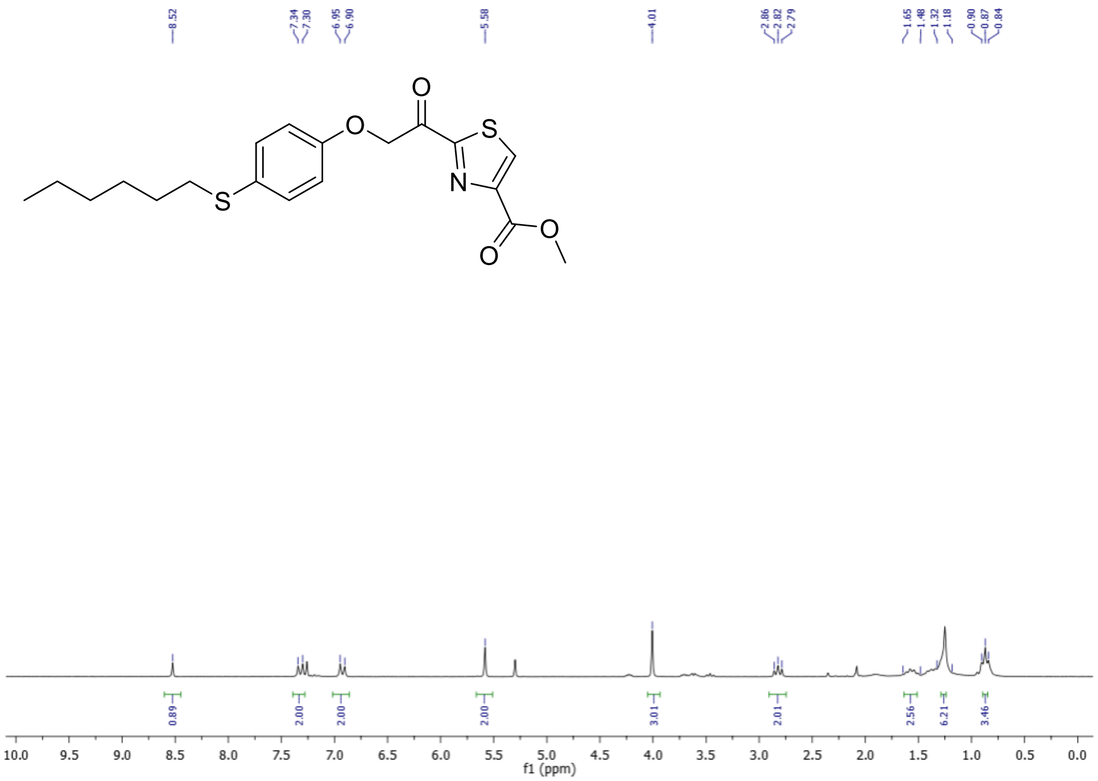

b. ^{13}C-NMR (50 MHz) of 17e in $CDCl_3$

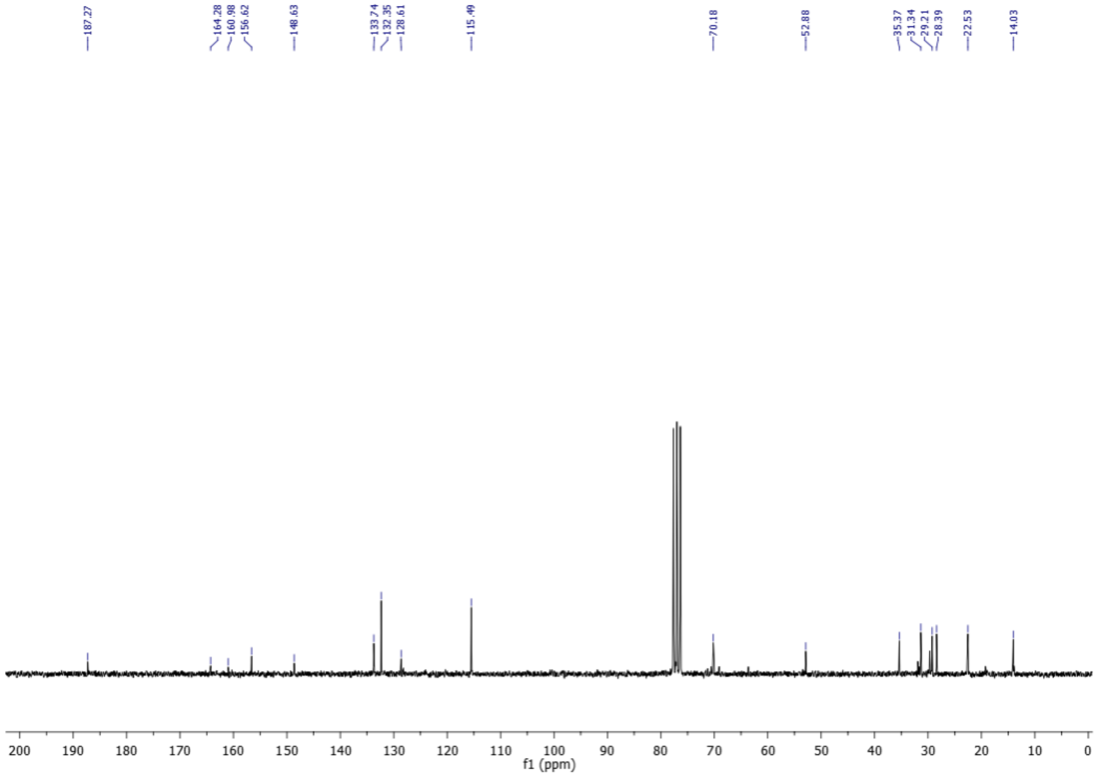

Figure S62. 17f

a. ^{1}H-NMR (200 MHz) of 17f in $CDCl_3$

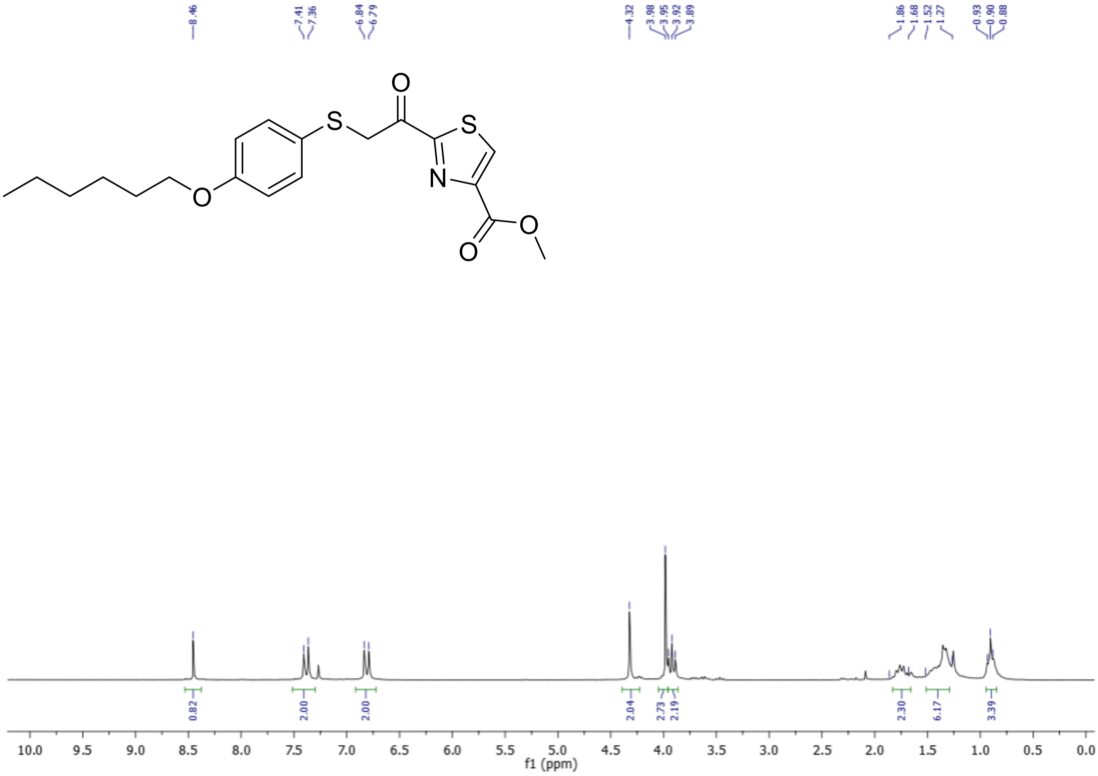

b. ^{13}C-NMR (50 MHz) of 17f in $CDCl_3$

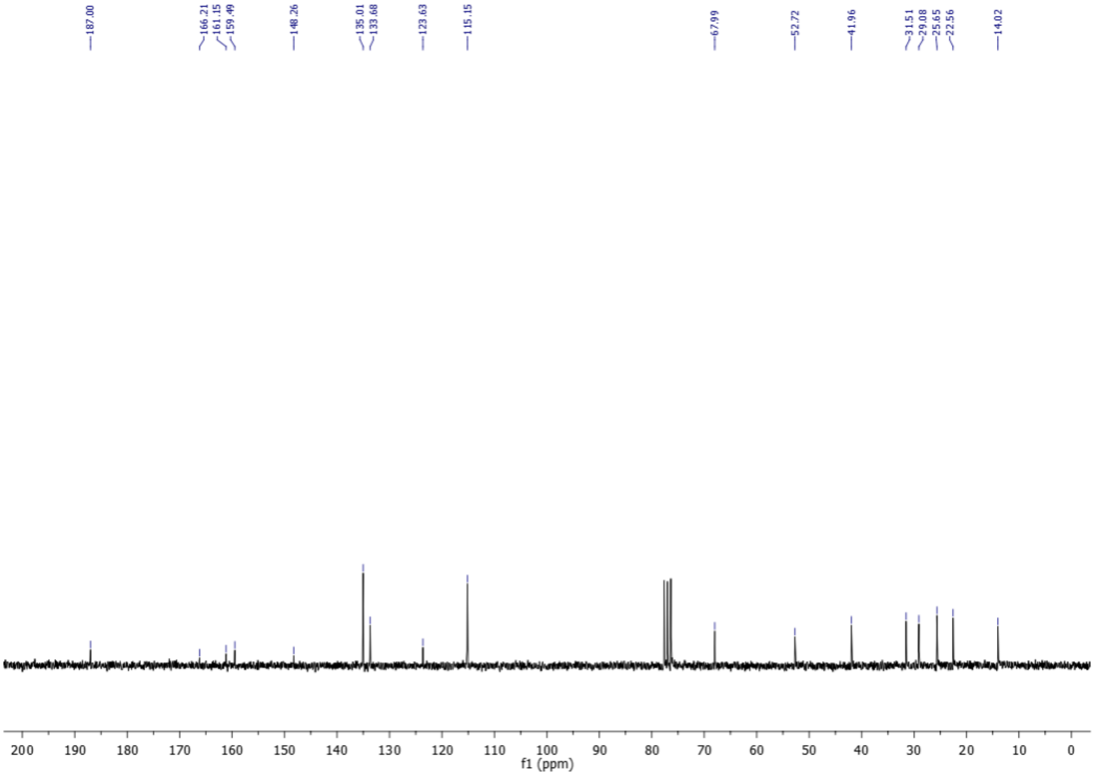

Figure S63. 17g

a. ^{1}H-NMR (200 MHz) of 17g in $CDCl_3$

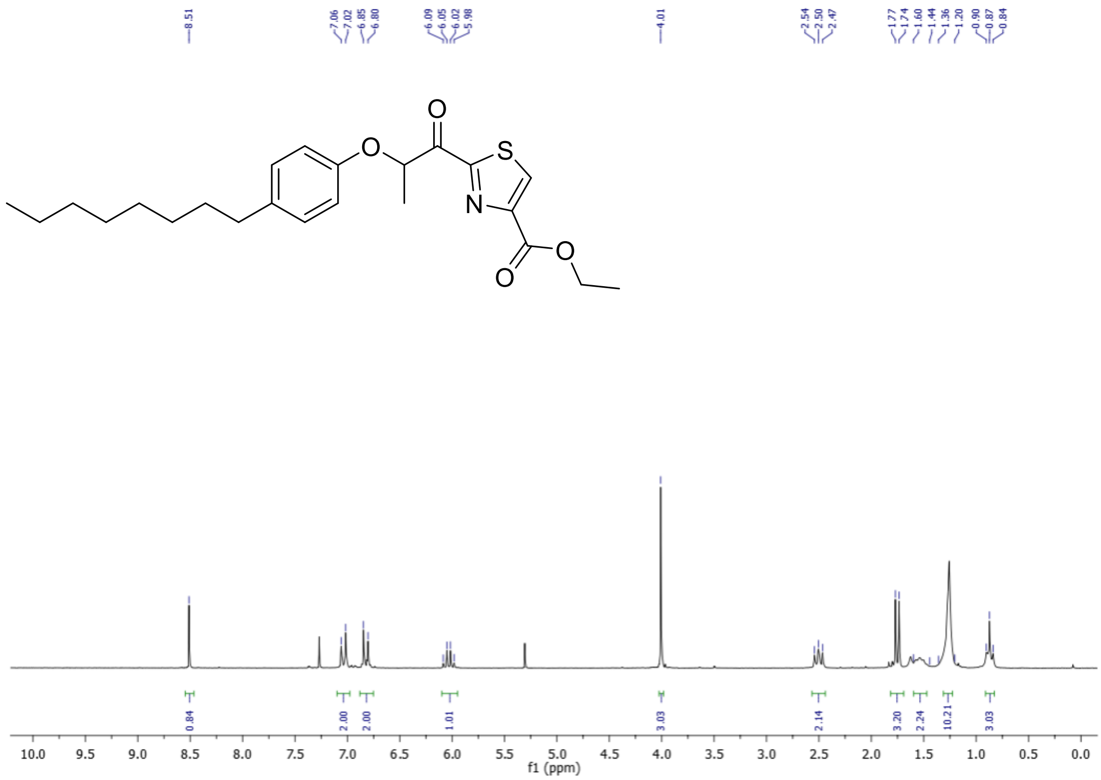

b. ^{13}C-NMR (50 MHz) of 17g in $CDCl_3$

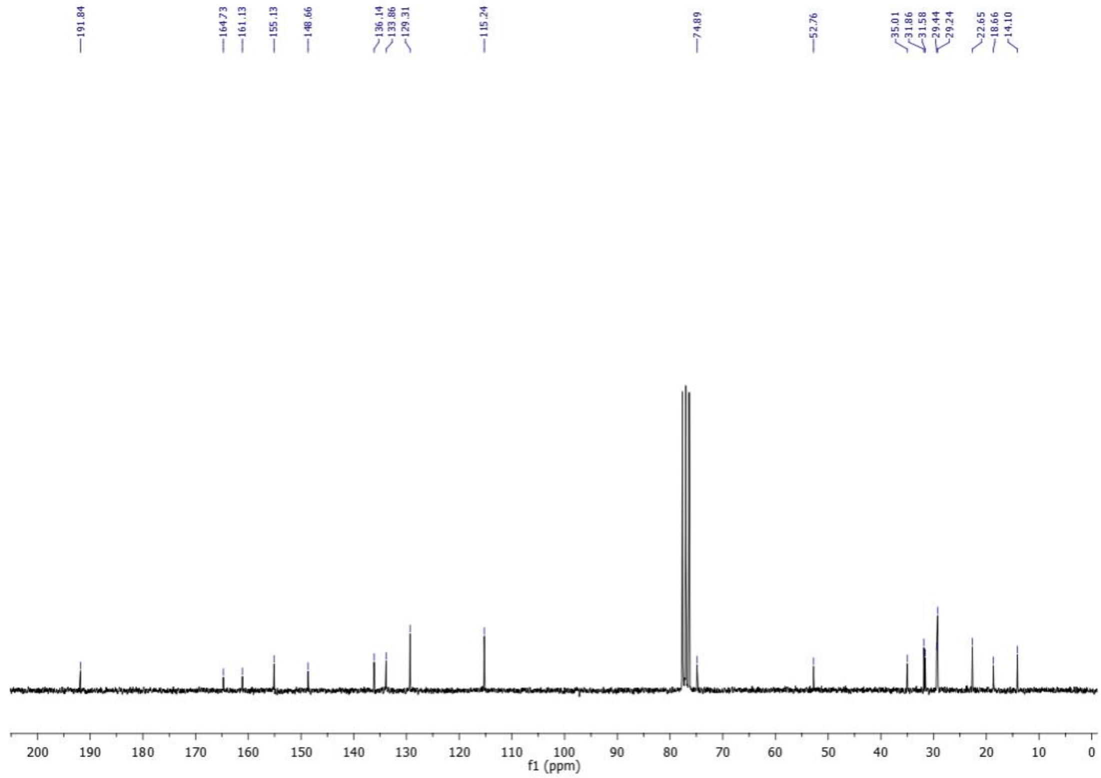

Figure S64. 17h

a. ^{1}H-NMR (600 MHz) of 17h in $CDCl_3$

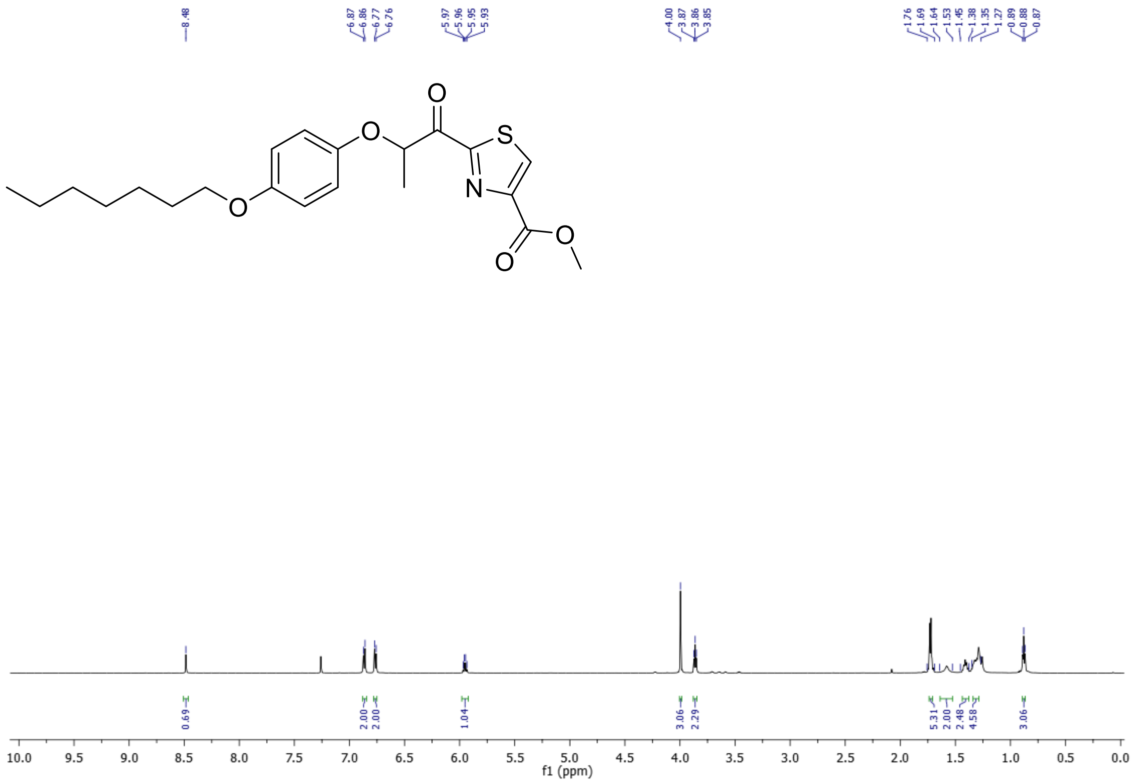

b. ^{13}C-NMR (150 MHz) of 17h in $CDCl_3$

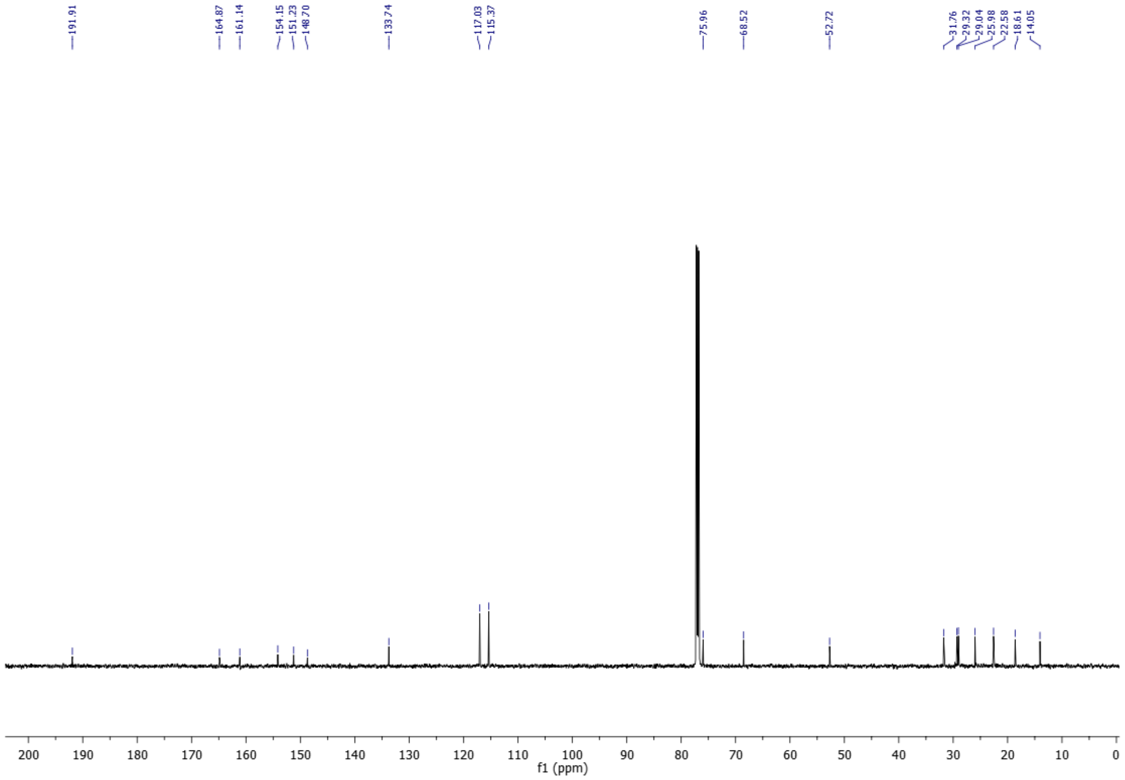

Figure S65. 17i

a. ^{1}H-NMR (600 MHz) of 17i in $CDCl_3$

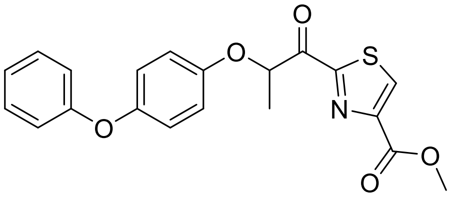

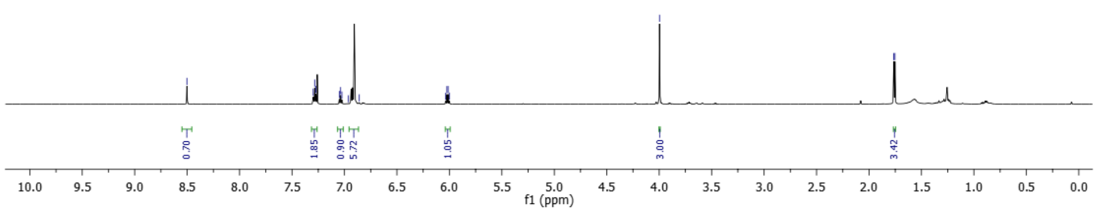

b. ^{13}C-NMR (150 MHz) of 17i in $CDCl_3$

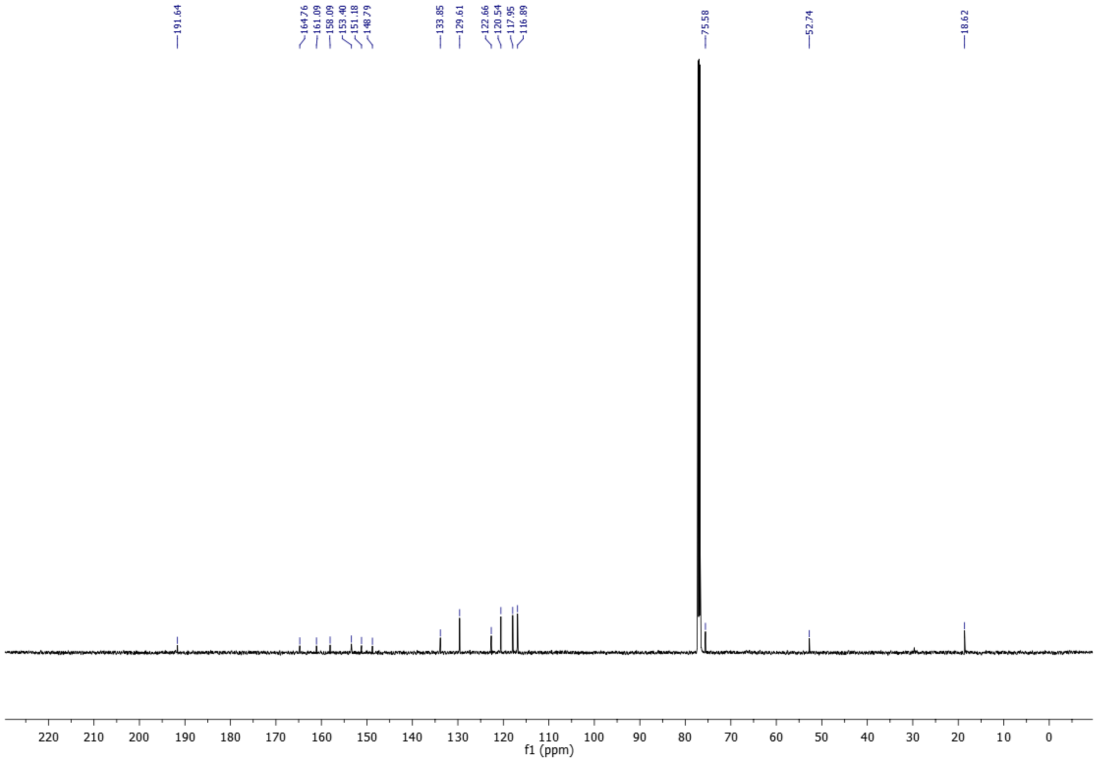

Figure S66. 17j

a. ^{1}H-NMR (600 MHz) of 17j in $CDCl_3$

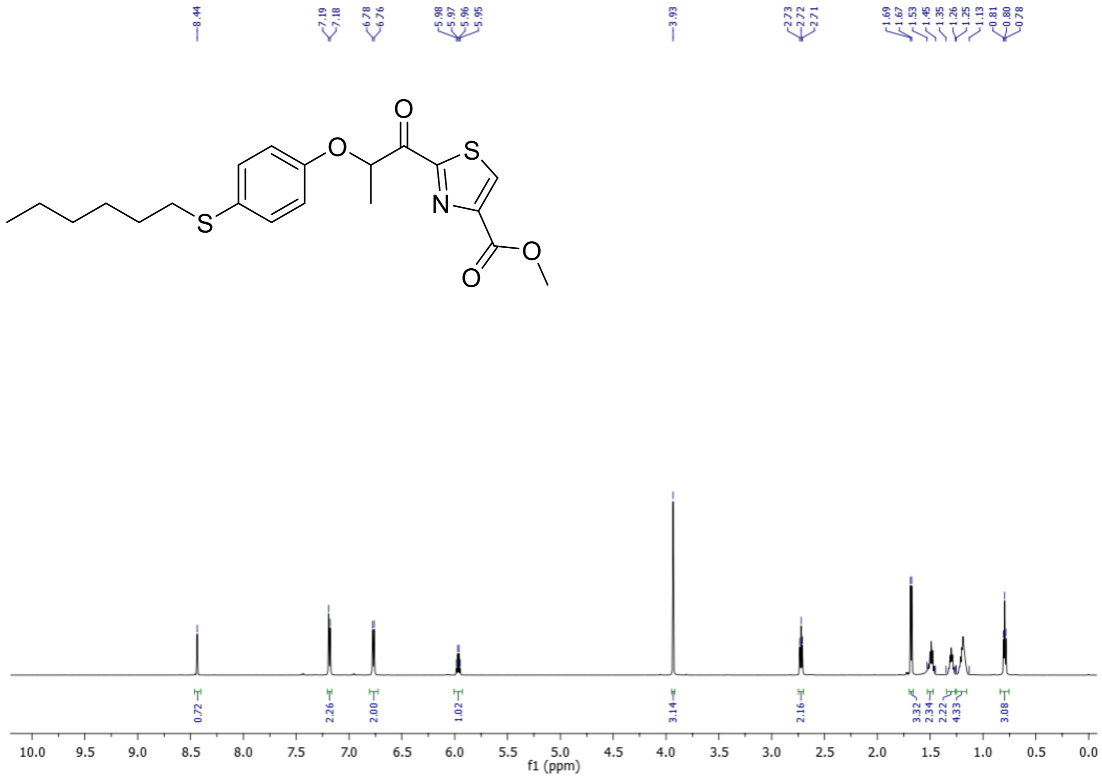

b. ^{13}C-NMR (150 MHz) of 17j in $CDCl_3$

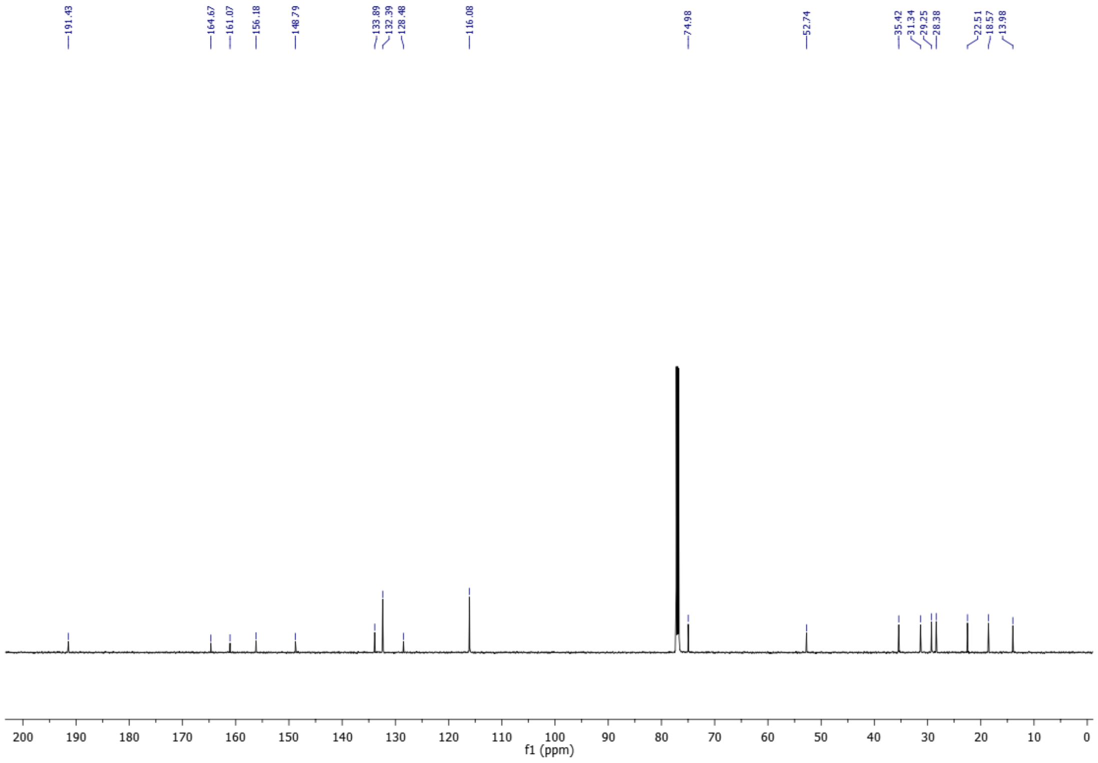

Figure S67. 18a

a. ^{1}H-NMR (400 MHz) of 18a in $CDCl_3$

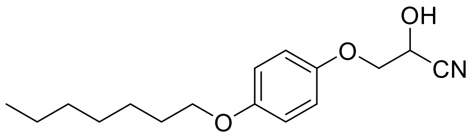

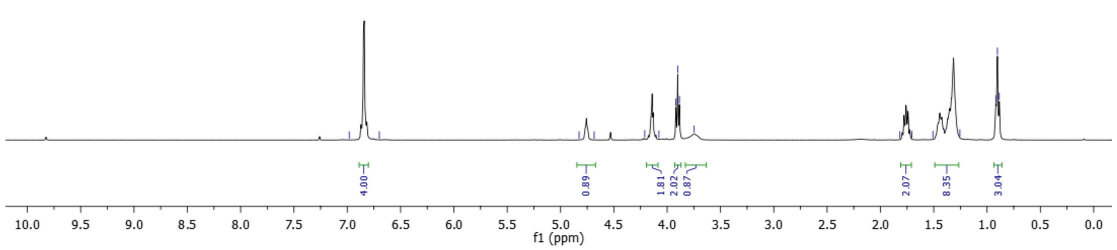

b. ^{13}C-NMR (100 MHz) of 18a in $CDCl_3$

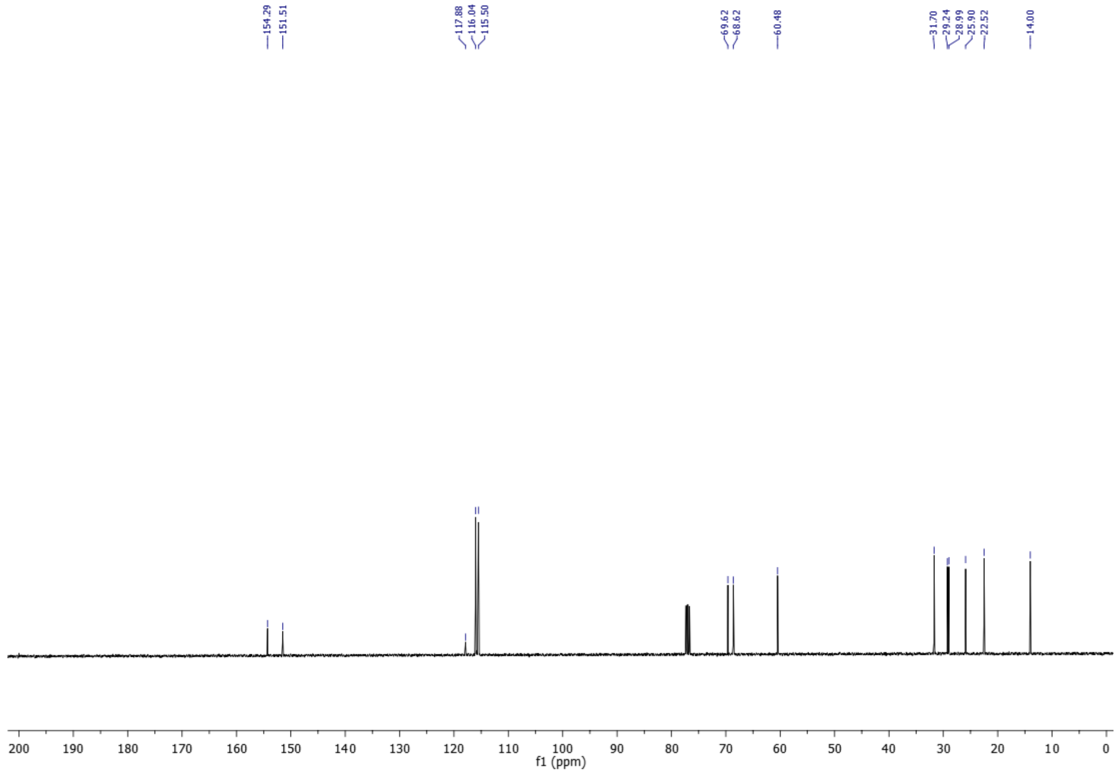

Figure S68. 18b

a. ^{1}H-NMR (400 MHz) of 18b in $CDCl_3$

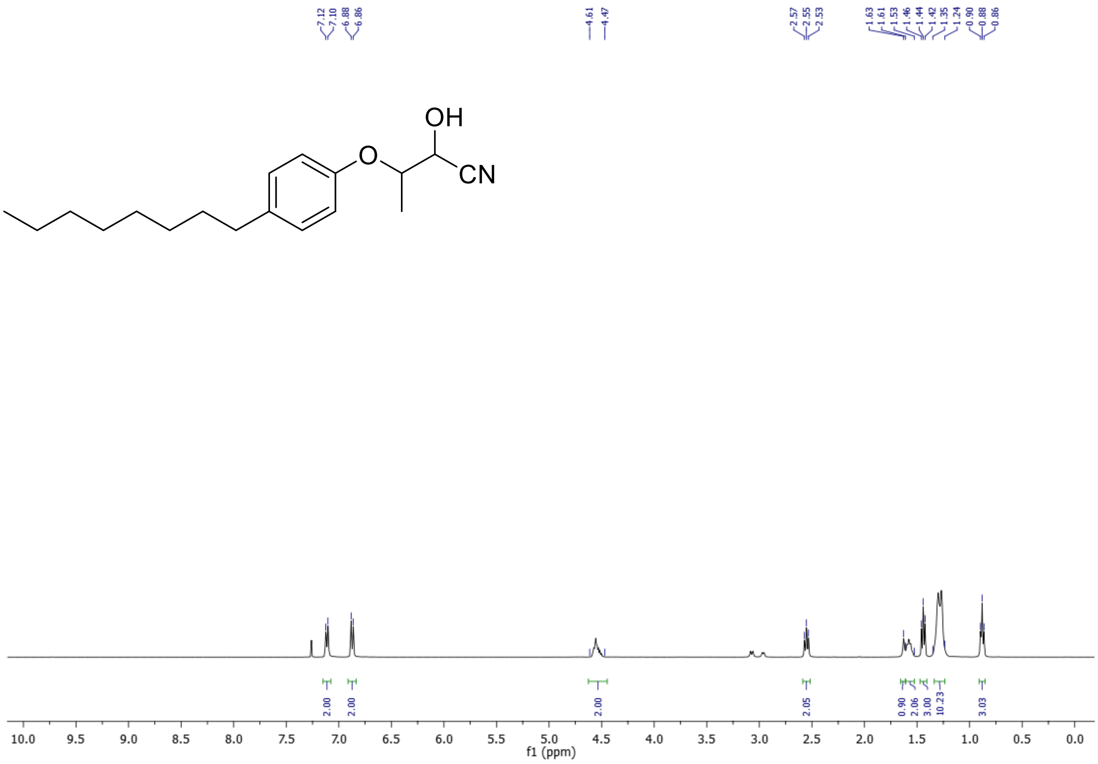

b. ^{13}C-NMR (100 MHz) of 18b in $CDCl_3$

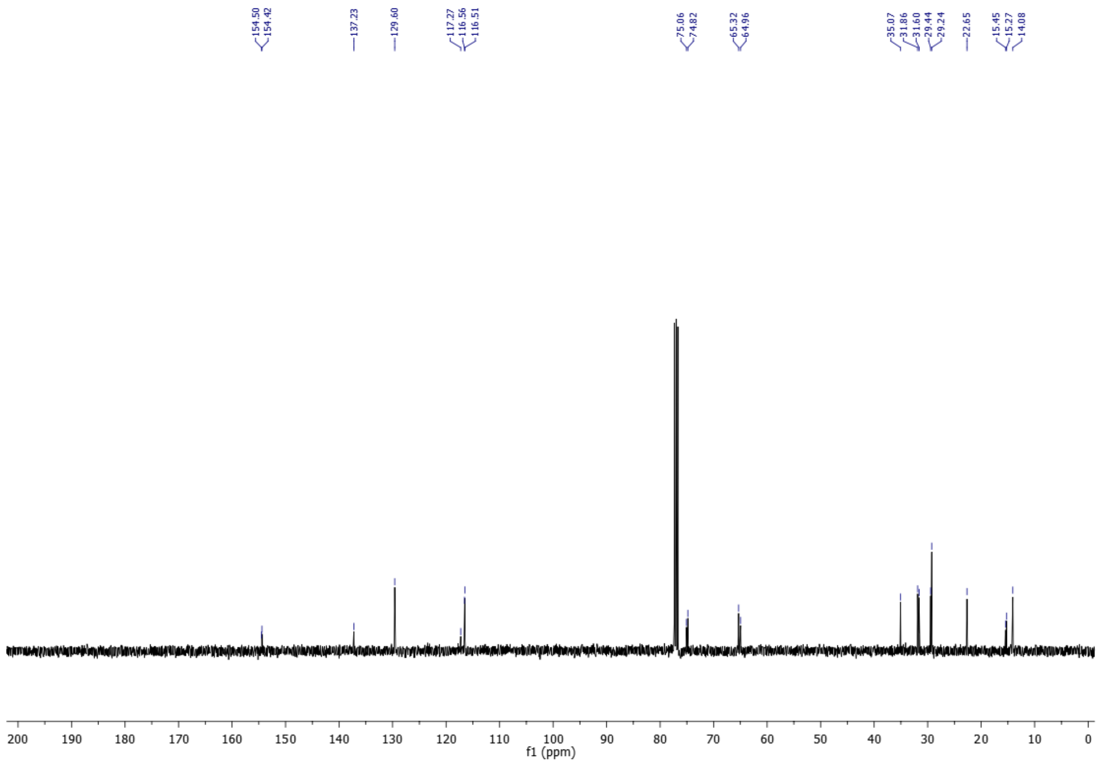

Figure S69. 18c

a. ^{1}H-NMR (400 MHz) of 18c in $CDCl_3$

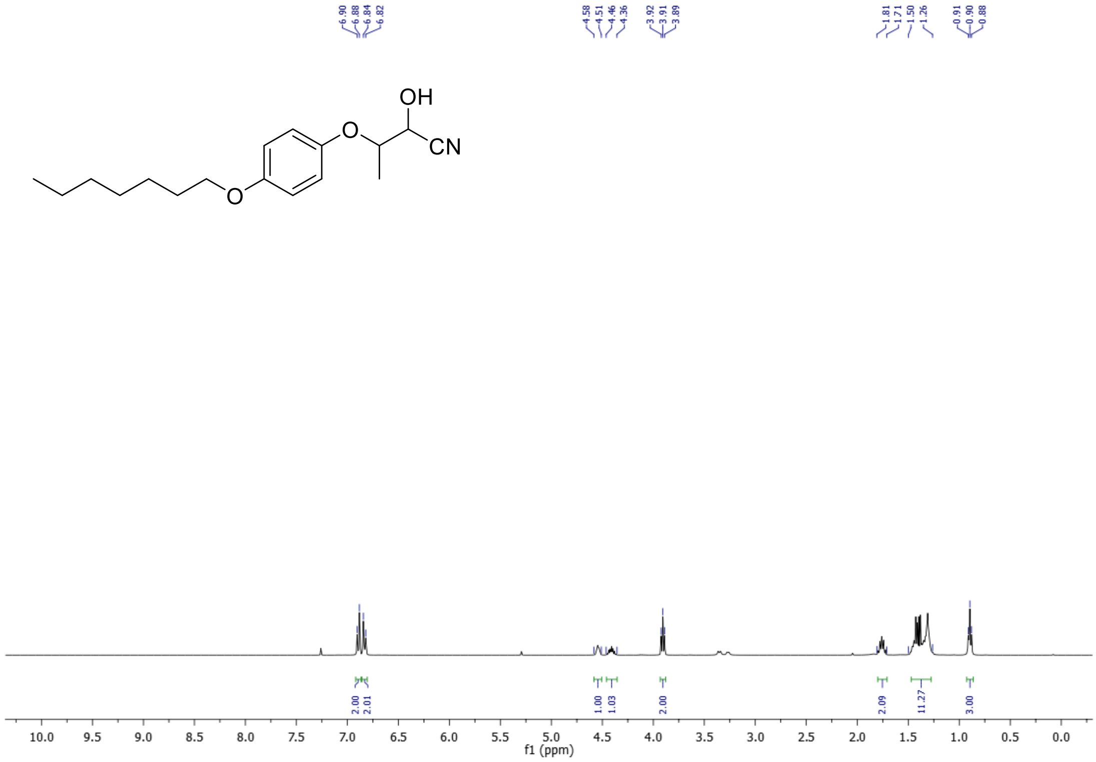

b. ^{13}C-NMR (100 MHz) of 18c in $CDCl_3$

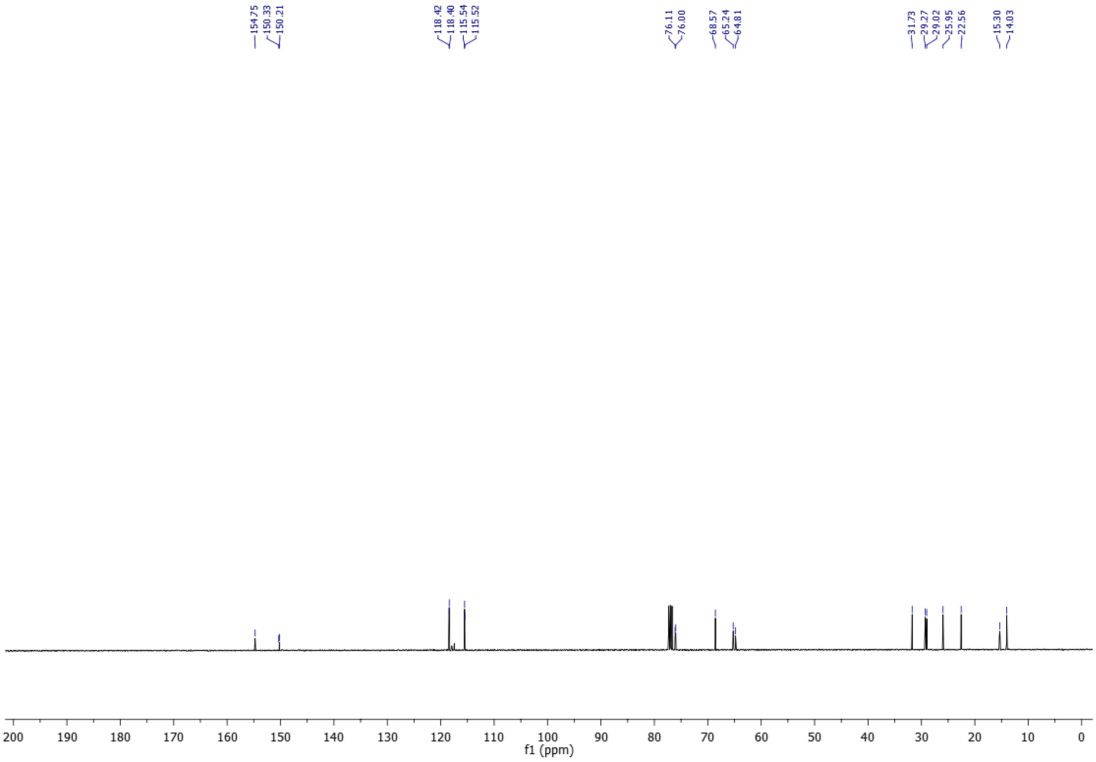

Figure S70. 18d

a. ^{1}H-NMR (200 MHz) of 18d in $CDCl_3$

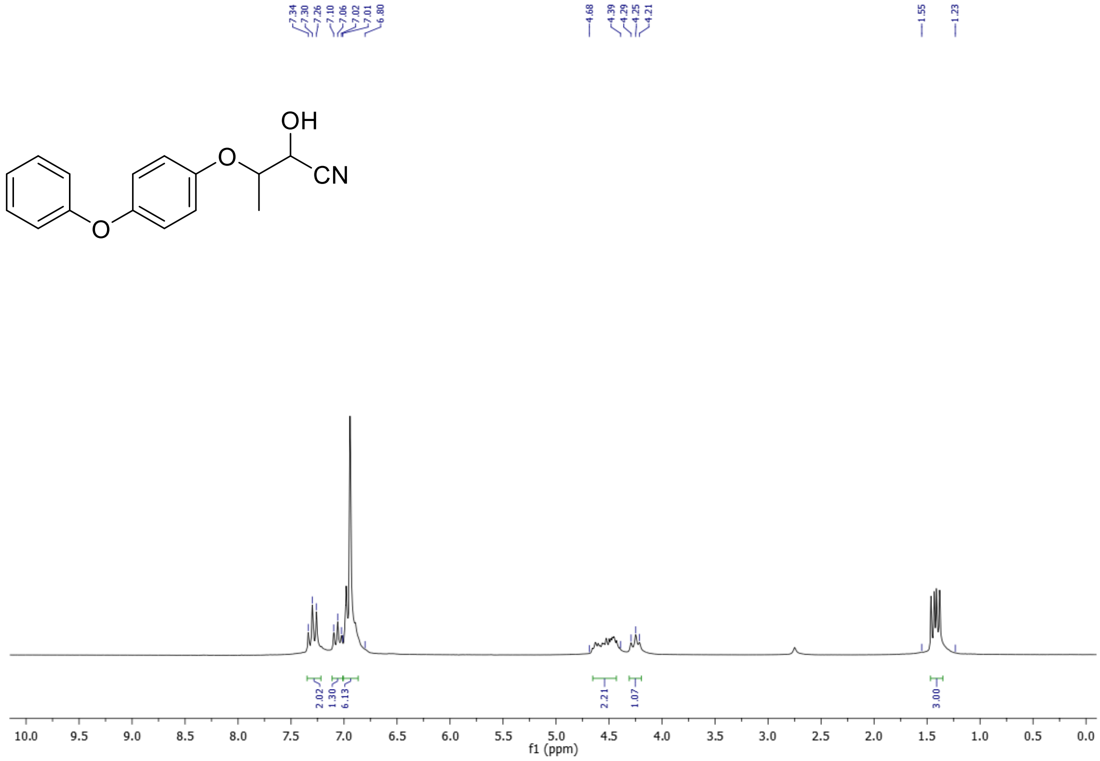

b. ^{13}C-NMR (50 MHz) of 18d in $CDCl_3$

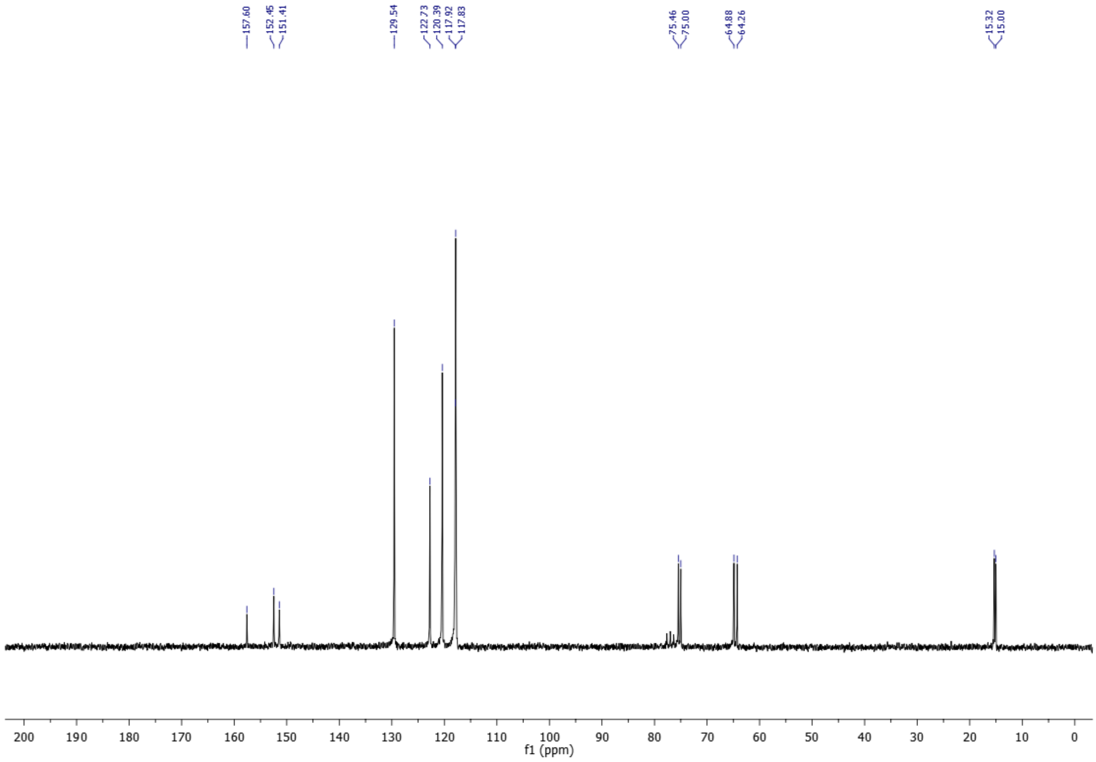

Supplemental references

1 Eisenberg, D., Schwarz, E., Komaromy, M. & Wall, R. Analysis of membrane and surface protein sequences with the hydrophobic moment plot. *J Mol Biol* **179**, 125-142 (1984). https://doi.org:10.1016/0022-2836(84)90309-7

2 Liu, Z. *et al.* Blood-Brain Barrier Permeable and NO-Releasing Multifunctional Nanoparticles for Alzheimer's Disease Treatment: Targeting NO/cGMP/CREB Signaling Pathways. *J Med Chem* **64**, 13853-13872 (2021). https://doi.org:10.1021/acs.jmedchem.1c01240

3 Kokotos, G. *et al.* Inhibition of group IVA cytosolic phospholipase A2 by thiazolyl ketones in vitro, ex vivo, and in vivo. *J Med Chem* **57**, 7523-7535 (2014). https://doi.org:10.1021/jm500192s

4 Sarges, R. *et al.* Glucose transport-enhancing and hypoglycemic activity of 2-methyl-2-phenoxy-3-phenylpropanoic acids. *J Med Chem* **39**, 4783-4803 (1996). https://doi.org:10.1021/jm950364f

5 Lawrence, M. S. *et al.* Discovery and saturation analysis of cancer genes across 21 tumour types. *Nature* **505**, 495-501 (2014). https://doi.org:10.1038/nature12912

6 Vogelstein, B. *et al.* Cancer genome landscapes. *Science* **339**, 1546-1558 (2013). https://doi.org:10.1126/science.1235122

7 Zack, T. I. *et al.* Pan-cancer patterns of somatic copy number alteration. *Nature Genetics* **45**, 1134-1140 (2013). https://doi.org:10.1038/ng.2760

8 Wagner, A. H. *et al.* DGIdb 2.0: mining clinically relevant drug-gene interactions. *Nucleic Acids Res* **44**, D1036-1044 (2016). https://doi.org:10.1093/nar/gkv1165
