# Supplementary material for: Next generation thiazolyl ketone inhibitors of cytosolic phospholipase A2 α for targeted cancer therapy
Source: Nat Commun. 2025 Jan 2;16:164. doi: 10.1038/s41467-024-55536-9 (PMC11696576; doi:10.1038/s41467-024-55536-9)
Supplement: Supplementary file 2 — Description of Additional Supplementary Files [file 41467_2024_55536_MOESM2_ESM.pdf]

Description of Additional Supplementary Files

Supplementary Movie 1. Representative trajectory of the 200 ns MD simulation of GK420 in the $cPLA_2\alpha$ active site. The carbon atoms of GK420 and $cPLA_2\alpha$ are shown in black and green, respectively. The amino acid residues shown in the movie are identical to those shown in Fig. S2b.

Supplementary Movie 2. Representative trajectory of the 200 ns MD simulation of GK420 acid in the $cPLA_2\alpha$ active site. The carbon atoms of GK420 acid and $cPLA_2\alpha$ are shown in black and green, respectively. Blue dashed lines indicate hydrogen bonds (distance cutoff: >3.2 Å and angle cutoff <135°). The amino acid residues shown in the movie are identical to those shown in Fig. 4b.
