# Supplementary material for: Next generation thiazolyl ketone inhibitors of cytosolic phospholipase A2 α for targeted cancer therapy
Source: Nat Commun. 2025 Jan 2;16:164. doi: 10.1038/s41467-024-55536-9 (PMC11696576; doi:10.1038/s41467-024-55536-9)
Supplement: Supplementary file 5 — Reporting Summary [file 41467_2024_55536_MOESM5_ESM.pdf]

nature portfolio

Corresponding author(s): Berit Johansen
George Kokotos

Last updated by author(s): Dec 16, 2024

Reporting Summary

Nature Portfolio wishes to improve the reproducibility of the work that we publish. This form provides structure for consistency and transparency in reporting. For further information on Nature Portfolio policies, see our Editorial Policies and the Editorial Policy Checklist.

Statistics

For all statistical analyses, confirm that the following items are present in the figure legend, table legend, main text, or Methods section.

n/a	Confirmed	
☐	☒	The exact sample size (*n*) for each experimental group/condition, given as a discrete number and unit of measurement
☒	☐	A statement on whether measurements were taken from distinct samples or whether the same sample was measured repeatedly
☐	☒	The statistical test(s) used AND whether they are one- or two-sided<br>*Only common tests should be described solely by name; describe more complex techniques in the Methods section.*
☐	☒	A description of all covariates tested
☐	☒	A description of any assumptions or corrections, such as tests of normality and adjustment for multiple comparisons
☐	☒	A full description of the statistical parameters including central tendency (e.g. means) or other basic estimates (e.g. regression coefficient) AND variation (e.g. standard deviation) or associated estimates of uncertainty (e.g. confidence intervals)
☐	☒	For null hypothesis testing, the test statistic (e.g. *F*, *t*, *r*) with confidence intervals, effect sizes, degrees of freedom and *P* value noted<br>*Give P values as exact values whenever suitable.*
☒	☐	For Bayesian analysis, information on the choice of priors and Markov chain Monte Carlo settings
☒	☐	For hierarchical and complex designs, identification of the appropriate level for tests and full reporting of outcomes
☐	☒	Estimates of effect sizes (e.g. Cohen's *d*, Pearson's *r*), indicating how they were calculated

Our web collection on statistics for biologists contains articles on many of the points above.

Software and code

Policy information about availability of computer code

Data collection	Lightycyler v1.1.0.1320,<br>Novoexpress v1.5.0,<br>CytExpert v2.5,<br>MultiQuant 3.0.2 and PeakView 2.1
Data analysis	ChemOffice Ultra 11.0<br>Maestro and Glide (Schrödinger, Inc., NY, USA)<br>NAMD 3.0<br>PoseView implemented in Protein Plus (https://proteins.plus/).<br>qPCR:LinReg v2021.2, Excel v2409;<br>RNA-Seq: Eclipsebio v1 proprietary software pipeline in R utilizing: umitools (v1.1.1), cutadapt (v3.2), STAR (v2.7.7a), umitools (v1.1.1), DESeq2, plotyl.js (v2.12.2), Bioconductor package clusterProfler (enrichGO and enrichKEGG)<br>Cell line screening: R utilizing pvclust, corrplot, igraph, and Piano package 10<br>SigmaPlot v14.0<br>GraphPad Prism v6 and v10<br>MultiQuant 3.0.2.

For manuscripts utilizing custom algorithms or software that are central to the research but not yet described in published literature, software must be made available to editors and reviewers. We strongly encourage code deposition in a community repository (e.g. GitHub). See the Nature Portfolio guidelines for submitting code & software for further information.

Data

Policy information about availability of data

All manuscripts must include a data availability statement. This statement should provide the following information, where applicable:

- Accession codes, unique identifiers, or web links for publicly available datasets
- A description of any restrictions on data availability
- For clinical datasets or third party data, please ensure that the statement adheres to our policy

The RNA-seq dataset that was generated in this study is available in the GEO repository with access code GSE251999 (https://www.ncbi.nlm.nih.gov/geo/query/acc.cgi?acc=GSE251999). Source data for figures are provided with the paper or available upon request.

Research involving human participants, their data, or biological material

Policy information about studies with human participants or human data. See also policy information about sex, gender (identity/presentation), and sexual orientation and race, ethnicity and racism.

Reporting on sex and gender	No reporting on sex.
Reporting on race, ethnicity, or other socially relevant groupings	No reporting on race, ethnicity or relevant groupings
Population characteristics	No population characteristics reported
Recruitment	Healthy blood donors
Ethics oversight	Regionale komiteer for medisinsk og helsefaglig forskningsetikk (REK)#2016/553.

Note that full information on the approval of the study protocol must also be provided in the manuscript.

Field-specific reporting

Please select the one below that is the best fit for your research. If you are not sure, read the appropriate sections before making your selection.

☒ Life sciences ☐ Behavioural & social sciences ☐ Ecological, evolutionary & environmental sciences

For a reference copy of the document with all sections, see nature.com/documents/nr-reporting-summary-flat.pdf

Life sciences study design

All studies must disclose on these points even when the disclosure is negative.

Sample size	Sample size determination was selected per method, and reported in the methods section
Data exclusions	Where appropriate, exclusion with associated criteria is reported in the methods section
Replication	For cell lines of interest the cancer cell line screen data was verified by replication with independently purchased cell lines and reagents.
Randomization	Studies were not randomized
Blinding	Studies were not blinded

Reporting for specific materials, systems and methods

We require information from authors about some types of materials, experimental systems and methods used in many studies. Here, indicate whether each material, system or method listed is relevant to your study. If you are not sure if a list item applies to your research, read the appropriate section before selecting a response.

Materials & experimental systems

n/a	Involved in the study
x	Antibodies
	x Eukaryotic cell lines
x	Palaeontology and archaeology
x	Animals and other organisms
x	Clinical data
x	Dual use research of concern
x	Plants

Methods

n/a	Involved in the study
x	ChIP-seq
	x Flow cytometry
x	MRI-based neuroimaging

Eukaryotic cell lines

Policy information about cell lines and Sex and Gender in Research

Cell line source(s)	CCRF-CEM (t# CCL-119) and Jurkat E6.1 (#TIB-152) cell lines were from American Type Culture Collection (ATCC, Manassas, VA, USA) while HL-60 and MV-4-11 cells were a kind gift from Prof. Bjørn Tore Gjertsen (University of Bergen, Norway). All cell lines used in the cancer cell line screen were licensed from the ATCC
Authentication	The cell lines were not authenticated
Mycoplasma contamination	The cell lines were confirmed to be negative for mycoplasm.
Commonly misidentified lines (See ICLAC register)	No commonly misidentified cell lines were used outside of the cancer cell line screening

Plants

Seed stocks	*Report on the source of all seed stocks or other plant material used. If applicable, state the seed stock centre and catalogue number. If plant specimens were collected from the field, describe the collection location, date and sampling procedures.*
Novel plant genotypes	*Describe the methods by which all novel plant genotypes were produced. This includes those generated by transgenic approaches, gene editing, chemical/radiation-based mutagenesis and hybridization. For transgenic lines, describe the transformation method, the number of independent lines analyzed and the generation upon which experiments were performed. For gene-edited lines, describe the editor used, the endogenous sequence targeted for editing, the targeting guide RNA sequence (if applicable) and how the editor was applied.*
Authentication	*Describe any authentication procedures for each seed stock used or novel genotype generated. Describe any experiments used to assess the effect of a mutation and, where applicable, how potential secondary effects (e.g. second site T-DNA insertions, mosiacism, off-target gene editing) were examined.*

Flow Cytometry

Plots

Confirm that:

- [x] The axis labels state the marker and fluorochrome used (e.g. CD4-FITC).
- [x] The axis scales are clearly visible. Include numbers along axes only for bottom left plot of group (a 'group' is an analysis of identical markers).
- [x] All plots are contour plots with outliers or pseudocolor plots.
- [x] A numerical value for number of cells or percentage (with statistics) is provided.

Methodology

Sample preparation	PBMCs were isolated from whole blood
Instrument	CytoFlex from Beckman coulter or NovoCyte from ACEA Biosciences
Software	CytExpert 2.4 or Novoexpess Software v1.5.0 from Aglent Technologies
Cell population abundance	Flow-assisted cell sorting: preliminary gate based on FSC-A vs BSC-A (40.26%). Secondary gate based on EGFP-H-compensated- 8.29% +ve (cPLA2sh) and 8.96% +ve cells (NTC-sh). Post-sort populations were considered to be predominantly GFP+ve but this was not empirically determined.
Gating strategy	Analysis of cell viability in T-cells.:preliminary gates based on FSC-H and SSC-H were used to isolate main PBMC population (50-60% of events). Gating was determined based on a control sample to define CD3+ and PI positive populations. This gate was applied to drug-treated populations to measure the proportion of CD3+ PI+ cells.

Analysis of ROS and PI staining in T-ALL cell lines: preliminary gates were based on FSC-H and SSC-H to exclude cell debris. Gates were established on the control sample to define a PI + or ROS+ population of <5% , this gate was applied to the drug treated or lentivirus expressing samples to measure the relative proportion of ROS+ or PI + cells.
Transduced cells were defined in the FITC (GFP) channel by setting the analysis gate to give <0.1% GFP+ve cells in the non-transduced control. This gate was applied to define transduced cells for analysis of ROS or PI and to measure the efficiency.

☒ Tick this box to confirm that a figure exemplifying the gating strategy is provided in the Supplementary Information.
